# Dual organism design cycle reveals small subunit substitutions that improve [NiFe] hydrogenase hydrogen evolution

**DOI:** 10.1186/1754-1611-7-17

**Published:** 2013-07-02

**Authors:** Isaac T Yonemoto, Christopher W Matteri, Thao Amy Nguyen, Hamilton O Smith, Philip D Weyman

**Affiliations:** 1 J. Craig Venter Institute, 10355 Science Center Dr, San Diego, CA 92121, USA

**Keywords:** Hydrogenase, Cyanobacteria, Iron-sulfur Cluster, *Alteromonas Macleodii* “Deep Ecotype”

## Abstract

**Background:**

Photosynthetic microorganisms that directly channel solar energy to the production of molecular hydrogen are a potential future biofuel system. Building such a system requires installation of a hydrogenase in the photosynthetic organism that is both tolerant to oxygen and capable of hydrogen production. Toward this end, we have identified the [NiFe] hydrogenase from the marine bacterium *Alteromonas macleodii* “Deep ecotype” that is able to be heterologously expressed in cyanobacteria and has tolerance to partial oxygen. The *A. macleodii* enzyme shares sequence similarity with the uptake hydrogenases that favor hydrogen uptake activity over hydrogen evolution. To improve hydrogen evolution from the *A. macleodii* hydrogenase, we examined the three Fe-S clusters found in the small subunit of many [NiFe] uptake hydrogenases that presumably act as a molecular wire to guide electrons to or from the active site of the enzyme. Studies by others altering the medial cluster of a *Desulfovibrio fructosovorans* hydrogenase from 3Fe-4S to 4Fe-4S resulted in two-fold improved hydrogen evolution activity.

**Results:**

We adopted a strategy of screening for improved hydrogenase constructs using an *Escherichia coli* expression system before testing in slower growing cyanobacteria. From the *A. macleodii* enzyme, we created a mutation in the gene encoding the hydrogenase small subunit that in other systems is known to convert the 3Fe-4S medial cluster to 4Fe-4S. The medial cluster substitution did not improve the hydrogen evolution activity of our hydrogenase. However, modifying both the medial cluster and the ligation of the distal Fe-S cluster improved *in vitro* hydrogen evolution activity relative to the wild type hydrogenase by three- to four-fold. Other properties of the enzyme including thermostability and tolerance to partial oxygen did not appear to be affected by the substitutions.

**Conclusions:**

Our results show that substitution of amino acids altering the ligation of Fe-S clusters in the *A. macleodii* [NiFe] uptake hydrogenase resulted in increased hydrogen evolution activity. This activity can be recapitulated in multiple host systems and with purified protein. These results validate the approach of using an *E. coli*-cyanobacteria shuttle system for enzyme expression and improvement.

## Background

Hydrogen produced photobiologically from sunlight and water is a potential future biofuel and is an alternative to traditional carbon-based fuels [1-4]. Enzymes that produce hydrogen include nitrogenases and hydrogenases, the latter comprising the unrelated families of [Fe-Fe] hydrogenases, Fe-only hydrogenases, and [NiFe] hydrogenase enzymes [5]. Cyanobacteria such as *Synechococcus elongatus* are ideal host strains for a biological system that converts solar energy to hydrogen because they are genetically tractable and easily cultured [6]. Because cyanobacteria also perform oxygenic photosynthesis, the oxygen produced will inactivate most hydrogenases [7,8]. Therefore, installing a hydrogenase that is less sensitive to oxygen is a primary design constraint of this system. Among the hydrogenase families, oxygen tolerance has been described only among the [NiFe] hydrogenases, although not all [NiFe] hydrogenases are oxygen tolerant [9-11]. There is some evidence the native cyanobacterial bidirectional hydrogenase may be capable of hydrogen production in the presence of oxygen [12], but the energetic consequences of using NADPH as an electron donor makes it less attractive.

We previously reported the discovery and characterization of a novel hydrogenase isolated from Sargasso Sea environmental DNA with high identity to the stable [NiFe] uptake hydrogenase from *Thiocapsa roseopersicina*[13]. A closely-related sequence was later identified in the *Alteromonas macleodii ‘*Deep ecotype’ genome (hereafter referred to as the *A. macleodii* hydrogenase), and was characterized as active in the presence of 2% oxygen [14]. The DNA encoding the enzyme was then cloned and used to express active enzyme in both *Escherichia coli*[15] and *S. elongatus*[16]. Expressing active *A. macleodii* hydrogenase in both *E. coli* and cyanobacteria requires the co-expression of at least nine additional maturation factors to properly assembly an active enzyme [17]. Due to the complex maturation requirements of [NiFe] hydrogenases, only a few other examples exist of heterologous expression in *E. coli*[18-20]. The dual expression system is ideal for rapidly generating variant hydrogenases using powerful *E. coli* genetic tools, quickly screening for desirable properties in *E. coli*, and confirming the persistence of the property in the final target cyanobacterial host.

**Figure 1:**
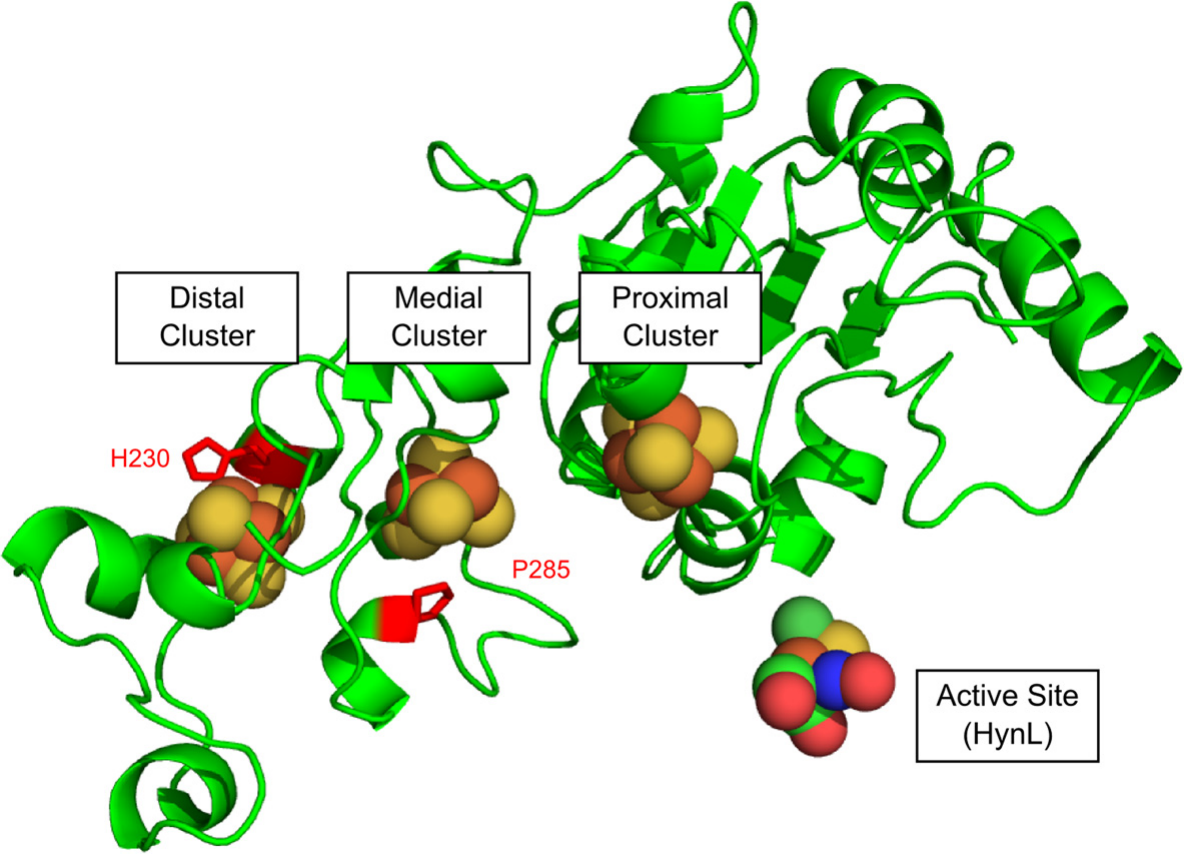
**Structural model of the *A. macleodii *[NiFe] uptake hydrogenase small subunit HynS, highlighting the three Fe-S clusters, and residues His230 and Pro285, which were targeted for substitution to cysteine residues.** For reference, the location of the active site is shown, but the rest of the large subunit structure was omitted to simplify viewing of the small subunit. The structural model was generated by Phyre using structure PDB: 1h2a as a model [27,28].

Although some uptake [NiFe] hydrogenases are oxygen-tolerant, they are known to be generally directionally biased against hydrogen evolution [21,22]. Enzyme bias in uptake hydrogenases appears to have been altered in separate experiments investigating the ligation of Fe-S clusters in the small subunit of [NiFe] hydrogenases [23,24]. These Fe-S clusters are believed to function as a molecular wire guiding electrons out of the active site and to a biological electron carrier molecule (Figure 1) [10,25,26]. Conversely, in a hydrogen-production scenario, these Fe-S clusters would predominantly operate in the reverse direction, channeling electrons into the active site from an electron donor at the enzyme surface.

At the medial Fe-S cluster, uptake [NiFe] hydrogenases have conserved amino acid residues deviating from the canonical 4-cysteine ligation characteristic of Fe-S cubane clusters. In *A. macleodii* and almost all members of the uptake NiFe hydrogenase family, the medial cluster features a proline in the place of a ligating cysteine resulting in the installation of a 3Fe-4S cluster instead of a 4Fe-4S cluster [26]. Interestingly, the closely related small subunits of the H_2_-sensing regulatory hydrogenases, which show almost no hydrogen evolution, and the NiFeSe hydrogenases, which show more hydrogen evolution activity [29], both feature cysteinyl ligations at the homologous amino acid position [24,30]. The unrelated small subunit of the F420-reducing hydrogenase, which also features 3 4Fe-4S clusters, has a 4Fe-4S cluster for its medial cluster [31]. In *A. macleodii* hydrogenase, and uptake hydrogenases in general, the cluster distal to the active site is a 4Fe-4S cluster featuring an unusual iron-histidinyl ligation that is conserved in most [NiFe] hydrogenases. While its function is unknown, the unusual ligation may be related to intramolecular electron transfer [23,32].

In *Desulfovibrio fructosovorans*, substitution of the proline residue associated with the medial cluster to cysteine (*Df* HynA P238C) resulted in approximately 30% reduction of hydrogen uptake activity and a nearly two-fold increase in evolution activity [24]. Separately, when the histidine residue at the distal cluster was changed to cysteine (*Df* HynA H184C), hydrogen uptake activity was nearly abolished while evolution activity was only 50% reduced [23]. We produced enzyme variants featuring substitutions in the *A. macleodii* hydrogenase at sites homologous to the *D. fructosovorans* uptake hydrogenase substitutions. The variants of the *A. macleodii* enzyme (HynSL - NCBI accession codes: YP_004425481.1, YP_004425482.1) featuring the corresponding substitutions based on homology (HynS H230C, P285C^1^) were created both individually and combined in an effort to find an enzyme with improved hydrogen evolution activity for production of hydrogen in cyanobacteria.

Taking advantage of our previously reported dual-species system, these modified enzymes were first screened in *E. coli* to assess their enzymatic properties. Initial screens with *E. coli* extracts indicated that the doubly-substituted enzyme had the highest hydrogen evolving activity. Purification of the enzyme indicated that this activity was intrinsic to the enzyme variant, and not due to enzyme titer or host factors persistent in crude lysate. Finally, we moved the expression system to the cyanobacterium *S. elongatus*, which recapitulated the relative activities of the enzymes, validating our general strategy of using *E. coli* as a rapid-turnover model system.

## Results and discussion

### *Heterologous expression for [NiFe] hydrogenase in* E. coli

**Figure 2:**
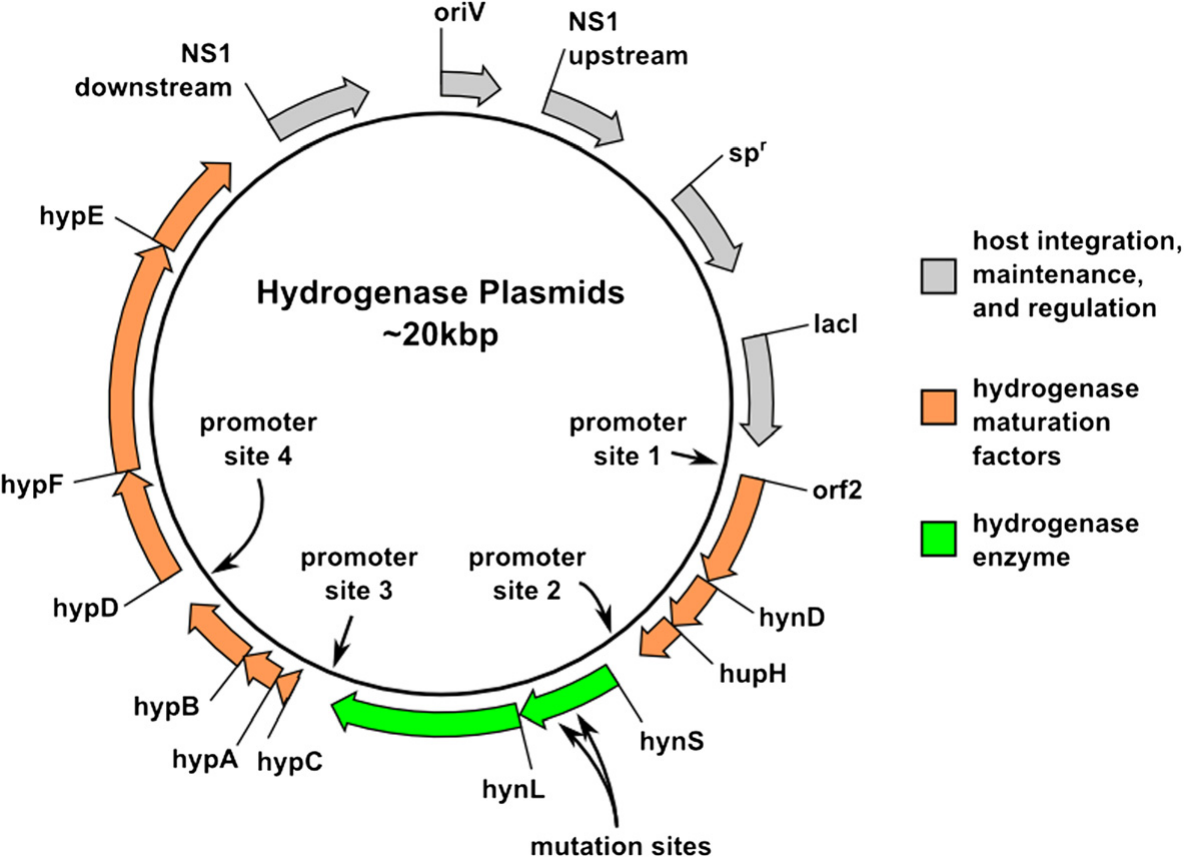
**A schematic diagram of the *A. macleodii *hydrogenase expression vectors used in our study.** Detailed maps and sequences for all vectors are available in the supplemental information.

Heterologous expression of the *A. macleodii* hydrogenase has been previously reported in both *E. coli*[15] and the cyanobacterium *S. elongatus*[16]. These reports address general issues of aerobic expression of the *A. macleodii* hydrogenase, as in both these reports, aerobic handling was used throughout, established as safe in the original report on the enzyme [14]. We have previously determined that the minimal gene set required for optimal activity in *E. coli* consists of the hydrogenase structural genes, *hynS* and *hynL*, the six pleiotropic maturation factors, *hypABCDEF*, the endoprotease *hynD*, and two additional genes, *hupH* and *orf2*. Additionally, four promoter sites can be used to add additional transcriptional regulation throughout the gene cluster (Figure 2). Expression plasmids used initially consisted of these essential 11 genes driven by a single P_TRC_ promoter installed in “promoter site 1” (Figure 2). The P_TRC_ promoter functions well in both *E. coli* and cyanobacteria [33]. Expression plasmids also contain a spectinomycin resistance gene and *lacI* gene that function in both *E. coli* and cyanobacteria. Variants containing a P_T7_ promoter or P_TRC_ promoters at all four “promoter site”s were also generated. For integration into cyanobacteria, these features are flanked by 1-kb of *S. elongatus* DNA on either side for homologous recombination into neutral site 1 (NS1) [34]. Amino acid substitutions were generated by PCR of the operon using primers which encode for mutations corresponding to the substitutions, followed by assembly with an empty vector. Amino acid substitutions of HynS can be transferred by restriction digest and reassembly process. In this process, the wild type sequence is excised using restriction enzymes whose sites flank the region of interest and the new sequence is installed by reassembly of the vector with PCR products featuring the corresponding nucleic acid sequence mutations (see Methods).

### Evolution activity assay

**Figure 3:**
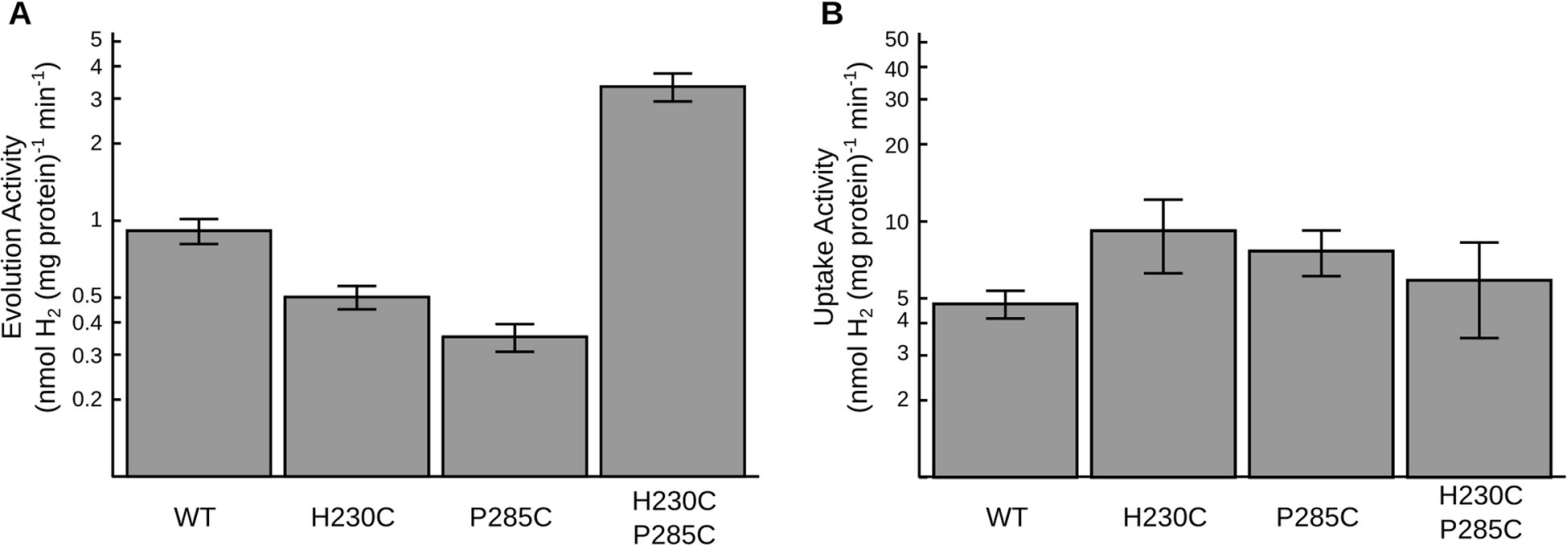
**Hydrogen evolution (A) and uptake (B) activities for lysates of *E. coli *strain FTD147 over-expressing wild type *A. macleodii *hydrogenase or substitutions H230C, P285C, and H230C/P285C.** Activities on both graphs are plotted on matching log scales over a 50-fold range to allow comparison of equivalent fold-changes. Error bars represent one standard error of the mean.

To test the effect of our substitutions on the hydrogen evolution activity, we performed methyl viologen hydrogen production assays on lysates of *E. coli* cultures expressing the wild type *A. macleodii* hydrogenase (WT), the *A. macleodii* hydrogenase bearing the individual substitutions H230C, P285C, or the doubly-substituted HynS H230C/P285C. In this assay, electrons are transferred to methyl viologen by oxidation of sodium dithionite, then from methyl viologen to hydrogenase. At the hydrogenase active site, these electrons are recombined with protons to generate molecular hydrogen, which undergoes a phase separation from solution and is detectable by gas chromatographic analysis (GC) of the assay vial headspace. Compared to wild type, lysates containing the singly-substituted enzymes had slightly lower H_2_ evolution activity. The H230C lysate had approximately half the activity of WT, and the P285C lysate had approximately one third the WT activity (Figure 3A). Lysates containing the H230C/P285C doubly-substituted enzyme, by contrast, exhibited a 3-4-fold increase in evolution activity compared to WT (Figure 3A).

### Uptake activity assay

To test the effect of our substitutions on hydrogen uptake, we performed a benzyl viologen hydrogen uptake assay on *E. coli* lysates expressing the wild type *A. macleodii* [NiFe] hydrogenase, and variants featuring the substitutions H230C, P285C, and H230C/P285C. In this reaction the hydrogenase catalyzes the dissociation of hydrogen to protons and electrons. The electrons are transferred to benzyl viologen, causing a color change detectable by spectrophotometry at 555 nm. Lysates containing the H230C and P285C enzymes each had slightly increased uptake activity relative to WT; H230C had nearly twice the uptake activity as WT while P285C had slightly less activity than H230C. The hydrogen uptake activity of lysate containing the doubly-substituted enzyme was similar to WT and within the range of experimental error (Figure 3B).

### Western blot

**Figure 4:**
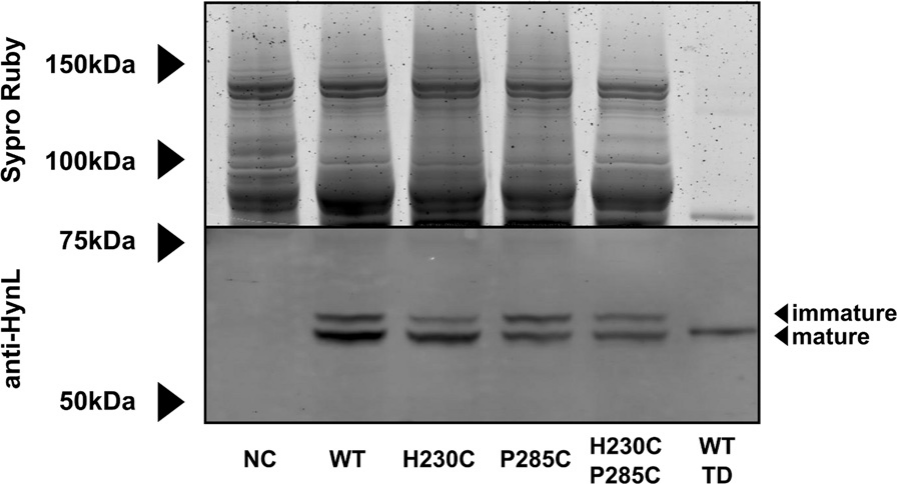
**Expression analysis for lysates of *E. coli *strain FTD147 with no hydrogenase construct (NC), over-expressing wild type *A. macleodii *hydrogenase enzyme (WT), or H230C, P285C, and H230C/P285C substitutions, and wild type hydrogenase subjected to thermal denaturation (WT TD).** Before loading, proteins were normalized for protein content at 5 mg mL^-1^. Associated enzyme activites can be found in Figure S1. Top: Sypro-Ruby stain of the gel, indicating approximately equivalent amounts of protein loaded per lane. Bottom: anti-HynL western blot of samples.

Because our activities are normalized against total protein from crude *E. coli* lysate, it was necessary to rule out the possibility that differences in hydrogenase expression levels were responsible for the observed differences in activity. Proteins in crude extracts were separated by SDS-PAGE and subjected to western blotting to estimate the relative expression of hydrogenase. Because the primary sequence of HynL is unaffected by the substitutions we made in HynS, we expect the relative intensity of resulting HynL bands to be a good indicator of enzyme titer. Lysates were diluted to 5 mg mL^-1^ total protein and enzyme activity was measured to ensure activities agreed with those measured previously (Additional file 1: Figure S2). To confirm approximately equivalent loading, a gel slice was stained with Sypro Ruby to illustrate protein content of loaded samples (Figure 4, top). Anti-HynL western blot (Figure 4, bottom) of the lysates shows two bands, presumably corresponding to the immature and the C-terminally processed mature forms of the enzyme subunit [35]. Identically-handled lysate was subjected to brief thermal denaturation (65°C, 10 min) and a second clearing step. Lysate treated in this manner retained nearly all activity (Additional file 1: Figure S2) but lost the larger HynL band in the western blot, lending support to the top band being the unprocessed, immature form of HynL. These western blots suggest that all variants were expressed at approximately equivalent levels and that there were no major defects in expression.

### Evolution assay on purified enzyme

**Figure 5:**
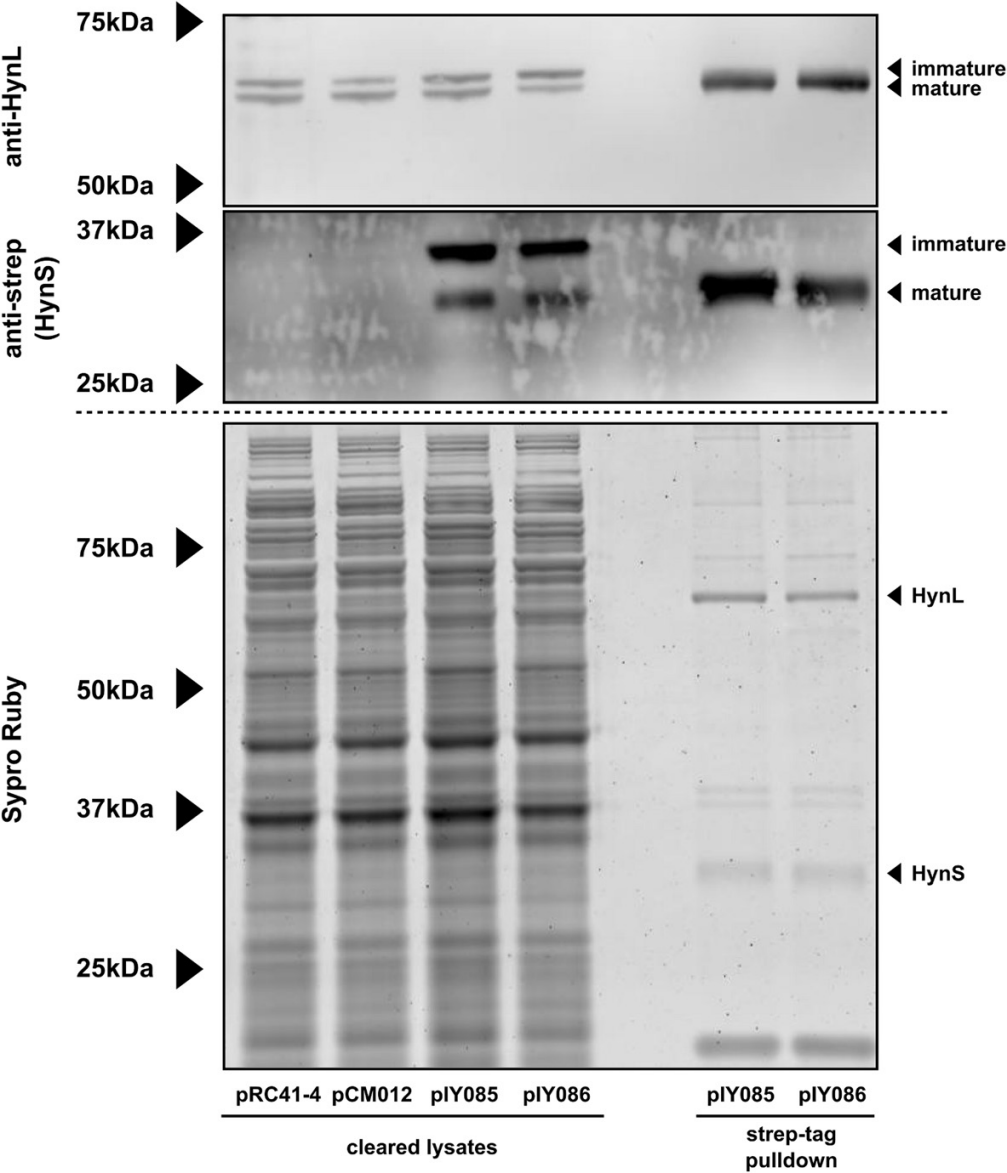
**Purification of hydrogenase from *E. coli *over-expressing wild type *A. macleodii *hydrogenase (pIY085) or H230C/P285C doubly-substituted enzyme (pIY086).** Associated enzyme activities can be found in Table S1. Samples are either cleared lysates (left) or Streptactin-purified proteins (right) as indicated at the bottom of the figure. Top: anti-HynL western blot. Middle: anti-Strep western blot, detecting HynS-strep protein. Bottom: Sypro-Ruby stain of a parallel gel. pRC41-4 and pCM012 are constructs expressing wild type and doubly-substituted enzymes, respectively, and are included as negative controls for the strep-tag blot. pIY085 and pIY086, respectively, are the equivalent constructs, except bearing a C-terminal strep-tag on the HynS protein.

To further test if the observed improvement in evolution activity found in the doubly-substituted enzyme is the result of an intrinsic change in enzymatic activity, we generated WT and doubly-substituted enzymes with strep-tags at the C-terminus of HynS. This tag was used in an affinity-purification strategy (Figure 5), and the resulting purified fraction was tested by the hydrogen evolution assay (Additional file 1: Table S1). Some impurities persist in the streptactin-mediated purification; we believe the major contamination in the SDS-PAGE of the strep-tagged protein to be immature small subunit lacking TAT-mediated cleavage, as well as trace amounts of high-molecular weight proteins that persist in the partially purified fractions.

Nonetheless, we found that purification afforded a more than 100-fold improved specific activity for both wild type and doubly-substituted enzymes. Furthermore, after parallel purification the doubly-substituted, strep-tagged enzyme still exhibited three-fold more activity than the wild type, strep-tagged enzyme, consistent with our observations using unpurified *E. coli* lysates in the hydrogen evolution assay (Figure 2A).

### Thermal and oxygen tolerance assays

**Figure 6:**
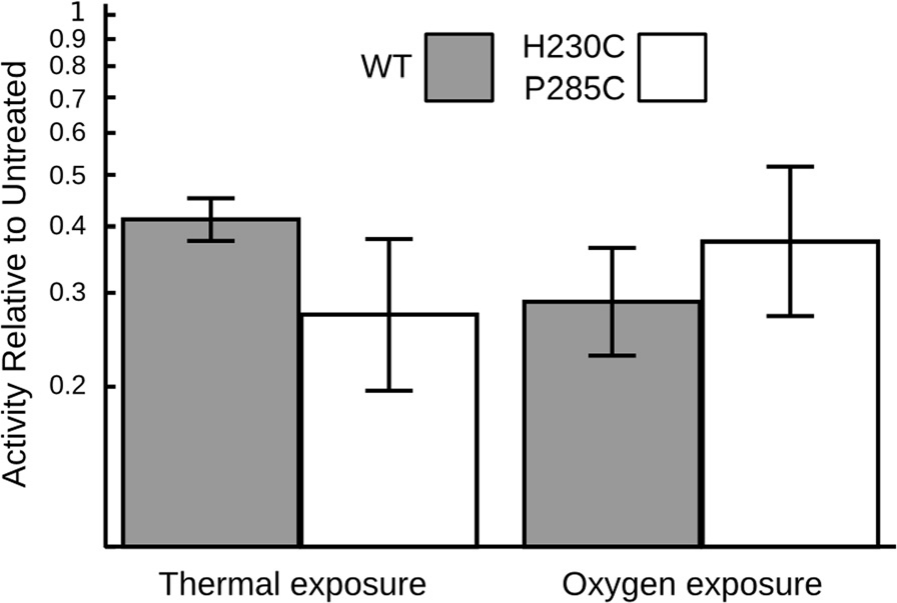
**Response of wild type and doubly-substituted *A. macleodii *hydrogenase to various environmental conditions.** Thermotolerance was measured as hydrogen evolution activity after denaturation at 85°C for 1 hour. Sensitivity to oxygen was measured by relative H-D exchange rate in the presence of 1% oxygen. Results are expressed as the ratio of activity remaining in the treated samples relative to the untreated controls, and plotted on a log scale over one decade. Error bars represent one geometric standard error of the geometric mean.

To test that the doubly-substituted enzyme did not gain improved evolution activity at the expense of other desirable intrinsic properties, we subjected the *E. coli* lysates to a thermal denaturation assay and an assay testing function in the presence of oxygen. We chose thermal denaturation (85°C for 1h) as a qualitative measure of structural perturbation and hypothesized that the doubly-substituted enzyme would exhibit less thermotolerance than the wild type enzyme due to its modifications. Other treatments were tested; for simplicity we report the harshest treatment we tested that exhibited > 10% activity. We also tested if the doubly-substituted enzymes would retain function in partial oxygen atmospheres as we had previously observed for the wild-type enzyme. We found that the doubly-substituted enzyme was not less thermotolerant than the WT enzyme within the range of experimental error. Substituting the two residues did not result in a significant defect in the ability to function in the presence of 1% oxygen (Figure 6).

### Relative performance in other bacterial strains

**Figure 7:**
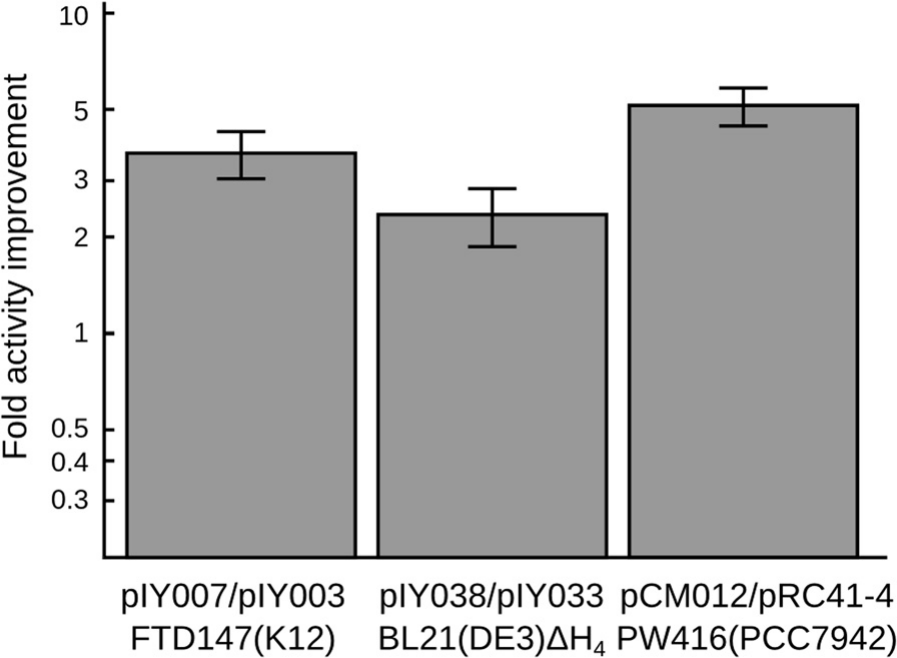
**Relative hydrogenase evolution activities of heterologous expression constructs bearing the wild type vs. doubly-substituted *A. macleodii *hydrogenase.** pIY007/pIY003 compares the doubly-substituted enzyme (DS) and the wild type (WT) enzyme produced by the hydrogenase operon as driven by P_TRC_ and transformed into the hydrogenase-free *E. coli* K12 strain FTD147. pIY038/pIY033 compares DS and WT enzymes produced by the hydrogenase operons as driven by P_T7_ and transformed into the hydrogenase-free *E. coli* BL21(DE3)ΔH_4_ derivative. pCM012/pRC41-4 compares the hydrogenase operons driven by 4 P_TRC_ promoters interspersed in the operon and integrated into an otherwise hydrogenase-free *S. elongatus* cyanobacterial strain. Relative activities are plotted on a log scale over a 50-fold range. Error bars represent one geometric standard error of the geometric mean.

To test the robustness of our observation that the doubly-substituted enzyme has improved hydrogen evolution activity, we expressed the enzyme with different promoters and in different host species. Plasmid pIY003 expresses the hydrogenase behind a single P_TRC_ promoter, with pIY007 as the corresponding plasmid bearing the doubly-substituted enzyme. Plasmid pIY033 expresses the hydrogenase gene cluster behind a single P_T7_ promoter with pIY038 as the corresponding plasmid bearing the doubly-substituted enzyme; pRC41-4 is a plasmid featuring the hydrogenase gene cluster supported by 4 P_TRC_ promoters with pCM012 as the corresponding plasmid bearing the doubly-substituted enzyme. While pIY003 and pIY007 were tested in the FTD147 strain, the pIY033/pIY038 pair was tested in an *E. coli* strain in which all of its hydrogenases were knocked out (BL21(DE3)Δ*hycG*Δ*hyaB*Δ*hybC*Δ*hyfG*, hereafter referred to as BL21(DE3)ΔH_4_) [18]. The pRC41-4 and pCM012 plasmids were tested in the cyanobacterial strain *S. elongatus* PCC 7942 PW416 (Δ*hoxYH*:: Em^r^) [16]. Our data indicate that *E. coli* strain FTD147 expressing the doubly-substituted enzyme from pIY007 showed 3.7±0.6-fold higher activity than the same strain expressing the wild-type enzyme from pIY003; *E. coli* strain BL21(DE3)ΔH_4_ expressing the doubly-substituted enzyme from pIY038 showed 2.3±0.8-fold higher activity than the same strain expressing the wild-type enzyme, and an *S. elongatus* strain expressing the doubly-substituted enzyme from pCM012 showed 5.1±0.7-fold higher activity than a strain expressing the wild-type enzyme (Figure 7). Thus, relative to the WT enzyme, the doubly-substituted enzyme consistently shows increased activity -- in both *E. coli* strains as well as in a cyanobacterial expression system.

By substituting two residues in the small subunit of *A. macleodii* [NiFe] uptake hydrogenase, we have increased its hydrogen evolution activity. To our knowledge this is the first reported instance of this doubly-substituted enzyme analyzed in the context of hydrogen evolution. In the homologous doubly-substituted enzyme from *D. fructosovorans* constructed by Dementin and co-workers [36], activity in the evolving direction was not reported. Comparing the homologous substitutions in *A. macleodii* and *D. fructosovorans* hydrogenases, we observe qualitatively different structure-activity relationships. Thus, the effect of these substitutions may not be generalizable across different members of the enzyme family. Indeed, the inverse experiment, changing a medial 4Fe-4S cluster to a 3Fe-4S cluster in the *Methanococcus janaschii* F_420_-reducing hydrogenase, resulted in severely impaired uptake, inconsistent with the loose pattern otherwise observed in uptake hydrogenases [31]. As such, others seeking to modify [NiFe] hydrogenase Fe-S cluster ligations should consider testing both single substitutions as well as the double substitution that are homologous to those discussed here when seeking an enzyme suitable for their needs.

The doubly-substituted *A. macleodii* hydrogenase we present here is an attractive launching point for future engineering of hydrogenases redesigned for use in photoautotrophs. The two substitutions have minimal effects on the thermotolerance of the enzyme, as measured by loss of activity after the harsh treatment of 85°C for 1 hour. Treatment at lower temperatures or shorter times did not cause appreciable loss of activity in either doubly-substituted or wild type enzymes (data not shown). Our observations suggest that the substitutions have not created severe structural changes, likely allowing further modification of the enzyme without disrupting function. Furthermore, the ability to function in the presence of partial oxygen, a critical property of photoautotrophic hydrogen production systems, appears to be unaffected and may be able to be improved by further modification.

Finally, because *A. macleodii* hydrogenase can be expressed as active enzyme in both *E. coli*[15] and in the photoautotrophic cyanobacterium *S. elongatus*[16], our strategy moving forward is to use expression in *E. coli* as a preliminary model system to rapidly generate and test promising modifications of the enzyme prior to experimentation in *S. elongatus*, which has a longer turnaround time for genetic manipulations. The H230C/P285C enzyme demonstrated the same qualitative performance over the wild type enzyme in extracts prepared from both *S. elongatus* and *E. coli*, validating this strategy. Initial attempts at in vivo activity were performed in both host species aerobically and anaerobically, but did not yield measurable hydrogen production. To that end, further modifications we are pursuing to achieve this important result include: improving the oxygen tolerance by engineering a substitution leading to a 4Fe-3S proximal Fe-S cluster that is not natively present in our enzyme [37-40], and connecting the hydrogenase to cyanobacterial electron carriers, such as ferredoxin.

## Conclusion

In summary, we have used an *E. coli* - cyanobacteria shuttle system to rapidly test candidate modifications of a heterologously-expressed *A. macleodii* [NiFe] hydrogenase in *E. coli* before transferring the most promising constructs to the slower-growing cyanobacteria. As a test of this strategy, we substituted the *A. macleodii* hydrogenase with modifications that in other systems have been shown to change the ligations of the medial and distal Fe-S clusters. When expressed in *E. coli*, we found that neither substitution alone improved enzyme activity; however, when combined, both substitutions increased hydrogen evolution activity approximately 4-fold relative to wild type, while hydrogen uptake remained unchanged. As we did not directly verify that these substitutions resulted in the corresponding alterations to the Fe-S clusters, we cannot completely rule out the possibility of more subtle effects on electron-transfer kinetics in the absence of the expected, new cysteinyl-iron bonds or the possibility that the native enzyme did not feature the expected Fe-S clusters. The effect of this combined modification was recapitulated with the enzymes in enriched form and with the enzymes expressed in cyanobacteria, suggesting that the enzymatic improvement is intrinsic to the enzyme and independent of extrinsic host factors. These results together validate our strategy of piloting hydrogenase modifications in the rapid-turnaround *E. coli* model system for use in cyanobacterial hosts.

## Methods

### Molecular biology

Primers, full plasmid maps and sequences are provided in supplementary materials. Plasmid pIY003 was constructed by PCR-amplification of the hydrogenase operon from *A. macleodii* genomic DNA. Two amplicons spanning the operon were produced where the sequences had overlapping ends with each other and to the plasmid pTRC-NS1 (the backbone plasmid from [16]), which was linearized by double digest with the restriction enzymes BamHI and EcoRI. These three DNA fragments were assembled using Gibson isothermal assembly [41], and transformed into DH10B (Invitrogen) cells by electroporation. After screening clones by PCR, candidate constructs were sequence-verified by Sanger DNA sequencing. Plasmids pIY004, bearing the H230C substitution and pIY006, bearing the P285C substitution were constructed similarly, except using PCR products generated from primers containing the appropriate DNA nucleotide substitutions; plasmid pIY007, bearing the doubly-substituted enzyme, was constructed by repeating the procedure used to generate pIY006 except using pIY004 plasmid as a template.

Plasmid pIY033 was constructed by moving the hydrogenase operon into a pDEST-23 vector backbone using Gibson isothermal assembly followed by re-amplification of this operon (now including T7 promoter and terminator) and recloning into the pTRC-NS1 plasmid by Gibson isothermal assembly. Successful construction was verified by Sanger sequencing. pIY038 was constructed by PCR amplification of a segment of pIY007 around the *hynS* ORF followed by Gibson isothermal assembly into plasmid pIY033 which was linearized by double digest with the BamHI and AgeI restriction enzymes. Construction of plasmid pRC41-4 will be discussed elsewhere but the sequence is provided in the Supplemental Information; pCM012 was constructed by PCR amplification of the second half of the *hynS* ORF from pIY038, followed by Gibson isothermal assembly into pRC41-4 which was linearized by double digest with BamHI and AgeI restriction enzymes. Successful construction of pIY038 and pCM012 was verified by Sanger sequencing initiated with PCR primers used to amplify the modified region. Plasmids pIY085 and pIY086 were constructed similarly to pCM012, with pIY003 and pIY007 as templates, respectively, except using two sequential rounds of amplification with two reverse primers together generating sequence encoding the strep-tag epitope –RSAWSHPQFEK and the sequence bridging the C-terminal stop codon of *hynS* and the beginning of *hynL* up to the AgeI restriction site. Successful construction of pIY085 and pIY086 was verified by Sanger sequencing initiated with the PCR primers used to amplify the modified region.

### *A. macleodii* Hydrogenase Expression in *E. coli*

All four plasmids pIY003, pIY004, pIY006, pIY007 were transformed into *E. coli* strain FTD147 cells, an MC4100(DE3) strain with the three active *E coli* hydrogenases removed [42]. Plasmids pIY033 and pIY038 were transformed into BL21(DE3)ΔH_4_ cells [18]. To grow *E. coli* cultures used to prepare extracts, 25 mL of autoinducer media [43] supplemented with 0.1 mM NiCl_2_ and 40 μg mL^-1^ spectinomycin was inoculated with 100 μL of a starter culture of the appropriate strain. Following inoculation, the expression culture was grown in an unbaffled 250 mL Erlenmeyer flask, rotating at 200 rpm, under ambient room atmosphere at 30°C overnight (~10-14 hrs). Under these conditions, the culture grew to a density in the approximate range of OD_600_ 6–7. Piloting experiments showed that growth in baffled flasks resulted in higher specific activity but was avoided to maintain comparability with previous experiments [15]. This was then harvested by centrifugation; the pellet was separated from the supernatant media and resuspended after addition of 0.8 mL lysis buffer (10 mM Tris, pH 7; 1 mM DTT; 0.5 mM EDTA). Lysis was performed by sonication on wet ice for one minute using a microprobe sonicator (Branson, Sonifier Model 250) at setting “4”, 40% duty cycle. The lysate was cleared by centrifugation for 10 minutes at 16,800 x g, at 4°C, and the supernatant was reserved as the “cleared lysate”. Protein content in the cleared lysate was measured with Bradford reagent (Bio-Rad) and comparison against a BSA (NEB) standard curve.

### *A. macleodii* Hydrogenase Expression in *S. elongatus*

Plasmids pRC41-4 and pCM012 were mobilized into *S. elongatus* PCC 7942 strain PW416 by conjugative transfer from *E. coli* as previously described [16,44]. Genomic integration into neutral site I (NSI) was verified by PCR. Cyanobacterial cultures were grown in 50 mL Bg11 medium in baffled 250 mL flasks to an OD_730_ of approximately 0.5. The cultures were induced by addition of IPTG to a final concentration of 0.25 mM, and additionally 0.5 μM NiCl_2_ was supplemented. Cells were grown for an additional 24 h, centrifuged, resuspended in 800 μL sonication buffer and sonicated as for *E. coli*. The lysates were cleared as described above for *E. coli* and the supernatant was reserved as the “cleared lysate”. *In vitro* hydrogenase evolution assays were conducted as described below.

### Evolution activity assay

Cleared lysate (0.2 mL) was added to 1.7 mL of methyl viologen assay buffer containing 1.5 mL deionized water, 0.1 mL 40 mg mL^-1^ methyl viologen (Aldrich), and 0.1 mL of 500 mM potassium phosphate, pH 7.0 (Gibco). The combined samples in 13 mL gastight vials was sealed using rubber septa (Aldrich), and sparged under argon (Westair) for 20 minutes to remove oxygen. After sparging, 0.1 mL of 2 M sodium dithionite (Aldrich) was added by syringe anoxically. The resulting assay solution was a 1:10 dilution of lysate containing 25 mM potassium phosphate, 8 mM methyl viologen, and 100 mM sodium dithionite, pH ~7.0. *E. coli* samples were incubated for 2 hours at 30°C, followed by gas chromatography (CP-3800, Varian) using a Fused Silica Molsieve 5A column (CP7537, Varian) of 100 μL samples taken from the vial headspace. Previous experiments [13,14] and piloting experiments for this experiment demonstrated time-dependent linearity of hydrogen evolution over the course of ~20 hours, suggesting that enzyme quality is unlikely to have been affected by the reaction conditions, including damage by oxidized dithionite products. *S. elongatus* samples were prepared similarly but were incubated at 30°C overnight before GC analysis. Hydrogen peaks were identified and integrated to quantify hydrogenase yield by comparison to a standard curve prepared with pure hydrogen from which a specific activity was calculated based on total protein in the lysate. For plasmids pIY003, pIY004, pIY006, and pIY007, the evolution activity was measured for four biological replicates with three technical replicates for each expression. For plasmids pIY033, pIY038, the evolution activity was measured for three technical replicates, and for pRC41-4, and pCM012, the evolution activity was measured for three biological replicates.

### Uptake activity assay

Cleared lysate (0.1 mL) was added to 0.9 mL of benzyl viologen assay buffer containing 0.05 mL of benzyl viologen (65 mg mL^-1^) and 0.05 mL of 0.5 M potassium phosphate buffer, pH 7.0, and 0.8 mL deionized water. The combined assay sample was prepared in a 2 mL round-rim disposable UV cuvette (Brandtech) and sealed with a rubber septum (Aldrich), coated with a small amount of vacuum grease (Fisher). The cuvette was sparged for 20 minutes under 10% hydrogen balanced by nitrogen (Westair). Cuvettes were then transferred to a multichannel spectrophotometer and monitored at 555 nm at 30°C. For all four variants, four instances of expression were conducted with four replicates for each instance, such that three of these replicate experiments appeared to have self-consistent progress curves without prejudice to the data processing. Reaction rates were identified by fitting a linear regression to the data between OD_555_ = 1 to OD_555_ = 3.

### Thermal exposure assay

The assay was conducted as the evolution assay, except cleared lysate was further incubated at 85°C for one hour and centrifuged a second time for 10 minutes at 16,800 x g and 4°C to clear additional insoluble debris prior to addition to MV buffer. Equivalent volumes of heat-treated and control-treated cleared lysate were added to assay buffer, and total hydrogen evolution activities without normalizing to protein concentration were used for subsequent calculations. Tolerance to thermal exposure was expressed by dividing the mean of three technical replicates of the denatured sample by the mean of three technical replicates of the undenatured sample. This process was subjected to three biological replicates; the geometric mean and geometric standard error are reported.

### Oxygen exposure assay

The H-D exchange assay to test sensitivity to oxygen exposure was performed as follows: cleared lysate (0.1 mL) was added to 0.8 mL of mass spectrometry buffer containing 0.05 mL of 0.5 M potassium phosphate buffer, pH 7.0 (Gibco), and 0.75 mL deionized water. The samples were placed in 10 mL scintillation vials, and capped with new rubber septa. Vials were sparged for 10 minutes under pressurized argon. A polyimide-coated fused silica capillary, ID 50 μm OD 220 μm (Scientific Instrument Services) adapted for mass spectrometry was then inserted into the vial headspace followed by mass spectrometric monitoring (Omnistar, Pfeiffer). The microcapillary substituted for the standard metal probe supplied with this instrument samples very low quantities of total gas making up an inconsequential amount of total gas volume for the duration of the experiment. Hydrogen (100%, Westair) was injected to a final concentration of 10% hydrogen headspace and verified by mass spectrometry. For microaerobic samples, air was injected to create a final 1% oxygen mixture. Anaerobic samples were incubated for 2h at 30°C to ensure quantitative enzyme recovery, followed by injection of 0.1 mL D_2_O, followed by monitoring of the developing m/z = 3, until χ_HD_ > 1000 ppm. Microaerobic samples, prepared in parallel, were then injected with 0.1 mL D_2_O followed by overnight mass spectrometric monitoring at m/z = 3. Oxygen tolerance was measured by filtering spikes from the progress curve, calculating enzyme rate and normalizing the slope of the microaerobic activity against the slope of the anaerobic activity. This process was subjected to three biological replicates; the geometric mean and geometric standard errors are reported.

### Protein purification

Cleared lysate from *E. coli* strains containing plasmid pIY085 and pIY086 was further purified using Streptactin magnetic bead resin (Qiagen). Cleared lysate was prepared as above but in NP buffer (50 mM sodium phosphate, pH 8.0, 300 mM NaCl) with 1 mM dithiothreitol and NeutrAvidin (Thermo) at 10 μg mL^-1^ final concentration. Streptactin resin (100 μl) was combined with cleared lysate (400 μl) and incubated at 4°C for 1 h with end-over-end rotation. The supernatant was removed and the resin was washed three times for five minutes each at room temperature in NP buffer containing 0.05% sodium dodecyl sulfate (SDS). This was followed by two washes in NP buffer supplemented with 0.01% Tween 20 as above. Final elution was performed at room temperature in 100 μl NP buffer supplemented with 0.01% Tween 20 and 10 mM biotin.

### Western blot

Lysates were diluted to 5 mg mL^-1^ total protein. One lysate from an overexpression of WT hydrogenase was further treated by denaturation at 65°C for 10 minutes and clearing by centrifugation, at 20,000 x g for 10 minutes. 25 μL of 4x NuPAGE SDS-PAGE loading buffer (Invitrogen) was added to 75 μL aliquots, followed by boiling for 10 minutes. These samples were frozen at −80°C and on a subsequent day thawed and 10 μL was separated onto a 10% NuPAGE Bis-Tris gel with the NuPAGE MOPS-SDS running buffer system (Invitrogen). Gel electrophoresis was performed at 150 V for 5 hours at 4°C, and the gel was cut separating the gel into two halves at approximately 75 kDa. The top gel was stained using the Sypro Ruby kit (Invitrogen) as a total protein loading control; the bottom gel was transferred onto nitrocellulose using NuPage transfer buffer (Invitrogen) with 30% MeOH for 10 hours at 10 mA constant current. These samples were then hybridized using rabbit-anti-HynL primary antibody [13] and goat-anti-rabbit-DyLight secondary antibody (Thermo), and imaged using a Typhoon fluorescence scanning imager (GE).

For gels associated with protein purification, samples were prepared as described above, but for cleared lysate, approximately 5 μg was loaded per lane and for purified proteins, approximately 0.5 μg protein was loaded per lane. Electrophoresis of the gels for western blot analysis of Strep-tagged HynS was only performed for 1 h at 150 V. Monoclonal antibody to the strep-tag antigen (Qiagen) was used with goat-anti-mouse-DyLight secondary antibody (Thermo) and imaged as above.

## Endnotes

^1^In this paper the amino acid sequence numbers used for *A. macleodii* HynS are relative to the translational start methionine as annotated (NCBI: YP_006975190.1), not the putative starting residue after N-terminal processing by the TAT system, as this site has not been experimentally verified.

## Abbreviations

WT: Wild type.

## Competing interests

The authors declare that they have no competing interests.

## Authors’ contributions

ITY conceived and designed the experiments, created plasmid constructs, collected all data pertaining to *E. coli*, and wrote the paper. CWM designed and created plasmid constructs, and helped draft the manuscript. TAN designed and created plasmids, and helped draft the manuscript. HOS designed the experiments, participated in its coordination, and helped draft the manuscript. PDW conceived and designed the experiments, collected all data pertaining to *S. elongatus*, performed enzyme purifications, and wrote the paper. All authors read and approved the final manuscript.

## Supplementary Material

Additional file 1: Table S1 Specific activities of enzyme preparations. **Table S2,** Primers used in this study. **Table S3,** (22pp) Maps and sequences for vectors used in this study. **Figure S1**, A representative enzyme activity progress curve. Click here for file
