# Supplementary material for: Dual organism design cycle reveals small subunit substitutions that improve [NiFe] hydrogenase hydrogen evolution
Source: J Biol Eng. 2013 Jul 2;7:17. doi: 10.1186/1754-1611-7-17 (PMC3701524; doi:10.1186/1754-1611-7-17)

Supporting Information

Table S1, Specific activities of enzyme preparations

E. coli	
**pIY003 (WT)**	
Evolution specific activity	0.91±0.10 nmH_2 min^{-1} (mg lysate)$^{-1}$
Uptake specific activity	4.8±0.6 nmH_2 min^{-1} (mg lysate)$^{-1}$
Relative activity after 85 °C, 1h	41% (std. err. 39-45%)*
Relative activity in 1% O_2	29% (std. err. 21-35%)*
**pIY004 (H230C)**	
Evolution specific activity	0.50±0.05 nmH_2 min^{-1} (mg lysate)$^{-1}$
Uptake specific activity	9.2±2.9 nmH_2 min^{-1} (mg lysate)$^{-1}$
**pIY006(P285C)**	
Evolution specific activity	0.35±0.04 nmH_2 min^{-1} (mg lysate)$^{-1}$
Uptake specific activity	7.7±1.2 nmH_2 min^{-1} (mg lysate)$^{-1}$
**pIY007(H230C/P285C)**	
Evolution specific activity	3.3±0.4 nmH_2 min^{-1} (mg lysate)$^{-1}$
Uptake specific activity	5.9±2.4 nmH_2 min^{-1} (mg lysate)$^{-1}$
Relative activity after 85 °C, 1h	27% (std. err. 17-35%)*
Relative activity in ~1% O_2	37% (std. err. 23-48%)*
Specific activity relative to pIY003	3.7±0.6
**pIY033(WT)**	
Evolution specific activity	1.6±0.3 nmH_2 min^{-1} (mg lysate)$^{-1}$
**pIY038(H230C/P285C)**	
Evolution specific activity	3.8±0.5 nmH_2 min^{-1} (mg lysate)$^{-1}$
Specific activity relative to pIY033	2.3±0.8
**pRC41-4(WT)**	
Evolution specific activity	9.1 nmH_2 min^{-1} (mg lysate)$^{-1}$
**pCM012(H230C/P285C)**	
Evolution specific activity	19 nmH_2 min^{-1} (mg lysate)$^{-1}$
**pIY085(WT, strep-tagged)**	
Evolution specific activity, unpurified	6.1 nmH_2 min^{-1} (mg lysate)$^{-1}$
Evolution specific activity, purified	690 nmH_2 min^{-1} (mg protein)$^{-1}$
**pIY086(H230C/P285C, strep-tagged)**	
Evolution specific activity, unpurified	12.7 nmH_2 min^{-1} (mg lysate)$^{-1}$
Evolution specific activity, purified	2200 nmH_2 min^{-1} (mg protein)$^{-1}$
Purified specific activity relative to pIY085	3.2
S. elongatus	
**pRC41-4(WT)**	
Evolution specific activity	0.10±0.01 nmH_2 min^{-1} (mg lysate)$^{-1}$
**pCM012(H230C/P285C)**	
Evolution specific activity	0.54±.06 nmH_2 min^{-1} (mg lysate)$^{-1}$
Specific activity relative to pRC41-4	5.1±0.7

*geometric standard error

Table S2, Primers used in this study

Name	Sequence	Purpose
AMR3-2	**CAATTTCACACAGGAAACAGACC**ATGGATG GCTTCCATTATTGGTAGC	Forward primer for amplification of hydrogenase operon starting at orf2 (light); overlaps with pTRC-NS1 (bold)
AMR-44	**TGCATGCCTGCAGGTCGACTCTAGAGGATC** TCAGCAGATCCTTGGAAGC	Reverse primer for amplification of hydrogenase operon ending at hypE (light); overlaps with pTRC-NS1(bold)
IY002HopMidF	CCAGGCTACTCGAAGAAACG	Forward primer in the middle of hydrogenase operon
IY003HopMidR	GCGCACGTATCGTTTCTTCG	Reverse primer in the middle of the hydrogenase operon
IY004HydSH238CF	TGACAGATGTTATCGACGCCC	Forward primer to generate H230C
IY005HydSH238CR	GGGCGTCGATAACATCTGTC**ACA**GATGCTT TCACCAAAAAAAG	Reverse primer to generate H230C mutation (bold)
IY010pDEST24F	GGCTGCTGCCACCGCTGAGC	Forward primer to amplify pDEST backbone
IY011pDEST24R	CTATAGTGAGTCGTATTAATTTCGCGGG	Reverse primer to amplify pDEST backbone
IY012HydT7CBAF	**CCCGCGAAATTAATACGACTCACTATAG**GG AAACAGACCATGGATGGC	Forward primer to amplify hydrogenase operon for insertion into pDEST24; overlaps with IY011pDEST24R (bold)
IY013HydT7CBAR	**GCTCAGCGGTGGCAGCAGCC**AGGATCTCAG CAGATCCTTGG	Reverse primer to amplify hydrogenase operon for insertion into pDEST24; overlaps with IY010pDEST24F (bold)
IY014pDEST24F	CCCGCGAAATTAATACGACTCACTATAG	Forward primer to amplify hydrogenase operon out of pDEST24 plasmid
IY015pDEST24R	CTCGGAGCACTGTCCGACCGC	Reverse primer to amplify hydrogenase operon out of pDEST24 plasmid
IY016NS1pDEST24F	**GCGGTCGGACAGTGCTCCGAG**CGAGCTTCT GGAGCAGGAAG	Forward primer to amplify pTRC-NS1 for insertion of T7-driven hydrogenase; overlaps with IY015pDEST24R(bold)
IY017NS1pDEST24R	**CTATAGTGAGTCGTATTAATTTCGCGGG**CC GATCCTCTAGTATGCTTGTAAACC	Reverse primer to amplify pTRC-NS1 for insertion of T7-driven hydrogenase; overlaps with IY014pDEST24F(bold)
IY018HydSP293CF	ATCGAATCTGGCCATCCGTGT	Forward primer to generate P285C
IY019HydSP293CR	ACACGGATGGCCAGATTCGAT**ACA**AAAACT CGTGCCTTG	Reverse primer to generate P285C mutation (bold)
IY170HynSF	GTATTATTGCCATCGGTACC	Forward primer upstream of BamHI site to transfer mutants
IY171HynSR	CGAGTGATAGGATCGACGAC	Reverse primer downstream of AgeI site to transfer mutants
AN000StrepF	CGTAGCGCTTGGTCCCACCCGCAGTTCGAA AAA**TAAGATAATAGGCAAGGCTCATGG**	Forward primer encoding the HynS C-terminal strep-tag; overlaps with hynS-hynL intergenic region (bold)
AN001StrepR	TTTTTCGAACTGCGGGTGGGACCAAGCGCT ACGTGAG**TTTTTCCCAGCATGCTTC**	Reverse primer encoding the HynS C-terminal strep-tag; overlaps with the c-terminus of hynS (bold)

Table S3 (22pp), Maps and sequences for vectors used in this study

Map Key:

Green – replication origins, transfer origins, transcriptional promoters

oriV – vegetative origin from p15

oriT – RP4 conjugation transfer origin

PTRC – P_{TRC} promoter (includes lacO repressor element)

PT7 – P_{T7} promoter

lacO – lacO repressor element

Red – transcriptional terminators, mutations

T4t – T4 terminator

rrnBt – rrnB terminator

Grey – upstream and downstreamNS1 homology regions for transfer into *S. elongatus*

Yellow – ORFs

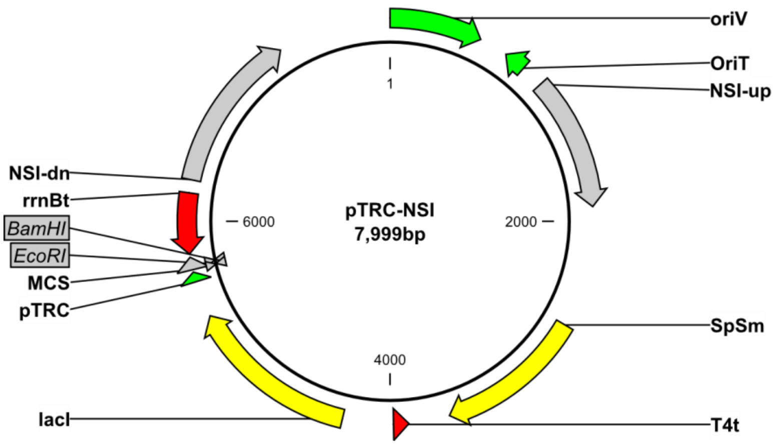

TTGAGATCCTTTTTTTCTGCGCGTAATCTGCTGCTTGCAAACAAAAAAACCACCGCTACCAGCGGTGGTTTGTTTGCCGGATCAAGAGCTACCAACTCTTTTTCCGAAGGTAACTGGCTTCAGCAGAGCGCAGATACCAAATACTGTCCTTCTAGTGTAGCCGTAGTTAG
GCCACCACTTCAAGAACTCTGTAGCACCGCCTACATACCTCGCTCTGCTAATCCTGTTACCAGTGGCTGCTGCCAGTGGCGATAAGTCGTGTCTTACCGGGTTGGACTCAAGACGATAGTTACCGGATAAGGCGCAGCGGTCGGGCTGAACGGGGGGTTCGTGCACACAG
CCCAGCTTGGAGCGAACGACCTACACCGAACTGAGATACCTACAGCGTGAGCTATGAGAAAGCGCCACGCTTCCCGAAGGGAGAAAGGCGGACAGGTATCCGGTAAGCGGCAGGGTCGGAACAGGAGAGCGCACGAGGGAGCTTCCAGGGGGAAACGCCTGGTATCTTTA
TAGTCCTGTCGGGTTTCGCCACCTCTGACTTGAGCGTCGATTTTTGTGATGCTCGTCAGGGGGGCGGAGCCTATGGAAAAACGCCAGCAACGCGGCCTTTTTACGGTTCCTGGCCTTTTGCTGGCCTTTTGCTCACATGTTCTTTCCTGCGTTATCCCCTGATTCTGTGG
ATAACCGTATTACCGCCTTTGAGTGAGCTGATACCGCTCGCCGCAGCCGAACGACCGAGCGCAGCGAGTCAGTGAGCGAGGAAGCGGAAGAGCGCCTGATGCGGTATTTTCTCCTTACGCATCTGTGCGGTATTTCACACCGCATATGGTGCACTCTCAGTACAATCTGC
TCTGATGCCGCATAGTTAAGCCAGTATACACTCCGCTATCGCTACGTGACTGGGTCATGGCTGCGCCCCGACACCCGCCAACACCCGCTGACGCGCCCTGACGGGCTTGTCTGCTCCCGGCATCCGCTTACAGACAAGCTGTGACCGTCTCCGGGAGCTGCATGTGTCAG
AGGTTTTCACCGTCATCACCGAAACGCGCGAGGCAGGATATCCGGCAGCCGGCGGAGCGCTGCTTTCTTGGCAAGCGGTCGCCAGCCCCAACGCCAGGGCTGCCAGCCCGAAACAGCGGGGCAAGGCAGCTTGGAAGGGCGATCGCAGCACGGGCATGGCAATGTCTCTC
TGAAGGAATGCAGACCTTATTCGTACAGCCAGGGTTGAATCGTGGGGGTCCAATCACTTAGCTCTGCTGGGCTAAACCAGAGAGCAATTTCCTGTTGTGCTGTTTCGATTGCATCCGAGCCATGGATGATGTTGCGGCCAATATTGACACCAAAATCACCACGGATGGTG
CCCGGTTCTGCCGTCAGCGGATTGGTAGCGCCGATCAACTTGCGAGCAGCCGCCACAACGCCTTCGCCTTCCAAGACGATCGCCACGATCGGCCCAGAGGTGATGAACTCGACGAGGCCATTGAAGAAGGGGCGCTCGCGGTGGACAGCATAGTGCTGTTCGGCCAGCTC
GCGACTGGGCTTCAGCTGCTTTAGGCCCACCAGTTTGAAGCCTTTTTGCTCAAAGCGGCCGATGATCGTACCGACCAAACCCCGCTGAACGCCATCGGGCTTGATGGCAATAAATGTGCGTTCCACAGACATCTAGATAGTCCTCAAGACGAGGCAAGCATTGAGCTTGC
CTTCCTATGGTTCGGGATCACTGGGATTCTTGACAAGCGATCGCGGTCACATCGCTATCTCTTAGGACTTCGCAGCGGGCGAGTCGGATTGACCCGGTAGGGATTTCGCCAGATCAATGCCCGTGGTTTGTTTCAGCTTCTCCAGCAAGCTAGCGATTTGGGTAGCGCTG
CCTTCCCCTTCGCCAATCACAGTGATCGACTCCACGTCGATATCTGGCACGGTGCCTGAAAGCGTGACGAGCAGGGACTCGAAGCTTGCATGCCTGCAGGTCGACTCTAGAGCTTTATGCTTGTAAACCGTTTTGTGAAAAAATTTTTAAAATAAAAAAGGGGACCTCTA
GGGTCCCCAATTAATTAGTAATATAATCTATTAAAGGTCATTCAAAAGGTCATCCACCGGATCAATTCCCCTGCTCGCGCAGGCTGGGTGCCAAGCTCTCGGGTAACATCAAGGCCCGATCCTTGGAGCCCTTGCCCTCCCGCACGATGATCGTGCCGTGATCGAAATCC
AGATCCTTGACCCGCAGTTGCAAACCCTCACTGATCCGCATGCCCGTTCCATACAGAAGCTGGGCGAACAAACGATGCTCGCCTTCCAGAAAACCGAGGATGCGAACCACTTCATCCGGGGTCAGCACCACCGGCAAGCGCCGCGACGGCCGAGGTCTTCCGATCTCCTG
AAGCCAGGGCAGATCCGTGCACAGCACCTTGCCGTAGAAGAACAGCAAGGCCGCCAATGCCTGACGATGCGTGGAGACCGAAACCTTGCGCTCGTTCGCCAGCCAGGACAGAAATGCCTCGACTTCGCTGCTGCCCAAGGTTGCCGGGTGACGCACACCGTGGAAACGGA
TGAAGGCACGAACCCAGTGGACATAAGCCTGTTCGGTTCGTAAGCTGTAATGCAAGTAGCGTATGCGCTCACGCAACTGGTCCAGAACCTTGACCGAACGCAGCGGTGGTAACGGCGCAGTGGCGGTTTTCATGGCTTGTTATGACTGTTTTTTTGGGGTACAGTCTATG
CCTCGGGCATCCAAGCAGCAAGCGCGTTACGCCGTGGGTCGATGTTTGATGTTATGGAGCAGCAACGATGTTACGCAGCAGGGCAGTCGCCCTAAAACAAAGTTAAACATCATGAGGGAAGCGGTGATCGCCGAAGTATCGACTCAACTATCAGAGGTAGTTGGCGTCAT
CGAGCGCCATCTCGAACCGACGTTGCTGGCCGTACATTTGTACGGCTCCGCAGTGGATGGCGGCCTGAAGCCACACAGTGATATTGATTTGCTGGTTACGGTGACCGTAAGGCTTGATGAAACAACGCGGCGAGCTTTGATCAACGACTTTTGGAAACTTCGGCTTCCCC
TGGAGAGAGCGAGATTCTCCGCGCTGTAGAAGTCACCATTGTTGTGCACGACGACATCATTCCGTGGCGTTATCCAGCTAAGCGCGAACTGCAATTTGGAGAATGGCAGCGCAATGACATTCTTGCAGGTATCTTCGAGCCAGCCACGATCGACATTGATCTGGCTATCT
TGCTGACAAAAGCAAGAGAACATAGCGTTGCCTTGGTAGGTCCAGCGGCGGAGGAACTCTTTGATCCGGTTCCTGAACAGGATCTATTTGAGGCGCTAAATGAAACCTTAACGCTATGGAACTCGCCGCCCGACTGGGCTGGCGATGAGCGAAATGTAGTGCTTACGTTG
TCCCGCATTTGGTACAGCGCAGTAACCGGCAAAATCGCGCCGAAGGATGTCGCTGCCGACTGGGCAATGGAGCGCCTGCCGGCCCAGTATCAGCCCGTCATACTTGAAGCTAGACAGGCTTATCTTGGACAAGAAGAAGATCGCTTGGCCTCGCGCGCAGATCAGTTGGA
AGAATTTGTCCACTACGTGAAAGGCGAGATCACCAAGGTAGTCGGCAAATAATGTCTAACAATTCGTTCAAGCCGACGCCGCTTCGCGGCGCGGCTTAACTCAAGCGTTAGATGCACTAAGCACATAATTGCTCACAGCCAAACTATCAGGTCAAGTCTGCTTTTATTAT
TTTTAAGCGTGCATAATAAGCCCTACACAAATTGGGAGATATATCATGAAAGGCTGGCTTTTTCTTGTTATCGCAATAGTTGGCGAAGTAATCGCAACATCCGCATTAAAATCTAGCGAGGGCTTTACTAAGCTGATCCGGTGGATGACCTTTTGAATGACCTTTAATAG
ATTATATTACTAATTAATTGGGGACCCTAGAGGTCCCCTTTTTTATTTTAAAAATTTTTTCACAAAACGGTTTACAAGCATACTAGAGGATCGGCGGCCGCGGATCTGGGCCGCGGGTCATGGCTGCGCCCCGACACCCGCCAACACCCGCTGACGCGCCCTGACGGGCT
TGTCTGCTCCCGGCATCCGCTTACAGACAAGCTGTGACCGTCTCCGGGAGCTGCATGTGTCAGAGGTTTTCACCGTCATCACCGAAACGCGCGAGGCAGCAGATCAATTCGCGCGCGAAGGCGAAGCGGCATGCATTTACGTTGACACCATCGAATGGTGCAAAACCTTT
CGCGGTATGGCATGATAGCGCCCGGAAGAGAGTCAATTCAGGGTGGTGAATGTGAAACCAGTAACGTTATACGATGTCGCAGAGTATGCCGGTGTCTCTTATCAGACCGTTTCCCGCGTGGTGAACCAGGCCAGCCACGTTTCTGCGAAAACGCGGGAAAAAGTGGAAGC
GGCGATGGCGGAGCTGAATTACATTCCCAACCGCGTGGCACAACAACTGGCGGGCAAACAGTCGTTGCTGATTGGCGTTGCCACCTCCAGTCTGGCCCTGCACGCGCCGTCGCAAATTGTCGCGGCGATTAAATCTCGCGCCGATCAACTGGGTGCCAGCGTGGTGGTGT
CGATGGTAGAACGAAGCGGCGTCGAAGCCTGTAAAGCGGCGGTGCACAATCTTCTCGCGCAACGCGTCAGTGGGCTGATCATTAACTATCCGCTGGATGACCAGGATGCCATTGCTGTGGAAGCTGCCTGCACTAATGTTCCGGCGTTATTTCTTGATGTCTCTGACCAG
ACACCCATCAACAGTATTATTTTCTCCCATGAAGACGGTACGCGACTGGGCGTGGAGCATCTGGTCGCATTGGGTCACCAGCAAATCGCGCTGTTAGCGGGCCCATTAAGTTCTGTCTCGGCGCGTCTGCGTCTGGCTGGCTGGCATAAATATCTCACTCGCAATCAAAT
TCAGCCGATAGCGGAACGGGAAGGCGACTGGAGTGCCATGTCCGGTTTTCAACAAACCATGCAAATGCTGAATGAGGGCATCGTTCCCACTGCGATGCTGGTTGCCAACGATCAGATGGCGCTGGGCGCAATGCGCGCCATTACCGAGTCCGGGCTGCGCGTTGGTGCGG
ATATCTCGGTAGTGGGATACGACGATACCGAAGACAGCTCATGTTATATCCCGCCGTTAACCACCATCAAACAGGATTTTCGCCTGCTGGGGCAAACCAGCGTGGACCGCTTGCTGCAACTCTCTCAGGGCCAGGCGGTGAAGGGCAATCAGCTGTTGCCCGTCTCACTG
GTGAAAAGAAAAACCACCCTGGCGCCCAATACGCAAACCGCCTCTCCCCGCGCGTTGGCCGATTCATTAATGCAGCTGGCACGACAGGTTTCCCGACTGGAAAGCGGGCAGTGAGCGCAACGCAATTAATGTAAGTTAGCGCGAATTGATCTGGTTTGACAGCTTATCAT
CGACTGCACGGTGCACCAATGCTTCTGGCGTCAGGCAGCCATCGGAAGCTGTGGTATGGCTGTGCAGGTCGTAAATCACTGCATAATTCGTGTCGCTCAAGGCGCACTCCCGTTCTGGATAATGTTTTTTGCGCCGACATCATAACGGTTCTGGCAAATATTCTGAAATG
AGCTGTTGACAATTAATCATCCGGCTCGTATAATGTGTGGAATTGTGAGCGGATAACAATTTCACACAGGAAACAGACCATGGAATTCGAGCTCAAGGAGGAATAACATATGGGATCCTCTAGAGTCGACCTGCAGGCATGCAAGCTTGGCTGTTTTGGCGGATGAGAGA
AGATTTTCAGCCTGATACAGATTAAATCAGAACGCAGAAGCGGTCTGATAAAACAGAATTTGCCTGGCGGCAGTAGCGCGGTGGTCCCACCTGACCCCATGCCGAACTCAGAAGTGAAACGCCGTAGCGCCGATGGTAGTGTGGGGTCTCCCCATGCGAGAGTAGGGAAC
TGCCAGGCATCAAATAAAACGAAAGGCTCAGTCGAAAGACTGGGCCTTTCGTTTTATCTGTTGTTTGTCGGTGAACGCTCTCCTGAGTAGGACAAATCCGCCGGGAGCGGATTTGAACGTTGCGAAGCAACGGCCCGGAGGGTGGCGGGCAGGACGCCCGCCATAAACTG
CCAGGCATCAAATTAAGCAGAAGGCCATCCTGACGGATGGCCTTTTTGCGTTTCTACAAACTCTTTTGTTTATTTTTCTAAATACATTCAAATATGTATCCGCTCATGGCGGCCCAGATCCCCGGGTACCGAGCTCGAATTTCGAGCTTCTGGAGCAGGAAGATGTCGCG
GGCATTAGCACCAGCGGTCTGCCAAGCCTCCGCCAGCCGTTGGGTCCCTTCCGCTTGAGCTTTTCCATCTTCGACGATACGGGCGGCGGCCCCCCGCGCTTCCGCGATCGCCCGTTTACAAGCTGCCTCAGCTGGGGCGATCACATCGGCTTGAAGTTGCTGCTGCACCT
GTTTGATCCGCTCCTGCTGCACAGGGAGTTCTGCTTGGCTACGAGCGACTTCGGTAGCAATGTCCGCTTCAGCTTCGGCCACCACCGCTTCGCGCCGCGTCAACGCATCCTGAATCCGGCGCTCGGCCTCGGCTTGGGCGATCGCTACATCGCGATCGATCCGACGCAGG
GCCGTGATCTTGTCATTTTCGGCCGTTTGGATCGCAGAGGCAGCCTGGGCATCGGCTTCAGCAATTCGGGCATCTCGCTGCAGATCAGCCCGCTGCTTGCGTCCACTAGCCGAGAGATAACCGACCTCATCGGAAATGTTCTGGACTTGCAGCGTATCGAGGACTAGACC
CAGCTGCTCAAGGTCATCCTCCGCCTCTTCCAGCAGACTTTTGGCAAAGGCAATTTTGTCCTCGTTGATCTGCTCCGGCGTGAGGCTGGCTAAAACACCACGCAAGTTGCCTTCGAGGGTCTCCTTGGCAATTTGCTCGATTTCCTTACGGTTTTTGCCAAGCAGCCGCT
CGATCGCGTTGTGGATGGTCGGTTCTTCCCCAGCAATCTTGATATTGGCAACGCCTTCAACAGTCAGGGGAATGCCGCCCTTGGAGAAGGCATTGGAAACGCGCAACTCAATGATCATGTTGGTCAGATCCATGCGGAGCGCTTTTTCCAGCAGAGGTACCCGCAGGCTG
CTGCCGCCCTTGACCAAGCGATAGCCAACTCGGCGGCCATCACTACTGCGGCGACTACTGCCAGCAAAGATCAAAATTTCACTGGGTTGGCAGATGTAGTAGAGATTGCGCAGGACTAAGCTGCCAGCCCCGGCGACTCACCAGTCACAGAAAAGCATCTTACGGATGGC
ATGACAGTAAGAGAATTATGCAGTGCTGCCATAACCATGAGTGATAACACTGCGGCCAACTTACTTCTGACAACGATCGGAGGACCGAAGGAGCTAACCGCTTTTTTGCACAACATGGGGGATCATGTAACTCGCCTTGATCGTTGGGAACCGGAGCTGAATGAAGCCAT
ACCAAACGACGAGCGTGACACCACGATGCCTGCAGCAATGGCAACAACGTTGCGCAAACTATTAACTGGCGAACTACTTACTCTAGCTTCCCGGCAACAATTAATAGACTGGATGGAGGCGGATAAAGTTGCAGGACCACTTCTGCGCTCGGCCCTTCCGGCTGGCTGGT
TTATTGCTGATAAATCTGGAGCCGGTGAGCGTGGGTCTCGCGGTATCATTGCAGCACTGGGGCCAGATGGTAAGCCCTCCCGTATCGTAGTTATCTACACGACGGGGAGTCAGGCAACTATGGATGAACGAAATAGACAGATCGCTGAGATAGGTGCCTCACTGATTAAG
CATTGGTAACTGTCAGACCAAGTTTACTCATATATACTTTAGATTGATTTAAAACTTCATTTTTAATTTAAAAGGATCTAGGTGAAGATCCTTTTTGATAATCTCATGACCAAAATCCCTTAACGTGAGTTTTCGTTCCACTGAGCGTCAGACCCCGTAGAAAAGATCAA
AGGATCTTC

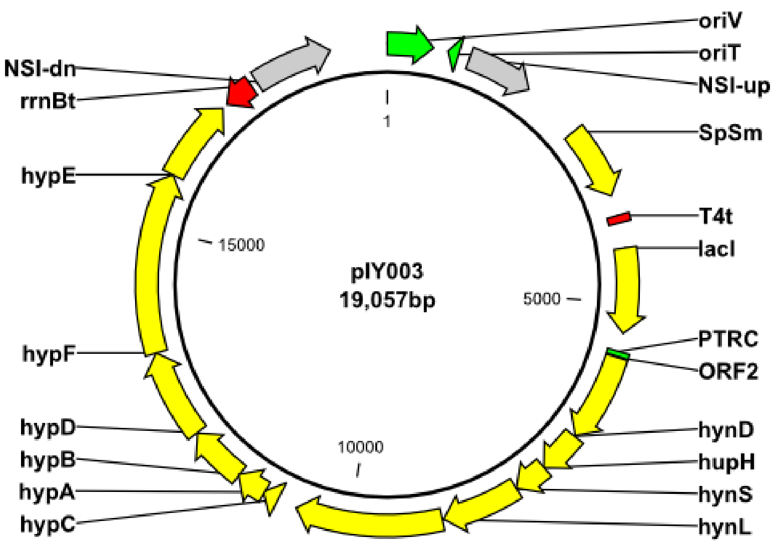

TTGAGATCCTTTTTTTCTGCGCGTAATCTGCTGCTTGCAAACAAAAAAACCACCGCTACCAGCGGTGGTTTGTTTGCCGGATCAAGAGCTACCAACTCTTTTTCCGAAGGTAACTGGCTTCAGCAGAGCGCAGATACCAAATACTGTCCTTCTAGTGTAGCCGTAGTTAG
GCCACCACTTCAAGAACTCTGTAGCACCGCCTACATACCTCGCTCTGCTAATCCTGTTACCAGTGGCTGCTGCCAGTGGCGATAAGTCGTGTCTTACCGGGTTGGACTCAAGACGATAGTTACCGGATAAGGCGCAGCGGTCGGGCTGAACGGGGGGTTCGTGCACACAG
CCCAGCTTGGAGCGAACGACCTACACCGAACTGAGATACCTACAGCGTGAGCTATGAGAAAGCGCCACGCTTCCCGAAGGGAGAAAGGCGGACAGGTATCCGGTAAGCGGCAGGGTCGGAACAGGAGAGCGCACGAGGGAGCTTCCAGGGGGAAACGCCTGGTATCTTTA
TAGTCCTGTCGGGTTTCGCCACCTCTGACTTGAGCGTCGATTTTTGTGATGCTCGTCAGGGGGGCGGAGCCTATGGAAAAACGCCAGCAACGCGGCCTTTTTACGGTTCCTGGCCTTTTGCTGGCCTTTTGCTCACATGTTCTTTCCTGCGTTATCCCCTGATTCTGTGG
ATAACCGTATTACCGCCTTTGAGTGAGCTGATACCGCTCGCCGCAGCCGAACGACCGAGCGCAGCGAGTCAGTGAGCGAGGAAGCGGAAGAGCGCCTGATGCGGTATTTTCTCCTTACGCATCTGTGCGGTATTTCACACCGCATATGGTGCACTCTCAGTACAATCTGC
TCTGATGCCGCATAGTTAAGCCAGTATACACTCCGCTATCGCTACGTGACTGGGTCATGGCTGCGCCCCGACACCCGCCAACACCCGCTGACGCGCCCTGACGGGCTTGTCTGCTCCCGGCATCCGCTTACAGACAAGCTGTGACCGTCTCCGGGAGCTGCATGTGTCAG
AGGTTTTCACCGTCATCACCGAAACGCGCGAGGCAGGATATCCGGCAGCCGGCGGAGCGCTGCTTTCTTGGCAAGCGGTCGCCAGCCCCAACGCCAGGGCTGCCAGCCCGAAACAGCGGGGCAAGGCAGCTTGGAAGGGCGATCGCAGCACGGGCATGGCAATGTCTCTC
TGAAGGAATGCAGACCTTATTCGTACAGCCAGGGTTGAATCGTGGGGGTCCAATCACTTAGCTCTGCTGGGCTAAACCAGAGAGCAATTTCCTGTTGTGCTGTTTCGATTGCATCCGAGCCATGGATGATGTTGCGGCCAATATTGACACCAAAATCACCACGGATGGTG
CCCGGTTCTGCCGTCAGCGGATTGGTAGCGCCGATCAACTTGCGAGCAGCCGCCACAACGCCTTCGCCTTCCAAGACGATCGCCACGATCGGCCCAGAGGTGATGAACTCGACGAGGCCATTGAAGAAGGGGCGCTCGCGGTGGACAGCATAGTGCTGTTCGGCCAGCTC
GCGACTGGGCTTCAGCTGCTTTAGGCCCACCAGTTTGAAGCCTTTTTGCTCAAAGCGGCCGATGATCGTACCGACCAAACCCCGCTGAACGCCATCGGGCTTGATGGCAATAAATGTGCGTTCCACAGACATCTAGATAGTCCTCAAGACGAGGCAAGCATTGAGCTTGC
CTTCCTATGGTTCGGGATCACTGGGATTCTTGACAAGCGATCGCGGTCACATCGCTATCTCTTAGGACTTCGCAGCGGGCGAGTCGGATTGACCCGGTAGGGATTTCGCCAGATCAATGCCCGTGGTTTGTTTCAGCTTCTCCAGCAAGCTAGCGATTTGGGTAGCGCTG
CCTTCCCCTTCGCCAATCACAGTGATCGACTCCACGTCGATATCTGGCACGGTGCCTGAAAGCGTGACGAGCAGGGACTCGAAGCTTGCATGCCTGCAGGTCGACTCTAGAGCTTTATGCTTGTAAACCGTTTTGTGAAAAAATTTTTAAAATAAAAAAGGGGACCTCTA
GGGTCCCCAATTAATTAGTAATATAATCTATTAAAGGTCATTCAAAAGGTCATCCACCGGATCAATTCCCCTGCTCGCGCAGGCTGGGTGCCAAGCTCTCGGGTAACATCAAGGCCCGATCCTTGGAGCCCTTGCCCTCCCGCACGATGATCGTGCCGTGATCGAAATCC
AGATCCTTGACCCGCAGTTGCAAACCCTCACTGATCCGCATGCCCGTTCCATACAGAAGCTGGGCGAACAAACGATGCTCGCCTTCCAGAAAACCGAGGATGCGAACCACTTCATCCGGGGTCAGCACCACCGGCAAGCGCCGCGACGGCCGAGGTCTTCCGATCTCCTG
AAGCCAGGGCAGATCCGTGCACAGCACCTTGCCGTAGAAGAACAGCAAGGCCGCCAATGCCTGACGATGCGTGGAGACCGAAACCTTGCGCTCGTTCGCCAGCCAGGACAGAAATGCCTCGACTTCGCTGCTGCCCAAGGTTGCCGGGTGACGCACACCGTGGAAACGGA
TGAAGGCACGAACCCAGTGGACATAAGCCTGTTCGGTTCGTAAGCTGTAATGCAAGTAGCGTATGCGCTCACGCAACTGGTCCAGAACCTTGACCGAACGCAGCGGTGGTAACGGCGCAGTGGCGGTTTTCATGGCTTGTTATGACTGTTTTTTTGGGGTACAGTCTATG
CCTCGGGCATCCAAGCAGCAAGCGCGTTACGCCGTGGGTCGATGTTTGATGTTATGGAGCAGCAACGATGTTACGCAGCAGGGCAGTCGCCCTAAAACAAAGTTAAACATCATGAGGGAAGCGGTGATCGCCGAAGTATCGACTCAACTATCAGAGGTAGTTGGCGTCAT
CGAGCGCCATCTCGAACCGACGTTGCTGGCCGTACATTTGTACGGCTCCGCAGTGGATGGCGGCCTGAAGCCACACAGTGATATTGATTTGCTGGTTACGGTGACCGTAAGGCTTGATGAAACAACGCGGCGAGCTTTGATCAACGACTTTTGGAAACTTCGGCTTCCCC
TGGAGAGAGCGAGATTCTCCGCGCTGTAGAAGTCACCATTGTTGTGCACGACGACATCATTCCGTGGCGTTATCCAGCTAAGCGCGAACTGCAATTTGGAGAATGGCAGCGCAATGACATTCTTGCAGGTATCTTCGAGCCAGCCACGATCGACATTGATCTGGCTATCT
TGCTGACAAAAGCAAGAGAACATAGCGTTGCCTTGGTAGGTCCAGCGGCGGAGGAACTCTTTGATCCGGTTCCTGAACAGGATCTATTTGAGGCGCTAAATGAAACCTTAACGCTATGGAACTCGCCGCCCGACTGGGCTGGCGATGAGCGAAATGTAGTGCTTACGTTG
TCCCGCATTTGGTACAGCGCAGTAACCGGCAAAATCGCGCCGAAGGATGTCGCTGCCGACTGGGCAATGGAGCGCCTGCCGGCCCAGTATCAGCCCGTCATACTTGAAGCTAGACAGGCTTATCTTGGACAAGAAGAAGATCGCTTGGCCTCGCGCGCAGATCAGTTGGA
AGAATTTGTCCACTACGTGAAAGGCGAGATCACCAAGGTAGTCGGCAAATAATGTCTAACAATTCGTTCAAGCCGACGCCGCTTCGCGGCGCGGCTTAACTCAAGCGTTAGATGCACTAAGCACATAATTGCTCACAGCCAAACTATCAGGTCAAGTCTGCTTTTATTAT
TTTTAAGCGTGCATAATAAGCCCTACACAAATTGGGAGATATATCATGAAAGGCTGGCTTTTTCTTGTTATCGCAATAGTTGGCGAAGTAATCGCAACATCCGCATTAAAATCTAGCGAGGGCTTTACTAAGCTGATCCGGTGGATGACCTTTTGAATGACCTTTAATAG
ATTATATTACTAATTAATTGGGGACCCTAGAGGTCCCCTTTTTTATTTTAAAAATTTTTTCACAAAACGGTTTACAAGCATACTAGAGGATCGGCGGCCGCGGATCTGGGCCGCGGGTCATGGCTGCGCCCCGACACCCGCCAACACCCGCTGACGCGCCCTGACGGGCT
TGTCTGCTCCCGGCATCCGCTTACAGACAAGCTGTGACCGTCTCCGGGAGCTGCATGTGTCAGAGGTTTTCACCGTCATCACCGAAACGCGCGAGGCAGCAGATCAATTCGCGCGCGAAGGCGAAGCGGCATGCATTTACGTTGACACCATCGAATGGTGCAAAACCTTT
CGCGGTATGGCATGATAGCGCCCGGAAGAGAGTCAATTCAGGGTGGTGAATGTGAAACCAGTAACGTTATACGATGTCGCAGAGTATGCCGGTGTCTCTTATCAGACCGTTTCCCGCGTGGTGAACCAGGCCAGCCACGTTTCTGCGAAAACGCGGGAAAAAGTGGAAGC
GGCGATGGCGGAGCTGAATTACATTCCCAACCGCGTGGCACAACAACTGGCGGGCAAACAGTCGTTGCTGATTGGCGTTGCCACCTCCAGTCTGGCCCTGCACGCGCCGTCGCAAATTGTCGCGGCGATTAAATCTCGCGCCGATCAACTGGGTGCCAGCGTGGTGGTGT
CGATGGTAGAACGAAGCGGCGTCGAAGCCTGTAAAGCGGCGGTGCACAATCTTCTCGCGCAACGCGTCAGTGGGCTGATCATTAACTATCCGCTGGATGACCAGGATGCCATTGCTGTGGAAGCTGCCTGCACTAATGTTCCGGCGTTATTTCTTGATGTCTCTGACCAG
ACACCCATCAACAGTATTATTTTCTCCCATGAAGACGGTACGCGACTGGGCGTGGAGCATCTGGTCGCATTGGGTCACCAGCAAATCGCGCTGTTAGCGGGCCCATTAAGTTCTGTCTCGGCGCGTCTGCGTCTGGCTGGCTGGCATAAATATCTCACTCGCAATCAAAT
TCAGCCGATAGCGGAACGGGAAGGCGACTGGAGTGCCATGTCCGGTTTTCAACAAACCATGCAAATGCTGAATGAGGGCATCGTTCCCACTGCGATGCTGGTTGCCAACGATCAGATGGCGCTGGGCGCAATGCGCGCCATTACCGAGTCCGGGCTGCGCGTTGGTGCGG
ATATCTCGGTAGTGGGATACGACGATACCGAAGACAGCTCATGTTATATCCCGCCGTTAACCACCATCAAACAGGATTTTCGCCTGCTGGGGCAAACCAGCGTGGACCGCTTGCTGCAACTCTCTCAGGGCCAGGCGGTGAAGGGCAATCAGCTGTTGCCCGTCTCACTG
GTGAAAAGAAAAACCACCCTGGCGCCCAATACGCAAACCGCCTCTCCCCGCGCGTTGGCCGATTCATTAATGCAGCTGGCACGACAGGTTTCCCGACTGGAAAGCGGGCAGTGAGCGCAACGCAATTAATGTAAGTTAGCGCGAATTGATCTGGTTTGACAGCTTATCAT
CGACTGCACGGTGCACCAATGCTTCTGGCGTCAGGCAGCCATCGGAAGCTGTGGTATGGCTGTGCAGGTCGTAAATCACTGCATAATTCGTGTCGCTCAAGGCGCACTCCCGTTCTGGATAATGTTTTTTGCGCCGACATCATAACGGTTCTGGCAAATATTCTGAAATG
AGCTGTTGACAATTAATCATCCGGCTCGTATAATGTGTGGAATTGTGAGCGGATAACAATTTCACACAGGAAACAGACCATGGATGGCTTCCATTATTGGTAGCGGCTTTTTGATCATTGCACCACTGCTGCATTCGGTAATGGGGAAATGGGCTCTGCTTGGAATAGTG
TTGTTGTCCATTCTGGCATATGCGCTGGGGTCGGTTATTCGCTTCAATATACGCCATGCAGAACCTGAGCTAGCTAGCAATCAACAAAGCAGTATTGTGACGCTTGAAAAAGCAAGTCAATGGGCGCTTGGTGCGGCATATGCCATTTCCGTGGCGTTTTATATTAGTTT
GTTTGCAGCGTTTGTTTTTGATCGTTTATCAATCTCTGATACGACGTACATCAAACTGTTTACCAGTGGGTTGCTTGTCATCATTACGGTGGTCGCTTGGCTCCGTGGTGCTAGGGGGCTAGAGACCATTGAGCTATTCGCCGTGACAATAAAGTTGGCTATTATAGTAG
GTGTGCTCGCAGCGCTGGCAACCTACGACATACAAGTACAAAGTGCTTGGTTTCAACATGAAGCGATACAGGCATTGAGCCATTTTGAAACTGTTAGCATGCTCGCAGGCATGCTCATGGTAACGCAAGGGTTCGAGACAACCCGTTTTATGGGGAATAACTACACGCCA
GAACAGCGCATTAAAGCGAGTCGTTATGCTCAGTGGATCGCTATTTTTCTCTATGTTGTTTTTATCGGTCTAACGTGTCCTATTTTTCTGGATTTTCCGATCACGGAATTAAACGAAACAACGATTAGCTACACCCTAGGGCAAGCAATATGGGTATTGCCGATTTTATT
ACTGGTTGCCGCCACGGCAAGTCAATTAAGTGCAGCATTAGCCGATACTATCGGTGGCGGAGGCCTCTTAAAAGAGCTTTTCCATTTACCTATCTCCCCGCAATTTTACTACGTTTTAGTCATCGCCATCGCAGGTATATTAGTTTGGTCATCCAACGTATTTGAAATTA
TTAATCTCGCTTCAAAAGGGTTTGCACTTTATTACCTGATCCAAACAATAATCGCGGTCAAACTCGTGATGCGTCAAAGCACAGGTCAACGCATTAGGTCAACAAAATTACTTGGTCTATCGGTGATCGTTCTCTGTTTATTCTTTGTGATTGGCTGGTCAATTCCAGCA
CCTCATAGCTAATAAGGAAAATACCGATGCTATCAGTAGAAACTACACCACCAATTGACCAAATCAAAAACGCGGCTAAGCGCTACGCTATCATTGGTGTTGGAAATCTATTGCAGCGAGATGATGGCGTTGGTGTGCACGCGGTTCGTGCTATGCAACCCCTGCTAAAG
TCTTATTCAAATGTATCTTGCATTGACGCTGGGACACTGAGCTATGAACTTTTGGAGTGGGTTGCGAGTTCCGATCATACGATAGTCATCGACGCGGCTTATATGCGCTGTCCGCCTGGCACGGTGAGAGTGTTTGAGAACGAGGCAATTGCGGATCAGTTTCAGCAAGC
GACGACCCATTCCGTTCACCAGATCACGTTACGCGACACATTGTTGACCAGCCAAACGTTATATTCAAAACCTTCTGCGGTGACGTTAATTGGCGTAGAGCCGGAGGCGATTGAGTGGGGAACCTCTTTGAGTCCAAAGGTAAATGCAGCGCTATCATCGATCATGGATA
TCGCATTTGCTTGCTTATCAGGCGACAACGTTCAGACCTATAAACCGGCAACCCAAAAGGAGGCACGATGACCGAAGAACTCATTTTTATGACATCAAATGCTGATAAAACGGGCAACGTAATGCCGCTTTTGCATCAGATACGTCATGCGTTGTCTCAGTTAATTGAAC
GACAAGAACAAACCACGATTGATCTGCGACGATTGCCTTTAAGCGCTAGCGAAGAAGCGCAGTTAGAAGCTTTTCTTGGGCATGGAGAGGTTAAAGCTGATATTCAGGCGCTGGGTGATACGGTGCTAATCGAATCACGCTACGCAGGCGTTTGGCTTGAAATTCACTAT
AACGAAGATGTGGAGATCATGGGCAAATACGTTCATATCTGTACCTGTCCACCAATCATAAAATCTCAGCCAGAAGATATGGTGTTATCGCTCAGCAATATAGTGTCTGACATCCATTCGTTGTCTCATCAATCTTCTGATGAAACGGCTAAGGAGGATTGAAATGGCGC
TACCAACATTAAACAAGCAGTTACAAGCGTCCGGTATCTCAAGACGCACATTTCTTAAGTTTTGCGCTACAACGGCGTCTTTACTGGCATTGCCGCAAAGCGCTGTTGCCGATTTGGCGACTGCCCTTGGCAATGCGAGAAGACCCTCTGTGATTTGGTTGCCCTTTCAA
GAGTGCACAGGGTGTACCGAAGCAATATTGCGCTCTCATGCTCCCACATTGGAAAGCCTTATTTTCGATCATATTTCGTTGGATTATCAGCATACGATAATGGCTGCTGCGGGAGAGCAAGCTGAAGACGCTAGGCGTGCGGCGATGAACGCGCACAAAGGGCAATATTT
GTTGTTGGTTGATGGTTCGGTTCCGGTGGGTAACCCAGGATACTCAACGATCAGTGGCATGAGTAATGTCGATATGCTGAGAGAATCGGCAAAAGATGCTGCGGGTATTATTGCCATCGGTACCTGCGCGTCTTTTGGCGGGATCCCTAAAGCAAACCCAAATCCGACGG
GGGCAGTGGCAGTAAGCGACATTATTACAGACAAGCCAATCGTAAACATTTCAGGCTGTCCGCCACTGCCTATCGCGATTACAGCTGTGTTGGTTCATTACCTGACGTTTAAGCGTTTCCCTGATCTCGACGAATTACAACGCCCACTCGCTTTTTTTGGTGAAAGCATC
CATGACAGATGTTATCGACGCCCATTTTTTGAACAACGTAAATTTGCAAAATCGTTTGATGATGAGGGGGCAAAAAATGGGTGGTGTTTGTTCGAACTTGGTTGTAAAGGGCCCGAAACCTTTAACGCATGTGCAACGGTTAAATGGAATCAAGGCACGAGTTTTCCTAT
CGAATCTGGCCATCCGTGTCTTGGTTGCTCTGAGCCCGATTTCTGGGATAAAAGCAGCTTTTACCAAGCCTTGGGTCCATGGGAGTGGTACAAATCCAAACCCGGCAAAGGTGCACAGAAGCATGCTGGGAAAAACTCATAAGATAATAGGCAAGGCTCATGGAAAATAC
AGCAAGTAACAACCGGTTAGTCGTCGATCCTATCACTCGAATTGAAGGGCATCTTCGAATAGAAGCTGAAATGGATGGGAATACCATCAAACAGGCGTTCTCATCAGGCACGTCTGTTCGGGGAATTGAACTGATTTTACAAGGCAGAGATCCGCGTGACGCTTGGGCTT
TTGCGCAACGTATCTGTGGCGTCTGCACGCTGGTGCATGGTATGGCATCGGTGCGTGCTGTCGAAGATGCAATTAGAAAAGCTTGGCGGTCAAACGCAAAATTAGGGGTTGCCATTGGAAAGCCCTCCATGACATCTATGCCAAAAGGACCGATGCAACATGGTAAAAAA
GGGCACCGACAGTCACGTACTTCAATAGGCGTACTGAGTGAAGCAGAAATGGCTATCCCTCAAAATGCACAACTGATAAGAAACATCATGATTGCAACCCAATATGTGCATGATCACGTGATGCATTTTTATCACTTGCATGCCCTAGATTGGGTTGACGTTGTTTCTGC
ACTAGATGCAGATCCAACGAGAACCGCTACGCTCGCCGGTCAATTGAGTGATTATCCTCGTTCATCGCCGGGATATTTCAAAGATGTGAAGCAAAAAGTCAAAACGCTGGTTGAGTCTGGGCAGCTAGGGATATTCAGTAACGCGTATTGGGGGCATCCTGGCTATAAAC
TGCCACCTGAAGTTAACTTGATGGCATTAGCACATTATTTAGATGCGCTAACGTGGCAGCGTGAAGTTGTAAAAGTTCACACCATATTTGGAGGGAAAAACCCTCATCCTAATTTTGTCGTTGGCGGCGTGCCTTCACCGATTAATCTCAATGCGTCAACGGGTATTAAC
ACGAGTCGATTAGTGCAACTACAAGATGCTATCACGCAAATGAAGAGCTTTGTCGATCAGGTGTATTACCCCGATATTGTGGCGATTGCGGGTTATTACAAAGAGTGGGGGACACGAGGGGAAGGGCTGGGTAACTTTCTTACCTATGGAGACTTACCTATGACATCAAT
GGATGACCCTGATTCTTTCTTGTTTCCACGAGGTGCAATACTTGGTCGAGACTTGAGTAAAGTGCATGACCTTGATCTAGATGATCCCTCTGAAATTCAAGAATTCGTCTCTTCCTCCTGGTATCGATATAGTGGAGGGAACGCAAGTGGTTTACACCCTTTTAATGGAC
AAACAACACTCGAGTATACTGGTCCGAAACCGCCTTACAAGCACCTAAATGTAGGGGCTGAATATTCATGGTTGAAAAGTCCGCGTTGGAAAGGCCATGCGATGGAGGTGGGACCGCTGGCTCGCGTGCTAATGATGTATGCTAAAAAAGATGCCGCTGCGCAAGACATC
GTTAATCGATCTCTTTCTATCTTGGATTTAGAGACCTCTGCACTTTTCTCCACACTCGGTAGGACGCTCGCCAGAGCAGTGGAAACAAAAATTGTGGTTAACCAGCTACAGTCTTGGTATGACCAACTATTGGATAATATCGCAAAGGGCGATACCGATACGTTTAACCC
TCTGTATTTTGACCCTACCAATTGGCCAATTAAAGGCCAGGGGGTAGGCGTGATGGAAGCGCCTCGTGGCGCGTTGGGGCATTGGTTGGTCATGCAAAATGGCAAAATTGAGAATTACCAATGTGTCGTGCCTACGACATGGAACGCTGGACCTCGAGATCCCAACTCAC
AGGCAGGTGCTTATGAAGCCGCTCTGCAAGATAAACATACGCTACATGATCCTGACCAACCTTTAGAGATTTTGCGAACACTTCATAGTTTTGACCCCTGCTTAGCATGTGCCGTGCACGTAATGGACGAAACAGGGGAAGAGCGTTTGCGTCTAAAAGTTCGTTAACCT

TACTTGAGGAATATTATGAAAATCAAAAATAGTATTAGAAAGTCAGTTACTTCTTTAATTGCCTTTTTTTACCTGGTGGTAGGTTCACCCGTTAGTTATGCGCACCCGGGTCATCAAGGCGATCATTCACTACTTTTGGGATCGCTGGTGTCAACAATCGTGCTTGTCAC
CGTTGCGCTGTTTATTTATCGAGCTAGAGCAAGTAGGCCCTATCAACGGGTAAAACAGTAATGTGTCTCGCTATCCCCATGAAAGTCATTGCAATTAAAGGCTTTAACGCAACGTGTGAAGCGAAAGGTGTCTCTCGCGAGGTGAGTTTACATTTAGTGCAAGGGCTAGA
GGTCAAAGTTGGCGACTATGTGATGGTACATGTTGGTTATGCTCTCCAAGTGATCACCTATGATGAAGCGCAGGTGACATGGGAGATGCTCGATCAGGTCATTGCTTACGATGCATGAAATTAGTCTTTGCTATAGCTTGCTTGACACAGTCGCTGTTCACCAGCGAGCA
AACATGAATAAGTCTGTCAGTCTGGTTCACGTCAAAGTCGGCCCACTGTCAGGTGTTGAGCCTGACTTGCTGCACCATGCGTTCTTGGCGTGTAGAACTCACACCATTGGCGATCAGGCAACGCTTAGAATTGACACAAGCGCTATCAAGATACGCTGTAAATTGTGTGG
CGAATTCAGCCGAGTTTCGGTGAATAGCATCCTATGTGCAAGTTGCGGTGCTTGGCAAACCGACCTCATTGAGGGCGATGAATTTATATTGCAACGAATAGAGTTTATCGCACCTGACAATTTTACAGAATCAAAGGAGAGAAAACATGTGCGGTAACTGTGGTTGCGAA
ACGCAAGTAAAATCAGAGAAAACTGACAAGCCATCAGCAAATATCGTTGAGGTTCACGCAAATTTAAAAAGCAACAATGACAACCAAGCGATAGCAAACCGTGCTTTATTTGACGCACATAATGTACTAGTGATTAATTTGATGTCATCCCCAGGCAGTGGAAAAACCAG
GCTACTCGAAGAAACGATACGTGCGCTTAAGTCGCAATACGCGATGGCGGTGATTGAAGGCGACCTCGAAACTGAAAATGACGCAAATAGAATTAGACGTCATGGTGTGCAGGCGGAGCAAATCGCCACGGGCCAAGGGTGTCACTTGGACGCCAGTATGGTGCAAAAGG
TACTTGGAAGATTTAGCTTACAAAAACTAGACGTTTTGTTTATAGAGAATGTTGGCAATTTGATTTGTCCAGCGTGTTTTGATTTAGGACAGCACTTAAACATTATTTTGCTGTCCGTACCAGAAGGTGATGATAAGCCTGAAAAGTACCCAGTGATGTTTCGTGCCGCG
GACGTCATGCTTATTTCTAAGACCGATTACTTGCAATTTCACGATGAATTCAATGTACCTCGCGCGATTGAATCATTTCGTAAAGTGGGCAACGATGCGCCCGTTCTCGAAGTGTCTTCATTGAAAAGTCAACATCTAGAGCGTTGGTTTTCTTATTTTACGCACGCCAT
TACAGCTCATCAGTCTCAGTCGTTACAAAATAGCGGAGTCTAAAGTCTTATGTACAGTGTTGAATCGTTGCTAAGAGACATTAAGAATACCTCGCTTTCAAAACCGTTTCGTATTCTTAATGTTTGCGGTGGCCATGAGCGTGCGATTACGCGCGCTGGATTTAGAACGT
TGTTTCAGCACAATATTCATCTGATACCTGGGCCAGGGTGCCCCGTTTGTATTTGCCCCGAAGAGGATATTGCATACGCGATACACTTAGCCATTAACGAAAATGTCGTTATCGTTAGTTTTGGTGACATGCTCCGCGTCCCAGTCGAACACGAAATGGGTGGCTGTAGC
TCATTGATAGACGCAAAAAATCAAGGGGCCGATATCCGTCCGATATCTTCTCCTCAAGAAGCGGTATCTATAGCATTGGAAGAACCTCGTAAGGTTATTGTGTTTTTTGCTGTTGGTTTTGAAACGACTATGGCACCGATTGCAGCAACACTGCTTAATGATTTACCCAA
TAACTTTAAAGTATTATTGTCTGGACGACTTACTTGGCCAGCAGTTGCCCATGTGCTTGAGAGTCAACCGAATACTTTTGATGCACTGATTGCGCCAGGCCACGTTGCGACAATTATGGGTAGTGAAGAATGGCAGTTTGCAATCGACCATCATAATTTGCCTGTCAGTA
TTGCTGGTTTTCATCCAGAAAGTTTGCTGTTGTCGCTCCAAACGCTGCTCGGTAATTGTTCAAATAAAGTCGTCACACTGAGTAACCGCTATCCCGAAGTGGTAAAGCAAAACGGCAATGCAGCAGCTAAAGCAATAATCAATAAAGCGTTCACTATCGTTGACGCACAT
TGGCGAGGAATTGGTGTTATTCCTGGTTCTGGATTTTCCTTTGCTTCGAGACTATCGCACCTAGACGCCACAAACGATTATGCGCCAGTCGATTTCCCTTCGCAATGTGCGCAAAACGTTCCTGAAACCACATCGCCCTGTGAAAAAGTGATCCTCGGAAAAATGGCACC
TGATGCGTGCCCGTTTTTTGGTCAGGAATGCAAACCAGCATCCCCTAAAGGCGCATGTATGGTGTCTGATGAGGGGGCTTGCCGAATTTGGTATAGCTCTGGTGAGCGCTCGATAACAAATGTTATCAAAAAGGGGAATACGCTAAAGGTGGAGATGAAGTGATGACAAC
TGCCCGCACATATGTTGCCAATACGAATAAGAGTAAAACTATCGCCTATGAGTTGAACATTACTGGAGTCATCCAGGGGGTTGGGTTCAGACCGTTAGTTTACTGCTTAGCGTATGAAAAAAGTATTGTTGGTTGGGTACAAAACGACTGTGGATGTGTGCGAATACACG
CTGAAGGCACTGAGCTAGACGTTGAACAATTTGTATTCGACTTACTGAACAATGGGTCGCTAGTTTCCATATCCTTGCTGGAAAAAAAGCAGGTTAAACTGAGTAATGTAGACTCGTTCACGATAAAAGAAAGCGGTCATGATATAGCGTGCGGGGCGATATCAATCCCC
AAAGATGTCTATTTGTGTGGAGCGTGTAAAGCTGAGTTACTGTCAAATACTAACCGACGTGGCACCTATAGCTTTATCGCTTGCAGTAAATGTGGTCCACGATTTTCTATGCTGCGAGCAATGCCATACGACAGAAAGAACATGAGTATGTCAGCATTTCCAATGTGTGA
GTTATGTCGTAAAGAATATCAATCGCCAACTGATAGGCGCTTTCATGCTCAACCAATAAGCTGTCGCGTATGCGGGCCTGAAGTATTTTGTTCAACTGTCGGCGGGCGCGTGGTCTCGCAAGGCGATGTTGATGTTGTAGCAACGGTTGTCGCGTATTTGAATCAAGGCA
AAATTGTCGCGTTAAAAAGTATAGGCGGTTATCACTTGCTCTGTGACGCACAAAGCACTGAAGCGGTAGACCTTTTACGCCAGCGTAAAAATAGACCAGATAAACCTTTCGCGGTCATGCTCCCAGAGCCTCGTGCAGACGAAACAAGTGAGGATTGGTTAGATAAATGC
GTTGCTGTAGACAGTCATCAAAGAGCGCTCTTACTAAGCCCCGTGCGACCCATTTTACTTGCAAAAAAGCAATCGAATGTACCCATTGCAGATAACGTAGCGCCCATGCTTCGTGATTTGGGGGTGATGTTGCCGTGCAGCGGACTGCATTTACTCATTATGCAGCAATT
CAATCGCCCCATGGTGGCTACGTCAGGAAATATTTACGGTGAACCGATGGTCACATCGACGTCATCGGCACAGCAACACCTTTCGATGTTGGCAGACATCTTCGTGCACCATAATCGAGACATTTTTCACAAACTGGATGATTCTGTGCTGCGAACGCTTAAGTCAGGCA
CCATTCCAATTCGCCTGGGGCGGGGTATAAGTCCGATAGAATTATCATTGCCATATACTGTGCAGGAACCAATGCTTGCTGTTGGCGCGAATCAGAAAAATACAGTCGCAATAGCTTTGAACAATCGACTTATCGTAACACCGCATATTGGCGACCTCGATTCTCCGATA
ATGCAAGCGCACTTTGAAAAATGTATCAGTGACTTTCAAGCTCTCTATCATGTTCATCCACAGCGTATTCTTTGCGATAGCCATCCTGGCTATATCAGTTCCCGTTGGGCCAAAGGCAGTGAACTACCGACTACTAAAGTTTTACATCACCACGCTCATGCATCAGCGTG
GGCATTAGCTGCTGAAATATCGACACCAAGCTTGGTTTTCGTGTGGGATGGAACGGGATTAGGCGAGAATGGCGAGATGTGGGGAGGGGAAGTTTTTTGTGGGACGCCGGGCAATTGGAAACGTGTTGCCTCATTAGCGCCTATGAAGTTGCAAGGCGGCAACAAGGTTC
CAAATCAACCATGGCGAAGTGCAGCATCACTAGTGTGGGGTACTAATTTCACCGGCATTGATTTTAAACCATTTGATCCACAACAATTAAGCTACAACGCATGGCAAAACGAACTCAATTGTCATGTTAGTAGTTCAATGGGAAGACTGTTCGACGCGGCGGCATTTGCC
TGCTGTGAACAGAGCGAAGTCTCTTATGAAGGGCAGGCGCCGATGATGTTGGAAAGCGTTGCGTCGACGCCCTCTAAGGTTATGCCGTTAACGTTGGTCCAGAAAAACGACGAATTTATTCAAATGGATTGGCGAACACTGGTACCGGAACTATTTGATTTCAAAGTGTC
TCCTCAAACTCGTGCTGCAAATTTCCACGCTTCGTTAATTGAAACGGTATGGGAGGTAACCCTACATTGCCGTAAAAAGTTTAATTTTGACCACATTGGATTGGCAGGAGGCGTCTTCCAAAATCGCATTTTGTGTGACGGTATATCACAACGGTTTTTACAATCAGACA
TTCCTGTGGTTATTCCAAGTAGTTTGCCATTAAACGATGCCGCAATAAGTGTTGGGCAAATCCACGAATACTGTTCACGGAGGTCATAATGAAAAGCGCAGAGGGCAATAATCATTCATCATCAGAGCCGTTGGGCACGGAATCTATTCAATTAGCTCAGGGGAACGGCG
GAACACTGACAAAAAGACTCATTGATCATGTTTTTAAAAATAAAGCGAACAAAGATCTAGATCTGCTTCATGATGCCGCCACAGTAACTTTTGATGGTCCTTATCTGAGTATAACAACGGATAGCTTCGTTGTATCCCCCCCAACATTTCCAGGCGGCAATGTAGGCGCG
TTGAGTGTTTACGGTACCGTCAATGATTTAGCAGTCGTTGGGGCAACACCACGGTACATCAGTACTGCATTTATTATTGAAGAAGGCTTTTCCCTTTCAACATTGAAACAAATCGTTAATGGAATGCATCAAGCTGCCGAAGAAACGAAGGTCGCCATTGTAACAGGTGA
TACGAAAGTTGTCCCGAAAGGCAGTGGTGGCGGCGTTTTTATCAATACAACGGGTGTTGGAGACAGCCGAGATGCGCCGATTTGGGATACCAGTCTTATAAAGGCAGGCGATCATGTACTGGTTAGTGGTTCTGTAGGTGATCACGGTGCGTGTGTCCTGCTTGCTCGCG
AGGACTATGGTTTGCAAGGTCAGCTAAAGTCTGATTGTGGAAGCGTTGTTCCTCTTATTGATCCAATCAAGCATTTGGAAGGTGTTCGTTTTGTGCGTGACCCAACTCGTGGAGGGTTGTCAGTATTACTCCATGACGTTGCTAATGAAACAGGCTTCGATATCGAGTTA
ATTGAAAATAATATTCCGGTGCGACCTGAAGTGGCAAGCGTGTGTGAAATATTAGGCTTTGATCCGTTTGTTTTAGCGTGTGAGGGACGCATTGTTGCTGTTGTTGCCCCTGACATAAGCGGTAAGGTGCTAGAACACTGGCGGAGCATTCGAAACGGGGAGCAGGCCGA
ACACATTGGTGTAATAGTAAAAAACACCGAATCAAAAGGGCGCGTGACCATTGTTACGCCAATGGGAGGAAGACGTTTCATGAATGAACTCGAAGATGAGCCGCTTCCAAGGATCTGCTGAGATCCTCTAGAGTCGACCTGCAGGCATGCAAGCTTGGCTGTTTTGGCGG
ATGAGAGAAGATTTTCAGCCTGATACAGATTAAATCAGAACGCAGAAGCGGTCTGATAAAACAGAATTTGCCTGGCGGCAGTAGCGCGGTGGTCCCACCTGACCCCATGCCGAACTCAGAAGTGAAACGCCGTAGCGCCGATGGTAGTGTGGGGTCTCCCCATGCGAGAG
TAGGGAACTGCCAGGCATCAAATAAAACGAAAGGCTCAGTCGAAAGACTGGGCCTTTCGTTTTATCTGTTGTTTGTCGGTGAACGCTCTCCTGAGTAGGACAAATCCGCCGGGAGCGGATTTGAACGTTGCGAAGCAACGGCCCGGAGGGTGGCGGGCAGGACGCCCGCC
ATAAACTGCCAGGCATCAAATTAAGCAGAAGGCCATCCTGACGGATGGCCTTTTTGCGTTTCTACAAACTCTTTTGTTTATTTTTCTAAATACATTCAAATATGTATCCGCTCATGGCGGCCCAGATCCCCGGGTACCGAGCTCGAATTTCGAGCTTCTGGAGCAGGAAG
ATGTCGCGGGCATTAGCACCAGCGGTCTGCCAAGCCTCCGCCAGCCGTTGGGTCCCTTCCGCTTGAGCTTTTCCATCTTCGACGATACGGGCGGCGGCCCCCCGCGCTTCCGCGATCGCCCGTTTACAAGCTGCCTCAGCTGGGGCGATCACATCGGCTTGAAGTTGCTG
CTGCACCTGTTTGATCCGCTCCTGCTGCACAGGGAGTTCTGCTTGGCTACGAGCGACTTCGGTAGCAATGTCCGCTTCAGCTTCGGCCACCACCGCTTCGCGCCGCGTCAACGCATCCTGAATCCGGCGCTCGGCCTCGGCTTGGGCGATCGCTACATCGCGATCGATCC
GACGCAGGGCCGTGATCTTGTCATTTTCGGCCGTTTGGATCGCAGAGGCAGCCTGGGCATCGGCTTCAGCAATTCGGGCATCTCGCTGCAGATCAGCCCGCTGCTTGCGTCCACTAGCCGAGAGATAACCGACCTCATCGGAAATGTTCTGGACTTGCAGCGTATCGAGG
ACTAGACCCAGCTGCTCAAGGTCATCCTCCGCCTCTTCCAGCAGACTTTTGGCAAAGGCAATTTTGTCCTCGTTGATCTGCTCCGGCGTGAGGCTGGCTAAAACACCACGCAAGTTGCCTTCGAGGGTCTCCTTGGCAATTTGCTCGATTTCCTTACGGTTTTTGCCAAG
CAGCCGCTCGATCGCGTTGTGGATGGTCGGTTCTTCCCCAGCAATCTTGATATTGGCAACGCCTTCAACAGTCAGGGGAATGCCGCCCTTGGAGAAGGCATTGGAAACGCGCAACTCAATGATCATGTTGGTCAGATCCATGCGGAGCGCTTTTTCCAGCAGAGGTACCC
GCAGGCTGCTGCCGCCCTTGACCAAGCGATAGCCAACTCGGCGGCCATCACTACTGCGGCGACTACTGCCAGCAAAGATCAAAATTTCACTGGGTTGGCAGATGTAGTAGAGATTGCGCAGGACTAAGCTGCCAGCCCCGGCGACTCACCAGTCACAGAAAAGCATCTTA
CGGATGGCATGACAGTAAGAGAATTATGCAGTGCTGCCATAACCATGAGTGATAACACTGCGGCCAACTTACTTCTGACAACGATCGGAGGACCGAAGGAGCTAACCGCTTTTTTGCACAACATGGGGGATCATGTAACTCGCCTTGATCGTTGGGAACCGGAGCTGAAT
GAAGCCATACCAAACGACGAGCGTGACACCACGATGCCTGCAGCAATGGCAACAACGTTGCGCAAACTATTAACTGGCGAACTACTTACTCTAGCTTCCCGGCAACAATTAATAGACTGGATGGAGGCGGATAAAGTTGCAGGACCACTTCTGCGCTCGGCCCTTCCGGC
TGGCTGGTTTATTGCTGATAAATCTGGAGCCGGTGAGCGTGGGTCTCGCGGTATCATTGCAGCACTGGGGCCAGATGGTAAGCCCTCCCGTATCGTAGTTATCTACACGACGGGGAGTCAGGCAACTATGGATGAACGAAATAGACAGATCGCTGAGATAGGTGCCTCAC
TGATTAAGCATTGGTAACTGTCAGACCAAGTTTACTCATATATACTTTAGATTGATTTAAAACTTCATTTTTAATTTAAAAGGATCTAGGTGAAGATCCTTTTTGATAATCTCATGACCAAAATCCCTTAACGTGAGTTTTCGTTCCACTGAGCGTCAGACCCCGTAGAA
AAGATCAAAGGATCTTC

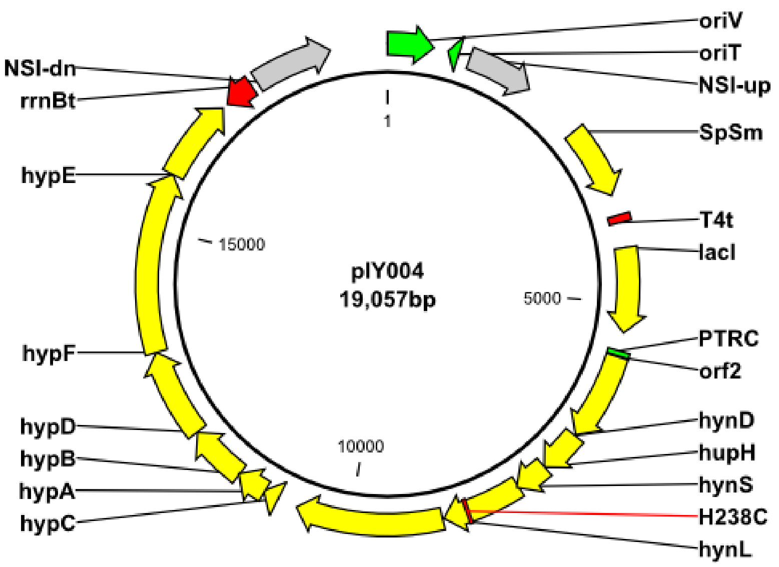

TTGAGATCCTTTTTTTCTGCGCGTAATCTGCTGCTTGCAAACAAAAAAACCACCGCTACCAGCGGTGGTTTGTTTGCCGGATCAAGAGCTACCAACTCTTTTTCCGAAGGTAACTGGCTTCAGCAGAGCGCAGATACCAAATACTGTCCTTCTAGTGTAGCCGTAGTTAG
GCCACCACTTCAAGAACTCTGTAGCACCGCCTACATACCTCGCTCTGCTAATCCTGTTACCAGTGGCTGCTGCCAGTGGCGATAAGTCGTGTCTTACCGGGTTGGACTCAAGACGATAGTTACCGGATAAGGCGCAGCGGTCGGGCTGAACGGGGGGTTCGTGCACACAG
CCCAGCTTGGAGCGAACGACCTACACCGAACTGAGATACCTACAGCGTGAGCTATGAGAAAGCGCCACGCTTCCCGAAGGGAGAAAGGCGGACAGGTATCCGGTAAGCGGCAGGGTCGGAACAGGAGAGCGCACGAGGGAGCTTCCAGGGGGAAACGCCTGGTATCTTTA
TAGTCCTGTCGGGTTTCGCCACCTCTGACTTGAGCGTCGATTTTTGTGATGCTCGTCAGGGGGGCGGAGCCTATGGAAAAACGCCAGCAACGCGGCCTTTTTACGGTTCCTGGCCTTTTGCTGGCCTTTTGCTCACATGTTCTTTCCTGCGTTATCCCCTGATTCTGTGG
ATAACCGTATTACCGCCTTTGAGTGAGCTGATACCGCTCGCCGCAGCCGAACGACCGAGCGCAGCGAGTCAGTGAGCGAGGAAGCGGAAGAGCGCCTGATGCGGTATTTTCTCCTTACGCATCTGTGCGGTATTTCACACCGCATATGGTGCACTCTCAGTACAATCTGC
TCTGATGCCGCATAGTTAAGCCAGTATACACTCCGCTATCGCTACGTGACTGGGTCATGGCTGCGCCCCGACACCCGCCAACACCCGCTGACGCGCCCTGACGGGCTTGTCTGCTCCCGGCATCCGCTTACAGACAAGCTGTGACCGTCTCCGGGAGCTGCATGTGTCAG
AGGTTTTCACCGTCATCACCGAAACGCGCGAGGCAGGATATCCGGCAGCCGGCGGAGCGCTGCTTTCTTGGCAAGCGGTCGCCAGCCCCAACGCCAGGGCTGCCAGCCCGAAACAGCGGGGCAAGGCAGCTTGGAAGGGCGATCGCAGCACGGGCATGGCAATGTCTCTC
TGAAGGAATGCAGACCTTATTCGTACAGCCAGGGTTGAATCGTGGGGGTCCAATCACTTAGCTCTGCTGGGCTAAACCAGAGAGCAATTTCCTGTTGTGCTGTTTCGATTGCATCCGAGCCATGGATGATGTTGCGGCCAATATTGACACCAAAATCACCACGGATGGTG
CCCGGTTCTGCCGTCAGCGGATTGGTAGCGCCGATCAACTTGCGAGCAGCCGCCACAACGCCTTCGCCTTCCAAGACGATCGCCACGATCGGCCCAGAGGTGATGAACTCGACGAGGCCATTGAAGAAGGGGCGCTCGCGGTGGACAGCATAGTGCTGTTCGGCCAGCTC
GCGACTGGGCTTCAGCTGCTTTAGGCCCACCAGTTTGAAGCCTTTTTGCTCAAAGCGGCCGATGATCGTACCGACCAAACCCCGCTGAACGCCATCGGGCTTGATGGCAATAAATGTGCGTTCCACAGACATCTAGATAGTCCTCAAGACGAGGCAAGCATTGAGCTTGC
CTTCCTATGGTTCGGGATCACTGGGATTCTTGACAAGCGATCGCGGTCACATCGCTATCTCTTAGGACTTCGCAGCGGGCGAGTCGGATTGACCCGGTAGGGATTTCGCCAGATCAATGCCCGTGGTTTGTTTCAGCTTCTCCAGCAAGCTAGCGATTTGGGTAGCGCTG
CCTTCCCCTTCGCCAATCACAGTGATCGACTCCACGTCGATATCTGGCACGGTGCCTGAAAGCGTGACGAGCAGGGACTCGAAGCTTGCATGCCTGCAGGTCGACTCTAGAGCTTTATGCTTGTAAACCGTTTTGTGAAAAAATTTTTAAAATAAAAAAGGGGACCTCTA
GGGTCCCCAATTAATTAGTAATATAATCTATTAAAGGTCATTCAAAAGGTCATCCACCGGATCAATTCCCCTGCTCGCGCAGGCTGGGTGCCAAGCTCTCGGGTAACATCAAGGCCCGATCCTTGGAGCCCTTGCCCTCCCGCACGATGATCGTGCCGTGATCGAAATCC
AGATCCTTGACCCGCAGTTGCAAACCCTCACTGATCCGCATGCCCGTTCCATACAGAAGCTGGGCGAACAAACGATGCTCGCCTTCCAGAAAACCGAGGATGCGAACCACTTCATCCGGGGTCAGCACCACCGGCAAGCGCCGCGACGGCCGAGGTCTTCCGATCTCCTG
AAGCCAGGGCAGATCCGTGCACAGCACCTTGCCGTAGAAGAACAGCAAGGCCGCCAATGCCTGACGATGCGTGGAGACCGAAACCTTGCGCTCGTTCGCCAGCCAGGACAGAAATGCCTCGACTTCGCTGCTGCCCAAGGTTGCCGGGTGACGCACACCGTGGAAACGGA
TGAAGGCACGAACCCAGTGGACATAAGCCTGTTCGGTTCGTAAGCTGTAATGCAAGTAGCGTATGCGCTCACGCAACTGGTCCAGAACCTTGACCGAACGCAGCGGTGGTAACGGCGCAGTGGCGGTTTTCATGGCTTGTTATGACTGTTTTTTTGGGGTACAGTCTATG
CCTCGGGCATCCAAGCAGCAAGCGCGTTACGCCGTGGGTCGATGTTTGATGTTATGGAGCAGCAACGATGTTACGCAGCAGGGCAGTCGCCCTAAAACAAAGTTAAACATCATGAGGGAAGCGGTGATCGCCGAAGTATCGACTCAACTATCAGAGGTAGTTGGCGTCAT
CGAGCGCCATCTCGAACCGACGTTGCTGGCCGTACATTTGTACGGCTCCGCAGTGGATGGCGGCCTGAAGCCACACAGTGATATTGATTTGCTGGTTACGGTGACCGTAAGGCTTGATGAAACAACGCGGCGAGCTTTGATCAACGACCTTTTGGAAACTTCGGCTTCCCC
TGGAGAGAGCGAGATTCTCCGCGCTGTAGAAGTCACCATTGTTGTGCACGACGACATCATTCCGTGGCGTTATCCAGCTAAGCGCGAACTGCAATTTGGAGAATGGCAGCGCAATGACATTCTTGCAGGTATCTTCGAGCCAGCCACGATCGACATTGATCTGGCTATCT
TGCTGACAAAAGCAAGAGAACATAGCGTTGCCTTGGTAGGTCCAGCGGCGGAGGAACTCTTTGATCCGGTTCCTGAACAGGATCTATTTGAGGCGCTAAATGAAACCTTAACGCTATGGAACTCGCCGCCCGACTGGGCTGGCGATGAGCGAAATGTAGTGCTTACGTTG
TCCCGCATTTGGTACAGCGCAGTAACCGGCAAAATCGCGCCGAAGGATGTCGCTGCCGACTGGGCAATGGAGCGCCTGCCGGCCCAGTATCAGCCCGTCATACTTGAAGCTAGACAGGCTTATCTTGGACAAGAAGAAGATCGCTTGGCCTCGCGCGCAGATCAGTTGGA
AGAATTTGTCCACTACGTGAAAGGCGAGATCACCAAGGTAGTCGGCAAATAATGTCTAACAATTCGTTCAAGCCGACGCCGCTTCGCGGCGCGGCTTAACTCAAGCGTTAGATGCACTAAGCACATAATTGCTCACAGCCAAACTATCAGGTCAAGTCTGCTTTTATTAT
TTTTAAGCGTGCATAATAAGCCCTACACAAATTGGGAGATATATCATGAAAGGCTGGCTTTTTCTTGTTATCGCAATAGTTGGCGAAGTAATCGCAACATCCGCATTAAAATCTAGCGAGGGCTTTACTAAGCTGATCCGGTGGATGACCTTTTGAATGACCTTTAATAG
ATTATATTACTAATTAATTGGGGACCCTAGAGGTCCCCTTTTTTATTTTAAAAATTTTTTCACAAAACGGTTTACAAGCATACTAGAGGATCTGCGTGCGGGCGCGGGGATCTGGGCCGCGGGTCATGGCTGCGCCCCGACACCCGCCAACACCCGCTGACGCGCCCTGACGGGCT
TGTCTGCTCCCGGCATCCGCTTACAGACAAGCTGTGACCGTCTCCGGGAGCTGCATGTGTCAGAGGTTTTCACCGTCATCACCGAAACGCGCGAGGCAGCAGATCAATTCGCGCGCGAAGGCGAAGCGGCATGCATTTACGTTGACACCATCGAATGGTGCAAAACCTTT
CGCGGTATGGCATGATAGCGCCCGGAAGAGAGTCAATTCAGGGTGGTGAATGTGAAACCAGTAACGTTATACGATGTCGCAGAGTATGCCGGTGTCTCTTATCAGACCGTTTCCCGCGTGGTGAACCAGGCCAGCCACGTTTCTGCGAAAACGCGGGAAAAAGTGGAAGC
GGCGATGGCGGAGCTGAATTACATTCCCAACCGCGTGGCACAACAACTGGCGGGCAAACAGTCGTTGCTGATTGGCGTTGCCACCTCCAGTCTGGCCCTGCACGCGCCGTCGCAAATTGTCGCGGCGATTAAATCTCGCGCCGATCAACTGGGTGCCAGCGTGGTGGTGT
CGATGGTAGAACGAAGCGGCGTCGAAGCCTGTAAAGCGGCGGTGCACAATCTTCTCGCGCAACGCGTCAGTGGGCTGATCATTAACTATCCGCTGGATGACCAGGATGCCATTGCTGTGGAAGCTGCCTGCACTAATGTTCCGGCGTTATTTCTTGATGTCTCTGACCAG
ACACCCATCAACAGTATTATTTTCTCCCATGAAGACGGTACGCGACTGGGCGTGGAGCATCTGGTCGCATTGGGTCACCAGCAAATCGCGCTGTTAGCGGGCCCATTAAGTTCTGTCTCGGCGCGTCTGCGTCTGGCTGGCTGGCATAAATATCTCACTCGCAATCAAAT
TCAGCCGATAGCGGAACGGGAAGGCGACTGGAGTGCCATGTCCGGTTTTCAACAAACCATGCAAATGCTGAATGAGGGCATCGTTCCCACTGCGATGCTGGTTGCCAACGATCAGATGGCGCTGGGCGCAATGCGCGCCATTACCGAGTCCGGGCTGCGCGTTGGTGCGG
ATATCTCGGTAGTGGGATACGACGATACCGAAGACAGCTCATGTTATATCCCGCCGTTAACCACCATCAAACAGGATTTTCGCCTGCTGGGGCAAACCAGCGTGGACCGCTTGCTGCAACTCTCTCAGGGCCAGGCGGTGAAGGGCAATCAGCTGTTGCCCGTCTCACTG
GTGAAAAGAAAAACCACCCTGGCGCCCAATACGCAAACCGCCTCTCCCCGCGCGTTGGCCGATTCATTAATGCAGCTGGCACGACAGGTTTCCCGACTGGAAAGCGGGCAGTGAGCGCAACGCAATTAATGTAAGTTAGCGCGAATTGATCTGGTTTGACAGCTTATCAT
CGACTGCACGGTGCACCAATGCTTCTGGCGTCAGGCAGCCATCGGAAGCTGTGGTATGGCTGTGCAGGTCGTAAATCACTGCATAATTCGTGTCGCTCAAGGCGCACTCCCGTTCTGGATAATGTTTTTTGCGCCGACATCATAACGGTTCTGGCAAATATTCTGAAATG
AGCTGTTGACAATTAATCATCCGGCTCGTATAATGTGTGGAATTGTGAGCGGATAACAATTTCACACAGGAAACAGACCATGGATGGCTTCCATTATTGGTAGCGGCTTTTTGATCATTGCACCACTGCTGCATTCGGTAATGGGGAAATGGGCTCTGCTTGGAATAGTG
TTGTTGTCCATTCTGGCATATGCGCTGGGGTCGGTTATTCGCTTCAATATACGCCATGCAGAACCTGAGCTAGCTAGCAATCAACAAAGCAGTATTGTGACGCTTGAAAAAGCAAGTCAATGGGCGCTTGGTGCGGCATATGCCATTTCCGTGGCGTTTTATATTAGTTT
GTTTGCAGCGTTTGTTTTTGATCGTTTATCAATCTCTGATACGACGTACATCAAACTGTTTACCAGTGGGTTGCTTGTCATCATTACGGTGGTCGCTTGGCTCCGTGGTGCTAGGGGGCTAGAGACCATTGAGCTATTCGCCGTGACAATAAAGTTGGCTATTATAGTAG
GTGTGCTCGCAGCGCTGGCAACCTACGACATACAAGTACAAAGTGCTTGGTTTCAACATGAAGCGATACAGGCATTGAGCCATTTTGAAACTGTTAGCATGCTCGCAGGCATGCTCATGGTAACGCAAGGGTTCGAGACAACCCGTTTTATGGGGAATAACTACACGCCA
GAACAGCGCATTAAAGCGAGTCGTTATGCTCAGTGGATCGCTATTTTTCTCTATGTTGTTTTTATCGGTCTAACGTGTCCTATTTTTCTGGATTTTCCGATCACGGAATTAAACGAAACAACGATTAGCTACACCCTAGGGCAAGCAATATGGGTATTGCCGATTTTATT
ACTGGTTGCCGCCACGGCAAGTCAATTAAGTGCAGCATTAGCCGATACTATCGGTGGCGGAGGCCTCTAAAAGAGCTTTTCCATTTACCTATCTCCCCGCAATTTTACTACGTTTTAGTCATCGCCATCGCAGGTATATTAGTTTGGTCATCCAACGTATTTGAAACTA
TTAATCTCGCTTCAAAAGGGTTTGCACTTTATTACCTGATCCAAACAATAATCGCGGTCAAACTCGTGATGCGTCAAAGCACAGGTCAACGCATTAGGTCAACAAAATTACTTGGTCTATCGGTGATCGTTCTCTGTTTATTCTTTGTGATTGGCTGGTCAATTCCAGCA
CCTCATAGCTAATAAGGAAAATACCGATGCTATCAGTAGAAACTACACCACCAATTGACCAAATCAAAAACGCGGCTAAGCGCTACGCTATCATTGGTGTTGGAAATCTATTGCAGCGAGATGATGGCGTTGGTGTGCACGCGGTTCGTGCTATGCAACCCCTGCTAAAG
TCTTATTCAAATGTATCTTGCATTGACGCTGGGACACTGAGCTATGAACTTTTGGAGTGGGTTGCAGGTTCCGATCATACGATAGTCATCGACGCGGCTTATATGCGCTGTCCGCCTGGCACGGTGAGAGTTGTTTGAGACGAGGCAATTGCGGATCAGTTTCAGCAAGC
GACGACCCATTCCGTTCACCAGATCACGTTACGCGACACATTGTTGACCAGCCAAACGTTATATTCAAAACCTTCTGCGGTGACGTTAATTGGCGTAGAGCCGGAGGCGATTGAGTGGGGAACCTCTTTGAGTCCAAGGTAAATGCAGCGCTATCATCGATCATCGATCGGATA
TCGCATTTGCTTGCTTATCAGGCGACAACGTTCAGACCTATAAACCGGCAACCCAAAAGGAGGCACGATGACCGAAGAACTCATTTTTATGACATCAAATGCTGATAAAACGGGCAACGTAATGCCGCTTTTGCATCAGATACGTCATGCGTTGTCTCAGTTAATTGAAC
GACAAGAACAAACCACGATTGATCTGCGACGATTGCCTTTAAGCGCTAGCGAAGAAGCGCAGTTAGAAGCTTTTCTTGGGCATGGAGAGGTTAAAGCTGATATTCAGGCGCTGGGTGATACGGTGCTAATCGAATCACGCTACGCAGGCGTTTGGCTTGAAATTCACTAT
AACGAAGATGTGGAGATCATGGGCAAATACGTTCATATCTGTACCTGTCCACCAATCATAAAATCTCAGCCAGAAGATATGGTGTTATCGCTCAGCAATATAGTGTCTGACATCCATTCGTTGTCTCATCAATCTTCTGATGAAACGGCTAAGGAGGATTGAAATGGCGC
TACCAACATTAAACAAGCAGTTACAAGCGTCCGGTATCTCAAGACGCACATTTCTTAAGTTTTGCGCTACAACGGCGTCTTTACTGGCATTGCCGCAAAGCGCTGTTGCCGATTTGGCGACTGCCCTTGGCAATGCGAGAAGACCCTCTGTGATTTGGTTGCCCTTTCAA
GAGTGCACAGGGTGTACCGAAGCAATATTGCGCTCTCATGCTCCCACATTGGAAAGCCTTATTTTCGATCATATTTCGTTGGATTATCAGCATACGATAATGGCTGCTGCGGGAGAGCAAGCTGAAGACGCTAGGCGTGCGGCGATGAACGCGCACAAAGGGCAATATTT
GTTGTTGGTTGATGGTTCGGTTCCGGTGGGTAACCCAGGATACTCAACGATCAGTGGCATGAGTAATGTCGATATGCTGAGAGAATCGGCAAAAGATGCTGCGGGTATTATTGCCATCGGTACCTGCGCGTCTTTTGGCGGGATCCCTAAAGCAAACCCAAATCCGACGG
GGGCAGTGGCAGTAAGCGACATTATTACAGACAAGCCAATCGTAAACATTTCAGGCTGTCCGCCACTGCCTATCGCGATTACAGCTGTGTTGGTTCATTACCTGACGTTTAAGCGTTTCCCTGATCTCGACGAATTACAACGCCCACTCGCTTTTTTTGGTGAAAGCATC
TGTGACAGATGTTATCGACGCCCATTTTTTGAACAACGTAAATTTGCAAAATCGTTTGATGATGAGGGGGCAAAAAATGGGTGGTGTTTGTTCGAACTTGGTTGAAGGGCCCGAAACCTTTAACGCATGTGCAACGGTTAAATGGAATCAAGGCACGAGTTTTCCTAT
CGAATCTGGCCATCCGTGTCTTGGTTGCTCTGAGCCCGATTTCTGGGATAAAAGCAGCTTTTACCAAGCCTTGGGTCCATGGAGTGGTACAAATCCAAACCCGGCAAAGGTGCACAGAAGCATGCTGGGAAAAACTCATAAGATAATAGGCAAGGCTCATGGAAAATAC
AGCAAGTAACAACCGGTTAGTCGTCGATCCTATCACTCGAATTGAAGGGCATCTTCGAATAGAAGCTGAAATGGATGGGAATACCATCAAACAGGCGTTCTCATCAGGCACGTCTGTTCGGGGAATTGAACTGATTTTACAAGGCAGAGATCCGCGTGACGCTTGGGCTT
TTGCGCAACGTATCTGTGGCGTCTGCACGCTGGTGCATGGTATGGCATCGGTGCGTGCTGTCGAAGACGCATTGAAAAGCTTGGCGGTCAAACGCAAAATTAGGGGTTGCCATTGGAAAGCCCTCCATGACATCTATGCCAAAGGACCGATGCACATGTATTAAAAAA
GGGCACCGACAGTCACGTACTTCATAGGCGTACTGAGTGAAGCAGAAATGGCTATCCCTCAAAATGCACAACTGATAAGAAACATCATGATTGCAACCCAATATGTGCATGATCACGTGATGCATTTTATCACTTGCATGCCCTAGATTGGGTTGACGTTGTTTCTGC
ACTAGATGCAGATCCAACGAGAACCGCTACGCTCGCCGGTCAATTGAGTGATTATCCTCGTTCATCGCCGGGATATTTCAAAGATGTGAAGCAAAAAGTCAAACGCTGGTTGAGTCTGGGCAGCTAGGGATATTCAGTAACGCGTATTGGGGGCATCCTGGCTATAAAC
TGCCACCTGAAGTTAACTTGATGGCATTAGCACATTATTTAGATGCGCTAACGTGGCAGCGTGAAGTTGTAAAAGTTCACACCATATTTGGAGGAAAAACCCTCATCCTAATTTTGTCGTTGGCGGCGTGCCTTCACCGATTAATCTCAATGCGTCAACGGGTATTAAC
ACGAGTCGATTAGTGCAACTACAAGATGCTATCACGCAAATGAAGAGCTTTGTCGATCAGGTGTATTACCCGATATTGTGGCGATTGCGGGTTATTACAAAGAGTGGGGGACACGAGGGGAAGGGCTGGGTAACTTTCTTACCTATGGAGACTTACCTATGGACATCAAT
GGATGACCCTGATTCTTTCTTGTTTCCACGAGGTGCAATACTTGGTCGAGACTTGAGTAAAGTGCATGACCTTGATCTAGATGATCCCTCTGAAATTCAAGAATTCGTCTCTTCCTCCTGGTATCGATATAGTGGAGGGAACGCAAGTGGTTTACACCCTTTTAATGGAC
AAACAACACTCGAGTATACTGGTCCGAAACCGCCTTACAAGCACCTAAATGTAGGGGCTGAATATTCATGGTGAAAAGTCCGCGTTGGAAAGGCCATGCGATGGAGGTGGGACCGCTGGCTCGCGTGCTAATGATGTATGCTAAAAAAGATGCCGCTGCGCAAGACATC
GTTAATCGATCTCTTTCTATCTTGGATTTAGAGACCTCTGCACTTTTCTCCACACTCGGTAGGACGCTCGCCAGAGCAGTGGAAACAAAAATTGTGGTTAACCAGCTACAGTCTTGGTATGACCAACTATTGGATAATATCGCAAAGGGCGATACCGATACGTTTAACCC
TCTGTATTTTGACCCTACCAATTGGCCAATTAAAGGCCAGGGGGTAGGCGTGATGGAGCGCCTCGTGGCGCGTTGGGGCATTGGTTGGTCATGCAAAATGGCAAAATTGAGAATTACCAATGTGTCGTGCCTACGACATGGAACGCTGGACCTCGAGATCCCAACTCAC
AGGCAGGTGCTTATGAAGCCGCTCTGCAAGATAAACATACGCTACATGATCCTGACCAACCTTTAGAGATTTTGCGAACACTTCATAGTTTTGACCCCTGCTTAGCATGTGCCGTGCACGTAATGGACGAAACAGGGGAAGAGCGTTTGCGTCTAAAAGTTCGTTAACCT
TACTTGAGGAATATTATGAAAATCAAAAATAGTATTAGAAAGTCAGTTACTTCTTTAATTGCCTTTTTTTACCTGGTGGTAGGTTCACCCGTTAGTTATGCGCACCCGGGTCATCAAGGCGATCATTCACTACTTTTGGGATCGCTGGTGTCAACAATCGTGCTTGTCAC
CGTTGCGCTGTTTATTTATCGAGCTAGAGCAAGTAGGCCCTATCAACGGGTAAAACAGTAATGTGTCTCGCTATCCCCATGAAAGTCATTGCAATTAAAGGCTTTAACGCAACGTGTGAAGCGAAAGGTGTCTCTCGCGAGGTGAGTTTACATTTAGTGCAAGGGCTAGA

GGTCAAAGTTGGCGACTATGTGATGGTACATGTTGGTTATGCTCTCCAAGTGATCACCTATGATGAAGCGCAGGTGACATGGGAGATGCTCGATCAGGTCATTGCTTACGATGCATGAAATTAGTCTTTGCTATAGCTTGCTTGACACAGTCGCTGTTCACCAGCGAGCA
AACATGAATAAGTCTGTCAGTCTGGTTCACGTCAAAGTCGGCCCACTGTCAGGTGTTGAGCCTGACTTGCTGCACCATGCGTTCTTGGCGTGTAGAACTCACACCATTGGCGATCAGGCAACGCTTAGAATTGACACAAGCGCTATCAAGATACGCTGTAAATTGTGTGG
CGAATTCAGCCGAGTTTCGGTGAATAGCATCCTATGTGCAAGTTGCGGTGCTTGGCAAACCGACCTCATTGAGGGCGATGAATTTATATTGCAACGAATAGAGTTTATCGCACCTGACAATTTTACAGAATCAAAGGAGAGAAAACATGTGCGGTAACTGTGGTTGCGAA
ACGCAAGTAAAATCAGAGAAAACTGACAAGCCATCAGCAAATATCGTTGAGGTTCACGCAAATTTAAAAAGCAACAATGACAACCAAGCGATAGCAAACCGTGCTTTATTTGACGCACATAATGTACTAGTGATTAATTTGATGTCATCCCCAGGCAGTGGAAAAACCAG
GCTACTCGAAGAAACGATACGTGCGCTTAAGTCGCAATACGCGATGGCGGTGATTGAAGGCGACCTCGAAACTGAAAATGACGCAAATAGAATTAGACGTCATGGTGTGCAGGCGGAGCAAATCGCCACGGGCCAAGGGTGTCACTTGGACGCCAGTATGGTGCAAAAGG
TACTTGGAAGATTTAGCTTACAAAAACTAGACGTTTTGTTTATAGAGAATGTTGGCAATTTGATTTGTCCAGCGTGTTTTGATTTAGGACAGCACTTAAACATTATTTTGCTGTCCGTACCAGAAGGTGATGATAAGCCTGAAAAGTACCCAGTGATGTTTCGTGCCGCG
GACGTCATGCTTATTTCTAAGACCGATTACTTGCAATTTCACGATGAATTCAATGTACCTCGCGCGATTGAATCATTTCGTAAAGTGGGCAACGATGCGCCCGTTCTCGAAGTGTCTTCATTGAAAAGTCAACATCTAGAGCGTTGGTTTTCTTATTTTACGCACGCCAT
TACAGCTCATCAGTCTCAGTCGTTACAAAATAGCGGAGTCTAAAGTCTTATGTACAGTGTTGAATCGTTGCTAAGAGACATTAAGAATACCTCGCTTTCAAAACCGTTTCGTATTCTTAATGTTTGCGGTGGCCATGAGCGTGCGATTACGCGCGCTGGATTTAGAACGT
TGTTTCAGCACAATATTCATCTGATACCTGGGCCAGGGTGCCCCGTTTGTATTTGCCCCGAAGAGGATATTGCATACGCGATACACTTAGCCATTAACGAAAATGTCGTTATCGTTAGTTTTGGTGACATGCTCCGCGTCCCAGTCGAACACGAAATGGGTGGCTGTAGC
TCATTGATAGACGCAAAAAATCAAGGGGCCGATATCCGTCCGATATCTTCTCCTCAAGAAGCGGTATCTATAGCATTGGAAGAACCTCGTAAGGTTATTGTGTTTTTTGCTGTTGGTTTTGAAACGACTATGGCACCGATTGCAGCAACACTGCTTAATGATTTACCCAA
TAACTTTAAAGTATTATTGTCTGGACGACTTACTTGGCCAGCAGTTGCCCATGTGCTTGAGAGTCAACCGAATACTTTTGATGCACTGATTGCGCCAGGCCACGTTGCGACAATTATGGGTAGTGAAGAATGGCAGTTTGCAATCGACCATCATAATTTGCCTGTCAGTA
TTGCTGGTTTTCATCCAGAAAGTTTGCTGTTGTCGCTCCAAACGCTGCTCGGTAATTGTTCAAATAAAGTCGTCACACTGAGTAACCGCTATCCCGAAGTGGTAAAGCAAAACGGCAATGCAGCAGCTAAAGCAATAATCAATAAAGCGTTCACTATCGTTGACGCACAT
TGGCGAGGAATTGGTGTTATTCCTGGTTCTGGATTTTCCTTTGCTTCGAGACTATCGCACCTAGACGCCACAAACGATTATGCGCCAGTCGATTTCCCTTCGCAATGTGCGCAAAACGTTCCTGAAACCACATCGCCCTGTGAAAAAGTGATCCTCGGAAAAATGGCACC
TGATGCGTGCCCGTTTTTTGGTCAGGAATGCAAACCAGCATCCCCTAAAGGCGCATGTATGGTGTCTGATGAGGGGGCTTGCCGAATTTGGTATAGCTCTGGTGAGCGCTCGATAACAAATGTTATCAAAAAGGGGAATACGCTAAAGGTGGAGATGAAGTGATGACAAC
TGCCCGCACATATGTTGCCAATACGAATAAGAGTAAAACTATCGCCTATGAGTTGAACATTACTGGAGTCATCCAGGGGGTTGGGTTCAGACCGTTAGTTTACTGCTTAGCGTATGAAAAAAGTATTGTTGGTTGGGTACAAAACGACTGTGGATGTGTGCGAATACACG
CTGAAGGCACTGAGCTAGACGTTGAACAATTTGTATTCGACTTACTGAACAATGGGTCGCTAGTTTCCATATCCTTGCTGGAAAAAAAGCAGGTTAAACTGAGTAATGTAGACTCGTTCACGATAAAAGAAAGCGGTCATGATATAGCGTGCGGGGCGATATCAATCCCC
AAAGATGTCTATTTGTGTGGAGCGTGTAAAGCTGAGTTACTGTCAAATACTAACCGACGTGGCACCTATAGCTTTATCGCTTGCAGTAAATGTGGTCCACGATTTTCTATGCTGCGAGCAATGCCATACGACAGAAAGAACATGAGTATGTCAGCATTTCCAATGTGTGA
GTTATGTCGTAAAGAATATCAATCGCCAACTGATAGGCGCTTTCATGCTCAACCAATAAGCTGTCGCGTATGCGGGCCTGAAGTATTTTGTTCAACTGTCGGCGGGCGCGTGGTCTCGCAAGGCGATGTTGATGTTGTAGCAACGGTTGTCGCGTATTTGAATCAAGGCA
AAATTGTCGCGTTAAAAAGTATAGGCGGTTATCACTTGCTCTGTGACGCACAAAGCACTGAAGCGGTAGACCTTTTACGCCAGCGTAAAAATAGACCAGATAAACCTTTCGCGGTCATGCTCCCAGAGCCTCGTGCAGACGAAACAAGTGAGGATTGGTTAGATAAATGC
GTTGCTGTAGACAGTCATCAAAGAGCGCTCTTACTAAGCCCCGTGCGACCCATTTTACTTGCAAAAAAGCAATCGAATGTACCCATTGCAGATAACGTAGCGCCCATGCTTCGTGATTTGGGGGTGATGTTGCCGTGCAGCGGACTGCATTTACTCATTATGCAGCAATT
CAATCGCCCCATGGTGGCTACGTCAGGAAATATTTACGGTGAACCGATGGTCACATCGACGTCATCGGCACAGCAACACCTTTCGATGTTGGCAGACATCTTCGTGCACCATAATCGAGACATTTTTCACAAACTGGATGATTCTGTGCTGCGAACGCTTAAGTCAGGCA
CCATTCCAATTCGCCTGGGGCGGGGTATAAGTCCGATAGAATTATCATTGCCATATACTGTGCAGGAACCAATGCTTGCTGTTGGCGCGAATCAGAAAAATACAGTCGCAATAGCTTTGAACAATCGACTTATCGTAACACCGCATATTGGCGACCTCGATTCTCCGATA
ATGCAAGCGCACTTTGAAAAATGTATCAGTGACTTTCAAGCTCTCTATCATGTTCATCCACAGCGTATTCTTTGCGATAGCCATCCTGGCTATATCAGTTCCCGTTGGGCCAAAGGCAGTGAACTACCGACTACTAAAGTTTTACATCACCACGCTCATGCATCAGCGTG
GGCATTAGCTGCTGAAATATCGACACCAAGCTTGGTTTTCGTGTGGGATGGAACGGGATTAGGCGAGAATGGCGAGATGTGGGGAGGGGAAGTTTTTTGTGGGACGCCGGGCAATTGGAAACGTGTTGCCTCATTAGCGCCTATGAAGTTGCAAGGCGGCAACAAGGTTC
CAAATCAACCATGGCGAAGTGCAGCATCACTAGTGTGGGGTACTAATTTCACCGGCATTGATTTTAAACCATTTGATCCACAACAATTAAGCTACAACGCATGGCAAAACGAACTCAATTGTCATGTTAGTAGTTCAATGGGAAGACTGTTCGACGCGGCGGCATTTGCC
TGCTGTGAACAGAGCGAAGTCTCTTATGAAGGGCAGGCGCCGATGATGTTGGAAAGCGTTGCGTCGACGCCCTCTAAGGTTATGCCGTTAACGTTGGTCCAGAAAAACGACGAATTTATTCAAATGGATTGGCGAACACTGGTACCGGAACTATTTGATTTCAAAGTGTC
TCCTCAAACTCGTGCTGCAAATTTCCACGCTTCGTTAATTGAAACGGTATGGGAGGTAACCCTACATTGCCGTAAAAAGTTTAATTTTGACCACATTGGATTGGCAGGAGGCGTCTTCCAAAATCGCATTTTGTGTGACGGTATATCACAACGGTTTTTACAATCAGACA
TTCCTGTGGTTATTCCAAGTAGTTTGCCATTAAACGATGCCGCAATAAGTGTTGGGCAAATCCACGAATACTGTTCACGGAGGTCATAATGAAAAGCGCAGAGGGCAATAATCATTCATCATCAGAGCCGTTGGGCACGGAATCTATTCAATTAGCTCAGGGGAACGGCG
GAACACTGACAAAAAGACTCATTGATCATGTTTTTAAAAATAAAGCGAACAAAGATCTAGATCTGCTTCATGATGCCGCCACAGTAACTTTTGATGGTCCTTATCTGAGTATAACAACGGATAGCTTCGTTGTATCCCCCCCAACATTTCCAGGCGGCAATGTAGGCGCG
TTGAGTGTTTACGGTACCGTCAATGATTTAGCAGTCGTTGGGGCAACACCACGGTACATCAGTACTGCATTTATTATTGAAGAAGGCTTTTCCCTTTCAACATTGAAACAAATCGTTAATGGAATGCATCAAGCTGCCGAAGAAACGAAGGTCGCCATTGTAACAGGTGA
TACGAAAGTTGTCCCGAAAGGCAGTGGTGGCGGCGTTTTTATCAATACAACGGGTGTTGGAGACAGCCGAGATGCGCCGATTTGGGATACCAGTCTTATAAAGGCAGGCGATCATGTACTGGTTAGTGGTTCTGTAGGTGATCACGGTGCGTGTGTCCTGCTTGCTCGCG
AGGACTATGGTTTGCAAGGTCAGCTAAAGTCTGATTGTGGAAGCGTTGTTCCTCTTATTGATCCAATCAAGCATTTGGAAGGTGTTCGTTTTGTGCGTGACCCAACTCGTGGAGGGTTGTCAGTATTACTCCATGACGTTGCTAATGAAACAGGCTTCGATATCGAGTTA
ATTGAAAATAATATTCCGGTGCGACCTGAAGTGGCAAGCGTGTGTGAAATATTAGGCTTTGATCCGTTTGTTTTAGCGTGTGAGGGACGCATTGTTGCTGTTGTTGCCCCTGACATAAGCGGTAAGGTGCTAGAACACTGGCGGAGCATTCGAAACGGGGAGCAGGCCGA
ACACATTGGTGTAATAGTAAAAAACACCGAATCAAAAGGGCGCGTGACCATTGTTACGCCAATGGGAGGAAGACGTTTCATGAATGAACTCGAAGATGAGCCGCTTCCAAGGATCTGCTGAGATCCTCTAGAGTCGACCTGCAGGCATGCAAGCTTGGCTGTTTTGGCGG
ATGAGAGAAGATTTTCAGCCTGATACAGATTAAATCAGAACGCAGAAGCGGTCTGATAAAACAGAATTTGCCTGGCGGCAGTAGCGCGGTGGTCCCACCTGACCCCATGCCGAACTCAGAAGTGAAACGCCGTAGCGCCGATGGTAGTGTGGGGTCTCCCCATGCGAGAG
TAGGGAACTGCCAGGCATCAAATAAAACGAAAGGCTCAGTCGAAAGACTGGGCCTTTCGTTTTATCTGTTGTTTGTCGGTGAACGCTCTCCTGAGTAGGACAAATCCGCCGGGAGCGGATTTGAACGTTGCGAAGCAACGGCCCGGAGGGTGGCGGGCAGGACGCCCGCC
ATAAACTGCCAGGCATCAAATTAAGCAGAAGGCCATCCTGACGGATGGCCTTTTTGCGTTTCTACAAACTCTTTTGTTTATTTTTCTAAATACATTCAAATATGTATCCGCTCATGGCGGCCCAGATCCCCGGGTACCGAGCTCGAATTTCGAGCTTCTGGAGCAGGAAG
ATGTCGCGGGCATTAGCACCAGCGGTCTGCCAAGCCTCCGCCAGCCGTTGGGTCCCTTCCGCTTGAGCTTTTCCATCTTCGACGATACGGGCGGCGGCCCCCCGCGCTTCCGCGATCGCCCGTTTACAAGCTGCCTCAGCTGGGGCGATCACATCGGCTTGAAGTTGCTG
CTGCACCTGTTTGATCCGCTCCTGCTGCACAGGGAGTTCTGCTTGGCTACGAGCGACTTCGGTAGCAATGTCCGCTTCAGCTTCGGCCACCACCGCTTCGCGCCGCGTCAACGCATCCTGAATCCGGCGCTCGGCCTCGGCTTGGGCGATCGCTACATCGCGATCGATCC
GACGCAGGGCCGTGATCTTGTCATTTTCGGCCGTTTGGATCGCAGAGGCAGCCTGGGCATCGGCTTCAGCAATTCGGGCATCTCGCTGCAGATCAGCCCGCTGCTTGCGTCCACTAGCCGAGAGATAACCGACCTCATCGGAAATGTTCTGGACTTGCAGCGTATCGAGG
ACTAGACCCAGCTGCTCAAGGTCATCCTCCGCCTCTTCCAGCAGACTTTTGGCAAAGGCAATTTTGTCCTCGTTGATCTGCTCCGGCGTGAGGCTGGCTAAAACACCACGCAAGTTGCCTTCGAGGGTCTCCTTGGCAATTTGCTCGATTTCCTTACGGTTTTTGCCAAG
CAGCCGCTCGATCGCGTTGTGGATGGTCGGTTCTTCCCCAGCAATCTTGATATTGGCAACGCCTTCAACAGTCAGGGGAATGCCGCCCTTGGAGAAGGCATTGGAAACGCGCAACTCAATGATCATGTTGGTCAGATCCATGCGGAGCGCTTTTTCCAGCAGAGGTACCC
GCAGGCTGCTGCCGCCCTTGACCAAGCGATAGCCAACTCGGCGGCCATCACTACTGCGGCGACTACTGCCAGCAAAGATCAAAATTTCACTGGGTTGGCAGATGTAGTAGAGATTGCGCAGGACTAAGCTGCCAGCCCCGGCGACTCACCAGTCACAGAAAAGCATCTTA
CGGATGGCATGACAGTAAGAGAATTATGCAGTGCTGCCATAACCATGAGTGATAACACTGCGGCCAACTTACTTCTGACAACGATCGGAGGACCGAAGGAGCTAACCGCTTTTTTGCACAACATGGGGGATCATGTAACTCGCCTTGATCGTTGGGAACCGGAGCTGAAT
GAAGCCATACCAAACGACGAGCGTGACACCACGATGCCTGCAGCAATGGCAACAACGTTGCGCAAACTATTAACTGGCGAACTACTTACTCTAGCTTCCCGGCAACAATTAATAGACTGGATGGAGGCGGATAAAGTTGCAGGACCACTTCTGCGCTCGGCCCTTCCGGC
TGGCTGGTTTATTGCTGATAAATCTGGAGCCGGTGAGCGTGGGTCTCGCGGTATCATTGCAGCACTGGGGCCAGATGGTAAGCCCTCCCGTATCGTAGTTATCTACACGACGGGGAGTCAGGCAACTATGGATGAACGAAATAGACAGATCGCTGAGATAGGTGCCTCAC
TGATTAAGCATTGGTAACTGTCAGACCAAGTTTACTCATATATACTTTAGATTGATTTAAAACTTCATTTTTAATTTAAAAGGATCTAGGTGAAGATCCTTTTTGATAATCTCATGACCAAAATCCCTTAACGTGAGTTTTCGTTCCACTGAGCGTCAGACCCCGTAGAA
AAGATCAAAGGATCTTC

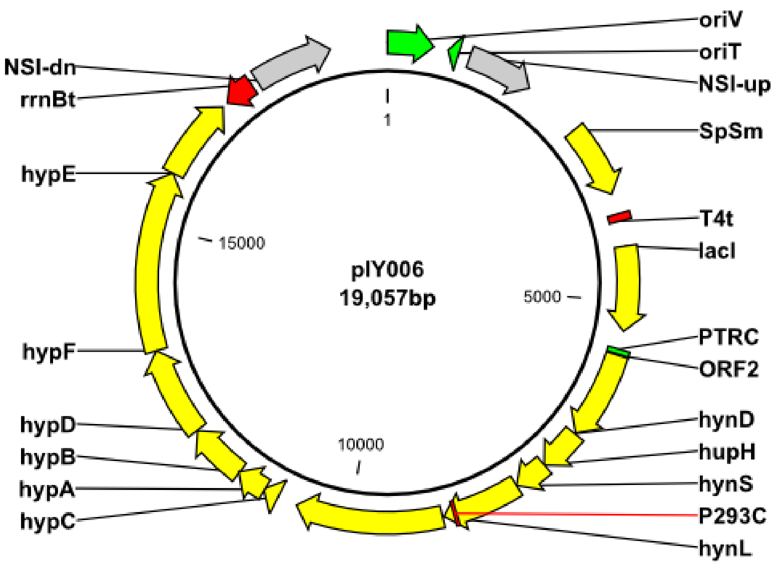

TTGAGATCCTTTTTTTCTGCGCGTAATCTGCTGCTTGCAAACAAAAAAACCACCGCTACCAGCGGTGGTTTGTTTGCCGGATCAAGAGCTACCAACTCTTTTTCCGAAGGTAACTGGCTTCAGCAGAGCGCAGATACCAAATACTGTCCTTCTAGTGTAGCCGTAGTTAG
GCCACCACTTCAAGAACTCTGTAGCACCGCCTACATACCTCGCTCTGCTAATCCTGTTACCAGTGGCTGCTGCCAGTGGCGATAAGTCGTGTCTTACCGGGTTGGACTCAAGACGATAGTTACCGGATAAGGCGCAGCGGTCGGGCTGAACGGGGGGTTCGTGCACACAG
CCCAGCTTGGAGCGAACGACCTACACCGAACTGAGATACCTACAGCGTGAGCTATGAGAAAGCGCCACGCTTCCCGAAGGGAGAAAGGCGGACAGGTATCCGGTAAGCGGCAGGGTCGGAACAGGAGAGCGCACGAGGGAGCTTCCAGGGGGAAACGCCTGGTATCTTTA
TAGTCCTGTCGGGTTTCGCCACCTCTGACTTGAGCGTCGATTTTTGTGATGCTCGTCAGGGGGGCGGAGCCTATGGAAAAACGCCAGCAACGCGGCCTTTTTACGGTTCCTGGCCTTTTGCTGGCCTTTTGCTCACATGTTCTTTCCTGCGTTATCCCCTGATTCTGTGG
ATAACCGTATTACCGCCTTTGAGTGAGCTGATACCGCTCGCCGCAGCCGAACGACCGAGCGCAGCGAGTCAGTGAGCGAGGAAGCGGAAGAGCGCCTGATGCGGTATTTTCTCCTTACGCATCTGTGCGGTATTTCACACCGCATATGGTGCACTCTCAGTACAATCTGC
TCTGATGCCGCATAGTTAAGCCAGTATACACTCCGCTATCGCTACGTGACTGGGTCATGGCTGCGCCCCGACACCCGCCAACACCCGCTGACGCGCCCTGACGGGCTTGTCTGCTCCCGGCATCCGCTTACAGACAAGCTGTGACCGTCTCCGGGAGCTGCATGTGTCAG
AGGTTTTCACCGTCATCACCGAAACGCGCGAGGCAGGATATCCGGCAGCCGGCGGAGCGCTGCTTTCTTGGCAAGCGGTCGCCAGCCCCAACGCCAGGGCTGCCAGCCCGAAACAGCGGGGCAAGGCAGCTTGGAAGGGCGATCGCAGCACGGGCATGGCAATGTCTCTC
TGAAGGAATGCAGACCTTATTCGTACAGCCAGGGTTGAATCGTGGGGGTCCAATCACTTAGCTCTGCTGGGCTAAACCAGAGAGCAATTTCCTGTTGTGCTGTTTCGATTGCATCCGAGCCATGGATGATGTTGCGGCCAATATTGACACCAAAATCACCACGGATGGTG
CCCGGTTCTGCCGTCAGCGGATTGGTAGCGCCGATCAACTTGCGAGCAGCCGCCACAACGCCTTCGCCTTCCAAGACGATCGCCACGATCGGCCCAGAGGTGATGAACTCGACGAGGCCATTGAAGAAGGGGCGCTCGCGGTGGACAGCATAGTGCTGTTCGGCCAGCTC
GCGACTGGGCTTCAGCTGCTTTAGGCCCACCAGTTTGAAGCCTTTTTGCTCAAAGCGGCCGATGATCGTACCGACCAAACCCCGCTGAACGCCATCGGGCTTGATGGCAATAAATGTGCGTTCCACAGACATCTAGATAGTCCTCAAGACGAGGCAAGCATTGAGCTTGC
CTTCCTATGGTTCGGGATCACTGGGATTCTTGACAAGCGATCGCGGTCACATCGCTATCTCTTAGGACTTCGCAGCGGGCGAGTCGGATTGACCCGGTAGGGATTTCGCCAGATCAATGCCCGTGGTTTGTTTCAGCTTCTCCAGCAAGCTAGCGATTTGGGTAGCGCTG
CCTTCCCCTTCGCCAATCACAGTGATCGACTCCACGTCGATATCTGGCACGGTGCCTGAAAGCGTGACGAGCAGGGACTCGAAGCTTGCATGCCTGCAGGTCGACTCTAGAGCTTTATGCTTGTAAACCGTTTTGTGAAAAAATTTTTAAAATAAAAAAGGGGACCTCTA
GGGTCCCCAATTAATTAGTAATATAATCTATTAAAGGTCATTCAAAAGGTCATCCACCGGATCAATTCCCCTGCTCGCGCAGGCTGGGTGCCAAGCTCTCGGGTAACATCAAGGCCCGATCCTTGGAGCCCTTGCCCTCCCGCACGATGATCGTGCCGTGATCGAAATCC
AGATCCTTGACCCGCAGTTGCAAACCCTCACTGATCCGCATGCCCGTTCCATACAGAAGCTGGGCGAACAAACGATGCTCGCCTTCCAGAAAACCGAGGATGCGAACCACTTCATCCGGGGTCAGCACCACCGGCAAGCGCCGCGACGGCCGAGGTCTTCCGATCTCCTG
AAGCCAGGGCAGATCCGTGCACAGCACCTTGCCGTAGAAGAACAGCAAGGCCGCCAATGCCTGACGATGCGTGGAGACCGAAACCTTGCGCTCGTTCGCCAGCCAGGACAGAAATGCCTCGACTTCGCTGCTGCCCAAGGTTGCCGGGTGACGCACACCGTGGAAACGGA
TGAAGGCACGAACCCAGTGGACATAAGCCTGTTCGGTTCGTAAGCTGTAATGCAAGTAGCGTATGCGCTCACGCAACTGGTCCAGAACCTTGACCGAACGCAGCGGTGGTAACGGCGCAGTGGCGGTTTTCATGGCTTGTTATGACTGTTTTTTTGGGGTACAGTCTATG
CCTCGGGCATCCAAGCAGCAAGCGCGTTACGCCGTGGGTCGATGTTTGATGTTATGGAGCAGCAACGATGTTACGCAGCAGGGCAGTCGCCCTAAAACAAAGTTAAACATCATGAGGGAAGCGGTGATCGCCGAAGTATCGACTCAACTATCAGAGGTAGTTGGCGTCAT
CGAGCGCCATCTCGAACCGACGTTGCTGGCCGTACATTTGTACGGCTCCGCAGTGGATGGCGGCCTGAAGCCACACAGTGATATTGATTTGCTGGTTACGGTGACCGTAAGGCTTGATGAAACAACGCGGCGAGCTTTGATCAACGACTTTTGGAAACTTCGGCTTCCCC
TGGAGAGAGCGAGATTCTCCGCGCTGTAGAAGTCACCATTGTTGTGCACGACGACATCATTCCGTGGCGTTATCCAGCTAAGCGCGAACTGCAATTTGGAGAATGGCAGCGCAATGACATTCTTGCAGGTATCTTCGAGCCAGCCACGATCGACATTGATCTGGCTATCT
TGCTGACAAAAGCAAGAGAACATAGCGTTGCCTTGGTAGGTCCAGCGGCGGAGGAACTCTTTGATCCGGTTCCTGAACAGGATCTATTTGAGGCGCTAAATGAAACCTTAACGCTATGGAACTCGCCGCCCGACTGGGCTGGCGATGAGCGAAATGTAGTGCTTACGTTG
TCCCGCATTTGGTACAGCGCAGTAACCGGCAAAATCGCGCCGAAGGATGTCGCTGCCGACTGGGCAATGGAGCGCCTGCCGGCCCAGTATCAGCCCGTCATACTTGAAGCTAGACAGGCTTATCTTGGACAAGAAGAAGATCGCTTGGCCTCGCGCGCAGATCAGTTGGA
AGAATTTGTCCACTACGTGAAAGGCGAGATCACCAAGGTAGTCGGCAAATAATGTCTAACAATTCGTTCAAGCCGACGCCGCTTCGCGGCGCGGCTTAACTCAAGCGTTAGATGCACTAAGCACATAATTGCTCACAGCCAAACTATCAGGTCAAGTCTGCTTTTATTAT
TTTTAAGCGTGCATAATAAGCCCTACACAAATTGGGAGATATATCATGAAAGGCTGGCTTTTTCTTGTTATCGCAATAGTTGGCGAAGTAATCGCAACATCCGCATTAAAATCTAGCGAGGGCTTTACTAAGCTGATCCGGTGGATGACCTTTTGAATGACCTTTAATAG
ATTATATTACTAATTAATTGGGGACCCTAGAGGTCCCCTTTTTTATTTTAAAAATTTTTTCACAAAACGGTTTACAAGCATACTAGAGGATCGGCGGCCGCGGATCTGGGCCGCGGGTCATGGCTGCGCCCCGACACCCGCCAACACCCGCTGACGCGCCCTGACGGGCT
TGTCTGCTCCCGGCATCCGCTTACAGACAAGCTGTGACCGTCTCCGGGAGCTGCATGTGTCAGAGGTTTTCACCGTCATCACCGAAACGCGCGAGGCAGCAGATCAATTCGCGCGCGAAGGCGAAGCGGCATGCATTTACGTTGACACCATCGAATGGTGCAAAACCTTT
CGCGGTATGGCATGATAGCGCCCGGAAGAGAGTCAATTCAGGGTGGTGAATGTGAAACCAGTAACGTTATACGATGTCGCAGAGTATGCCGGTGTCTCTTATCAGACCGTTTCCCGCGTGGTGAACCAGGCCAGCCACGTTTCTGCGAAAACGCGGGAAAAAGTGGAAGC
GGCGATGGCGGAGCTGAATTACATTCCCAACCGCGTGGCACAACAACTGGCGGGCAAACAGTCGTTGCTGATTGGCGTTGCCACCTCCAGTCTGGCCCTGCACGCGCCGTCGCAAATTGTCGCGGCGATTAAATCTCGCGCCGATCAACTGGGTGCCAGCGTGGTGGTGT
CGATGGTAGAACGAAGCGGCGTCGAAGCCTGTAAAGCGGCGGTGCACAATCTTCTCGCGCAACGCGTCAGTGGGCTGATCATTAACTATCCGCTGGATGACCAGGATGCCATTGCTGTGGAAGCTGCCTGCACTAATGTTCCGGCGTTATTTCTTGATGTCTCTGACCAG
ACACCCATCAACAGTATTATTTTCTCCCATGAAGACGGTACGCGACTGGGCGTGGAGCATCTGGTCGCATTGGGTCACCAGCAAATCGCGCTGTTAGCGGGCCCATTAAGTTCTGTCTCGGCGCGTCTGCGTCTGGCTGGCTGGCATAAATATCTCACTCGCAATCAAAT
TCAGCCGATAGCGGAACGGGAAGGCGACTGGAGTGCCATGTCCGGTTTTCAACAAACCATGCAAATGCTGAATGAGGGCATCGTTCCCACTGCGATGCTGGTTGCCAACGATCAGATGGCGCTGGGCGCAATGCGCGCCATTACCGAGTCCGGGCTGCGCGTTGGTGCGG
ATATCTCGGTAGTGGGATACGACGATACCGAAGACAGCTCATGTTATATCCCGCCGTTAACCACCATCAAACAGGATTTTCGCCTGCTGGGGCAAACCAGCGTGGACCGCTTGCTGCAACTCTCTCAGGGCCAGGCGGTGAAGGGCAATCAGCTGTTGCCCGTCTCACTG
GTGAAAAGAAAAACCACCCTGGCGCCCAATACGCAAACCGCCTCTCCCCGCGCGTTGGCCGATTCATTAATGCAGCTGGCACGACAGGTTTCCCGACTGGAAAGCGGGCAGTGAGCGCAACGCAATTAATGTAAGTTAGCGCGAATTGATCTGGTTTGACAGCTTATCAT
CGACTGCACGGTGCACCAATGCTTCTGGCGTCAGGCAGCCATCGGAAGCTGTGGTATGGCTGTGCAGGTCGTAAATCACTGCATAATTCGTGTCGCTCAAGGCGCACTCCCGTTCTGGATAATGTTTTTTGCGCCGACATCATAACGGTTCTGGCAAATATTCTGAAATG
AGCTGTTGACAATTAATCATCCGGCTCGTATAATGTGTGGAATTGTGAGCGGATAACAATTTCACACAGGAAACAGACCATGGATGGCTTCCATTATTGGTAGCGGCTTTTTGATCATTGCACCACTGCTGCATTCGGTAATGGGGAAATGGGCTCTGCTTGGAATAGTG
TTGTTGTCCATTCTGGCATATGCGCTGGGGTCGGTTATTCGCTTCAATATACGCCATGCAGAACCTGAGCTAGCTAGCAATCAACAAAGCAGTATTGTGACGCTTGAAAAAGCAAGTCAATGGGCGCTTGGTGCGGCATATGCCATTTCCGTGGCGTTTTATATTAGTTT
GTTTGCAGCGTTTGTTTTTGATCGTTTATCAATCTCTGATACGACGTACATCAAACTGTTTACCAGTGGGTTGCTTGTCATCATTACGGTGGTCGCTTGGCTCCGTGGTGCTAGGGGGCTAGAGACCATTGAGCTATTCGCCGTGACAATAAAGTTGGCTATTATAGTAG
GTGTGCTCGCAGCGCTGGCAACCTACGACATACAAGTACAAAGTGCTTGGTTTCAACATGAAGCGATACAGGCATTGAGCCATTTTGAAACTGTTAGCATGCTCGCAGGCATGCTCATGGTAACGCAAGGGTTCGAGACAACCCGTTTTATGGGGAATAACTACACGCCA
GAACAGCGCATTAAAGCGAGTCGTTATGCTCAGTGGATCGCTATTTTTCTCTATGTTGTTTTTATCGGTCTAACGTGTCCTATTTTTCTGGATTTTCCGATCACGGAATTAAACGAAACAACGATTAGCTACACCCTAGGGCAAGCAATATGGGTATTGCCGATTTTATT
ACTGGTTGCCGCCACGGCAAGTCAATTAAGTGCAGCATTAGCCGATACTATCGGTGGCGGAGGCCTCTTAAAAGAGCTTTTCCATTTACCTATCTCCCCGCAATTTTACTACGTTTTAGTCATCGCCATCGCAGGTATATTAGTTTGGTCATCCAACGTATTTGAAATTA
TTAATCTCGCTTCAAAAGGGTTTGCACTTTATTACCTGATCCAAACAATAATCGCGGTCAAACTCGTGATGCGTCAAAGCACAGGTCAACGCATTAGGTCAACAAAATTACTTGGTCTATCGGTGATCGTTCTCTGTTTATTCTTTGTGATTGGCTGGTCAATTCCAGCA
CCTCATAGCTAATAAGGAAAATACCGATGCTATCAGTAGAAACTACACCACCAATTGACCAAATCAAAAACGCGGCTAAGCGCTACGCTATCATTGGTGTTGGAAATCTATTGCAGCGAGATGATGGCGTTGGTGTGCACGCGGTTCGTGCTATGCAACCCCTGCTAAAG
TCTTATTCAAATGTATCTTGCATTGACGCTGGGACACTGAGCTATGAACTTTTGGAGTGGGTTGCGAGTTCCGATCATACGATAGTCATCGACGCGGCTTATATGCGCTGTCCGCCTGGCACGGTGAGAGTGTTTGAGAACGAGGCAATTGCGGATCAGTTTCAGCAAGC
GACGACCCATTCCGTTCACCAGATCACGTTACGCGACACATTGTTGACCAGCCAAACGTTATATTCAAAACCTTCTGCGGTGACGTTAATTGGCGTAGAGCCGGAGGCGATTGAGTGGGGAACCTCTTTGAGTCCAAAGGTAAATGCAGCGCTATCATCGATCATGGATA
TCGCATTTGCTTGCTTATCAGGCGACAACGTTCAGACCTATAAACCGGCAACCCAAAAGGAGGCACGATGACCGAAGAACTCATTTTTATGACATCAAATGCTGATAAAACGGGCAACGTAATGCCGCTTTTGCATCAGATACGTCATGCGTTGTCTCAGTTAATTGAAC
GACAAGAACAAACCACGATTGATCTGCGACGATTGCCTTTAAGCGCTAGCGAAGAAGCGCAGTTAGAAGCTTTTCTTGGGCATGGAGAGGTTAAAGCTGATATTCAGGCGCTGGGTGATACGGTGCTAATCGAATCACGCTACGCAGGCGTTTGGCTTGAAATTCACTAT
AACGAAGATGTGGAGATCATGGGCAAATACGTTCATATCTGTACCTGTCCACCAATCATAAAATCTCAGCCAGAAGATATGGTGTTATCGCTCAGCAATATAGTGTCTGACATCCATTCGTTGTCTCATCAATCTTCTGATGAAACGGCTAAGGAGGATTGAAATGGCGC
TACCAACATTAAACAAGCAGTTACAAGCGTCCGGTATCTCAAGACGCACATTTCTTAAGTTTTGCGCTACAACGGCGTCTTTACTGGCATTGCCGCAAAGCGCTGTTGCCGATTTGGCGACTGCCCTTGGCAATGCGAGAAGACCCTCTGTGATTTGGTTGCCCTTTCAA
GAGTGCACAGGGTGTACCGAAGCAATATTGCGCTCTCATGCTCCCACATTGGAAAGCCTTATTTTCGATCATATTTCGTTGGATTATCAGCATACGATAATGGCTGCTGCGGGAGAGCAAGCTGAAGACGCTAGGCGTGCGGCGATGAACGCGCACAAAGGGCAATATTT
GTTGTTGGTTGATGGTTCGGTTCCGGTGGGTAACCCAGGATACTCAACGATCAGTGGCATGAGTAATGTCGATATGCTGAGAGAATCGGCAAAAGATGCTGCGGGTATTATTGCCATCGGTACCTGCGCGTCTTTTGGCGGGATCCCTAAAGCAAACCCAAATCCGACGG
GGGCAGTGGCAGTAAGCGACATTATTACAGACAAGCCAATCGTAAACATTTCAGGCTGTCCGCCACTGCCTATCGCGATTACAGCTGTGTTGGTTCATTACCTGACGTTTAAGCGTTTCCCTGATCTCGACGAATTACAACGCCCACTCGCTTTTTTTGGTGAAAGCATC
CATGACAGATGTTATCGACGCCCATTTTTTGAACAACGTAAATTTGCAAAATCGTTTGATGATGAGGGGGCAAAAAATGGGTGGTGTTTGTTCGAACTTGGTTGTAAAGGGCCCGAAACCTTTAACGCATGTGCAACGGTTAAATGGAATCAAGGCACGAGTTTTTGTAT
CGAATCTGGCCATCCGTGTCTTGGTTGCTCTGAGCCCGATTTCTGGGATAAAAGCAGCTTTTACCAAGCCTTGGGTCCATGGGAGTGGTACAAATCCAAACCCGGCAAAGGTGCACAGAAGCATGCTGGGAAAAACTCATAAGATAATAGGCAAGGCTCATGGAAAATAC
AGCAAGTAACAACCGGTTAGTCGTCGATCCTATCACTCGAATTGAAGGGCATCTTCGAATAGAAGCTGAAATGGATGGGAATACCATCAAACAGGCGTTCTCATCAGGCACGTCTGTTCGGGGAATTGAACTGATTTTACAAGGCAGAGATCCGCGTGACGCTTGGGCTT
TTGCGCAACGTATCTGTGGCGTCTGCACGCTGGTGCATGGTATGGCATCGGTGCGTGCTGTCGAAGATGCAATTAGAAAAGCTTGGCGGTCAAACGCAAAATTAGGGGTTGCCATTGGAAAGCCCTCCATGACATCTATGCCAAAAGGACCGATGCAACATGGTAAAAAA
GGGCACCGACAGTCACGTACTTCAATAGGCGTACTGAGTGAAGCAGAAATGGCTATCCCTCAAAATGCACAACTGATAAGAAACATCATGATTGCAACCCAATATGTGCATGATCACGTGATGCATTTTTATCACTTGCATGCCCTAGATTGGGTTGACGTTGTTTCTGC
ACTAGATGCAGATCCAACGAGAACCGCTACGCTCGCCGGTCAATTGAGTGATTATCCTCGTTCATCGCCGGGATATTTCAAAGATGTGAAGCAAAAAGTCAAAACGCTGGTTGAGTCTGGGCAGCTAGGGATATTCAGTAACGCGTATTGGGGGCATCCTGGCTATAAAC
TGCCACCTGAAGTTAACTTGATGGCATTAGCACATTATTTAGATGCGCTAACGTGGCAGCGTGAAGTTGTAAAAGTTCACACCATATTTGGAGGGAAAAACCCTCATCCTAATTTTGTCGTTGGCGGCGTGCCTTCACCGATTAATCTCAATGCGTCAACGGGTATTAAC
ACGAGTCGATTAGTGCAACTACAAGATGCTATCACGCAAATGAAGAGCTTTGTCGATCAGGTGTATTACCCCGATATTGTGGCGATTGCGGGTTATTACAAAGAGTGGGGGACACGAGGGGAAGGGCTGGGTAACTTTCTTACCTATGGAGACTTACCTATGACATCAAT
GGATGACCCTGATTCTTTCTTGTTTCCACGAGGTGCAATACTTGGTCGAGACTTGAGTAAAGTGCATGACCTTGATCTAGATGATCCCTCTGAAATTCAAGAATTCGTCTCTTCCTCCTGGTATCGATATAGTGGAGGGAACGCAAGTGGTTTACACCCTTTTAATGGAC
AAACAACACTCGAGTATACTGGTCCGAAACCGCCTTACAAGCACCTAAATGTAGGGGCTGAATATTCATGGTTGAAAAGTCCGCGTTGGAAAGGCCATGCGATGGAGGTGGGACCGCTGGCTCGCGTGCTAATGATGTATGCTAAAAAAGATGCCGCTGCGCAAGACATC
GTTAATCGATCTCTTTCTATCTTGGATTTAGAGACCTCTGCACTTTTCTCCACACTCGGTAGGACGCTCGCCAGAGCAGTGGAAACAAAAATTGTGGTTAACCAGCTACAGTCTTGGTATGACCAACTATTGGATAATATCGCAAAGGGCGATACCGATACGTTTAACCC
TCTGTATTTTGACCCTACCAATTGGCCAATTAAAGGCCAGGGGGTAGGCGTGATGGAAGCGCCTCGTGGCGCGTTGGGGCATTGGTTGGTCATGCAAAATGGCAAAATTGAGAATTACCAATGTGTCGTGCCTACGACATGGAACGCTGGACCTCGAGATCCCAACTCAC
AGGCAGGTGCTTATGAAGCCGCTCTGCAAGATAAACATACGCTACATGATCCTGACCAACCTTTAGAGATTTTGCGAACACTTCATAGTTTTGACCCCTGCTTAGCATGTGCCGTGCACGTAATGGACGAAACAGGGGAAGAGCGTTTGCGTCTAAAAGTTCGTTAACCT
TACTTGAGGAATATTATGAAAATCAAAAATAGTATTAGAAAGTCAGTTACTTCTTTAATTGCCTTTTTTTACCTGGTGGTAGGTTCACCCGTTAGTTATGCGCACCCGGGTCATCAAGGCGATCATTCACTACTTTTGGGATCGCTGGTGTCAACAATCGTGCTTGTCAC
CGTTGCGCTGTTTATTTATCGAGCTAGAGCAAGTAGGCCCTATCAACGGGTAAAACAGTAATGTGTCTCGCTATCCCCATGAAAGTCATTGCAATTAAAGGCTTTAACGCAACGTGTGAAGCGAAAGGTGTCTCTCGCGAGGTGAGTTTACATTTAGTGCAAGGGCTAGA

GGTCAAAGTTGGCGACTATGTGATGGTACATGTTGGTTATGCTCTCCAAGTGATCACCTATGATGAAGCGCAGGTGACATGGGAGATGCTCGATCAGGTCATTGCTTACGATGCATGAAATTAGTCTTTGCTATAGCTTGCTTGACACAGTCGCTGTTCACCAGCGAGCA
AACATGAATAAGTCTGTCAGTCTGGTTCACGTCAAAGTCGGCCCACTGTCAGGTGTTGAGCCTGACTTGCTGCACCATGCGTTCTTGGCGTGTAGAACTCACACCATTGGCGATCAGGCAACGCTTAGAATTGACACAAGCGCTATCAAGATACGCTGTAAATTGTGTGG
CGAATTCAGCCGAGTTTCGGTGAATAGCATCCTATGTGCAAGTTGCGGTGCTTGGCAAACCGACCTCATTGAGGGCGATGAATTTATATTGCAACGAATAGAGTTTATCGCACCTGACAATTTTACAGAATCAAAGGAGAGAAAACATGTGCGGTAACTGTGGTTGCGAA
ACGCAAGTAAAATCAGAGAAAACTGACAAGCCATCAGCAAATATCGTTGAGGTTCACGCAAATTTAAAAAGCAACAATGACAACCAAGCGATAGCAAACCGTGCTTTATTTGACGCACATAATGTACTAGTGATTAATTTGATGTCATCCCCAGGCAGTGGAAAAACCAG
GCTACTCGAAGAAACGATACGTGCGCTTAAGTCGCAATACGCGATGGCGGTGATTGAAGGCGACCTCGAAACTGAAAATGACGCAAATAGAATTAGACGTCATGGTGTGCAGGCGGAGCAAATCGCCACGGGCCAAGGGTGTCACTTGGACGCCAGTATGGTGCAAAAGG
TACTTGGAAGATTTAGCTTACAAAAACTAGACGTTTTGTTTATAGAGAATGTTGGCAATTTGATTTGTCCAGCGTGTTTTGATTTAGGACAGCACTTAAACATTATTTTGCTGTCCGTACCAGAAGGTGATGATAAGCCTGAAAAGTACCCAGTGATGTTTCGTGCCGCG
GACGTCATGCTTATTTCTAAGACCGATTACTTGCAATTTCACGATGAATTCAATGTACCTCGCGCGATTGAATCATTTCGTAAAGTGGGCAACGATGCGCCCGTTCTCGAAGTGTCTTCATTGAAAAGTCAACATCTAGAGCGTTGGTTTTCTTATTTTACGCACGCCAT
TACAGCTCATCAGTCTCAGTCGTTACAAAATAGCGGAGTCTAAAGTCTTATGTACAGTGTTGAATCGTTGCTAAGAGACATTAAGAATACCTCGCTTTCAAAACCGTTTCGTATTCTTAATGTTTGCGGTGGCCATGAGCGTGCGATTACGCGCGCTGGATTTAGAACGT
TGTTTCAGCACAATATTCATCTGATACCTGGGCCAGGGTGCCCCGTTTGTATTTGCCCCGAAGAGGATATTGCATACGCGATACACTTAGCCATTAACGAAAATGTCGTTATCGTTAGTTTTGGTGACATGCTCCGCGTCCCAGTCGAACACGAAATGGGTGGCTGTAGC
TCATTGATAGACGCAAAAAATCAAGGGGCCGATATCCGTCCGATATCTTCTCCTCAAGAAGCGGTATCTATAGCATTGGAAGAACCTCGTAAGGTTATTGTGTTTTTTGCTGTTGGTTTTGAAACGACTATGGCACCGATTGCAGCAACACTGCTTAATGATTTACCCAA
TAACTTTAAAGTATTATTGTCTGGACGACTTACTTGGCCAGCAGTTGCCCATGTGCTTGAGAGTCAACCGAATACTTTTGATGCACTGATTGCGCCAGGCCACGTTGCGACAATTATGGGTAGTGAAGAATGGCAGTTTGCAATCGACCATCATAATTTGCCTGTCAGTA
TTGCTGGTTTTCATCCAGAAAGTTTGCTGTTGTCGCTCCAAACGCTGCTCGGTAATTGTTCAAATAAAGTCGTCACACTGAGTAACCGCTATCCCGAAGTGGTAAAGCAAAACGGCAATGCAGCAGCTAAAGCAATAATCAATAAAGCGTTCACTATCGTTGACGCACAT
TGGCGAGGAATTGGTGTTATTCCTGGTTCTGGATTTTCCTTTGCTTCGAGACTATCGCACCTAGACGCCACAAACGATTATGCGCCAGTCGATTTCCCTTCGCAATGTGCGCAAAACGTTCCTGAAACCACATCGCCCTGTGAAAAAGTGATCCTCGGAAAAATGGCACC
TGATGCGTGCCCGTTTTTTGGTCAGGAATGCAAACCAGCATCCCCTAAAGGCGCATGTATGGTGTCTGATGAGGGGGCTTGCCGAATTTGGTATAGCTCTGGTGAGCGCTCGATAACAAATGTTATCAAAAAGGGGAATACGCTAAAGGTGGAGATGAAGTGATGACAAC
TGCCCGCACATATGTTGCCAATACGAATAAGAGTAAAACTATCGCCTATGAGTTGAACATTACTGGAGTCATCCAGGGGGTTGGGTTCAGACCGTTAGTTTACTGCTTAGCGTATGAAAAAAGTATTGTTGGTTGGGTACAAAACGACTGTGGATGTGTGCGAATACACG
CTGAAGGCACTGAGCTAGACGTTGAACAATTTGTATTCGACTTACTGAACAATGGGTCGCTAGTTTCCATATCCTTGCTGGAAAAAAAGCAGGTTAAACTGAGTAATGTAGACTCGTTCACGATAAAAGAAAGCGGTCATGATATAGCGTGCGGGGCGATATCAATCCCC
AAAGATGTCTATTTGTGTGGAGCGTGTAAAGCTGAGTTACTGTCAAATACTAACCGACGTGGCACCTATAGCTTTATCGCTTGCAGTAAATGTGGTCCACGATTTTCTATGCTGCGAGCAATGCCATACGACAGAAAGAACATGAGTATGTCAGCATTTCCAATGTGTGA
GTTATGTCGTAAAGAATATCAATCGCCAACTGATAGGCGCTTTCATGCTCAACCAATAAGCTGTCGCGTATGCGGGCCTGAAGTATTTTGTTCAACTGTCGGCGGGCGCGTGGTCTCGCAAGGCGATGTTGATGTTGTAGCAACGGTTGTCGCGTATTTGAATCAAGGCA
AAATTGTCGCGTTAAAAAGTATAGGCGGTTATCACTTGCTCTGTGACGCACAAAGCACTGAAGCGGTAGACCTTTTACGCCAGCGTAAAAATAGACCAGATAAACCTTTCGCGGTCATGCTCCCAGAGCCTCGTGCAGACGAAACAAGTGAGGATTGGTTAGATAAATGC
GTTGCTGTAGACAGTCATCAAAGAGCGCTCTTACTAAGCCCCGTGCGACCCATTTTACTTGCAAAAAAGCAATCGAATGTACCCATTGCAGATAACGTAGCGCCCATGCTTCGTGATTTGGGGGTGATGTTGCCGTGCAGCGGACTGCATTTACTCATTATGCAGCAATT
CAATCGCCCCATGGTGGCTACGTCAGGAAATATTTACGGTGAACCGATGGTCACATCGACGTCATCGGCACAGCAACACCTTTCGATGTTGGCAGACATCTTCGTGCACCATAATCGAGACATTTTTCACAAACTGGATGATTCTGTGCTGCGAACGCTTAAGTCAGGCA
CCATTCCAATTCGCCTGGGGCGGGGTATAAGTCCGATAGAATTATCATTGCCATATACTGTGCAGGAACCAATGCTTGCTGTTGGCGCGAATCAGAAAAATACAGTCGCAATAGCTTTGAACAATCGACTTATCGTAACACCGCATATTGGCGACCTCGATTCTCCGATA
ATGCAAGCGCACTTTGAAAAATGTATCAGTGACTTTCAAGCTCTCTATCATGTTCATCCACAGCGTATTCTTTGCGATAGCCATCCTGGCTATATCAGTTCCCGTTGGGCCAAAGGCAGTGAACTACCGACTACTAAAGTTTTACATCACCACGCTCATGCATCAGCGTG
GGCATTAGCTGCTGAAATATCGACACCAAGCTTGGTTTTCGTGTGGGATGGAACGGGATTAGGCGAGAATGGCGAGATGTGGGGAGGGGAAGTTTTTTGTGGGACGCCGGGCAATTGGAAACGTGTTGCCTCATTAGCGCCTATGAAGTTGCAAGGCGGCAACAAGGTTC
CAAATCAACCATGGCGAAGTGCAGCATCACTAGTGTGGGGTACTAATTTCACCGGCATTGATTTTAAACCATTTGATCCACAACAATTAAGCTACAACGCATGGCAAAACGAACTCAATTGTCATGTTAGTAGTTCAATGGGAAGACTGTTCGACGCGGCGGCATTTGCC
TGCTGTGAACAGAGCGAAGTCTCTTATGAAGGGCAGGCGCCGATGATGTTGGAAAGCGTTGCGTCGACGCCCTCTAAGGTTATGCCGTTAACGTTGGTCCAGAAAAACGACGAATTTATTCAAATGGATTGGCGAACACTGGTACCGGAACTATTTGATTTCAAAGTGTC
TCCTCAAACTCGTGCTGCAAATTTCCACGCTTCGTTAATTGAAACGGTATGGGAGGTAACCCTACATTGCCGTAAAAAGTTTAATTTTGACCACATTGGATTGGCAGGAGGCGTCTTCCAAAATCGCATTTTGTGTGACGGTATATCACAACGGTTTTTACAATCAGACA
TTCCTGTGGTTATTCCAAGTAGTTTGCCATTAAACGATGCCGCAATAAGTGTTGGGCAAATCCACGAATACTGTTCACGGAGGTCATAATGAAAAGCGCAGAGGGCAATAATCATTCATCATCAGAGCCGTTGGGCACGGAATCTATTCAATTAGCTCAGGGGAACGGCG
GAACACTGACAAAAAGACTCATTGATCATGTTTTTAAAAATAAAGCGAACAAAGATCTAGATCTGCTTCATGATGCCGCCACAGTAACTTTTGATGGTCCTTATCTGAGTATAACAACGGATAGCTTCGTTGTATCCCCCCCAACATTTCCAGGCGGCAATGTAGGCGCG
TTGAGTGTTTACGGTACCGTCAATGATTTAGCAGTCGTTGGGGCAACACCACGGTACATCAGTACTGCATTTATTATTGAAGAAGGCTTTTCCCTTTCAACATTGAAACAAATCGTTAATGGAATGCATCAAGCTGCCGAAGAAACGAAGGTCGCCATTGTAACAGGTGA
TACGAAAGTTGTCCCGAAAGGCAGTGGTGGCGGCGTTTTTATCAATACAACGGGTGTTGGAGACAGCCGAGATGCGCCGATTTGGGATACCAGTCTTATAAAGGCAGGCGATCATGTACTGGTTAGTGGTTCTGTAGGTGATCACGGTGCGTGTGTCCTGCTTGCTCGCG
AGGACTATGGTTTGCAAGGTCAGCTAAAGTCTGATTGTGGAAGCGTTGTTCCTCTTATTGATCCAATCAAGCATTTGGAAGGTGTTCGTTTTGTGCGTGACCCAACTCGTGGAGGGTTGTCAGTATTACTCCATGACGTTGCTAATGAAACAGGCTTCGATATCGAGTTA
ATTGAAAATAATATTCCGGTGCGACCTGAAGTGGCAAGCGTGTGTGAAATATTAGGCTTTGATCCGTTTGTTTTAGCGTGTGAGGGACGCATTGTTGCTGTTGTTGCCCCTGACATAAGCGGTAAGGTGCTAGAACACTGGCGGAGCATTCGAAACGGGGAGCAGGCCGA
ACACATTGGTGTAATAGTAAAAAACACCGAATCAAAAGGGCGCGTGACCATTGTTACGCCAATGGGAGGAAGACGTTTCATGAATGAACTCGAAGATGAGCCGCTTCCAAGGATCTGCTGAGATCCTCTAGAGTCGACCTGCAGGCATGCAAGCTTGGCTGTTTTGGCGG
ATGAGAGAAGATTTTCAGCCTGATACAGATTAAATCAGAACGCAGAAGCGGTCTGATAAAACAGAATTTGCCTGGCGGCAGTAGCGCGGTGGTCCCACCTGACCCCATGCCGAACTCAGAAGTGAAACGCCGTAGCGCCGATGGTAGTGTGGGGTCTCCCCATGCGAGAG
TAGGGAACTGCCAGGCATCAAATAAAACGAAAGGCTCAGTCGAAAGACTGGGCCTTTCGTTTTATCTGTTGTTTGTCGGTGAACGCTCTCCTGAGTAGGACAAATCCGCCGGGAGCGGATTTGAACGTTGCGAAGCAACGGCCCGGAGGGTGGCGGGCAGGACGCCCGCC
ATAAACTGCCAGGCATCAAATTAAGCAGAAGGCCATCCTGACGGATGGCCTTTTTGCGTTTCTACAAACTCTTTTGTTTATTTTTCTAAATACATTCAAATATGTATCCGCTCATGGCGGCCCAGATCCCCGGGTACCGAGCTCGAATTTCGAGCTTCTGGAGCAGGAAG
ATGTCGCGGGCATTAGCACCAGCGGTCTGCCAAGCCTCCGCCAGCCGTTGGGTCCCTTCCGCTTGAGCTTTTCCATCTTCGACGATACGGGCGGCGGCCCCCCGCGCTTCCGCGATCGCCCGTTTACAAGCTGCCTCAGCTGGGGCGATCACATCGGCTTGAAGTTGCTG
CTGCACCTGTTTGATCCGCTCCTGCTGCACAGGGAGTTCTGCTTGGCTACGAGCGACTTCGGTAGCAATGTCCGCTTCAGCTTCGGCCACCACCGCTTCGCGCCGCGTCAACGCATCCTGAATCCGGCGCTCGGCCTCGGCTTGGGCGATCGCTACATCGCGATCGATCC
GACGCAGGGCCGTGATCTTGTCATTTTCGGCCGTTTGGATCGCAGAGGCAGCCTGGGCATCGGCTTCAGCAATTCGGGCATCTCGCTGCAGATCAGCCCGCTGCTTGCGTCCACTAGCCGAGAGATAACCGACCTCATCGGAAATGTTCTGGACTTGCAGCGTATCGAGG
ACTAGACCCAGCTGCTCAAGGTCATCCTCCGCCTCTTCCAGCAGACTTTTGGCAAAGGCAATTTTGTCCTCGTTGATCTGCTCCGGCGTGAGGCTGGCTAAAACACCACGCAAGTTGCCTTCGAGGGTCTCCTTGGCAATTTGCTCGATTTCCTTACGGTTTTTGCCAAG
CAGCCGCTCGATCGCGTTGTGGATGGTCGGTTCTTCCCCAGCAATCTTGATATTGGCAACGCCTTCAACAGTCAGGGGAATGCCGCCCTTGGAGAAGGCATTGGAAACGCGCAACTCAATGATCATGTTGGTCAGATCCATGCGGAGCGCTTTTTCCAGCAGAGGTACCC
GCAGGCTGCTGCCGCCCTTGACCAAGCGATAGCCAACTCGGCGGCCATCACTACTGCGGCGACTACTGCCAGCAAAGATCAAAATTTCACTGGGTTGGCAGATGTAGTAGAGATTGCGCAGGACTAAGCTGCCAGCCCCGGCGACTCACCAGTCACAGAAAAGCATCTTA
CGGATGGCATGACAGTAAGAGAATTATGCAGTGCTGCCATAACCATGAGTGATAACACTGCGGCCAACTTACTTCTGACAACGATCGGAGGACCGAAGGAGCTAACCGCTTTTTTGCACAACATGGGGGATCATGTAACTCGCCTTGATCGTTGGGAACCGGAGCTGAAT
GAAGCCATACCAAACGACGAGCGTGACACCACGATGCCTGCAGCAATGGCAACAACGTTGCGCAAACTATTAACTGGCGAACTACTTACTCTAGCTTCCCGGCAACAATTAATAGACTGGATGGAGGCGGATAAAGTTGCAGGACCACTTCTGCGCTCGGCCCTTCCGGC
TGGCTGGTTTATTGCTGATAAATCTGGAGCCGGTGAGCGTGGGTCTCGCGGTATCATTGCAGCACTGGGGCCAGATGGTAAGCCCTCCCGTATCGTAGTTATCTACACGACGGGGAGTCAGGCAACTATGGATGAACGAAATAGACAGATCGCTGAGATAGGTGCCTCAC
TGATTAAGCATTGGTAACTGTCAGACCAAGTTTACTCATATATACTTTAGATTGATTTAAAACTTCATTTTTAATTTAAAAGGATCTAGGTGAAGATCCTTTTTGATAATCTCATGACCAAAATCCCTTAACGTGAGTTTTCGTTCCACTGAGCGTCAGACCCCGTAGAA
AAGATCAAAGGATCTTC

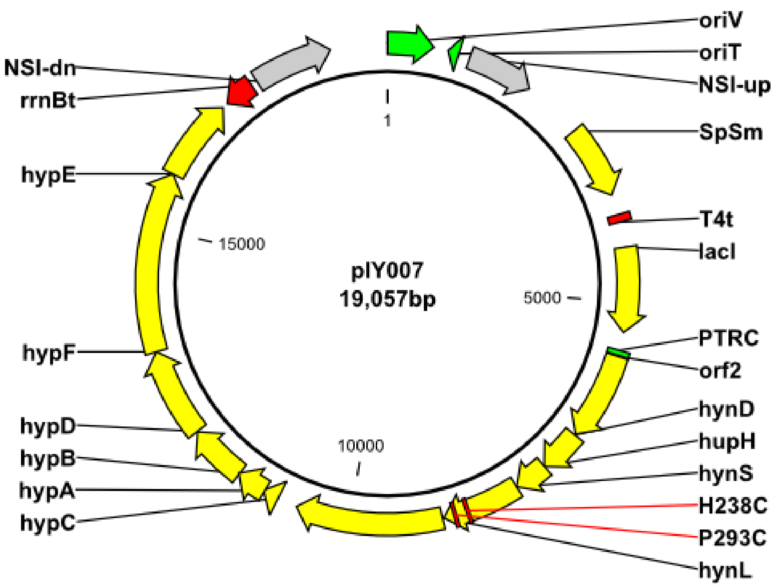

TTGAGATCCTTTTTTTCTGCGCGTAATCTGCTGCTTGCAAACAAAAAAACCACCGCTACCAGCGGTGGTTTGTTTGCCGGATCAAGAGCTACCAACTCTTTTTCCGAAGGTAACTGGCTTCAGCAGAGCGCAGATACCAAATACTGTCCTTCTAGTGTAGCCGTAGTTAG
GCCACCACTTCAAGAACTCTGTAGCACCGCCTACATACCTCGCTCTGCTAATCCTGTTACCAGTGGCTGCTGCCAGTGGCGATAAGTCGTGTCTTACCGGGTTGGACTCAAGACGATAGTTACCGGATAAGGCGCAGCGGTCGGGCTGAACGGGGGGTTCGTGCACACAG
CCCAGCTTGGAGCGAACGACCTACACCGAACTGAGATACCTACAGCGTGAGCTATGAGAAAGCGCCACGCTTCCCGAAGGGAGAAAGGCGGACAGGTATCCGGTAAGCGGCAGGGTCGGAACAGGAGAGCGCACGAGGGAGCTTCCAGGGGGAAACGCCTGGTATCTTTA
TAGTCCTGTCGGGTTTCGCCACCTCTGACTTGAGCGTCGATTTTTGTGATGCTCGTCAGGGGGGCGGAGCCTATGGAAAAACGCCAGCAACGCGGCCTTTTTACGGTTCCTGGCCTTTTGCTGGCCTTTTGCTCACATGTTCTTTCCTGCGTTATCCCCTGATTCTGTGG
ATAACCGTATTACCGCCTTTGAGTGAGCTGATACCGCTCGCCGCAGCCGAACGACCGAGCGCAGCGAGTCAGTGAGCGAGGAAGCGGAAGAGCGCCTGATGCGGTATTTTCTCCTTACGCATCTGTGCGGTATTTCACACCGCATATGGTGCACTCTCAGTACAATCTGC
TCTGATGCCGCATAGTTAAGCCAGTATACACTCCGCTATCGCTACGTGACTGGGTCATGGCTGCGCCCCGACACCCGCCAACACCCGCTGACGCGCCCTGACGGGCTTGTCTGCTCCCGGCATCCGCTTACAGACAAGCTGTGACCGTCTCCGGGAGCTGCATGTGTCAG
AGGTTTTCACCGTCATCACCGAAACGCGCGAGGCAGGATATCCGGCAGCCGGCGGAGCGCTGCTTTCTTGGCAAGCGGTCGCCAGCCCCAACGCCAGGGCTGCCAGCCCGAAACAGCGGGGCAAGGCAGCTTGGAAGGGCGATCGCAGCACGGGCATGGCAATGTCTCTC
TGAAGGAATGCAGACCTTATTCGTACAGCCAGGGTTGAATCGTGGGGGTCCAATCACTTAGCTCTGCTGGGCTAAACCAGAGAGCAATTTCCTGTTGTGCTGTTTCGATTGCATCCGAGCCATGGATGATGTTGCGGCCAATATTGACACCAAAATCACCACGGATGGTG
CCCGGTTCTGCCGTCAGCGGATTGGTAGCGCCGATCAACTTGCGAGCAGCCGCCACAACGCCTTCGCCTTCCAAGACGATCGCCACGATCGGCCCAGAGGTGATGAACTCGACGAGGCCATTGAAGAAGGGGCGCTCGCGGTGGACAGCATAGTGCTGTTCGGCCAGCTC
GCGACTGGGCTTCAGCTGCTTTAGGCCCACCAGTTTGAAGCCTTTTTGCTCAAAGCGGCCGATGATCGTACCGACCAAACCCCGCTGAACGCCATCGGGCTTGATGGCAATAAATGTGCGTTCCACAGACATCTAGATAGTCCTCAAGACGAGGCAAGCATTGAGCTTGC
CTTCCTATGGTTCGGGATCACTGGGATTCTTGACAAGCGATCGCGGTCACATCGCTATCTCTTAGGACTTCGCAGCGGGCGAGTCGGATTGACCCGGTAGGGATTTCGCCAGATCAATGCCCGTGGTTTGTTTCAGCTTCTCCAGCAAGCTAGCGATTTGGGTAGCGCTG
CCTTCCCCTTCGCCAATCACAGTGATCGACTCCACGTCGATATCTGGCACGGTGCCTGAAAGCGTGACGAGCAGGGACTCGAAGCTTGCATGCCTGCAGGTCGACTCTAGAGCTTTATGCTTGTAAACCGTTTTGTGAAAAAATTTTTAAAATAAAAAAGGGGACCTCTA
GGGTCCCCAATTAATTAGTAATATAATCTATTAAAGGTCATTCAAAAGGTCATCCACCGGATCAATTCCCCTGCTCGCGCAGGCTGGGTGCCAAGCTCTCGGGTAACATCAAGGCCCGATCCTTGGAGCCCTTGCCCTCCCGCACGATGATCGTGCCGTGATCGAAATCC
AGATCCTTGACCCGCAGTTGCAAACCCTCACTGATCCGCATGCCCGTTCCATACAGAAGCTGGGCGAACAAACGATGCTCGCCTTCCAGAAAACCGAGGATGCGAACCACTTCATCCGGGGTCAGCACCACCGGCAAGCGCCGCGACGGCCGAGGTCTTCCGATCTCCTG
AAGCCAGGGCAGATCCGTGCACAGCACCTTGCCGTAGAAGAACAGCAAGGCCGCCAATGCCTGACGATGCGTGGAGACCGAAACCTTGCGCTCGTTCGCCAGCCAGGACAGAAATGCCTCGACTTCGCTGCTGCCCAAGGTTGCCGGGTGACGCACACCGTGGAAACGGA
TGAAGGCACGAACCCAGTGGACATAAGCCTGTTCGGTTCGTAAGCTGTAATGCAAGTAGCGTATGCGCTCACGCAACTGGTCCAGAACCTTGACCGAACGCAGCGGTGGTAACGGCGCAGTGGCGGTTTTCATGGCTTGTTATGACTGTTTTTTTGGGGTACAGTCTATG
CCTCGGGCATCCAAGCAGCAAGCGCGTTACGCCGTGGGTCGATGTTTGATGTTATGGAGCAGCAACGATGTTACGCAGCAGGGCAGTCGCCCTAAAACAAAGTTAAACATCATGAGGGAAGCGGTGATCGCCGAAGTATCGACTCAACTATCAGAGGTAGTTGGCGTCAT
CGAGCGCCATCTCGAACCGACGTTGCTGGCCGTACATTTGTACGGCTCCGCAGTGGATGGCGGCCTGAAGCCACACAGTGATATTGATTTGCTGGTTACGGTGACCGTAAGGCTTGATGAAACAACGCGGCGAGCTTTGATCAACGACTTTTGGAAACTTCGGCTTCCCC
TGGAGAGAGCGAGATTCTCCGCGCTGTAGAAGTCACCATTGTTGTGCACGACGACATCATTCCGTGGCGTTATCCAGCTAAGCGCGAACTGCAATTTGGAGAATGGCAGCGCAATGACATTCTTGCAGGTATCTTCGAGCCAGCCACGATCGACATTGATCTGGCTATCT
TGCTGACAAAAGCAAGAGAACATAGCGTTGCCTTGGTAGGTCCAGCGGCGGAGGAACTCTTTGATCCGGTTCCTGAACAGGATCTATTTGAGGCGCTAAATGAAACCTTAACGCTATGGAACTCGCCGCCCGACTGGGCTGGCGATGAGCGAAATGTAGTGCTTACGTTG
TCCCGCATTTGGTACAGCGCAGTAACCGGCAAAATCGCGCCGAAGGATGTCGCTGCCGACTGGGCAATGGAGCGCCTGCCGGCCCAGTATCAGCCCGTCATACTTGAAGCTAGACAGGCTTATCTTGGACAAGAAGAAGATCGCTTGGCCTCGCGCGCAGATCAGTTGGA
AGAATTTGTCCACTACGTGAAAGGCGAGATCACCAAGGTAGTCGGCAAATAATGTCTAACAATTCGTTCAAGCCGACGCCGCTTCGCGGCGCGGCTTAACTCAAGCGTTAGATGCACTAAGCACATAATTGCTCACAGCCAAACTATCAGGTCAAGTCTGCTTTTATTAT
TTTTAAGCGTGCATAATAAGCCCTACACAAATTGGGAGATATATCATGAAAGGCTGGCTTTTTCTTGTTATCGCAATAGTTGGCGAAGTAATCGCAACATCCGCATTAAAATCTAGCGAGGGCTTTACTAAGCTGATCCGGTGGATGACCTTTTGAATGACCTTTAATAG
ATTATATTACTAATTAATTGGGGACCCTAGAGGTCCCCTTTTTTATTTTAAAAATTTTTTCACAAAACGGTTTACAAGCATACTAGAGGATCGGCGGCCGCGGATCTGGGCCGCGGGTCATGGCTGCGCCCCGACACCCGCCAACACCCGCTGACGCGCCCTGACGGGCT
TGTCTGCTCCCGGCATCCGCTTACAGACAAGCTGTGACCGTCTCCGGGAGCTGCATGTGTCAGAGGTTTTCACCGTCATCACCGAAACGCGCGAGGCAGCAGATCAATTCGCGCGCGAAGGCGAAGCGGCATGCATTTACGTTGACACCATCGAATGGTGCAAAACCTTT
CGCGGTATGGCATGATAGCGCCCGGAAGAGAGTCAATTCAGGGTGGTGAATGTGAAACCAGTAACGTTATACGATGTCGCAGAGTATGCCGGTGTCTCTTATCAGACCGTTTCCCGCGTGGTGAACCAGGCCAGCCACGTTTCTGCGAAAACGCGGGAAAAAGTGGAAGC
GGCGATGGCGGAGCTGAATTACATTCCCAACCGCGTGGCACAACAACTGGCGGGCAAACAGTCGTTGCTGATTGGCGTTGCCACCTCCAGTCTGGCCCTGCACGCGCCGTCGCAAATTGTCGCGGCGATTAAATCTCGCGCCGATCAACTGGGTGCCAGCGTGGTGGTGT
CGATGGTAGAACGAAGCGGCGTCGAAGCCTGTAAAGCGGCGGTGCACAATCTTCTCGCGCAACGCGTCAGTGGGCTGATCATTAACTATCCGCTGGATGACCAGGATGCCATTGCTGTGGAAGCTGCCTGCACTAATGTTCCGGCGTTATTTCTTGATGTCTCTGACCAG
ACACCCATCAACAGTATTATTTTCTCCCATGAAGACGGTACGCGACTGGGCGTGGAGCATCTGGTCGCATTGGGTCACCAGCAAATCGCGCTGTTAGCGGGCCCATTAAGTTCTGTCTCGGCGCGTCTGCGTCTGGCTGGCTGGCATAAATATCTCACTCGCAATCAAAT
TCAGCCGATAGCGGAACGGGAAGGCGACTGGAGTGCCATGTCCGGTTTTCAACAAACCATGCAAATGCTGAATGAGGGCATCGTTCCCACTGCGATGCTGGTTGCCAACGATCAGATGGCGCTGGGCGCAATGCGCGCCATTACCGAGTCCGGGCTGCGCGTTGGTGCGG
ATATCTCGGTAGTGGGATACGACGATACCGAAGACAGCTCATGTTATATCCCGCCGTTAACCACCATCAAACAGGATTTTCGCCTGCTGGGGCAAACCAGCGTGGACCGCTTGCTGCAACTCTCTCAGGGCCAGGCGGTGAAGGGCAATCAGCTGTTGCCCGTCTCACTG
GTGAAAAGAAAAACCACCCTGGCGCCCAATACGCAAACCGCCTCTCCCCGCGCGTTGGCCGATTCATTAATGCAGCTGGCACGACAGGTTTCCCGACTGGAAAGCGGGCAGTGAGCGCAACGCAATTAATGTAAGTTAGCGCGAATTGATCTGGTTTGACAGCTTATCAT
CGACTGCACGGTGCACCAATGCTTCTGGCGTCAGGCAGCCATCGGAAGCTGTGGTATGGCTGTGCAGGTCGTAAATCACTGCATAATTCGTGTCGCTCAAGGCGCACTCCCGTTCTGGATAATGTTTTTTGCGCCGACATCATAACGGTTCTGGCAAATATTCTGAAATG
AGCTGTTGACAATTAATCATCCGGCTCGTATAATGTGTGGAATTGTGAGCGGATAACAATTTCACACAGGAAACAGACCATGGATGGCTTCCATTATTGGTAGCGGCTTTTTGATCATTGCACCACTGCTGCATTCGGTAATGGGGAAATGGGCTCTGCTTGGAATAGTG
TTGTTGTCCATTCTGGCATATGCGCTGGGGTCGGTTATTCGCTTCAATATACGCCATGCAGAACCTGAGCTAGCTAGCAATCAACAAAGCAGTATTGTGACGCTTGAAAAAGCAAGTCAATGGGCGCTTGGTGCGGCATATGCCATTTCCGTGGCGTTTTATATTAGTTT
GTTTGCAGCGTTTGTTTTTGATCGTTTATCAATCTCTGATACGACGTACATCAAACTGTTTACCAGTGGGTTGCTTGTCATCATTACGGTGGTCGCTTGGCTCCGTGGTGCTAGGGGGCTAGAGACCATTGAGCTATTCGCCGTGACAATAAAGTTGGCTATTATAGTAG
GTGTGCTCGCAGCGCTGGCAACCTACGACATACAAGTACAAAGTGCTTGGTTTCAACATGAAGCGATACAGGCATTGAGCCATTTTGAAACTGTTAGCATGCTCGCAGGCATGCTCATGGTAACGCAAGGGTTCGAGACAACCCGTTTTATGGGGAATAACTACACGCCA
GAACAGCGCATTAAAGCGAGTCGTTATGCTCAGTGGATCGCTATTTTTCTCTATGTTGTTTTTATCGGTCTAACGTGTCCTATTTTTCTGGATTTTCCGATCACGGAATTAAACGAAACAACGATTAGCTACACCCTAGGGCAAGCAATATGGGTATTGCCGATTTTATT
ACTGGTTGCCGCCACGGCAAGTCAATTAAGTGCAGCATTAGCCGATACTATCGGTGGCGGAGGCCTCTTAAAAGAGCTTTTCCATTTACCTATCTCCCCGCAATTTTACTACGTTTTAGTCATCGCCATCGCAGGTATATTAGTTTGGTCATCCAACGTATTTGAAATTA
TTAATCTCGCTTCAAAAGGGTTTGCACTTTATTACCTGATCCAAACAATAATCGCGGTCAAACTCGTGATGCGTCAAAGCACAGGTCAACGCATTAGGTCAACAAAATTACTTGGTCTATCGGTGATCGTTCTCTGTTTATTCTTTGTGATTGGCTGGTCAATTCCAGCA
CCTCATAGCTAATAAGGAAAATACCGATGCTATCAGTAGAAACTACACCACCAATTGACCAAATCAAAAACGCGGCTAAGCGCTACGCTATCATTGGTGTTGGAAATCTATTGCAGCGAGATGATGGCGTTGGTGTGCACGCGGTTCGTGCTATGCAACCCCTGCTAAAG
TCTTATTCAAATGTATCTTGCATTGACGCTGGGACACTGAGCTATGAACTTTTGGAGTGGGTTGCGAGTTCCGATCATACGATAGTCATCGACGCGGCTTATATGCGCTGTCCGCCTGGCACGGTGAGAGTGTTTGAGAACGAGGCAATTGCGGATCAGTTTCAGCAAGC
GACGACCCATTCCGTTCACCAGATCACGTTACGCGACACATTGTTGACCAGCCAAACGTTATATTCAAAACCTTCTGCGGTGACGTTAATTGGCGTAGAGCCGGAGGCGATTGAGTGGGGAACCTCTTTGAGTCCAAAGGTAAATGCAGCGCTATCATCGATCATGGATA
TCGCATTTGCTTGCTTATCAGGCGACAACGTTCAGACCTATAAACCGGCAACCCAAAAGGAGGCACGATGACCGAAGAACTCATTTTTATGACATCAAATGCTGATAAAACGGGCAACGTAATGCCGCTTTTGCATCAGATACGTCATGCGTTGTCTCAGTTAATTGAAC
GACAAGAACAAACCACGATTGATCTGCGACGATTGCCTTTAAGCGCTAGCGAAGAAGCGCAGTTAGAAGCTTTTCTTGGGCATGGAGAGGTTAAAGCTGATATTCAGGCGCTGGGTGATACGGTGCTAATCGAATCACGCTACGCAGGCGTTTGGCTTGAAATTCACTAT
AACGAAGATGTGGAGATCATGGGCAAATACGTTCATATCTGTACCTGTCCACCAATCATAAAATCTCAGCCAGAAGATATGGTGTTATCGCTCAGCAATATAGTGTCTGACATCCATTCGTTGTCTCATCAATCTTCTGATGAAACGGCTAAGGAGGATTGAAATGGCGC
TACCAACATTAAACAAGCAGTTACAAGCGTCCGGTATCTCAAGACGCACATTTCTTAAGTTTTGCGCTACAACGGCGTCTTTACTGGCATTGCCGCAAAGCGCTGTTGCCGATTTGGCGACTGCCCTTGGCAATGCGAGAAGACCCTCTGTGATTTGGTTGCCCTTTCAA
GAGTGCACAGGGTGTACCGAAGCAATATTGCGCTCTCATGCTCCCACATTGGAAAGCCTTATTTTCGATCATATTTCGTTGGATTATCAGCATACGATAATGGCTGCTGCGGGAGAGCAAGCTGAAGACGCTAGGCGTGCGGCGATGAACGCGCACAAAGGGCAATATTT
GTTGTTGGTTGATGGTTCGGTTCCGGTGGGTAACCCAGGATACTCAACGATCAGTGGCATGAGTAATGTCGATATGCTGAGAGAATCGGCAAAAGATGCTGCGGGTATTATTGCCATCGGTACCTGCGCGTCTTTTGGCGGGATCCCTAAAGCAAACCCAAATCCGACGG
GGGCAGTGGCAGTAAGCGACATTATTACAGACAAGCCAATCGTAAACATTTCAGGCTGTCCGCCACTGCCTATCGCGATTACAGCTGTGTTGGTTCATTACCTGACGTTTAAGCGTTTCCCTGATCTCGACGAATTACAACGCCCACTCGCTTTTTTTGGTGAAAGCATC
TGTGACAGATGTTATCGACGCCCATTTTTTGAACAACGTAAATTTGCAAAATCGTTTGATGATGAGGGGGCAAAAAATGGGTGGTGTTTGTTCGAACTTGGTTGTAAAGGGCCCGAAACCTTTAACGCATGTGCAACGGTTAAATGGAATCAAGGCACGAGTTTTTGTAT
CGAATCTGGCCATCCGTGTCTTGGTTGCTCTGAGCCCGATTTCTGGGATAAAAGCAGCTTTTACCAAGCCTTGGGTCCATGGGAGTGGTACAAATCCAAACCCGGCAAAGGTGCACAGAAGCATGCTGGGAAAAACTCATAAGATAATAGGCAAGGCTCATGGAAAATAC
AGCAAGTAACAACCGGTTAGTCGTCGATCCTATCACTCGAATTGAAGGGCATCTTCGAATAGAAGCTGAAATGGATGGGAATACCATCAAACAGGCGTTCTCATCAGGCACGTCTGTTCGGGGAATTGAACTGATTTTACAAGGCAGAGATCCGCGTGACGCTTGGGCTT
TTGCGCAACGTATCTGTGGCGTCTGCACGCTGGTGCATGGTATGGCATCGGTGCGTGCTGTCGAAGATGCAATTAGAAAAGCTTGGCGGTCAAACGCAAAATTAGGGGTTGCCATTGGAAAGCCCTCCATGACATCTATGCCAAAAGGACCGATGCAACATGGTAAAAAA
GGGCACCGACAGTCACGTACTTCAATAGGCGTACTGAGTGAAGCAGAAATGGCTATCCCTCAAAATGCACAACTGATAAGAAACATCATGATTGCAACCCAATATGTGCATGATCACGTGATGCATTTTTATCACTTGCATGCCCTAGATTGGGTTGACGTTGTTTCTGC
ACTAGATGCAGATCCAACGAGAACCGCTACGCTCGCCGGTCAATTGAGTGATTATCCTCGTTCATCGCCGGGATATTTCAAAGATGTGAAGCAAAAAGTCAAAACGCTGGTTGAGTCTGGGCAGCTAGGGATATTCAGTAACGCGTATTGGGGGCATCCTGGCTATAAAC
TGCCACCTGAAGTTAACTTGATGGCATTAGCACATTATTTAGATGCGCTAACGTGGCAGCGTGAAGTTGTAAAAGTTCACACCATATTTGGAGGGAAAAACCCTCATCCTAATTTTGTCGTTGGCGGCGTGCCTTCACCGATTAATCTCAATGCGTCAACGGGTATTAAC
ACGAGTCGATTAGTGCAACTACAAGATGCTATCACGCAAATGAAGAGCTTTGTCGATCAGGTGTATTACCCCGATATTGTGGCGATTGCGGGTTATTACAAAGAGTGGGGGACACGAGGGGAAGGGCTGGGTAACTTTCTTACCTATGGAGACTTACCTATGACATCAAT
GGATGACCCTGATTCTTTCTTGTTTCCACGAGGTGCAATACTTGGTCGAGACTTGAGTAAAGTGCATGACCTTGATCTAGATGATCCCTCTGAAATTCAAGAATTCGTCTCTTCCTCCTGGTATCGATATAGTGGAGGGAACGCAAGTGGTTTACACCCTTTTAATGGAC
AAACAACACTCGAGTATACTGGTCCGAAACCGCCTTACAAGCACCTAAATGTAGGGGCTGAATATTCATGGTTGAAAAGTCCGCGTTGGAAAGGCCATGCGATGGAGGTGGGACCGCTGGCTCGCGTGCTAATGATGTATGCTAAAAAAGATGCCGCTGCGCAAGACATC
GTTAATCGATCTCTTTCTATCTTGGATTTAGAGACCTCTGCACTTTTCTCCACACTCGGTAGGACGCTCGCCAGAGCAGTGGAAACAAAAATTGTGGTTAACCAGCTACAGTCTTGGTATGACCAACTATTGGATAATATCGCAAAGGGCGATACCGATACGTTTAACCC
TCTGTATTTTGACCCTACCAATTGGCCAATTAAAGGCCAGGGGGTAGGCGTGATGGAAGCGCCTCGTGGCGCGTTGGGGCATTGGTTGGTCATGCAAAATGGCAAAATTGAGAATTACCAATGTGTCGTGCCTACGACATGGAACGCTGGACCTCGAGATCCCAACTCAC

AGGCAGGTGCTTATGAAGCCGCTCTGCAAGATAAACATACGCTACATGATCCTGACCAACCTTTAGAGATTTTGCGAACACTTCATAGTTTTGACCCCTGCTTAGCATGTGCCGTGCACGTAATGGACGAAACAGGGGAAGAGCGTTTGCGTCTAAAAGTTCGTTAACCT
TACTTGAGGAATATTATGAAAATCAAAAATAGTATTAGAAAGTCAGTTACTTCTTTAATTGCCTTTTTTTACCTGGTGGTAGGTTCACCCGTTAGTTATGCGCACCCGGGTCATCAAGGCGATCATTCACTACTTTTGGGATCGCTGGTGTCAACAATCGTGCTTGTCAC
CGTTGCGCTGTTTATTTATCGAGCTAGAGCAAGTAGGCCCTATCAACGGGTAAAACAGTAATGTGTCTCGCTATCCCCATGAAAGTCATTGCAATTAAAGGCTTTAACGCAACGTGTGAAGCGAAAGGTGTCTCTCGCGAGGTGAGTTTACATTTAGTGCAAGGGCTAGA
GGTCAAAGTTGGCGACTATGTGATGGTACATGTTGGTTATGCTCTCCAAGTGATCACCTATGATGAAGCGCAGGTGACATGGGAGATGCTCGATCAGGTCATTGCTTACGATGCATGAAATTAGTCTTTGCTATAGCTTGCTTGACACAGTCGCTGTTCACCAGCGAGCA
AACATGAATAAGTCTGTCAGTCTGGTTCACGTCAAAGTCGGCCCACTGTCAGGTGTTGAGCCTGACTTGCTGCACCATGCGTTCTTGGCGTGTAGAACTCACACCATTGGCGATCAGGCAACGCTTAGAATTGACACAAGCGCTATCAAGATACGCTGTAAATTGTGTGG
CGAATTCAGCCGAGTTTCGGTGAATAGCATCCTATGTGCAAGTTGCGGTGCTTGGCAAACCGACCTCATTGAGGGCGATGAATTTATATTGCAACGAATAGAGTTTATCGCACCTGACAATTTTACAGAATCAAAGGAGAGAAAACATGTGCGGTAACTGTGGTTGCGAA
ACGCAAGTAAAATCAGAGAAAACTGACAAGCCATCAGCAAATATCGTTGAGGTTCACGCAAATTTAAAAAGCAACAATGACAACCAAGCGATAGCAAACCGTGCTTTATTTGACGCACATAATGTACTAGTGATTAATTTGATGTCATCCCCAGGCAGTGGAAAAACCAG
GCTACTCGAAGAAACGATACGTGCGCTTAAGTCGCAATACGCGATGGCGGTGATTGAAGGCGACCTCGAAACTGAAAATGACGCAAATAGAATTAGACGTCATGGTGTGCAGGCGGAGCAAATCGCCACGGGCCAAGGGTGTCACTTGGACGCCAGTATGGTGCAAAAGG
TACTTGGAAGATTTAGCTTACAAAAACTAGACGTTTTGTTTATAGAGAATGTTGGCAATTTGATTTGTCCAGCGTGTTTTGATTTAGGACAGCACTTAAACATTATTTTGCTGTCCGTACCAGAAGGTGATGATAAGCCTGAAAAGTACCCAGTGATGTTTCGTGCCGCG
GACGTCATGCTTATTTCTAAGACCGATTACTTGCAATTTCACGATGAATTCAATGTACCTCGCGCGATTGAATCATTTCGTAAAGTGGGCAACGATGCGCCCGTTCTCGAAGTGTCTTCATTGAAAAGTCAACATCTAGAGCGTTGGTTTTCTTATTTTACGCACGCCAT
TACAGCTCATCAGTCTCAGTCGTTACAAAATAGCGGAGTCTAAAGTCTTATGTACAGTGTTGAATCGTTGCTAAGAGACATTAAGAATACCTCGCTTTCAAAACCGTTTCGTATTCTTAATGTTTGCGGTGGCCATGAGCGTGCGATTACGCGCGCTGGATTTAGAACGT
TGTTTCAGCACAATATTCATCTGATACCTGGGCCAGGGTGCCCCGTTTGTATTTGCCCCGAAGAGGATATTGCATACGCGATACACTTAGCCATTAACGAAAATGTCGTTATCGTTAGTTTTGGTGACATGCTCCGCGTCCCAGTCGAACACGAAATGGGTGGCTGTAGC
TCATTGATAGACGCAAAAAATCAAGGGGCCGATATCCGTCCGATATCTTCTCCTCAAGAAGCGGTATCTATAGCATTGGAAGAACCTCGTAAGGTTATTGTGTTTTTTGCTGTTGGTTTTGAAACGACTATGGCACCGATTGCAGCAACACTGCTTAATGATTTACCCAA
TAACTTTAAAGTATTATTGTCTGGACGACTTACTTGGCCAGCAGTTGCCCATGTGCTTGAGAGTCAACCGAATACTTTTGATGCACTGATTGCGCCAGGCCACGTTGCGACAATTATGGGTAGTGAAGAATGGCAGTTTGCAATCGACCATCATAATTTGCCTGTCAGTA
TTGCTGGTTTTCATCCAGAAAGTTTGCTGTTGTCGCTCCAAACGCTGCTCGGTAATTGTTCAAATAAAGTCGTCACACTGAGTAACCGCTATCCCGAAGTGGTAAAGCAAAACGGCAATGCAGCAGCTAAAGCAATAATCAATAAAGCGTTCACTATCGTTGACGCACAT
TGGCGAGGAATTGGTGTTATTCCTGGTTCTGGATTTTCCTTTGCTTCGAGACTATCGCACCTAGACGCCACAAACGATTATGCGCCAGTCGATTTCCCTTCGCAATGTGCGCAAAACGTTCCTGAAACCACATCGCCCTGTGAAAAAGTGATCCTCGGAAAAATGGCACC
TGATGCGTGCCCGTTTTTTGGTCAGGAATGCAAACCAGCATCCCCTAAAGGCGCATGTATGGTGTCTGATGAGGGGGCTTGCCGAATTTGGTATAGCTCTGGTGAGCGCTCGATAACAAATGTTATCAAAAAGGGGAATACGCTAAAGGTGGAGATGAAGTGATGACAAC
TGCCCGCACATATGTTGCCAATACGAATAAGAGTAAAACTATCGCCTATGAGTTGAACATTACTGGAGTCATCCAGGGGGTTGGGTTCAGACCGTTAGTTTACTGCTTAGCGTATGAAAAAAGTATTGTTGGTTGGGTACAAAACGACTGTGGATGTGTGCGAATACACG
CTGAAGGCACTGAGCTAGACGTTGAACAATTTGTATTCGACTTACTGAACAATGGGTCGCTAGTTTCCATATCCTTGCTGGAAAAAAAGCAGGTTAAACTGAGTAATGTAGACTCGTTCACGATAAAAGAAAGCGGTCATGATATAGCGTGCGGGGCGATATCAATCCCC
AAAGATGTCTATTTGTGTGGAGCGTGTAAAGCTGAGTTACTGTCAAATACTAACCGACGTGGCACCTATAGCTTTATCGCTTGCAGTAAATGTGGTCCACGATTTTCTATGCTGCGAGCAATGCCATACGACAGAAAGAACATGAGTATGTCAGCATTTCCAATGTGTGA
GTTATGTCGTAAAGAATATCAATCGCCAACTGATAGGCGCTTTCATGCTCAACCAATAAGCTGTCGCGTATGCGGGCCTGAAGTATTTTGTTCAACTGTCGGCGGGCGCGTGGTCTCGCAAGGCGATGTTGATGTTGTAGCAACGGTTGTCGCGTATTTGAATCAAGGCA
AAATTGTCGCGTTAAAAAGTATAGGCGGTTATCACTTGCTCTGTGACGCACAAAGCACTGAAGCGGTAGACCTTTTACGCCAGCGTAAAAATAGACCAGATAAACCTTTCGCGGTCATGCTCCCAGAGCCTCGTGCAGACGAAACAAGTGAGGATTGGTTAGATAAATGC
GTTGCTGTAGACAGTCATCAAAGAGCGCTCTTACTAAGCCCCGTGCGACCCATTTTACTTGCAAAAAAGCAATCGAATGTACCCATTGCAGATAACGTAGCGCCCATGCTTCGTGATTTGGGGGTGATGTTGCCGTGCAGCGGACTGCATTTACTCATTATGCAGCAATT
CAATCGCCCCATGGTGGCTACGTCAGGAAATATTTACGGTGAACCGATGGTCACATCGACGTCATCGGCACAGCAACACCTTTCGATGTTGGCAGACATCTTCGTGCACCATAATCGAGACATTTTTCACAAACTGGATGATTCTGTGCTGCGAACGCTTAAGTCAGGCA
CCATTCCAATTCGCCTGGGGCGGGGTATAAGTCCGATAGAATTATCATTGCCATATACTGTGCAGGAACCAATGCTTGCTGTTGGCGCGAATCAGAAAAATACAGTCGCAATAGCTTTGAACAATCGACTTATCGTAACACCGCATATTGGCGACCTCGATTCTCCGATA
ATGCAAGCGCACTTTGAAAAATGTATCAGTGACTTTCAAGCTCTCTATCATGTTCATCCACAGCGTATTCTTTGCGATAGCCATCCTGGCTATATCAGTTCCCGTTGGGCCAAAGGCAGTGAACTACCGACTACTAAAGTTTTACATCACCACGCTCATGCATCAGCGTG
GGCATTAGCTGCTGAAATATCGACACCAAGCTTGGTTTTCGTGTGGGATGGAACGGGATTAGGCGAGAATGGCGAGATGTGGGGAGGGGAAGTTTTTTGTGGGACGCCGGGCAATTGGAAACGTGTTGCCTCATTAGCGCCTATGAAGTTGCAAGGCGGCAACAAGGTTC
CAAATCAACCATGGCGAAGTGCAGCATCACTAGTGTGGGGTACTAATTTCACCGGCATTGATTTTAAACCATTTGATCCACAACAATTAAGCTACAACGCATGGCAAAACGAACTCAATTGTCATGTTAGTAGTTCAATGGGAAGACTGTTCGACGCGGCGGCATTTGCC
TGCTGTGAACAGAGCGAAGTCTCTTATGAAGGGCAGGCGCCGATGATGTTGGAAAGCGTTGCGTCGACGCCCTCTAAGGTTATGCCGTTAACGTTGGTCCAGAAAAACGACGAATTTATTCAAATGGATTGGCGAACACTGGTACCGGAACTATTTGATTTCAAAGTGTC
TCCTCAAACTCGTGCTGCAAATTTCCACGCTTCGTTAATTGAAACGGTATGGGAGGTAACCCTACATTGCCGTAAAAAGTTTAATTTTGACCACATTGGATTGGCAGGAGGCGTCTTCCAAAATCGCATTTTGTGTGACGGTATATCACAACGGTTTTTACAATCAGACA
TTCCTGTGGTTATTCCAAGTAGTTTGCCATTAAACGATGCCGCAATAAGTGTTGGGCAAATCCACGAATACTGTTCACGGAGGTCATAATGAAAAGCGCAGAGGGCAATAATCATTCATCATCAGAGCCGTTGGGCACGGAATCTATTCAATTAGCTCAGGGGAACGGCG
GAACACTGACAAAAAGACTCATTGATCATGTTTTTAAAAATAAAGCGAACAAAGATCTAGATCTGCTTCATGATGCCGCCACAGTAACTTTTGATGGTCCTTATCTGAGTATAACAACGGATAGCTTCGTTGTATCCCCCCCAACATTTCCAGGCGGCAATGTAGGCGCG
TTGAGTGTTTACGGTACCGTCAATGATTTAGCAGTCGTTGGGGCAACACCACGGTACATCAGTACTGCATTTATTATTGAAGAAGGCTTTTCCCTTTCAACATTGAAACAAATCGTTAATGGAATGCATCAAGCTGCCGAAGAAACGAAGGTCGCCATTGTAACAGGTGA
TACGAAAGTTGTCCCGAAAGGCAGTGGTGGCGGCGTTTTTATCAATACAACGGGTGTTGGAGACAGCCGAGATGCGCCGATTTGGGATACCAGTCTTATAAAGGCAGGCGATCATGTACTGGTTAGTGGTTCTGTAGGTGATCACGGTGCGTGTGTCCTGCTTGCTCGCG
AGGACTATGGTTTGCAAGGTCAGCTAAAGTCTGATTGTGGAAGCGTTGTTCCTCTTATTGATCCAATCAAGCATTTGGAAGGTGTTCGTTTTGTGCGTGACCCAACTCGTGGAGGGTTGTCAGTATTACTCCATGACGTTGCTAATGAAACAGGCTTCGATATCGAGTTA
ATTGAAAATAATATTCCGGTGCGACCTGAAGTGGCAAGCGTGTGTGAAATATTAGGCTTTGATCCGTTTGTTTTAGCGTGTGAGGGACGCATTGTTGCTGTTGTTGCCCCTGACATAAGCGGTAAGGTGCTAGAACACTGGCGGAGCATTCGAAACGGGGAGCAGGCCGA
ACACATTGGTGTAATAGTAAAAAACACCGAATCAAAAGGGCGCGTGACCATTGTTACGCCAATGGGAGGAAGACGTTTCATGAATGAACTCGAAGATGAGCCGCTTCCAAGGATCTGCTGAGATCCTCTAGAGTCGACCTGCAGGCATGCAAGCTTGGCTGTTTTGGCGG
ATGAGAGAAGATTTTCAGCCTGATACAGATTAAATCAGAACGCAGAAGCGGTCTGATAAAACAGAATTTGCCTGGCGGCAGTAGCGCGGTGGTCCCACCTGACCCCATGCCGAACTCAGAAGTGAAACGCCGTAGCGCCGATGGTAGTGTGGGGTCTCCCCATGCGAGAG
TAGGGAACTGCCAGGCATCAAATAAAACGAAAGGCTCAGTCGAAAGACTGGGCCTTTCGTTTTATCTGTTGTTTGTCGGTGAACGCTCTCCTGAGTAGGACAAATCCGCCGGGAGCGGATTTGAACGTTGCGAAGCAACGGCCCGGAGGGTGGCGGGCAGGACGCCCGCC
ATAAACTGCCAGGCATCAAATTAAGCAGAAGGCCATCCTGACGGATGGCCTTTTTGCGTTTCTACAAACTCTTTTGTTTATTTTTCTAAATACATTCAAATATGTATCCGCTCATGGCGGCCCAGATCCCCGGGTACCGAGCTCGAATTTCGAGCTTCTGGAGCAGGAAG
ATGTCGCGGGCATTAGCACCAGCGGTCTGCCAAGCCTCCGCCAGCCGTTGGGTCCCTTCCGCTTGAGCTTTTCCATCTTCGACGATACGGGCGGCGGCCCCCCGCGCTTCCGCGATCGCCCGTTTACAAGCTGCCTCAGCTGGGGCGATCACATCGGCTTGAAGTTGCTG
CTGCACCTGTTTGATCCGCTCCTGCTGCACAGGGAGTTCTGCTTGGCTACGAGCGACTTCGGTAGCAATGTCCGCTTCAGCTTCGGCCACCACCGCTTCGCGCCGCGTCAACGCATCCTGAATCCGGCGCTCGGCCTCGGCTTGGGCGATCGCTACATCGCGATCGATCC
GACGCAGGGCCGTGATCTTGTCATTTTCGGCCGTTTGGATCGCAGAGGCAGCCTGGGCATCGGCTTCAGCAATTCGGGCATCTCGCTGCAGATCAGCCCGCTGCTTGCGTCCACTAGCCGAGAGATAACCGACCTCATCGGAAATGTTCTGGACTTGCAGCGTATCGAGG
ACTAGACCCAGCTGCTCAAGGTCATCCTCCGCCTCTTCCAGCAGACTTTTGGCAAAGGCAATTTTGTCCTCGTTGATCTGCTCCGGCGTGAGGCTGGCTAAAACACCACGCAAGTTGCCTTCGAGGGTCTCCTTGGCAATTTGCTCGATTTCCTTACGGTTTTTGCCAAG
CAGCCGCTCGATCGCGTTGTGGATGGTCGGTTCTTCCCCAGCAATCTTGATATTGGCAACGCCTTCAACAGTCAGGGGAATGCCGCCCTTGGAGAAGGCATTGGAAACGCGCAACTCAATGATCATGTTGGTCAGATCCATGCGGAGCGCTTTTTCCAGCAGAGGTACCC
GCAGGCTGCTGCCGCCCTTGACCAAGCGATAGCCAACTCGGCGGCCATCACTACTGCGGCGACTACTGCCAGCAAAGATCAAAATTTCACTGGGTTGGCAGATGTAGTAGAGATTGCGCAGGACTAAGCTGCCAGCCCCGGCGACTCACCAGTCACAGAAAAGCATCTTA
CGGATGGCATGACAGTAAGAGAATTATGCAGTGCTGCCATAACCATGAGTGATAACACTGCGGCCAACTTACTTCTGACAACGATCGGAGGACCGAAGGAGCTAACCGCTTTTTTGCACAACATGGGGGATCATGTAACTCGCCTTGATCGTTGGGAACCGGAGCTGAAT
GAAGCCATACCAAACGACGAGCGTGACACCACGATGCCTGCAGCAATGGCAACAACGTTGCGCAAACTATTAACTGGCGAACTACTTACTCTAGCTTCCCGGCAACAATTAATAGACTGGATGGAGGCGGATAAAGTTGCAGGACCACTTCTGCGCTCGGCCCTTCCGGC
TGGCTGGTTTATTGCTGATAAATCTGGAGCCGGTGAGCGTGGGTCTCGCGGTATCATTGCAGCACTGGGGCCAGATGGTAAGCCCTCCCGTATCGTAGTTATCTACACGACGGGGAGTCAGGCAACTATGGATGAACGAAATAGACAGATCGCTGAGATAGGTGCCTCAC
TGATTAAGCATTGGTAACTGTCAGACCAAGTTTACTCATATATACTTTAGATTGATTTAAAACTTCATTTTTAATTTAAAAGGATCTAGGTGAAGATCCTTTTTGATAATCTCATGACCAAAATCCCTTAACGTGAGTTTTCGTTCCACTGAGCGTCAGACCCCGTAGAA
AAGATCAAAGGATCTTC

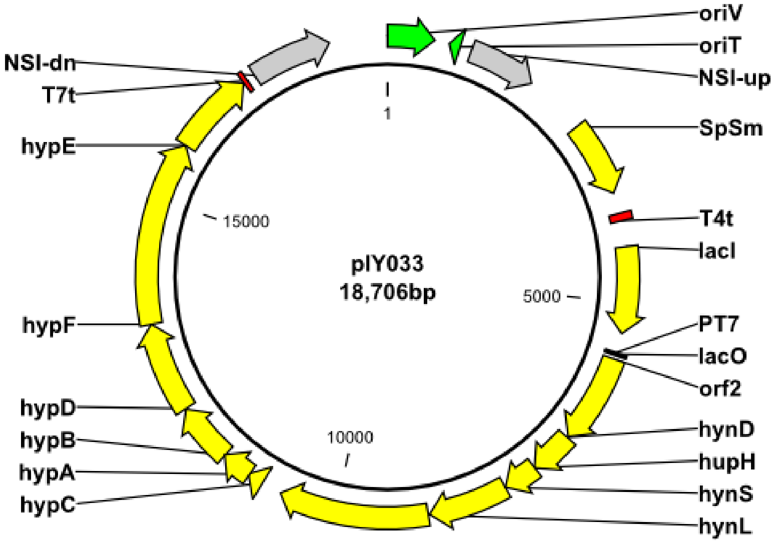

TTGAGATCCTTTTTTTCTGCGCGTAATCTGCTGCTTGCAAACAAAAAAACCACCGCTACCAGCGGTGGTTTGTTTGCCGGATCAAGAGCTACCAACTCTTTTTCCGAAGGTAACTGGCTTCAGCAGAGCGCAGATACCAAATACTGTCCTTCTAGTGTAGCCGTAGTTAG
GCCACCACTTCAAGAACTCTGTAGCACCGCCTACATACCTCGCTCTGCTAATCCTGTTACCAGTGGCTGCTGCCAGTGGCGATAAGTCGTGTCTTACCGGGTTGGACTCAAGACGATAGTTACCGGATAAGGCGCAGCGGTCGGGCTGAACGGGGGGTTCGTGCACACAG
CCCAGCTTGGAGCGAACGACCTACACCGAACTGAGATACCTACAGCGTGAGCTATGAGAAAGCGCCACGCTTCCCGAAGGGAGAAAGGCGGACAGGTATCCGGTAAGCGGCAGGGTCGGAACAGGAGAGCGCACGAGGGAGCTTCCAGGGGGAAACGCCTGGTATCTTTA
TAGTCCTGTCGGGTTTCGCCACCTCTGACTTGAGCGTCGATTTTTGTGATGCTCGTCAGGGGGGCGGAGCCTATGGAAAAACGCCAGCAACGCGGCCTTTTTACGGTTCCTGGCCTTTTGCTGGCCTTTTGCTCACATGTTCTTTCCTGCGTTATCCCCTGATTCTGTGG
ATAACCGTATTACCGCCTTTGAGTGAGCTGATACCGCTCGCCGCAGCCGAACGACCGAGCGCAGCGAGTCAGTGAGCGAGGAAGCGGAAGAGCGCCTGATGCGGTATTTTCTCCTTACGCATCTGTGCGGTATTTCACACCGCATATGGTGCACTCTCAGTACAATCTGC
TCTGATGCCGCATAGTTAAGCCAGTATACACTCCGCTATCGCTACGTGACTGGGTCATGGCTGCGCCCCGACACCCGCCAACACCCGCTGACGCGCCCTGACGGGCTTGTCTGCTCCCGGCATCCGCTTACAGACAAGCTGTGACCGTCTCCGGGAGCTGCATGTGTCAG
AGGTTTTCACCGTCATCACCGAAACGCGCGAGGCAGNNNNNNCGGCAGCCGGCGGAGCGCTGCTTTCTTGGCAAGCGGTCGCCAGCCCCAACGCCAGGGCTGCCAGCCCGAAACAGCGGGGCAAGGCAGCTTGGAAGGGCGATCGCAGCACGGGCATGGCAATGTCTCTC
TGAAGGAATGCAGACCTTATTCGTACAGCCAGGGTTGAATCGTGGGGGTCCAATCACTTAGCTCTGCTGGGCTAAACCAGAGAGCAATTTCCTGTTGTGCTGTTTCGATTGCATCCGAGCCATGGATGATGTTGCGGCCAATATTGACACCAAAATCACCACGGATGGTG
CCCGGTTCTGCCGTCAGCGGATTGGTAGCGCCGATCAACTTGCGAGCAGCCGCCACAACGCCTTCGCCTTCCAAGACGATCGCCACGATCGGCCCAGAGGTGATGAACTCGACGAGGCCATTGAAGAAGGGGCGCTCGCGGTGGACAGCATAGTGCTGTTCGGCCAGCTC
GCGACTGGGCTTCAGCTGCTTTAGGCCCACCAGTTTGAAGCCTTTTTGCTCAAAGCGGCCGATGATCGTACCGACCAAACCCCGCTGAACGCCATCGGGCTTGATGGCAATAAATGTGCGTTCCACAGACATCTAGATAGTCCTCAAGACGAGGCAAGCATTGAGCTTGC
CTTCCTATGGTTCGGGATCACTGGGATTCTTGACAAGCGATCGCGGTCACATCGCTATCTCTTAGGACTTCGCAGCGGGCGAGTCGGATTGACCCGGTAGGGATTTCGCCAGATCAATGCCCGTGGTTTGTTTCAGCTTCTCCAGCAAGCTAGCGATTTGGGTAGCGCTG
CCTTCCCCTTCGCCAATCACAGTGATCGACTCCACGTCGATATCTGGCACGGTGCCTGAAAGCGTGACGAGCAGGGACTCGAAGCTTGCATGCCTGCAGGTCGACTCTAGAGCTTTATGCTTGTAAACCGTTTTGTGAAAAAATTTTTAAAATAAAAAAGGGGACCTCTA
GGGTCCCCAATTAATTAGTAATATAATCTATTAAAGGTCATTCAAAAGGTCATCCACCGGATCAATTCCCCTGCTCGCGCAGGCTGGGTGCCAAGCTCTCGGGTAACATCAAGGCCCGATCCTTGGAGCCCTTGCCCTCCCGCACGATGATCGTGCCGTGATCGAAATCC
AGATCCTTGACCCGCAGTTGCAAACCCTCACTGATCCGCATGCCCGTTCCATACAGAAGCTGGGCGAACAAACGATGCTCGCCTTCCAGAAAACCGAGGATGCGAACCACTTCATCCGGGGTCAGCACCACCGGCAAGCGCCGCGACGGCCGAGGTCTTCCGATCTCCTG
AAGCCAGGGCAGATCCGTGCACAGCACCTTGCCGTAGAAGAACAGCAAGGCCGCCAATGCCTGACGATGCGTGGAGACCGAAACCTTGCGCTCGTTCGCCAGCCAGGACAGAAATGCCTCGACTTCGCTGCTGCCCAAGGTTGCCGGGTGACGCACACCGTGGAAACGGA
TGAAGGCACGAACCCAGTGGACATAAGCCTGTTCGGTTCGTAAGCTGTAATGCAAGTAGCGTATGCGCTCACGCAACTGGTCCAGAACCTTGACCGAACGCAGCGGTGGTAACGGCGCAGTGGCGGTTTTCATGGCTTGTTATGACTGTTTTTTTGGGGTACAGTCTATG
CCTCGGGCATCCAAGCAGCAAGCGCGTTACGCCGTGGGTCGATGTTTGATGTTATGGAGCAGCAACGATGTTACGCAGCAGGGCAGTCGCCCTAAAACAAAGTTAAACATCATGAGGGAAGCGGTGATCGCCGAAGTATCGACTCAACTATCAGAGGTAGTTGGCGTCAT
CGAGCGCCATCTCGAACCGACGTTGCTGGCCGTACATTTGTACGGCTCCGCAGTGGATGGCGGCCTGAAGCCACACAGTGATATTGATTTGCTGGTTACGGTGACCGTAAGGCTTGATGAAACAACGCGGCGAGCTTTGATCAACGACTTTTGGAAACTTCGGCTTCCCC
TGGAGAGAGCGAGATTCTCCGCGCTGTAGAAGTCACCATTGTTGTGCACGACGACATCATTCCGTGGCGTTATCCAGCTAAGCGCGAACTGCAATTTGGAGAATGGCAGCGCAATGACATTCTTGCAGGTATCTTCGAGCCAGCCACGATCGACATTGATCTGGCTATCT
TGCTGACAAAAGCAAGAGAACATAGCGTTGCCTTGGTAGGTCCAGCGGCGGAGGAACTCTTTGATCCGGTTCCTGAACAGGATCTATTTGAGGCGCTAAATGAAACCTTAACGCTATGGAACTCGCCGCCCGACTGGGCTGGCGATGAGCGAAATGTAGTGCTTACGTTG
TCCCGCATTTGGTACAGCGCAGTAACCGGCAAAATCGCGCCGAAGGATGTCGCTGCCGACTGGGCAATGGAGCGCCTGCCGGCCCAGTATCAGCCCGTCATACTTGAAGCTAGACAGGCTTATCTTGGACAAGAAGAAGATCGCTTGGCCTCGCGCGCAGATCAGTTGGA
AGAATTTGTCCACTACGTGAAAGGCGAGATCACCAAGGTAGTCGGCAAATAATGTCTAACAATTCGTTCAAGCCGACGCCGCTTCGCGGCGCGGCTTAACTCAAGCGTTAGATGCACTAAGCACATAATTGCTCACAGCCAAACTATCAGGTCAAGTCTGCTTTTATTAT
TTTTAAGCGTGCATAATAAGCCCTACACAAATTGGGAGATATATCATGAAAGGCTGGCTTTTTCTTGTTATCGCAATAGTTGGCGAAGTAATCGCAACATCCGCATTAAAATCTAGCGAGGGCTTTACTAAGCTGATCCGGTGGATGACCTTTTGAATGACCTTTAATAG
ATTATATTACTAATTAATTGGGGACCCTAGAGGTCCCCTTTTTTATTTTAAAAATTTTTTCACAAAACGGTTTACAAGCATACTAGAGGATCGGCGGCCGCGGATCTGGGCCGCGGGTCATGGCTGCGCCCCGACACCCGCCAACACCCGCTGACGCGCCCTGACGGGCT
TGTCTGCTCCCGGCATCCGCTTACAGACAAGCTGTGACCGTCTCCGGGAGCTGCATGTGTCAGAGGTTTTCACCGTCATCACCGAAACGCGCGAGGCAGCAGATCAATTCGCGCGCGAAGGCGAAGCGGCATGCATTTACGTTGACACCATCGAATGGTGCAAAACCTTT
CGCGGTATGGCATGATAGCGCCCGGAAGAGAGTCAATTCAGGGTGGTGAATGTGAAACCAGTAACGTTATACGATGTCGCAGAGTATGCCGGTGTCTCTTATCAGACCGTTTCCCGCGTGGTGAACCAGGCCAGCCACGTTTCTGCGAAAACGCGGGAAAAAGTGGAAGC
GGCGATGGCGGAGCTGAATTACATTCCCAACCGCGTGGCACAACAACTGGCGGGCAAACAGTCGTTGCTGATTGGCGTTGCCACCTCCAGTCTGGCCCTGCACGCGCCGTCGCAAATTGTCGCGGCGATTAAATCTCGCGCCGATCAACTGGGTGCCAGCGTGGTGGTGT
CGATGGTAGAACGAAGCGGCGTCGAAGCCTGTAAAGCGGCGGTGCACAATCTTCTCGCGCAACGCGTCAGTGGGCTGATCATTAACTATCCGCTGGATGACCAGGATGCCATTGCTGTGGAAGCTGCCTGCACTAATGTTCCGGCGTTATTTCTTGATGTCTCTGACCAG
ACACCCATCAACAGTATTATTTTCTCCCATGAAGACGGTACGCGACTGGGCGTGGAGCATCTGGTCGCATTGGGTCACCAGCAAATCGCGCTGTTAGCGGGCCCATTAAGTTCTGTCTCGGCGCGTCTGCGTCTGGCTGGCTGGCATAAATATCTCACTCGCAATCAAAT
TCAGCCGATAGCGGAACGGGAAGGCGACTGGAGTGCCATGTCCGGTTTTCAACAAACCATGCAAATGCTGAATGAGGGCATCGTTCCCACTGCGATGCTGGTTGCCAACGATCAGATGGCGCTGGGCGCAATGCGCGCCATTACCGAGTCCGGGCTGCGCGTTGGTGCGG
ATATCTCGGTAGTGGGATACGACGATACCGAAGACAGCTCATGTTATATCCCGCCGTTAACCACCATCAAACAGGATTTTCGCCTGCTGGGGCAAACCAGCGTGGACCGCTTGCTGCAACTCTCTCAGGGCCAGGCGGTGAAGGGCAATCAGCTGTTGCCCGTCTCACTG
GTGAAAAGAAAAACCACCCTGGCGCCCAATACGCAAACCGCCTCTCCCCGCGCGTTGGCCGATTCATTAATGCAGCTGGCACGACAGGTTTCCCGACTGGAAAGCGGGCAGTGAGCGCAACGCAATTAATGTAAGTTAGCGCGAATTGATCTGGTTTGACAGCTTATCAT
CGACTGCACGGTGCACCAATGCTTCTGGCGTCAGGCAGCCATCGGAAGCTGTGGTATGGCTGTGCAGGTCGTAAATCACTGCATAATTCGTGTCGCTCAAGGCGCACTCCCGTTCTGGATAATGTTTTTTGCGCCGACATCATAACGGTTCTGGCAAATATTCTGAAATG
AGCTGCCCGCGAAATTAATACGACTCACTATAGGggaattgtgagcggataacaattcccctctagaaataattttgtttaactttaagaaggagatataccATGGCTTCCATTATTGGTAGCGGCTTTTTGATCATTGCACCACTGCTGCATTCGGTAATGGGGAAATG
GGCTCTGCTTGGAATAGTGTTGTTGTCCATTCTGGCATATGCGCTGGGGTCGGTTATTCGCTTCAATATACGCCATGCAGAACCTGAGCTAGCTAGCAATCAACAAAGCAGTATTGTGACGCTTGAAAAAGCAAGTCAATGGGCGCTTGGTGCGGCATATGCCATTTCCG
TGGCGTTTTATATTAGTTTGTTTGCAGCGTTTGTTTTTGATCGTTTATCAATCTCTGATACGACGTACATCAAACTGTTTACCAGTGGGTTGCTTGTCATCATTACGGTGGTCGCTTGGCTCCGTGGTGCTAGGGGGCTAGAGACCATTGAGCTATTCGCCGTGACAATA
AAGTTGGCTATTATAGTAGGTGTGCTCGCAGCGCTGGCAACCTACGACATACAAGTACAAAGTGCTTGGTTTCAACATGAAGCGATACAGGCATTGAGCCATTTTGAAACTGTTAGCATGCTCGCAGGCATGCTCATGGTAACGCAAGGGTTCGAGACAACCCGTTTTAT
GGGGAATAACTACACGCCAGAACAGCGCATTAAAGCGAGTCGTTATGCTCAGTGGATCGCTATTTTTCTCTATGTTGTTTTTATCGGTCTAACGTGTCCTATTTTTCTGGATTTTCCGATCACGGAATTAAACGAAACAACGATTAGCTACACCCTAGGGCAAGCAATAT
GGGTATTGCCGATTTTATTACTGGTTGCCGCCACGGCAAGTCAATTAAGTGCAGCATTAGCCGATACTATCGGTGGCGGAGGCCTCTTAAAAGAGCTTTTCCATTTACCTATCTCCCCGCAATTTTACTACGTTTTAGTCATCGCCATCGCAGGTATATTAGTTTGGTCA
TCCAACGTATTTGAAATTATTAATCTCGCTTCAAAAGGGTTTGCACTTTATTACCTGATCCAAACAATAATCGCGGTCAAACTCGTGATGCGTCAAAGCACAGGTCAACGCATTAGGTCAACAAAATTACTTGGTCTATCGGTGATCGTTCTCTGTTTATTCTTTGTGAT
TGGCTGGTCAATTCCAGCACCTCATAGCTAATAAGGAAAATACCGATGCTATCAGTAGAAACTACACCACCAATTGACCAAATCAAAAACGCGGCTAAGCGCTACGCTATCATTGGTGTTGGAAATCTATTGCAGCGAGATGATGGCGTTGGTGTGCACGCGGTTCGTGC
TATGCAACCCCTGCTAAAGTCTTATTCAAATGTATCTTGCATTGACGCTGGGACACTGAGCTATGAACTTTTGGAGTGGGTTGCGAGTTCCGATCATACGATAGTCATCGACGCGGCTTATATGCGCTGTCCGCCTGGCACGGTGAGAGTGTTTGAGAACGAGGCAATTG
CGGATCAGTTTCAGCAAGCGACGACCCATTCCGTTCACCAGATCACGTTACGCGACACATTGTTGACCAGCCAAACGTTATATTCAAAACCTTCTGCGGTGACGTTAATTGGCGTAGAGCCGGAGGCGATTGAGTGGGGAACCTCTTTGAGTCCAAAGGTAAATGCAGCG
CTATCATCGATCATGGATATCGCATTTGCTTGCTTATCAGGCGACAACGTTCAGACCTATAAACCGGCAACCCAAAAGGAGGCACGATGACCGAAGAACTCATTTTTATGACATCAAATGCTGATAAAACGGGCAACGTAATGCCGCTTTTGCATCAGATACGTCATGCG
TTGTCTCAGTTAATTGAACGACAAGAACAAACCACGATTGATCTGCGACGATTGCCTTTAAGCGCTAGCGAAGAAGCGCAGTTAGAAGCTTTTCTTGGGCATGGAGAGGTTAAAGCTGATATTCAGGCGCTGGGTGATACGGTGCTAATCGAATCACGCTACGCAGGCGT
TTGGCTTGAAATTCACTATAACGAAGATGTGGAGATCATGGGCAAATACGTTCATATCTGTACCTGTCCACCAATCATAAAATCTCAGCCAGAAGATATGGTGTTATCGCTCAGCAATATAGTGTCTGACATCCATTCGTTGTCTCATCAATCTTCTGATGAAACGGCTA
AGGAGGATTGAAATGGCGCTACCAACATTAAACAAGCAGTTACAAGCGTCCGGTATCTCAAGACGCACATTTCTTAAGTTTTGCGCTACAACGGCGTCTTTACTGGCATTGCCGCAAAGCGCTGTTGCCGATTTGGCGACTGCCCTTGGCAATGCGAGAAGACCCTCTGT
GATTTGGTTGCCCTTTCAAGAGTGCACAGGGTGTACCGAAGCAATATTGCGCTCTCATGCTCCCACATTGGAAAGCCTTATTTTCGATCATATTTCGTTGGATTATCAGCATACGATAATGGCTGCTGCGGGAGAGCAAGCTGAAGACGCTAGGCGTGCGGCGATGAACG
CGCACAAAGGGCAATATTTGTTGTTGGTTGATGGTTCGGTTCCGGTGGGTAACCCAGGATACTCAACGATCAGTGGCATGAGTAATGTCGATATGCTGAGAGAATCGGCAAAAGATGCTGCGGGTATTATTGCCATCGGTACCTGCGCGTCTTTTGGCGGGATCCCTAAA
GCAAACCCAAATCCGACGGGGGCAGTGGCAGTAAGCGACATTATTACAGACAAGCCAATCGTAAACATTTCAGGCTGTCCGCCACTGCCTATCGCGATTACAGCTGTGTTGGTTCATTACCTGACGTTTAAGCGTTTCCCTGATCTCGACGAATTACAACGCCCACTCGC
TTTTTTTGGTGAAAGCATCCATGACAGATGTTATCGACGCCCATTTTTTGAACAACGTAAATTTGCAAAATCGTTTGATGATGAGGGGGCAAAAAATGGGTGGTGTTTGTTCGAACTTGGTTGTAAAGGGCCCGAAACCTTTAACGCATGTGCAACGGTTAAATGGAATC
AAGGCACGAGTTTTCCTATCGAATCTGGCCATCCGTGTCTTGGTTGCTCTGAGCCCGATTTCTGGGATAAAAGCAGCTTTTACCAAGCCTTGGGTCCATGGGAGTGGTACAAATCCAAACCCGGCAAAGGTGCACAGAAGCATGCTGGGAAAAACTCATAAGATAATAGG
CAAGGCTCATGGAAAATACAGCAAGTAACAACCGGTTAGTCGTCGATCCTATCACTCGAATTGAAGGGCATCTTCGAATAGAAGCTGAAATGGATGGGAATACCATCAAACAGGCGTTCTCATCAGGCACGTCTGTTCGGGGAATTGAACTGATTTTACAAGGCAGAGAT
CCGCGTGACGCTTGGGCTTTTGCGCAACGTATCTGTGGCGTCTGCACGCTGGTGCATGGTATGGCATCGGTGCGTGCTGTCGAAGATGCAATTAGAAAAGCTTGGCGGTCAAACGCAAAATTAGGGGTTGCCATTGGAAAGCCCTCCATGACATCTATGCCAAAAGGACC
GATGCAACATGGTAAAAAAGGGCACCGACAGTCACGTACTTCAATAGGCGTACTGAGTGAAGCAGAAATGGCTATCCCTCAAAATGCACAACTGATAAGAAACATCATGATTGCAACCCAATATGTGCATGATCACGTGATGCATTTTTATCACTTGCATGCCCTAGATT
GGGTTGACGTTGTTTCTGCACTAGATGCAGATCCAACGAGAACCGCTACGCTCGCCGGTCAATTGAGTGATTATCCTCGTTCATCGCCGGGATATTTCAAAGATGTGAAGCAAAAAGTCAAAACGCTGGTTGAGTCTGGGCAGCTAGGGATATTCAGTAACGCGTATTGG
GGGCATCCTGGCTATAAACTGCCACCTGAAGTTAACTTGATGGCATTAGCACATTATTTAGATGCGCTAACGTGGCAGCGTGAAGTTGTAAAAGTTCACACCATATTTGGAGGGAAAAACCCTCATCCTAATTTTGTCGTTGGCGGCGTGCCTTCACCGATTAATCTCAA
TGCGTCAACGGGTATTAACACGAGTCGATTAGTGCAACTACAAGATGCTATCACGCAAATGAAGAGCTTTGTCGATCAGGTGTATTACCCCGATATTGTGGCGATTGCGGGTTATTACAAAGAGTGGGGGACACGAGGGGAAGGGCTGGGTAACTTTCTTACCTATGGAG
ACTTACCTATGACATCAATGGATGACCCTGATTCTTTCTTGTTTCCACGAGGTGCAATACTTGGTCGAGACTTGAGTAAAGTGCATGACCTTGATCTAGATGATCCCTCTGAAATTCAAGAATTCGTCTCTTCCTCCTGGTATCGATATAGTGGAGGGAACGCAAGTGGT
TTACACCCTTTTAATGGACAAACAACACTCGAGTATACTGGTCCGAAACCGCCTTACAAGCACCTAAATGTAGGGGCTGAATATTCATGGTTGAAAAGTCCGCGTTGGAAAGGCCATGCGATGGAGGTGGGACCGCTGGCTCGCGTGCTAATGATGTATGCTAAAAAAGA
TGCCGCTGCGCAAGACATCGTTAATCGATCTCTTTCTATCTTGGATTTAGAGACCTCTGCACTTTTCTCCACACTCGGTAGGACGCTCGCCAGAGCAGTGGAAACAAAAATTGTGGTTAACCAGCTACAGTCTTGGTATGACCAACTATTGGATAATATCGCAAAGGGCG
ATACCGATACGTTTAACCCTCTGTATTTTGACCCTACCAATTGGCCAATTAAAGGCCAGGGGGTAGGCGTGATGGAAGCGCCTCGTGGCGCGTTGGGGCATTGGTTGGTCATGCAAAATGGCAAAATTGAGAATTACCAATGTGTCGTGCCTACGACATGGAACGCTGGA
CCTCGAGATCCCAACTCACAGGCAGGTGCTTATGAAGCCGCTCTGCAAGATAAACATACGCTACATGATCCTGACCAACCTTTAGAGATTTTGCGAACACTTCATAGTTTTGACCCCTGCTTAGCATGTGCCGTGCACGTAATGGACGAAACAGGGGAAGAGCGTTTGCG

TCTAAAAGTTCGTTAACCTTACTTGAGGAATATTATGAAAATCAAAAATAGTATTAGAAAGTCAGTTACTTCTTTAATTGCCTTTTTTTACCTGGTGGTAGGTTCACCCGTTAGTTATGCGCACCCGGGTCATCAAGGCGATCATTCACTACTTTTGGGATCGCTGGTGT
CAACAATCGTGCTTGTCACCGTTGCGCTGTTTATTTATCGAGCTAGAGCAAGTAGGCCCTATCAACGGGTAAAACAGTAATGTGTCTCGCTATCCCCATGAAAGTCATTGCAATTAAAGGCTTTAACGCAACGTGTGAAGCGAAAGGTGTCTCTCGCGAGGTGAGTTTAC
ATTTAGTGCAAGGGCTAGAGGTCAAAGTTGGCGACTATGTGATGGTACATGTTGGTTATGCTCTCCAAGTGATCACCTATGATGAAGCGCAGGTGACATGGGAGATGCTCGATCAGGTCATTGCTTACGATGCATGAAATTAGTCTTTGCTATAGCTTGCTTGACACAGT
CGCTGTTCACCAGCGAGCAAACATGAATAAGTCTGTCAGTCTGGTTCACGTCAAAGTCGGCCCACTGTCAGGTGTTGAGCCTGACTTGCTGCACCATGCGTTCTTGGCGTGTAGAACTCACACCATTGGCGATCAGGCAACGCTTAGAATTGACACAAGCGCTATCAAGA
TACGCTGTAAATTGTGTGGCGAATTCAGCCGAGTTTCGGTGAATAGCATCCTATGTGCAAGTTGCGGTGCTTGGCAAACCGACCTCATTGAGGGCGATGAATTTATATTGCAACGAATAGAGTTTATCGCACCTGACAATTTTACAGAATCAAAGGAGAGAAAACATGTG
CGGTAACTGTGGTTGCGAAACGCAAGTAAAATCAGAGAAAACTGACAAGCCATCAGCAAATATCGTTGAGGTTCACGCAAATTTAAAAAGCAACAATGACAACCAAGCGATAGCAAACCGTGCTTTATTTGACGCACATAATGTACTAGTGATTAATTTGATGTCATCCC
CAGGCAGTGGAAAAACCAGGCTACTCGAAGAAACGATACGTGCGCTTAAGTCGCAATACGCGATGGCGGTGATTGAAGGCGACCTCGAAACTGAAAATGACGCAAATAGAATTAGACGTCATGGTGTGCAGGCGGAGCAAATCGCCACGGGCCAAGGGTGTCACTTGGAC
GCCAGTATGGTGCAAAGGTACTTGGAAGATTTAGCTTACAAAAACTAGACGTTTTGTTTATAGAGAATGTTGGCAATTTGATTTGTCCAGCGTGTTTTGATTTAGGACAGCACTTAAACATTATTTTGCTGTCCGTACCAGAAGGTGATGATAAGCCTGAAAAGTACCC
AGTGATGTTTCGTGCCGCGGACGTCATGCTTATTTCTAAGACCGATTACTTGCAATTTCACGATGAATTCAATGTACCTCGCGCGATTGAATCATTTCGTAAAGTGGGCAACGATGCGCCCGTTCTCGAAGTGTCTTCATTGAAAAGTCAACATCTAGAGCGTTGGTTTT
CTTATTTTACGCACGCCATTACAGCTCATCAGTCTCAGTCGTTACAAAATAGCGGAGTCTAAAGTCTTATGTACAGTGTTGAATCGTTGCTAAGAGACATTAAGAATACCTCGCTTTCAAAACCGTTTCGTATTCTTAATGTTTGCGGTGGCCATGAGCGTGCGATTACG
CGCGCTGGATTTAGAACGTTGTTTCAGCACAATATTCATCTGATACCTGGGCCAGGGTGCCCCGTTTGTATTTGCCCCGAAGAGGATATTGCATACGCGATACACTTAGCCATTAACGAAAATGTCGTTATCGTTAGTTTTGGTGACATGCTCCGCGTCCCAGTCGAACA
CGAAATGGGTGGCTGTAGCTCATTGATAGACGCAAAAAATCAAGGGGCCGATATCCGTCCGATATCTTCTCCTCAAGAAGCGGTATCTATAGCATTGGAAGAACCTCGTAAGGTTATTGTGTTTTTTGCTGTTGGTTTTGAAACGACTATGGCACCGATTGCAGCAACAC
TGCTTAATGATTTACCCAATAACTTTAAAGTATTATTGTCTGGACGACTTACTTGGCCAGCAGTTGCCCATGTGCTTGAGAGTCAACCGAATACTTTTGATGCACTGATTGCGCCAGGCCACGTTGCGACAATTATGGGTAGTGAAGAATGGCAGTTTGCAATCGACCAT
CATAATTTGCCTGTCAGTATTGCTGGTTTTCATCCAGAAAGTTTGCTGTTGTCGCTCCAAACGCTGCTCGGTAATTGTTCAAATAAAGTCGTCACACTGAGTAACCGCTATCCCGAAGTGGTAAAGCAAAACGGCAATGCAGCAGCTAAAGCAATAATCAATAAAGCGTT
CACTATCGTTGACGCACATTGGCGAGGAATTGGTGTTATTCCTGGTTCTGGATTTTCCTTTGCTTCGAGACTATCGCACCTAGACGCCACAAACGATTATGCGCCAGTCGATTTCCCTTCGCAATGTGCGCAAAACGTTCCTGAAACCACATCGCCCTGTGAAAAAGTGA
TCCTCGGAAAAATGGCACCTGATGCGTGCCCGTTTTTTGGTCAGGAATGCAAACCAGCATCCCCTAAAGGCGCATGTATGGTGTCTGATGAGGGGGCTTGCCGAATTTGGTATAGCTCTGGTGAGCGCTCGATAACAAATGTTATCAAAAAGGGGAATACGCTAAAGGTG
GAGATGAAGTGATGACAACTGCCCGCACATATGTTGCCAATACGAATAAGAGTAAAACTATCGCCTATGAGTTGAACATTACTGGAGTCATCCAGGGGGTTGGGTTCAGACCGTTAGTTTACTGCTTAGCGTATGAAAAAAGTATTGTTGGTTGGGTACAAAACGACTGT
GGATGTGTGCGAATACACGCTGAAGGCACTGAGCTAGACGTTGAACAATTTGTATTCGACTTACTGAACAATGGGTCGCTAGTTTCCATATCCTTGCTGGAAAAAAAGCAGGTTAAACTGAGTAATGTAGACTCGTTCACGATAAAAGAAAGCGGTCATGATATAGCGTG
CGGGGCGATATCAATCCCCAAAGATGTCTATTTGTGTGGAGCGTGTAAAGCTGAGTTACTGTCAAATACTAACCGACGTGGCACCTATAGCTTTATCGCTTGCAGTAAATGTGGTCCACGATTTTCTATGCTGCGAGCAATGCCATACGACAGAAAGAACATGAGTATGT
CAGCATTTCCAATGTGTGAGTTATGTCGTAAAGAATATCAATCGCCAACTGATAGGCGCTTTCATGCTCAACCAATAAGCTGTCGCGTATGCGGGCCTGAAGTATTTTGTTCAACTGTCGGCGGGCGCGTGGTCTCGCAAGGCGATGTTGATGTTGTAGCAACGGTTGTC
GCGTATTTGAATCAAGGCAAAATTGTCGCGTTAAAAAGTATAGGCGGTTATCACTTGCTCTGTGACGCACAAAGCACTGAAGCGGTAGACCTTTTACGCCAGCGTAAAAATAGACCAGATAAACCTTTCGCGGTCATGCTCCCAGAGCCTCGTGCAGACGAAACAAGTGA
GGATTGGTTAGATAAATGCGTTGCTGTAGACAGTCATCAAAGAGCGCTCTTACTAAGCCCCGTGCGACCCATTTTACTTGCAAAAAAGCAATCGAATGTACCCATTGCAGATAACGTAGCGCCCATGCTTCGTGATTTGGGGGTGATGTTGCCGTGCAGCGGACTGCATT
TACTCATTATGCAGCAATTCAATCGCCCCATGGTGGCTACGTCAGGAAATATTTACGGTGAACCGATGGTCACATCGACGTCATCGGCACAGCAACACCTTTCGATGTTGGCAGACATCTTCGTGCACCATAATCGAGACATTTTTCACAAACTGGATGATTCTGTGCTG
CGAACGCTTAAGTCAGGCACCATTCCAATTCGCCTGGGGCGGGGTATAAGTCCGATAGAATTATCATTGCCATATACTGTGCAGGAACCAATGCTTGCTGTTGGCGCGAATCAGAAAAATACAGTCGCAATAGCTTTGAACAATCGACTTATCGTAACACCGCATATTGG
CGACCTCGATTCTCCGATAATGCAAGCGCACTTTGAAAAATGTATCAGTGACTTTCAAGCTCTCTATCATGTTCATCCACAGCGTATTCTTTGCGATAGCCATCCTGGCTATATCAGTTCCCGTTGGGCCAAAGGCAGTGAACTACCGACTACTAAAGTTTTACATCACC
ACGCTCATGCATCAGCGTGGGCATTAGCTGCTGAAATATCGACACCAAGCTTGGTTTTCGTGTGGGATGGAACGGGATTAGGCGAGAATGGCGAGATGTGGGGAGGGGAAGTTTTTTGTGGGACGCCGGGCAATTGGAAACGTGTTGCCTCATTAGCGCCTATGAAGTTG
CAAGGCGGCAACAAGGTTCCAAATCAACCATGGCGAAGTGCAGCATCACTAGTGTGGGGTACTAATTTCACCGGCATTGATTTTAAACCATTTGATCCACAACAATTAAGCTACAACGCATGGCAAAACGAACTCAATTGTCATGTTAGTAGTTCAATGGGAAGACTGTT
CGACGCGGCGGCATTTGCCTGCTGTGAACAGAGCGAAGTCTCTTATGAAGGGCAGGCGCCGATGATGTTGGAAAGCGTTGCGTCGACGCCCTCTAAGGTTATGCCGTTAACGTTGGTCCAGAAAAACGACGAATTTATTCAAATGGATTGGCGAACACTGGTACCGGAAC
TATTTGATTTCAAAGTGTCTCCTCAAACTCGTGCTGCAAATTTCCACGCTTCGTTAATTGAAACGGTATGGGAGGTAACCCTACATTGCCGTAAAAAGTTTAATTTTGACCACATTGGATTGGCAGGAGGCGTCTTCCAAAATCGCATTTTGTGTGACGGTATATCACAA
CGGTTTTTACAATCAGACATTCCTGTGGTTATTCCAAGTAGTTTGCCATTAAACGATGCCGCAATAAGTGTTGGGCAAATCCACGAATACTGTTCACGGAGGTCATAATGAAAAGCGCAGAGGGCAATAATCATTCATCATCAGAGCCGTTGGGCACGGAATCTATTCAA
TTAGCTCAGGGGAACGGCGGAACACTGACAAAAAGACTCATTGATCATGTTTTTAAAAATAAAGCGAACAAAGATCTAGATCTGCTTCATGATGCCGCCACAGTAACTTTTGATGGTCCTTATCTGAGTATAACAACGGATAGCTTCGTTGTATCCCCCCCAACATTTCC
AGGCGGCAATGTAGGCGCGTTGAGTGTTTACGGTACCGTCAATGATTTAGCAGTCGTTGGGGCAACACCACGGTACATCAGTACTGCATTTATTATTGAAGAAGGCTTTTCCCTTTCAACATTGAAACAAATCGTTAATGGAATGCATCAAGCTGCCGAAGAAACGAAGG
TCGCCATTGTAACAGGTGATACGAAAGTTGTCCCGAAAGGCAGTGGTGGCGGCGTTTTTATCAATACAACGGGTGTTGGAGACAGCCGAGATGCGCCGATTTGGGATACCAGTCTTATAAAGGCAGGCGATCATGTACTGGTTAGTGGTTCTGTAGGTGATCACGGTGCG
TGTGTCCTGCTTGCTCGCGAGGACTATGGTTTGCAAGGTCAGCTAAAGTCTGATTGTGGAAGCGTTGTTCCTCTTATTGATCCAATCAAGCATTTGGAAGGTGTTCGTTTTGTGCGTGACCCAACTCGTGGAGGGTTGTCAGTATTACTCCATGACGTTGCTAATGAAAC
AGGCTTCGATATCGAGTTAATTGAAAATAATATTCCGGTGCGACCTGAAGTGGCAAGCGTGTGTGAAATATTAGGCTTTGATCCGTTTGTTTTAGCGTGTGAGGGACGCATTGTTGCTGTTGTTGCCCCTGACATAAGCGGTAAGGTGCTAGAACACTGGCGGAGCATTC
GAAACGGGGAGCAGGCCGAACACATTGGTGTAATAGTAAAAAACACCGAATCAAAAGGGCGCGTGACCATTGTTACGCCAATGGGAGGAAGACGTTTCATGAATGAACTCGAAGATGAGCCGCTTCCAAGGATCTGCTGAGATCCTGGCCTCTAAACGGGTCTTGAGGGG
TTTTTTGCTGAAAGGAGGAACTATATCCGGATATCCACAGGACGGGTGTGGTCGCCATGATCGCGTAGTCGATAGTGGCTCCAAGTAGCGAAGCGAGCAGGACTGGGCGGCGGCCAAAGCGGTCGGACAGTGCTCCGAGCGAGCTTCTGGAGCAGGAAGATGTCGCGGGC
ATTAGCACCAGCGGTCTGCCAAGCCTCCGCCAGCCGTTGGGTCCCTTCCGCTTGAGCTTTTCCATCTTCGACGATACGGGCGGCGGCCCCCCGCGCTTCCGCGATCGCCCGTTTACAAGCTGCCTCAGCTGGGGCGATCACATCGGCTTGAAGTTGCTGCTGCACCTGTT
TGATCCGCTCCTGCTGCACAGGGAGTTCTGCTTGGCTACGAGCGACTTCGGTAGCAATGTCCGCTTCAGCTTCGGCCACCACCGCTTCGCGCCGCGTCAACGCATCCTGAATCCGGCGCTCGGCCTCGGCTTGGGCGATCGCTACATCGCGATCGATCCGACGCAGGGCC
GTGATCTTGTCATTTTCGGCCGTTTGGATCGCAGAGGCAGCCTGGGCATCGGCTTCAGCAATTCGGGCATCTCGCTGCAGATCAGCCCGCTGCTTGCGTCCACTAGCCGAGAGATAACCGACCTCATCGGAAATGTTCTGGACTTGCAGCGTATCGAGGACTAGACCCAG
CTGCTCAAGGTCATCCTCCGCCTCTTCCAGCAGACTTTTGGCAAAGGCAATTTTGTCCTCGTTGATCTGCTCCGGCGTGAGGCTGGCTAAAACACCACGCAAGTTGCCTTCGAGGGTCTCCTTGGCAATTTGCTCGATTTCCTTACGGTTTTTGCCAAGCAGCCGCTCGA
TCGCGTTGTGGATGGTCGGTTCTTCCCCAGCAATCTTGATATTGGCAACGCCTTCAACAGTCAGGGGAATGCCGCCCTTGGAGAAGGCATTGGAAACGCGCAACTCAATGATCATGTTGGTCAGATCCATGCGGAGCGCTTTTTCCAGCAGAGGTACCCGCAGGCTGCTG
CCGCCCTTGACCAAGCGATAGCCAACTCGGCGGCCATCACTACTGCGGCGACTACTGCCAGCAAAGATCAAAATTTCACTGGGTTGGCAGATGTAGTAGAGATTGCGCAGGACTAAGCTGCCAGCCCCGGCGACTCACCAGTCACAGAAAAGCATCTTACGGATGGCATG
ACAGTAAGAGAATTATGCAGTGCTGCCATAACCATGAGTGATAACACTGCGGCCAACTTACTTCTGACAACGATCGGAGGACCGAAGGAGCTAACCGCTTTTTTGCACAACATGGGGGATCATGTAACTCGCCTTGATCGTTGGGAACCGGAGCTGAATGAAGCCATACC
AAACGACGAGCGTGACACCACGATGCCTGCAGCAATGGCAACAACGTTGCGCAAACTATTAACTGGCGAACTACTTACTCTAGCTTCCCGGCAACAATTAATAGACTGGATGGAGGCGGATAAAGTTGCAGGACCACTTCTGCGCTCGGCCCTTCCGGCTGGCTGGTTTA
TTGCTGATAAATCTGGAGCCGGTGAGCGTGGGTCTCGCGGTATCATTGCAGCACTGGGGCCAGATGGTAAGCCCTCCCGTATCGTAGTTATCTACACGACGGGGAGTCAGGCAACTATGGATGAACGAAATAGACAGATCGCTGAGATAGGTGCCTCACTGATTAAGCAT
TGGTAACTGTCAGACCAAGTTTACTCATATATACTTTAGATTGATTTAAAACTTCATTTTTAATTTAAAAGGATCTAGGTGAAGATCCTTTTTGATAATCTCATGACCAAAATCCCTTAACGTGAGTTTTCGTTCCACTGAGCGTCAGACCCCGTAGAAAAGATCAAAGG
ATCTTC

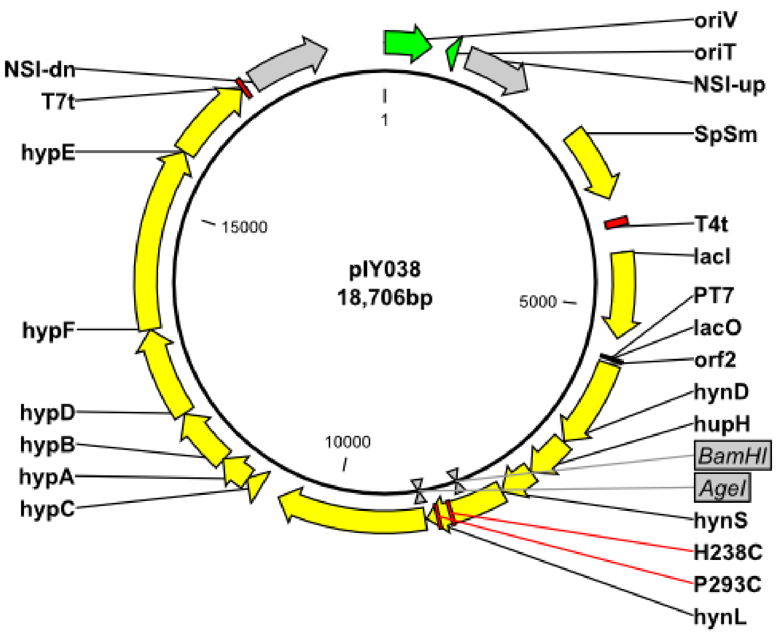

TTGAGATCCTTTTTTTCTGCGCGTAATCTGCTGCTTGCAAACAAAAAAACCACCGCTACCAGCGGTGGTTTGTTTGCCGGATCAAGAGCTACCAACTCTTTTTCCGAAGGTAACTGGCTTCAGCAGAGCGCAGATACCAAATACTGTCCTTCTAGTGTAGCCGTAGTTAG
GCCACCACTTCAAGAACTCTGTAGCACCGCCTACATACCTCGCTCTGCTAATCCTGTTACCAGTGGCTGCTGCCAGTGGCGATAAGTCGTGTCTTACCGGGTTGGACTCAAGACGATAGTTACCGGATAAGGCGCAGCGGTCGGGCTGAACGGGGGGTTCGTGCACACAG
CCCAGCTTGGAGCGAACGACCTACACCGAACTGAGATACCTACAGCGTGAGCTATGAGAAAGCGCCACGCTTCCCGAAGGGAGAAAGGCGGACAGGTATCCGGTAAGCGGCAGGGTCGGAACAGGAGAGCGCACGAGGGAGCTTCCAGGGGGAAACGCCTGGTATCTTTA
TAGTCCTGTCGGGTTTCGCCACCTCTGACTTGAGCGTCGATTTTTGTGATGCTCGTCAGGGGGGCGGAGCCTATGGAAAAACGCCAGCAACGCGGCCTTTTTACGGTTCCTGGCCTTTTGCTGGCCTTTTGCTCACATGTTCTTTCCTGCGTTATCCCCTGATTCTGTGG
ATAACCGTATTACCGCCTTTGAGTGAGCTGATACCGCTCGCCGCAGCCGAACGACCGAGCGCAGCGAGTCAGTGAGCGAGGAAGCGGAAGAGCGCCTGATGCGGTATTTTCTCCTTACGCATCTGTGCGGTATTTCACACCGCATATGGTGCACTCTCAGTACAATCTGC
TCTGATGCCGCATAGTTAAGCCAGTATACACTCCGCTATCGCTACGTGACTGGGTCATGGCTGCGCCCCGACACCCGCCAACACCCGCTGACGCGCCCTGACGGGCTTGTCTGCTCCCGGCATCCGCTTACAGACAAGCTGTGACCGTCTCCGGGAGCTGCATGTGTCAG
AGGTTTTCACCGTCATCACCGAAACGCGCGAGGCAGGATATCCGGCAGCCGGCGGAGCGCTGCTTTCTTGGCAAGCGGTCGCCAGCCCCAACGCCAGGGCTGCCAGCCCGAAACAGCGGGGCAAGGCAGCTTGGAAGGGCGATCGCAGCACGGGCATGGCAATGTCTCTC
TGAAGGAATGCAGACCTTATTCGTACAGCCAGGGTTGAATCGTGGGGGTCCAATCACTTAGCTCTGCTGGGCTAAACCAGAGAGCAATTTCCTGTTGTGCTGTTTCGATTGCATCCGAGCCATGGATGATGTTGCGGCCAATATTGACACCAAAATCACCACGGATGGTG
CCCGGTTCTGCCGTCAGCGGATTGGTAGCGCCGATCAACTTGCGAGCAGCCGCCACAACGCCTTCGCCTTCCAAGACGATCGCCACGATCGGCCCAGAGGTGATGAACTCGACGAGGCCATTGAAGAAGGGGCGCTCGCGGTGGACAGCATAGTGCTGTTCGGCCAGCTC
GCGACTGGGCTTCAGCTGCTTTAGGCCCACCAGTTTGAAGCCTTTTTGCTCAAAGCGGCCGATGATCGTACCGACCAAACCCCGCTGAACGCCATCGGGCTTGATGGCAATAAATGTGCGTTCCACAGACATCTAGATAGTCCTCAAGACGAGGCAAGCATTGAGCTTGC
CTTCCTATGGTTCGGGATCACTGGGATTCTTGACAAGCGATCGCGGTCACATCGCTATCTCTTAGGACTTCGCAGCGGGCGAGTCGGATTGACCCGGTAGGGATTTCGCCAGATCAATGCCCGTGGTTTGTTTCAGCTTCTCCAGCAAGCTAGCGATTTGGGTAGCGCTG
CCTTCCCCTTCGCCAATCACAGTGATCGACTCCACGTCGATATCTGGCACGGTGCCTGAAAGCGTGACGAGCAGGGACTCGAAGCTTGCATGCCTGCAGGTCGACTCTAGAGCTTTATGCTTGTAAACCGTTTTGTGAAAAAATTTTTAAAATAAAAAAGGGGACCTCTA
GGGTCCCCAATTAATTAGTAATATAATCTATTAAAGGTCATTCAAAAGGTCATCCACCGGATCAATTCCCCTGCTCGCGCAGGCTGGGTGCCAAGCTCTCGGGTAACATCAAGGCCCGATCCTTGGAGCCCTTGCCCTCCCGCACGATGATCGTGCCGTGATCGAAATCC
AGATCCTTGACCCGCAGTTGCAAACCCTCACTGATCCGCATGCCCGTTCCATACAGAAGCTGGGCGAACAAACGATGCTCGCCTTCCAGAAAACCGAGGATGCGAACCACTTCATCCGGGGTCAGCACCACCGGCAAGCGCCGCGACGGCCGAGGTCTTCCGATCTCCTG
AAGCCAGGGCAGATCCGTGCACAGCACCTTGCCGTAGAAGAACAGCAAGGCCGCCAATGCCTGACGATGCGTGGAGACCGAAACCTTGCGCTCGTTCGCCAGCCAGGACAGAAATGCCTCGACTTCGCTGCTGCCCAAGGTTGCCGGGTGACGCACACCGTGGAAACGGA
TGAAGGCACGAACCCAGTGGACATAAGCCTGTTCGGTTCGTAAGCTGTAATGCAAGTAGCGTATGCGCTCACGCAACTGGTCCAGAACCTTGACCGAACGCAGCGGTGGTAACGGCGCAGTGGCGGTTTTCATGGCTTGTTATGACTGTTTTTTTGGGGTACAGTCTATG
CCTCGGGCATCCAAGCAGCAAGCGCGTTACGCCGTGGGTCGATGTTTGATGTTATGGAGCAGCAACGATGTTACGCAGCAGGGCAGTCGCCCTAAAACAAAGTTAAACATCATGAGGGAAGCGGTGATCGCCGAAGTATCGACTCAACTATCAGAGGTAGTTGGCGTCAT
CGAGCGCCATCTCGAACCGACGTTGCTGGCCGTACATTTGTACGGCTCCGCAGTGGATGGCGGCCTGAAGCCACACAGTGATATTGATTTGCTGGTTACGGTGACCGTAAGGCTTGATGAAACAACGCGGCGAGCTTTGATCAACGACTTTTGGAAACTTCGGCTTCCCC
TGGAGAGAGCGAGATTCTCCGCGCTGTAGAAGTCACCATTGTTGTGCACGACGACATCATTCCGTGGCGTTATCCAGCTAAGCGCGAACTGCAATTTGGAGAATGGCAGCGCAATGACATTCTTGCAGGTATCTTCGAGCCAGCCACGATCGACATTGATCTGGCTATCT
TGCTGACAAAAGCAAGAGAACATAGCGTTGCCTTGGTAGGTCCAGCGGCGGAGGAACTCTTTGATCCGGTTCCTGAACAGGATCTATTTGAGGCGCTAAATGAAACCTTAACGCTATGGAACTCGCCGCCCGACTGGGCTGGCGATGAGCGAAATGTAGTGCTTACGTTG
TCCCGCATTTGGTACAGCGCAGTAACCGGCAAAATCGCGCCGAAGGATGTCGCTGCCGACTGGGCAATGGAGCGCCTGCCGGCCCAGTATCAGCCCGTCATACTTGAAGCTAGACAGGCTTATCTTGGACAAGAAGAAGATCGCTTGGCCTCGCGCGCAGATCAGTTGGA
AGAATTTGTCCACTACGTGAAAGGCGAGATCACCAAGGTAGTCGGCAAATAATGTCTAACAATTCGTTCAAGCCGACGCCGCTTCGCGGCGCGGCTTAACTCAAGCGTTAGATGCACTAAGCACATAATTGCTCACAGCCAAACTATCAGGTCAAGTCTGCTTTTATTAT
TTTTAAGCGTGCATAATAAGCCCTACACAAATTGGGAGATATATCATGAAAGGCTGGCTTTTTCTTGTTATCGCAATAGTTGGCGAAGTAATCGCAACATCCGCATTAAAATCTAGCGAGGGCTTTACTAAGCTGATCCGGTGGATGACCTTTTGAATGACCTTTAATAG
ATTATATTACTAATTAATTGGGGACCCTAGAGGTCCCCTTTTTTATTTTAAAAATTTTTTCACAAAACGGTTTACAAGCATACTAGAGGATCGGCGGCCGCGGATCTGGGCCGCGGGTCATGGCTGCGCCCCGACACCCGCCAACACCCGCTGACGCGCCCTGACGGGCT
TGTCTGCTCCCGGCATCCGCTTACAGACAAGCTGTGACCGTCTCCGGGAGCTGCATGTGTCAGAGGTTTTCACCGTCATCACCGAAACGCGCGAGGCAGCAGATCAATTCGCGCGCGAAGGCGAAGCGGCATGCATTTACGTTGACACCATCGAATGGTGCAAAACCTTT
CGCGGTATGGCATGATAGCGCCCGGAAGAGAGTCAATTCAGGGTGGTGAATGTGAAACCAGTAACGTTATACGATGTCGCAGAGTATGCCGGTGTCTCTTATCAGACCGTTTCCCGCGTGGTGAACCAGGCCAGCCACGTTTCTGCGAAAACGCGGGAAAAAGTGGAAGC
GGCGATGGCGGAGCTGAATTACATTCCCAACCGCGTGGCACAACAACTGGCGGGCAAACAGTCGTTGCTGATTGGCGTTGCCACCTCCAGTCTGGCCCTGCACGCGCCGTCGCAAATTGTCGCGGCGATTAAATCTCGCGCCGATCAACTGGGTGCCAGCGTGGTGGTGT
CGATGGTAGAACGAAGCGGCGTCGAAGCCTGTAAAGCGGCGGTGCACAATCTTCTCGCGCAACGCGTCAGTGGGCTGATCATTAACTATCCGCTGGATGACCAGGATGCCATTGCTGTGGAAGCTGCCTGCACTAATGTTCCGGCGTTATTTCTTGATGTCTCTGACCAG
ACACCCATCAACAGTATTATTTTCTCCCATGAAGACGGTACGCGACTGGGCGTGGAGCATCTGGTCGCATTGGGTCACCAGCAAATCGCGCTGTTAGCGGGCCCATTAAGTTCTGTCTCGGCGCGTCTGCGTCTGGCTGGCTGGCATAAATATCTCACTCGCAATCAAAT
TCAGCCGATAGCGGAACGGGAAGGCGACTGGAGTGCCATGTCCGGTTTTCAACAAACCATGCAAATGCTGAATGAGGGCATCGTTCCCACTGCGATGCTGGTTGCCAACGATCAGATGGCGCTGGGCGCAATGCGCGCCATTACCGAGTCCGGGCTGCGCGTTGGTGCGG
ATATCTCGGTAGTGGGATACGACGATACCGAAGACAGCTCATGTTATATCCCGCCGTTAACCACCATCAAACAGGATTTTCGCCTGCTGGGGCAAACCAGCGTGGACCGCTTGCTGCAACTCTCTCAGGGCCAGGCGGTGAAGGGCAATCAGCTGTTGCCCGTCTCACTG
GTGAAAAGAAAAACCACCCTGGCGCCCAATACGCAAACCGCCTCTCCCCGCGCGTTGGCCGATTCATTAATGCAGCTGGCACGACAGGTTTCCCGACTGGAAAGCGGGCAGTGAGCGCAACGCAATTAATGTAAGTTAGCGCGAATTGATCTGGTTTGACAGCTTATCAT
CGACTGCACGGTGCACCAATGCTTCTGGCGTCAGGCAGCCATCGGAAGCTGTGGTATGGCTGTGCAGGTCGTAAATCACTGCATAATTCGTGTCGCTCAAGGCGCACTCCCGTTCTGGATAATGTTTTTTGCGCCGACATCATAACGGTTCTGGCAAATATTCTGAAATG
AGCTGCCCGCGAAATTAATACGACTCACTATAGGggaattgtgagcggataacaattcccctctagaaataattttgtttaactttaagaaggagatataccATGGCTTCCATTATTGGTAGCGGCTTTTTGATCATTGCACCACTGCTGCATTCGGTAATGGGGAAATG
GGCTCTGCTTGGAATAGTGTTGTTGTCCATTCTGGCATATGCGCTGGGGTCGGTTATTCGCTTCAATATACGCCATGCAGAACCTGAGCTAGCTAGCAATCAACAAAGCAGTATTGTGACGCTTGAAAAAGCAAGTCAATGGGCGCTTGGTGCGGCATATGCCATTTCCG
TGGCGTTTTATATTAGTTTGTTTGCAGCGTTTGTTTTTGATCGTTTATCAATCTCTGATACGACGTACATCAAACTGTTTACCAGTGGGTTGCTTGTCATCATTACGGTGGTCGCTTGGCTCCGTGGTGCTAGGGGGCTAGAGACCATTGAGCTATTCGCCGTGACAATA
AAGTTGGCTATTATAGTAGGTGTGCTCGCAGCGCTGGCAACCTACGACATACAAGTACAAAGTGCTTGGTTTCAACATGAAGCGATACAGGCATTGAGCCATTTTGAAACTGTTAGCATGCTCGCAGGCATGCTCATGGTAACGCAAGGGTTCGAGACAACCCGTTTTAT
GGGGAATAACTACACGCCAGAACAGCGCATTAAAGCGAGTCGTTATGCTCAGTGGATCGCTATTTTTCTCTATGTTGTTTTTATCGGTCTAACGTGTCCTATTTTTCTGGATTTTCCGATCACGGAATTAAACGAAACAACGATTAGCTACACCCTAGGGCAAGCAATAT
GGGTATTGCCGATTTTATTACTGGTTGCCGCCACGGCAAGTCAATTAAGTGCAGCATTAGCCGATACTATCGGTGGCGGAGGCCTCTTAAAAGAGCTTTTCCATTTACCTATCTCCCCGCAATTTTACTACGTTTTAGTCATCGCCATCGCAGGTATATTAGTTTGGTCA
TCCAACGTATTTGAAATTATTAATCTCGCTTCAAAAGGGTTTGCACTTTATTACCTGATCCAAACAATAATCGCGGTCAAACTCGTGATGCGTCAAAGCACAGGTCAACGCATTAGGTCAACAAAATTACTTGGTCTATCGGTGATCGTTCTCTGTTTATTCTTTGTGAT
TGGCTGGTCAATTCCAGCACCTCATAGCTAATAAGGAAAATACCGATGCTATCAGTAGAAACTACACCACCAATTGACCAAATCAAAAACGCGGCTAAGCGCTACGCTATCATTGGTGTTGGAAATCTATTGCAGCGAGATGATGGCGTTGGTGTGCACGCGGTTCGTGC
TATGCAACCCCTGCTAAAGTCTTATTCAAATGTATCTTGCATTGACGCTGGGACACTGAGCTATGAACTTTTGGAGTGGGTTGCGAGTTCCGATCATACGATAGTCATCGACGCGGCTTATATGCGCTGTCCGCCTGGCACGGTGAGAGTGTTTGAGAACGAGGCAATTG
CGGATCAGTTTCAGCAAGCGACGACCCATTCCGTTCACCAGATCACGTTACGCGACACATTGTTGACCAGCCAAACGTTATATTCAAAACCTTCTGCGGTGACGTTAATTGGCGTAGAGCCGGAGGCGATTGAGTGGGGAACCTCTTTGAGTCCAAAGGTAAATGCAGCG
CTATCATCGATCATGGATATCGCATTTGCTTGCTTATCAGGCGACAACGTTCAGACCTATAAACCGGCAACCCAAAAGGAGGCACGATGACCGAAGAACTCATTTTTATGACATCAAATGCTGATAAAACGGGCAACGTAATGCCGCTTTTGCATCAGATACGTCATGCG
TTGTCTCAGTTAATTGAACGACAAGAACAAACCACGATTGATCTGCGACGATTGCCTTTAAGCGCTAGCGAAGAAGCGCAGTTAGAAGCTTTTCTTGGGCATGGAGAGGTTAAAGCTGATATTCAGGCGCTGGGTGATACGGTGCTAATCGAATCACGCTACGCAGGCGT
TTGGCTTGAAATTCACTATAACGAAGATGTGGAGATCATGGGCAAATACGTTCATATCTGTACCTGTCCACCAATCATAAAATCTCAGCCAGAAGATATGGTGTTATCGCTCAGCAATATAGTGTCTGACATCCATTCGTTGTCTCATCAATCTTCTGATGAAACGGCTA
AGGAGGATTGAAATGGCGCTACCAACATTAAACAAGCAGTTACAAGCGTCCGGTATCTCAAGACGCACATTTCTTAAGTTTTGCGCTACAACGGCGTCTTTACTGGCATTGCCGCAAAGCGCTGTTGCCGATTTGGCGACTGCCCTTGGCAATGCGAGAAGACCCTCTGT
GATTTGGTTGCCCTTTCAAGAGTGCACAGGGTGTACCGAAGCAATATTGCGCTCTCATGCTCCCACATTGGAAAGCCTTATTTCGATCATATTTCGTTGGATTATCAGCATACGATAATGGCTGCTGCGGGAGAGCAAGCTGAAGACGCTAGGCGTGCGGCGATGAACG
CGCACAAAGGGCAATATTTGTTGTTGGTTGATGGTTCGGTTCCGGTGGGTAACCCAGGATACTCAACGATCAGTGGCATGAGTAATGTCGATATGCTGAGAGAATCGGCAAAAGATGCTGCGGGTATTATTGCCATCGGTACCTGCGCGTCTTTTGGCGGGATCCCTAAA
GCAAACCCAAATCCGACGGGGGCAGTGGCAGTAAGCGACATTATTACAGACAAGCCAATCGTAAACATTTCAGGCTGTCCGCCACTGCCTATCGCGATTACAGCTGTGTTGGTTCATTACCTGACGTTTAAGCGTTTCCCTGATCTCGACGAATTACAACGCCCACTCGC
TTTTTTTGGTGAAAGCATCTGTGACAGATGTTATCGACGCCCATTTTTTGAACAACGTAAATTTGCAAAATCGTTTGATGATGAGGGGGCAAAAAATGGGTGGTGTTTGTTCGAACTTGGTTGTAAAGGGCCCGAAACCTTTAACGCATGTGCAACGGTTAAATGGAATC
AAGGCACGAGTTTTTGTATCGAATCTGGCCATCCGTGTCTTGGTTGCTCTGAGCCCGATTTCTGGGATAAAAGCAGCTTTTACCAAGCCTTGGGTCCATGGGAGTGGTACAAATCCAAACCCGGCAAAGGTGCACAGAAGCATGCTGGGAAAAACTCATAAGATAATAGG
CAAGGCTCATGGAAAATACAGCAAGTAACAACCGGTTAGTCGTCGATCCTATCACTCGAATTGAAGGGCATCTTCGAATAGAAGCTGAAATGGATGGGAATACCATCAAACAGGCGTTCTCATCAGGCACGTCTGTTCGGGGAATTGAACTGATTTTACAAGGCAGAGAT
CCGCGTGACGCTTGGGCTTTTGCGCAACGTATCTGTGGCGTCTGCACGCTGGTGCATGGTATGGCATCGGTGCGTGCTGTCGAAGATGCAATTAGAAAAGCTTGGCGGTCAAACGCAAAATTAGGGGTTGCCATTGGAAAGCCCTCCATGACATCTATGCCAAAAGGACC
GATGCAACATGGTAAAAAAGGGCACCGACAGTCACGTACTTCAATAGGCGTACTGAGTGAAGCAGAAATGGCTATCCCTCAAAATGCACAACTGATAAGAAACATCATGATTGCAACCCAATATGTGCATGATCACGTGATGCATTTTTATCACTTGCATGCCCTAGATT
GGGTTGACGTTGTTTCTGCACTAGATGCAGATCCAACGAGAACCGCTACGCTCGCCGGTCAATTGAGTGATTATCCTCGTTCATCGCCGGGATATTTCAAAGATGTGAAGCAAAAAGTCAAAACGCTGGTTGAGTCTGGGCAGCTAGGGATATTCAGTAACGCGTATTGG
GGGCATCCTGGCTATAAACTGCCACCTGAAGTTAACTTGATGGCATTAGCACATTATTTAGATGCGCTAACGTGGCAGCGTGAAGTTGTAAAAGTTCACACCATATTTGGAGGGAAAAACCCTCATCCTAATTTTGTCGTTGGCGGCGTGCCTTCACCGATTAATCTCAA
TGCGTCAACGGGTATTAACACGAGTCGATTAGTGCAACTACAAGATGCTATCACGCAAATGAAGAGCTTTGTCGATCAGGTGTATTACCCCGATATTGTGGCGATTGCGGGTTATTACAAAGAGTGGGGGACACGAGGGGAAGGGCTGGGTAACTTTCTTACCTATGGAG

ACTTACCTATGACATCAATGGATGACCCTGATTCTTTCTTGTTTCCACGAGGTGCAATACTTGGTCGAGACTTGAGTAAAGTGCATGACCTTGATCTAGATGATCCCTCTGAAATTCAAGAATTCGTCTCTTCCTCCTGGTATCGATATAGTGGAGGGAACGCAAGTGGT
TTACACCCTTTTAATGGACAAACAACACTCGAGTATACTGGTCCGAAACCGCCTTACAAGCACCTAAATGTAGGGGCTGAATATTCATGGTTGAAAAGTCCGCGTTGGAAAGGCCATGCGATGGAGGTGGGACCGCTGGCTCGCGTGCTAATGATGTATGCTAAAAAAGA
TGCCGCTGCGCAAGACATCGTTAATCGATCTCTTTCTATCTTGGATTTAGAGACCTCTGCACTTTTCTCCACACTCGGTAGGACGCTCGCCAGAGCAGTGGAAACAAAAATTGTGGTTAACCAGCTACAGTCTTGGTATGACCAACTATTGGATAATATCGCAAAGGGCG
ATACCGATACGTTTAACCCTCTGTATTTTGACCCTACCAATTGGCCAATTAAAGGCCAGGGGGTAGGCGTGATGGAAGCGCCTCGTGGCGCGTTGGGGCATTGGTTGGTCATGCAAAATGGCAAAATTGAGAATTACCAATGTGTCGTGCCTACGACATGGAACGCTGGA
CCTCGAGATCCCAACTCACAGGCAGGTGCTTATGAAGCCGCTCTGCAAGATAAACATACGCTACATGATCCTGACCAACCTTTAGAGATTTTGCGAACACTTCATAGTTTTGACCCCTGCTTAGCATGTGCCGTGCACGTAATGGACGAAACAGGGGAAGAGCGTTTGCG
TCTAAAAGTTCGTTAACCTTACTTGAGGAATATTATGAAAATCAAAAATAGTATTAGAAAGTCAGTTACTTCTTTAATTGCCTTTTTTTACCTGGTGGTAGGTTCACCCGTTAGTTATGCGCACCCGGGTCATCAAGGCGATCATTCACTACTTTTGGGATCGCTGGTGT
CAACAATCGTGCTTGTCACCGTTGCGCTGTTTATTTATCGAGCTAGAGCAAGTAGGCCCTATCAACGGGTAAAACAGTAATGTGTCTCGCTATCCCCATGAAAGTCATTGCAATTAAAGGCTTTAACGCAACGTGTGAAGCGAAAGGTGTCTCTCGCGAGGTGAGTTTAC
ATTTAGTGCAAGGGCTAGAGGTCAAAGTTGGCGACTATGTGATGGTACATGTTGGTTATGCTCTCCAAGTGATCACCTATGATGAAGCGCAGGTGACATGGGAGATGCTCGATCAGGTCATTGCTTACGATGCATGAAATTAGTCTTTGCTATAGCTTGCTTGACACAGT
CGCTGTTCACCAGCGAGCAAACATGAATAAGTCTGTCAGTCTGGTTCACGTCAAAGTCGGCCCACTGTCAGGTGTTGAGCCTGACTTGCTGCACCATGCGTTCTTGGCGTGTAGAACTCACACCATTGGCGATCAGGCAACGCTTAGAATTGACACAAGCGCTATCAAGA
TACGCTGTAAATTGTGTGGCGAATTCAGCCGAGTTTCGGTGAATAGCATCCTATGTGCAAGTTGCGGTGCTTGGCAAACCGACCTCATTGAGGGCGATGAATTTATATTGCAACGAATAGAGTTTATCGCACCTGACAATTTTACAGAATCAAAGGAGAGAAAACATGTG
CGGTAACTGTGGTTGCGAAACGCAAGTAAAATCAGAGAAAACTGACAAGCCATCAGCAAATATCGTTGAGGTTCACGCAAATTTAAAAAGCAACAATGACAACCAAGCGATAGCAAACCGTGCTTTATTTGACGCACATAATGTACTAGTGATTAATTTGATGTCATCCC
CAGGCAGTGGAAAAACCAGGCTACTCGAAGAAACGATACGTGCGCTTAAGTCGCAATACGCGATGGCGGTGATTGAAGGCGACCTCGAAACTGAAAATGACGCAAATAGAATTAGACGTCATGGTGTGCAGGCGGAGCAAATCGCCACGGGCCAAGGGTGTCACTTGGAC
GCCAGTATGGTGCAAAGGTACTTGGAAGATTTAGCTTACAAAAACTAGACGTTTTGTTTATAGAGAATGTTGGCAATTTGATTTGTCCAGCGTGTTTTGATTTAGGACAGCACTTAAACATTATTTTGCTGTCCGTACCAGAAGGTGATGATAAGCCTGAAAAGTACCC
AGTGATGTTTCGTGCCGCGGACGTCATGCTTATTTCTAAGACCGATTACTTGCAATTTCACGATGAATTCAATGTACCTCGCGCGATTGAATCATTTCGTAAAGTGGGCAACGATGCGCCCGTTCTCGAAGTGTCTTCATTGAAAAGTCAACATCTAGAGCGTTGGTTTT
CTTATTTTACGCACGCCATTACAGCTCATCAGTCTCAGTCGTTACAAAATAGCGGAGTCTAAAGTCTTATGTACAGTGTTGAATCGTTGCTAAGAGACATTAAGAATACCTCGCTTTCAAAACCGTTTCGTATTCTTAATGTTTGCGGTGGCCATGAGCGTGCGATTACG
CGCGCTGGATTTAGAACGTTGTTTCAGCACAATATTCATCTGATACCTGGGCCAGGGTGCCCCGTTTGTATTTGCCCCGAAGAGGATATTGCATACGCGATACACTTAGCCATTAACGAAAATGTCGTTATCGTTAGTTTTGGTGACATGCTCCGCGTCCCAGTCGAACA
CGAAATGGGTGGCTGTAGCTCATTGATAGACGCAAAAAATCAAGGGGCCGATATCCGTCCGATATCTTCTCCTCAAGAAGCGGTATCTATAGCATTGGAAGAACCTCGTAAGGTTATTGTGTTTTTTGCTGTTGGTTTTGAAACGACTATGGCACCGATTGCAGCAACAC
TGCTTAATGATTTACCCAATAACTTTAAAGTATTATTGTCTGGACGACTTACTTGGCCAGCAGTTGCCCATGTGCTTGAGAGTCAACCGAATACTTTTGATGCACTGATTGCGCCAGGCCACGTTGCGACAATTATGGGTAGTGAAGAATGGCAGTTTGCAATCGACCAT
CATAATTTGCCTGTCAGTATTGCTGGTTTTCATCCAGAAAGTTTGCTGTTGTCGCTCCAAACGCTGCTCGGTAATTGTTCAAATAAAGTCGTCACACTGAGTAACCGCTATCCCGAAGTGGTAAAGCAAAACGGCAATGCAGCAGCTAAAGCAATAATCAATAAAGCGTT
CACTATCGTTGACGCACATTGGCGAGGAATTGGTGTTATTCCTGGTTCTGGATTTTCCTTTGCTTCGAGACTATCGCACCTAGACGCCACAAACGATTATGCGCCAGTCGATTTCCCTTCGCAATGTGCGCAAAACGTTCCTGAAACCACATCGCCCTGTGAAAAAGTGA
TCCTCGGAAAAATGGCACCTGATGCGTGCCCGTTTTTTGGTCAGGAATGCAAACCAGCATCCCCTAAAGGCGCATGTATGGTGTCTGATGAGGGGGCTTGCCGAATTTGGTATAGCTCTGGTGAGCGCTCGATAACAAATGTTATCAAAAAGGGGAATACGCTAAAGGTG
GAGATGAAGTGATGACAACTGCCCGCACATATGTTGCCAATACGAATAAGAGTAAAACTATCGCCTATGAGTTGAACATTACTGGAGTCATCCAGGGGGTTGGGTTCAGACCGTTAGTTTACTGCTTAGCGTATGAAAAAAGTATTGTTGGTTGGGTACAAAACGACTGT
GGATGTGTGCGAATACACGCTGAAGGCACTGAGCTAGACGTTGAACAATTTGTATTCGACTTACTGAACAATGGGTCGCTAGTTTCCATATCCTTGCTGGAAAAAAAGCAGGTTAAACTGAGTAATGTAGACTCGTTCACGATAAAAGAAAGCGGTCATGATATAGCGTG
CGGGGCGATATCAATCCCCAAAGATGTCTATTTGTGTGGAGCGTGTAAAGCTGAGTTACTGTCAAATACTAACCGACGTGGCACCTATAGCTTTATCGCTTGCAGTAAATGTGGTCCACGATTTTCTATGCTGCGAGCAATGCCATACGACAGAAAGAACATGAGTATGT
CAGCATTTCCAATGTGTGAGTTATGTCGTAAAGAATATCAATCGCCAACTGATAGGCGCTTTCATGCTCAACCAATAAGCTGTCGCGTATGCGGGCCTGAAGTATTTTGTTCAACTGTCGGCGGGCGCGTGGTCTCGCAAGGCGATGTTGATGTTGTAGCAACGGTTGTC
GCGTATTTGAATCAAGGCAAAATTGTCGCGTTAAAAAGTATAGGCGGTTATCACTTGCTCTGTGACGCACAAAGCACTGAAGCGGTAGACCTTTTACGCCAGCGTAAAAATAGACCAGATAAACCTTTCGCGGTCATGCTCCCAGAGCCTCGTGCAGACGAAACAAGTGA
GGATTGGTTAGATAAATGCGTTGCTGTAGACAGTCATCAAAGAGCGCTCTTACTAAGCCCCGTGCGACCCATTTTACTTGCAAAAAAGCAATCGAATGTACCCATTGCAGATAACGTAGCGCCCATGCTTCGTGATTTGGGGGTGATGTTGCCGTGCAGCGGACTGCATT
TACTCATTATGCAGCAATTCAATCGCCCCATGGTGGCTACGTCAGGAAATATTTACGGTGAACCGATGGTCACATCGACGTCATCGGCACAGCAACACCTTTCGATGTTGGCAGACATCTTCGTGCACCATAATCGAGACATTTTTCACAAACTGGATGATTCTGTGCTG
CGAACGCTTAAGTCAGGCACCATTCCAATTCGCCTGGGGCGGGGTATAAGTCCGATAGAATTATCATTGCCATATACTGTGCAGGAACCAATGCTTGCTGTTGGCGCGAATCAGAAAAATACAGTCGCAATAGCTTTGAACAATCGACTTATCGTAACACCGCATATTGG
CGACCTCGATTCTCCGATAATGCAAGCGCACTTTGAAAAATGTATCAGTGACTTTCAAGCTCTCTATCATGTTCATCCACAGCGTATTCTTTGCGATAGCCATCCTGGCTATATCAGTTCCCGTTGGGCCAAAGGCAGTGAACTACCGACTACTAAAGTTTTACATCACC
ACGCTCATGCATCAGCGTGGGCATTAGCTGCTGAAATATCGACACCAAGCTTGGTTTTCGTGTGGGATGGAACGGGATTAGGCGAGAATGGCGAGATGTGGGGAGGGGAAGTTTTTTGTGGGACGCCGGGCAATTGGAAACGTGTTGCCTCATTAGCGCCTATGAAGTTG
CAAGGCGGCAACAAGGTTCCAAATCAACCATGGCGAAGTGCAGCATCACTAGTGTGGGGTACTAATTTCACCGGCATTGATTTTAAACCATTTGATCCACAACAATTAAGCTACAACGCATGGCAAAACGAACTCAATTGTCATGTTAGTAGTTCAATGGGAAGACTGTT
CGACGCGGCGGCATTTGCCTGCTGTGAACAGAGCGAAGTCTCTTATGAAGGGCAGGCGCCGATGATGTTGGAAAGCGTTGCGTCGACGCCCTCTAAGGTTATGCCGTTAACGTTGGTCCAGAAAAACGACGAATTTATTCAAATGGATTGGCGAACACTGGTACCGGAAC
TATTTGATTTCAAAGTGTCTCCTCAAACTCGTGCTGCAAATTTCCACGCTTCGTTAATTGAAACGGTATGGGAGGTAACCCTACATTGCCGTAAAAAGTTTAATTTTGACCACATTGGATTGGCAGGAGGCGTCTTCCAAAATCGCATTTTGTGTGACGGTATATCACAA
CGGTTTTTACAATCAGACATTCCTGTGGTTATTCCAAGTAGTTTGCCATTAAACGATGCCGCAATAAGTGTTGGGCAAATCCACGAATACTGTTCACGGAGGTCATAATGAAAAGCGCAGAGGGCAATAATCATTCATCATCAGAGCCGTTGGGCACGGAATCTATTCAA
TTAGCTCAGGGGAACGGCGGAACACTGACAAAAAGACTCATTGATCATGTTTTTAAAAATAAAGCGAACAAAGATCTAGATCTGCTTCATGATGCCGCCACAGTAACTTTTGATGGTCCTTATCTGAGTATAACAACGGATAGCTTCGTTGTATCCCCCCCAACATTTCC
AGGCGGCAATGTAGGCGCGTTGAGTGTTTACGGTACCGTCAATGATTTAGCAGTCGTTGGGGCAACACCACGGTACATCAGTACTGCATTTATTATTGAAGAAGGCTTTTCCCTTTCAACATTGAAACAAATCGTTAATGGAATGCATCAAGCTGCCGAAGAAACGAAGG
TCGCCATTGTAACAGGTGATACGAAAGTTGTCCCGAAAGGCAGTGGTGGCGGCGTTTTTATCAATACAACGGGTGTTGGAGACAGCCGAGATGCGCCGATTTGGGATACCAGTCTTATAAAGGCAGGCGATCATGTACTGGTTAGTGGTTCTGTAGGTGATCACGGTGCG
TGTGTCCTGCTTGCTCGCGAGGACTATGGTTTGCAAGGTCAGCTAAAGTCTGATTGTGGAAGCGTTGTTCCTCTTATTGATCCAATCAAGCATTTGGAAGGTGTTCGTTTTGTGCGTGACCCAACTCGTGGAGGGTTGTCAGTATTACTCCATGACGTTGCTAATGAAAC
AGGCTTCGATATCGAGTTAATTGAAAATAATATTCCGGTGCGACCTGAAGTGGCAAGCGTGTGTGAAATATTAGGCTTTGATCCGTTTGTTTTAGCGTGTGAGGGACGCATTGTTGCTGTTGTTGCCCCTGACATAAGCGGTAAGGTGCTAGAACACTGGCGGAGCATTC
GAAACGGGGAGCAGGCCGAACACATTGGTGTAATAGTAAAAAACACCGAATCAAAAGGGCGCGTGACCATTGTTACGCCAATGGGAGGAAGACGTTTCATGAATGAACTCGAAGATGAGCCGCTTCCAAGGATCTGCTGAGATCCTGGCCTCTAAACGGGTCTTGAGGGG
TTTTTTGCTGAAAGGAGGAACTATATCCGGATATCCACAGGACGGGTGTGGTCGCCATGATCGCGTAGTCGATAGTGGCTCCAAGTAGCGAAGCGAGCAGGACTGGGCGGCGGCCAAAGCGGTCGGACAGTGCTCCGAGCGAGCTTCTGGAGCAGGAAGATGTCGCGGGC
ATTAGCACCAGCGGTCTGCCAAGCCTCCGCCAGCCGTTGGGTCCCTTCCGCTTGAGCTTTTCCATCTTCGACGATACGGGCGGCGGCCCCCCGCGCTTCCGCGATCGCCCGTTTACAAGCTGCCTCAGCTGGGGCGATCACATCGGCTTGAAGTTGCTGCTGCACCTGTT
TGATCCGCTCCTGCTGCACAGGGAGTTCTGCTTGGCTACGAGCGACTTCGGTAGCAATGTCCGCTTCAGCTTCGGCCACCACCGCTTCGCGCCGCGTCAACGCATCCTGAATCCGGCGCTCGGCCTCGGCTTGGGCGATCGCTACATCGCGATCGATCCGACGCAGGGCC
GTGATCTTGTCATTTTCGGCCGTTTGGATCGCAGAGGCAGCCTGGGCATCGGCTTCAGCAATTCGGGCATCTCGCTGCAGATCAGCCCGCTGCTTGCGTCCACTAGCCGAGAGATAACCGACCTCATCGGAAATGTTCTGGACTTGCAGCGTATCGAGGACTAGACCCAG
CTGCTCAAGGTCATCCTCCGCCTCTTCCAGCAGACTTTTGGCAAAGGCAATTTTGTCCTCGTTGATCTGCTCCGGCGTGAGGCTGGCTAAAACACCACGCAAGTTGCCTTCGAGGGTCTCCTTGGCAATTTGCTCGATTTCCTTACGGTTTTTGCCAAGCAGCCGCTCGA
TCGCGTTGTGGATGGTCGGTTCTTCCCCAGCAATCTTGATATTGGCAACGCCTTCAACAGTCAGGGGAATGCCGCCCTTGGAGAAGGCATTGGAAACGCGCAACTCAATGATCATGTTGGTCAGATCCATGCGGAGCGCTTTTTCCAGCAGAGGTACCCGCAGGCTGCTG
CCGCCCTTGACCAAGCGATAGCCAACTCGGCGGCCATCACTACTGCGGCGACTACTGCCAGCAAAGATCAAAATTTCACTGGGTTGGCAGATGTAGTAGAGATTGCGCAGGACTAAGCTGCCAGCCCCGGCGACTCACCAGTCACAGAAAAGCATCTTACGGATGGCATG
ACAGTAAGAGAATTATGCAGTGCTGCCATAACCATGAGTGATAACACTGCGGCCAACTTACTTCTGACAACGATCGGAGGACCGAAGGAGCTAACCGCTTTTTTGCACAACATGGGGGATCATGTAACTCGCCTTGATCGTTGGGAACCGGAGCTGAATGAAGCCATACC
AAACGACGAGCGTGACACCACGATGCCTGCAGCAATGGCAACAACGTTGCGCAAACTATTAACTGGCGAACTACTTACTCTAGCTTCCCGGCAACAATTAATAGACTGGATGGAGGCGGATAAAGTTGCAGGACCACTTCTGCGCTCGGCCCTTCCGGCTGGCTGGTTTA
TTGCTGATAAATCTGGAGCCGGTGAGCGTGGGTCTCGCGGTATCATTGCAGCACTGGGGCCAGATGGTAAGCCCTCCCGTATCGTAGTTATCTACACGACGGGGAGTCAGGCAACTATGGATGAACGAAATAGACAGATCGCTGAGATAGGTGCCTCACTGATTAAGCAT
TGGTAACTGTCAGACCAAGTTTACTCATATATACTTTAGATTGATTTAAAACTTCATTTTTAATTTAAAAGGATCTAGGTGAAGATCCTTTTTGATAATCTCATGACCAAAATCCCTTAACGTGAGTTTTCGTTCCACTGAGCGTCAGACCCCGTAGAAAAGATCAAAGG
ATCTTC

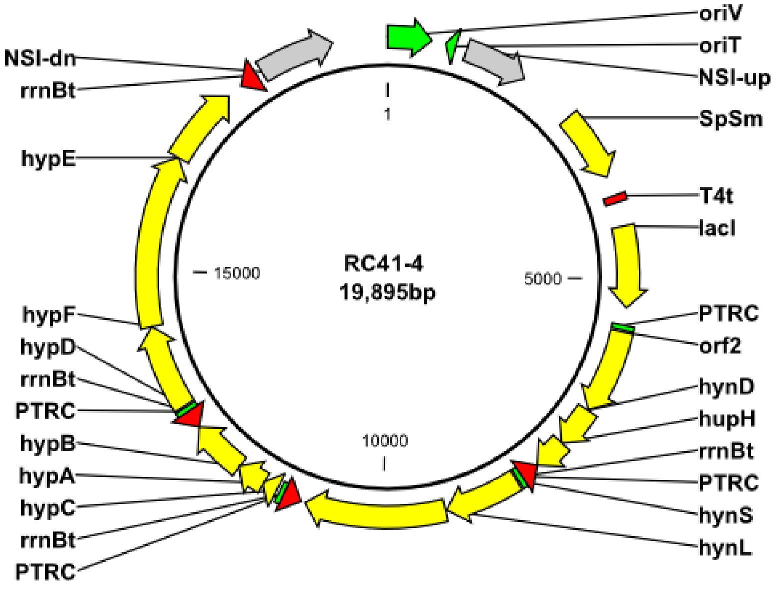

TTGAGATCCTTTTTTTCTGCGCGTAATCTGCTGCTTGCAAACAAAAAAACCACCGCTACCAGCGGTGGTTTGTTTGCCGGATCAAGAGCTACCAACTCTTTTTCCGAAGGTAACTGGCTTCAGCAGAGCGCAGATACCAAATACTGTCCTTCTAGTGTAGCCGTAGTTAG
GCCACCACTTCAAGAACTCTGTAGCACCGCCTACATACCTCGCTCTGCTAATCCTGTTACCAGTGGCTGCTGCCAGTGGCGATAAGTCGTGTCTTACCGGGTTGGACTCAAGACGATAGTTACCGGATAAGGCGCAGCGGTCGGGCTGAACGGGGGGTTCGTGCACACAG
CCCAGCTTGGAGCGAACGACCTACACCGAACTGAGATACCTACAGCGTGAGCTATGAGAAAGCGCCACGCTTCCCGAAGGGAGAAAGGCGGACAGGTATCCGGTAAGCGGCAGGGTCGGAACAGGAGAGCGCACGAGGGAGCTTCCAGGGGGAAACGCCTGGTATCTTTA
TAGTCCTGTCGGGTTTCGCCACCTCTGACTTGAGCGTCGATTTTTGTGATGCTCGTCAGGGGGGCGGAGCCTATGGAAAAACGCCAGCAACGCGGCCTTTTTACGGTTCCTGGCCTTTTGCTGGCCTTTTGCTCACATGTTCTTTCCTGCGTTATCCCCTGATTCTGTGG
ATAACCGTATTACCGCCTTTGAGTGAGCTGATACCGCTCGCCGCAGCCGAACGACCGAGCGCAGCGAGTCAGTGAGCGAGGAAGCGGAAGAGCGCCTGATGCGGTATTTTCTCCTTACGCATCTGTGCGGTATTTCACACCGCATATGGTGCACTCTCAGTACAATCTGC
TCTGATGCCGCATAGTTAAGCCAGTATACACTCCGCTATCGCTACGTGACTGGGTCATGGCTGCGCCCCGACACCCGCCAACACCCGCTGACGCGCCCTGACGGGCTTGTCTGCTCCCGGCATCCGCTTACAGACAAGCTGTGACCGTCTCCGGGAGCTGCATGTGTCAG
AGGTTTTCACCGTCATCACCGAAACGCGCGAGGCAGGATATCCGGCAGCCGGCGGAGCGCTGCTTTCTTGGCAAGCGGTCGCCAGCCCCAACGCCAGGGCTGCCAGCCCGAAACAGCGGGGCAAGGCAGCTTGGAAGGGCGATCGCAGCACGGGCATGGCAATGTCTCTC
TGAAGGAATGCAGACCTTATTCGTACAGCCAGGGTTGAATCGTGGGGGTCCAATCACTTAGCTCTGCTGGGCTAAACCAGAGAGCAATTTCCTGTTGTGCTGTTTCGATTGCATCCGAGCCATGGATGATGTTGCGGCCAATATTGACACCAAAATCACCACGGATGGTG
CCCGGTTCTGCCGTCAGCGGATTGGTAGCGCCGATCAACTTGCGAGCAGCCGCCACAACGCCTTCGCCTTCCAAGACGATCGCCACGATCGGCCCAGAGGTGATGAACTCGACGAGGCCATTGAAGAAGGGGCGCTCGCGGTGGACAGCATAGTGCTGTTCGGCCAGCTC
GCGACTGGGCTTCAGCTGCTTTAGGCCCACCAGTTTGAAGCCTTTTTGCTCAAAGCGGCCGATGATCGTACCGACCAAACCCCGCTGAACGCCATCGGGCTTGATGGCAATAAATGTGCGTTCCACAGACATCTAGATAGTCCTCAAGACGAGGCAAGCATTGAGCTTGC
CTTCCTATGGTTCGGGATCACTGGGATTCTTGACAAGCGATCGCGGTCACATCGCTATCTCTTAGGACTTCGCAGCGGGCGAGTCGGATTGACCCGGTAGGGATTTCGCCAGATCAATGCCCGTGGTTTGTTTCAGCTTCTCCAGCAAGCTAGCGATTTGGGTAGCGCTG
CCTTCCCCTTCGCCAATCACAGTGATCGACTCCACGTCGATATCTGGCACGGTGCCTGAAAGCGTGACGAGCAGGGACTCGAAGCTTGCATGCCTGCAGGTCGACTCTAGAGCTTTATGCTTGTAAACCGTTTTGTGAAAAAATTTTTAAAATAAAAAAGGGGACCTCTA
GGGTCCCCAATTAATTAGTAATATAATCTATTAAAGGTCATTCAAAAGGTCATCCACCGGATCAATTCCCCTGCTCGCGCAGGCTGGGTGCCAAGCTCTCGGGTAACATCAAGGCCCGATCCTTGGAGCCCTTGCCCTCCCGCACGATGATCGTGCCGTGATCGAAATCC
AGATCCTTGACCCGCAGTTGCAAACCCTCACTGATCCGCATGCCCGTTCCATACAGAAGCTGGGCGAACAAACGATGCTCGCCTTCCAGAAAACCGAGGATGCGAACCACTTCATCCGGGGTCAGCACCACCGGCAAGCGCCGCGACGGCCGAGGTCTTCCGATCTCCTG
AAGCCAGGGCAGATCCGTGCACAGCACCTTGCCGTAGAAGAACAGCAAGGCCGCCAATGCCTGACGATGCGTGGAGACCGAAACCTTGCGCTCGTTCGCCAGCCAGGACAGAAATGCCTCGACTTCGCTGCTGCCCAAGGTTGCCGGGTGACGCACACCGTGGAAACGGA
TGAAGGCACGAACCCAGTGGACATAAGCCTGTTCGGTTCGTAAGCTGTAATGCAAGTAGCGTATGCGCTCACGCAACTGGTCCAGAACCTTGACCGAACGCAGCGGTGGTAACGGCGCAGTGGCGGTTTTCATGGCTTGTTATGACTGTTTTTTTGGGGTACAGTCTATG
CCTCGGGCATCCAAGCAGCAAGCGCGTTACGCCGTGGGTCGATGTTTGATGTTATGGAGCAGCAACGATGTTACGCAGCAGGGCAGTCGCCCTAAAACAAAGTTAAACATCATGAGGGAAGCGGTGATCGCCGAAGTATCGACTCAACTATCAGAGGTAGTTGGCGTCAT
CGAGCGCCATCTCGAACCGACGTTGCTGGCCGTACATTTGTACGGCTCCGCAGTGGATGGCGGCCTGAAGCCACACAGTGATATTGATTTGCTGGTTACGGTGACCGTAAGGCTTGATGAAACAACGCGGCGAGCTTTGATCAACGACTTTTGGAAACTTCGGCTTCCCC
TGGAGAGAGCGAGATTCTCCGCGCTGTAGAAGTCACCATTGTTGTGCACGACGACATCATTCCGTGGCGTTATCCAGCTAAGCGCGAACTGCAATTTGGAGAATGGCAGCGCAATGACATTCTTGCAGGTATCTTCGAGCCAGCCACGATCGACATTGATCTGGCTATCT
TGCTGACAAAAGCAAGAGAACATAGCGTTGCCTTGGTAGGTCCAGCGGCGGAGGAACTCTTTGATCCGGTTCCTGAACAGGATCTATTTGAGGCGCTAAATGAAACCTTAACGCTATGGAACTCGCCGCCCGACTGGGCTGGCGATGAGCGAAATGTAGTGCTTACGTTG
TCCCGCATTTGGTACAGCGCAGTAACCGGCAAAATCGCGCCGAAGGATGTCGCTGCCGACTGGGCAATGGAGCGCCTGCCGGCCCAGTATCAGCCCGTCATACTTGAAGCTAGACAGGCTTATCTTGGACAAGAAGAAGATCGCTTGGCCTCGCGCGCAGATCAGTTGGA
AGAATTTGTCCACTACGTGAAAGGCGAGATCACCAAGGTAGTCGGCAAATAATGTCTAACAATTCGTTCAAGCCGACGCCGCTTCGCGGCGCGGCTTAACTCAAGCGTTAGATGCACTAAGCACATAATTGCTCACAGCCAAACTATCAGGTCAAGTCTGCTTTTATTAT
TTTTAAGCGTGCATAATAAGCCCTACACAAATTGGGAGATATATCATGAAAGGCTGGCTTTTTCTTGTTATCGCAATAGTTGGCGAAGTAATCGCAACATCCGCATTAAAATCTAGCGAGGGCTTTACTAAGCTGATCCGGTGGATGACCTTTTGAATGACCTTTAATAG
ATTATATTACTAATTAATTGGGGACCCTAGAGGTCCCCTTTTTTATTTTAAAAATTTTTTCACAAAACGGTTTACAAGCATACTAGAGGATCGGCGGCCGCGGATCTGGGCCGCGGGTCATGGCTGCGCCCCGACACCCGCCAACACCCGCTGACGCGCCCTGACGGGCT
TGTCTGCTCCCGGCATCCGCTTACAGACAAGCTGTGACCGTCTCCGGGAGCTGCATGTGTCAGAGGTTTTCACCGTCATCACCGAAACGCGCGAGGCAGCAGATCAATTCGCGCGCGAAGGCGAAGCGGCATGCATTTACGTTGACACCATCGAATGGTGCAAAACCTTT
CGCGGTATGGCATGATAGCGCCCGGAAGAGAGTCAATTCAGGGTGGTGAATGTGAAACCAGTAACGTTATACGATGTCGCAGAGTATGCCGGTGTCTCTTATCAGACCGTTTCCCGCGTGGTGAACCAGGCCAGCCACGTTTCTGCGAAAACGCGGGAAAAAGTGGAAGC
GGCGATGGCGGAGCTGAATTACATTCCCAACCGCGTGGCACAACAACTGGCGGGCAAACAGTCGTTGCTGATTGGCGTTGCCACCTCCAGTCTGGCCCTGCACGCGCCGTCGCAAATTGTCGCGGCGATTAAATCTCGCGCCGATCAACTGGGTGCCAGCGTGGTGGTGT
CGATGGTAGAACGAAGCGGCGTCGAAGCCTGTAAAGCGGCGGTGCACAATCTTCTCGCGCAACGCGTCAGTGGGCTGATCATTAACTATCCGCTGGATGACCAGGATGCCATTGCTGTGGAAGCTGCCTGCACTAATGTTCCGGCGTTATTTCTTGATGTCTCTGACCAG
ACACCCATCAACAGTATTATTTTCTCCCATGAAGACGGTACGCGACTGGGCGTGGAGCATCTGGTCGCATTGGGTCACCAGCAAATCGCGCTGTTAGCGGGCCCATTAAGTTCTGTCTCGGCGCGTCTGCGTCTGGCTGGCTGGCATAAATATCTCACTCGCAATCAAAT
TCAGCCGATAGCGGAACGGGAAGGCGACTGGAGTGCCATGTCCGGTTTTCAACAAACCATGCAAATGCTGAATGAGGGCATCGTTCCCACTGCGATGCTGGTTGCCAACGATCAGATGGCGCTGGGCGCAATGCGCGCCATTACCGAGTCCGGGCTGCGCGTTGGTGCGG
ATATCTCGGTAGTGGGATACGACGATACCGAAGACAGCTCATGTTATATCCCGCCGTTAACCACCATCAAACAGGATTTTCGCCTGCTGGGGCAAACCAGCGTGGACCGCTTGCTGCAACTCTCTCAGGGCCAGGCGGTGAAGGGCAATCAGCTGTTGCCCGTCTCACTG
GTGAAAAGAAAAACCACCCTGGCGCCCAATACGCAAACCGCCTCTCCCCGCGCGTTGGCCGATTCATTAATGCAGCTGGCACGACAGGTTTCCCGACTGGAAAGCGGGCAGTGAGCGCAACGCAATTAATGTAAGTTAGCGCGAATTGATCTGGTTTGACAGCTTATCAT
CGACTGCACGGTGCACCAATGCTTCTGGCGTCAGGCAGCCATCGGAAGCTGTGGTATGGCTGTGCAGGTCGTAAATCACTGCATAATTCGTGTCGCTCAAGGCGCACTCCCGTTCTGGATAATGTTTTTTGCGCCGACATCATAACGGTTCTGGCAAATATTCTGAAATG
AGCTGTTGACAATTAATCATCCGGCTCGTATAATGTGTGGAATTGTGAGCGGATAACAATTTCACACAGGAAACAGACCATGGAggaggaactagATGGCTTCCATTATTGGTAGCGGCTTTTTGATCATTGCACCACTGCTGCATTCGGTAATGGGGAAATGGGCTCTG
CTTGGAATAGTGTTGTTGTCCATTCTGGCATATGCGCTGGGGTCGGTTATTCGCTTCAATATACGCCATGCAGAACCTGAGCTAGCTAGCAATCAACAAAGCAGTATTGTGACGCTTGAAAAAGCAAGTCAATGGGCGCTTGGTGCGGCATATGCCATTTCCGTGGCGTT
TTATATTAGTTTGTTTGCAGCGTTTGTTTTTGATCGTTTATCAATCTCTGATACGACGTACATCAAACTGTTTACCAGTGGGTTGCTTGTCATCATTACGGTGGTCGCTTGGCTCCGTGGTGCTAGGGGGCTAGAGACCATTGAGCTATTCGCCGTGACAATAAAGTTGG
CTATTATAGTAGGTGTGCTCGCAGCGCTGGCAACCTACGACATACAAGTACAAAGTGCTTGGTTTCAACATGAAGCGATACAGGCATTGAGCCATTTTGAAACTGTTAGCATGCTCGCAGGCATGCTCATGGTAACGCAAGGGTTCGAGACAACCCGTTTTATGGGGAAT
AACTACACGCCAGAACAGCGCATTAAAGCGAGTCGTTATGCTCAGTGGATCGCTATTTTTCTCTATGTTGTTTTTATCGGTCTAACGTGTCCTATTTTTCTGGATTTTCCGATCACGGAATTAAACGAAACAACGATTAGCTACACCCTAGGGCAAGCAATATGGGTATT
GCCGATTTTATTACTGGTTGCCGCCACGGCAAGTCAATTAAGTGCAGCATTAGCCGATACTATCGGTGGCGGAGGCCTCTTAAAAGAGCTTTTCCATTTACCTATCTCCCCGCAATTTTACTACGTTTTAGTCATCGCCATCGCAGGTATATTAGTTTGGTCATCCAACG
TATTTGAAATTATTAATCTCGCTTCAAAAGGGTTTGCACTTTATTACCTGATCCAAACAATAATCGCGGTCAAACTCGTGATGCGTCAAAGCACAGGTCAACGCATTAGGTCAACAAAATTACTTGGTCTATCGGTGATCGTTCTCTGTTTATTCTTTGTGATTGGCTGG
TCAATTCCAGCACCTCATAGCTAATAAGGAAAATACCGATGCTATCAGTAGAAACTACACCACCAATTGACCAAATCAAAAACGCGGCTAAGCGCTACGCTATCATTGGTGTTGGAAATCTATTGCAGCGAGATGATGGCGTTGGTGTGCACGCGGTTCGTGCTATGCAA
CCCCTGCTAAAGTCTTATTCAAATGTATCTTGCATTGACGCTGGGACACTGAGCTATGAACTTTTGGAGTGGGTTGCGAGTTCCGATCATACGATAGTCATCGACGCGGCTTATATGCGCTGTCCGCCTGGCACGGTGAGAGTGTTTGAGAACGAGGCAATTGCGGATCA
GTTTCAGCAAGCGACGACCCATTCCGTTCACCAGATCACGTTACGCGACACATTGTTGACCAGCCAAACGTTATATTCAAAACCTTCTGCGGTGACGTTAATTGGCGTAGAGCCGGAGGCGATTGAGTGGGGAACCTCTTTGAGTCCAAAGGTAAATGCAGCGCTATCAT
CGATCATGGATATCGCATTTGCTTGCTTATCAGGCGACAACGTTCAGACCTATAAACCGGCAACCCAAAAGGAGGCACGATGACCGAAGAACTCATTTTTATGACATCAAATGCTGATAAAACGGGCAACGTAATGCCGCTTTTGCATCAGATACGTCATGCGTTGTCTC
AGTTAATTGAACGACAAGAACAAACCACGATTGATCTGCGACGATTGCCTTTAAGCGCTAGCGAAGAAGCGCAGTTAGAAGCTTTTCTTGGGCATGGAGAGGTTAAAGCTGATATTCAGGCGCTGGGTGATACGGTGCTAATCGAATCACGCTACGCAGGCGTTTGGCTT
GAAATTCACTATAACGAAGATGTGGAGATCATGGGCAAATACGTTCATATCTGTACCTGTCCACCAATCATAAAATCTCAGCCAGAAGATATGGTGTTATCGCTCAGCAATATAGTGTCTGACATCCATTCGTTGTCTCATCAATCTTCTGATGAAACGGCTAAGGAGGA
TTGACATGCGAGAGTAGGGAACTGCCAGGCATCAAATAAAACGAAAGGCTCAGTCGAAAGACTGGGCCTTTCGTTTTATCTGTTGTTTGTCGGTGAACGCTCTCCTGAGTAGGACAAATCCGCCGGGAGCGGATTTGAACGTTGCGAAGCAACGGCCCGGAGGGTGGCGG
GCAGGACGCCCGCCATAAACTGCCAGGCATCAAATTAAGCAGAAGGCCATCCTGACGGATGGCCTTTTTGCGTTTCTACTACTAGTTGACAATTAATCATCCGGCTCGTATAATGTGTGGAATTGTGAGCGGATAACAATTTCACACATACTAGAAAGAGGAGAAATACT
AGATGGCGCTACCAACATTAAACAAGCAGTTACAAGCGTCCGGTATCTCAAGACGCACATTTCTTAAGTTTTGCGCTACAACGGCGTCTTTACTGGCATTGCCGCAAAGCGCTGTTGCCGATTTGGCGACTGCCCTTGGCAATGCGAGAAGACCCTCTGTGATTTGGTTG
CCCTTTCAAGAGTGCACAGGGTGTACCGAAGCAATATTGCGCTCTCATGCTCCCACATTGGAAAGCCTTATTTTCGATCATATTTCGTTGGATTATCAGCATACGATAATGGCTGCTGCGGGAGAGCAAGCTGAAGACGCTAGGCGTGCGGCGATGAACGCGCACAAAGG
GCAATATTTGTTGTTGGTTGATGGTTCGGTTCCGGTGGGTAACCCAGGATACTCAACGATCAGTGGCATGAGTAATGTCGATATGCTGAGAGAATCGGCAAAAGATGCTGCGGGTATTATTGCCATCGGTACCTGCGCGTCTTTTGGCGGGATCCCTAAAGCAAACCCAA
ATCCGACGGGGGCAGTGGCAGTAAGCGACATTATTACAGACAAGCCAATCGTAAACATTTCAGGCTGTCCGCCACTGCCTATCGCGATTACAGCTGTGTTGGTTCATTACCTGACGTTTAAGCGTTTCCCTGATCTCGACGAATTACAACGCCCACTCGCTTTTTTTGGT
GAAAGCATCCATGACAGATGTTATCGACGCCCATTTTTTGAACAACGTAAATTTGCAAAATCGTTTGATGATGAGGGGGCAAAAAATGGGTGGTGTTTGTTCGAACTTGGTTGTAAAGGGCCCGAAACCTTTAACGCATGTGCAACGGTTAAATGGAATCAAGGCACGAG
TTTTCCTATCGAATCTGGCCATCCGTGTCTTGGTTGCTCTGAGCCCGATTTCTGGGATAAAAGCAGCTTTTACCAAGCCTTGGGTCCATGGGAGTGGTACAAATCCAAACCCGGCAAAGGTGCACAGAAGCATGCTGGGAAAAACTCATAAGATAATAGGCAAGGCTCAT
GGAAAATACAGCAAGTAACAACCGGTTAGTCGTCGATCCTATCACTCGAATTGAAGGGCATCTTCGAATAGAAGCTGAAATGGATGGGAATACCATCAAACAGGCGTTCTCATCAGGCACGTCTGTTCGGGGAATTGAACTGATTTTACAAGGCAGAGATCCGCGTGACG
CTTGGGCTTTTGCGCAACGTATCTGTGGCGTCTGCACGCTGGTGCATGGTATGGCATCGGTGCGTGCTGTCGAAGATGCAATTAGAAAAGCTTGGCGGTCAAACGCAAAATTAGGGGTTGCCATTGGAAAGCCCTCCATGACATCTATGCCAAAAGGACCGATGCAACAT
GGTAAAAAAGGGCACCGACAGTCACGTACTTCAATAGGCGTACTGAGTGAAGCAGAAATGGCTATCCCTCAAAATGCACAACTGATAAGAAACATCATGATTGCAACCCAATATGTGCATGATCACGTGATGCATTTTTATCACTTGCATGCCCTAGATTGGGTTGACGT
TGTTTCTGCACTAGATGCAGATCCAACGAGAACCGCTACGCTCGCCGGTCAATTGAGTGATTATCCTCGTTCATCGCCGGGATATTTCAAAGATGTGAAGCAAAAAGTCAAAACGCTGGTTGAGTCTGGGCAGCTAGGGATATTCAGTAACGCGTATTGGGGGCATCCTG
GCTATAAACTGCCACCTGAAGTTAACTTGATGGCATTAGCACATTATTTAGATGCGCTAACGTGGCAGCGTGAAGTTGTAAAAGTTCACACCATATTTGGAGGGAAAAACCCTCATCCTAATTTTGTCGTTGGCGGCGTGCCTTCACCGATTAATCTCAATGCGTCAACG

GGTATTAACACGAGTCGATTAGTGCAACTACAAGATGCTATCACGCAAATGAAGAGCTTTGTCGATCAGGTGTATTACCCCGATATTGTGGCGATTGCGGGTTATTACAAAGAGTGGGGGACACGAGGGGAAGGGCTGGGTAACTTTCTTACCTATGGAGACTTACCTAT
GACATCAATGGATGACCCTGATTCTTTCTTGTTTCCACGAGGTGCAATACTTGGTCGAGACTTGAGTAAAGTGCATGACCTTGATCTAGATGATCCCTCTGAAATTCAAGAATTCGTCTCTTCCTCCTGGTATCGATATAGTGGAGGGAACGCAAGTGGTTTACACCCTT
TTAATGGACAAACAACACTCGAGTATACTGGTCCGAAACCGCCTTACAAGCACCTAAATGTAGGGGCTGAATATTCATGGTTGAAAAGTCCGCGTTGGAAAGGCCATGCGATGGAGGTGGGACCGCTGGCTCGCGTGCTAATGATGTATGCTAAAAAAGATGCCGCTGCG
CAAGACATCGTTAATCGATCTCTTTCTATCTTGGATTTAGAGACCTCTGCACTTTTCTCCACACTCGGTAGGACGCTCGCCAGAGCAGTGGAAACAAAAATTGTGGTTAACCAGCTACAGTCTTGGTATGACCAACTATTGGATAATATCGCAAAGGGCGATACCGATAC
GTTTAACCCTCTGTATTTTGACCCTACCAATTGGCCAATTAAAGGCCAGGGGGTAGGCGTGATGGAAGCGCCTCGTGGCGCGTTGGGGCATTGGTTGGTCATGCAAAATGGCAAAATTGAGAATTACCAATGTGTCGTGCCTACGACATGGAACGCTGGACCTCGAGATC
CCAACTCACAGGCAGGTGCTTATGAAGCCGCTCTGCAAGATAAACATACGCTACATGATCCTGACCAACCTTTAGAGATTTTGCGAACACTTCATAGTTTTGACCCCTGCTTAGCATGTGCCGTGCACGTAATGGACGAAACAGGGGAAGAGCGTTTGCGTCTAAAAGTT
CGTTAAgatcctctacgccggacgcatcgtggccggTCGAGCTCAAGGAGGAATAACACATGCGAGAGTAGGGAACTGCCAGGCATCAAATAAAACGAAAGGCTCAGTCGAAAGACTGGGCCTTTCGTTTTATCTGTTGTTTGTCGGTGAACGCTCTCCTGAGTAGGACA
AATCCGCCGGGAGCGGATTTGAACGTTGCGAAGCAACGGCCCGGAGGGTGGCGGGCAGGACGCCCGCCATAAACTGCCAGGCATCAAATTAAGCAGAAGGCCATCCTGACGGATGGCCTTTTTGCGTTTCTACTACTAGTTGACAATTAATCATCCGGCTCGTATAATGT
GTGGAATTGTGAGCGGATAACAATTTCACACATACTAGAAAGAGGAGAAATACTAGATGTGTCTCGCTATCCCCATGAAAGTCATTGCAATTAAAGGCTTTAACGCAACGTGTGAAGCGAAAGGTGTCTCTCGCGAGGTGAGTTTACATTTAGTGCAAGGGCTAGAGGTC
AAAGTTGGCGACTATGTGATGGTACATGTTGGTTATGCTCTCCAAGTGATCACCTATGATGAAGCGCAGGTGACATGGGAGATGCTCGATCAGGTCATTGCTTACGATGCATGAAATTAGTCTTTGCTATAGCTTGCTTGACACAGTCGCTGTTCACCAGCGAGCAAACA
TGAATAAGTCTGTCAGTCTGGTTCACGTCAAAGTCGGCCCACTGTCAGGTGTTGAGCCTGACTTGCTGCACCATGCGTTCTTGGCGTGTAGAACTCACACCATTGGCGATCAGGCAACGCTTAGAATTGACACAAGCGCTATCAAGATACGCTGTAAATTGTGTGGCGAA
TTCAGCCGAGTTTCGGTGAATAGCATCCTATGTGCAAGTTGCGGTGCTTGGCAAACCGACCTCATTGAGGGCGATGAATTTATATTGCAACGAATAGAGTTTATCGCACCTGACAATTTTACAGAATCAAAGGAGAGAAAACATGTGCGGTAACTGTGGTTGCGAAACGC
AAGTAAAATCAGAGAAAACTGACAAGCCATCAGCAAATATCGTTGAGGTTCACGCAAATTTAAAAAGCAACAATGACAACCAAGCGATAGCAAACCGTGCTTTATTTGACGCACATAATGTACTAGTGATTAATTTGATGTCATCCCCAGGCAGTGGAAAAACCAGGCTA
CTCGAAGAAACGATACGTGCGCTTAAGTCGCAATACGCGATGGCGGTGATTGAAGGCGACCTCGAAACTGAAAATGACGCAAATAGAATTAGACGTCATGGTGTGCAGGCGGAGCAAATCGCCACGGGCCAAGGGTGTCACTTGGACGCCAGTATGGTGCAAAAGGTACT
TGGAAGATTTAGCTTACAAAAACTAGACGTTTTGTTTATAGAGAATGTTGGCAATTTGATTTGTCCAGCGTGTTTTGATTTAGGACAGCACTTAAACATTATTTTGCTGTCCGTACCAGAAGGTGATGATAAGCCTGAAAAGTACCCAGTGATGTTTCGTGCCGCGGACG
TCATGCTTATTTCTAAGACCGATTACTTGCAATTTCACGATGAATTCAATGTACCTCGCGCGATTGAATCATTTCGTAAAGTGGGCAACGATGCGCCCGTTCTCGAAGTGTCTTCATTGAAAAGTCAACATCTAGAGCGTTGGTTTTCTTATTTTACGCACGCCATTACA
GCTCATCAGTCTCAGTCGTTACAAAATAGCGGAGTCTAACATGCGAGAGTAGGGAACTGCCAGGCATCAAATAAAACGAAAGGCTCAGTCGAAAGACTGGGCCTTTCGTTTTATCTGTTGTTTGTCGGTGAACGCTCTCCTGAGTAGGACAAATCCGCCGGGAGCGGATT
TGAACGTTGCGAAGCAACGGCCCGGAGGGTGGCGGGCAGGACGCCCGCCATAAACTGCCAGGCATCAAATTAAGCAGAAGGCCATCCTGACGGATGGCCTTTTTGCGTTTCTACTACTAGTTGACAATTAATCATCCGGCTCGTATAATGTGTGGAATTGTGAGCGGATA
ACAATTTCACACATACTAGAAAGAGGAGAAATACTAGATGTACAGTGTTGAATCGTTGCTAAGAGACATTAAGAATACCTCGCTTTCAAAACCGTTTCGTATTCTTAATGTTTGCGGTGGCCATGAGCGTGCGATTACGCGCGCTGGATTTAGAACGTTGTTTCAGCACA
ATATTCATCTGATACCTGGGCCAGGGTGCCCCGTTTGTATTTGCCCCGAAGAGGATATTGCATACGCGATACACTTAGCCATTAACGAAAATGTCGTTATCGTTAGTTTTGGTGACATGCTCCGCGTCCCAGTCGAACACGAAATGGGTGGCTGTAGCTCATTGATAGAC
GCAAAAAATCAAGGGGCCGATATCCGTCCGATATCTTCTCCTCAAGAAGCGGTATCTATAGCATTGGAAGAACCTCGTAAGGTTATTGTGTTTTTTGCTGTTGGTTTTGAAACGACTATGGCACCGATTGCAGCAACACTGCTTAATGATTTACCCAATAACTTTAAAGT
ATTATTGTCTGGACGACTTACTTGGCCAGCAGTTGCCCATGTGCTTGAGAGTCAACCGAATACTTTTGATGCACTGATTGCGCCAGGCCACGTTGCGACAATTATGGGTAGTGAAGAATGGCAGTTTGCAATCGACCATCATAATTTGCCTGTCAGTATTGCTGGTTTTC
ATCCAGAAAGTTTGCTGTTGTCGCTCCAAACGCTGCTCGGTAATTGTTCAAATAAAGTCGTCACACTGAGTAACCGCTATCCCGAAGTGGTAAAGCAAAACGGCAATGCAGCAGCTAAAGCAATAATCAATAAAGCGTTCACTATCGTTGACGCACATTGGCGAGGAATT
GGTGTTATTCCTGGTTCTGGATTTTCCTTTGCTTCGAGACTATCGCACCTAGACGCCACAAACGATTATGCGCCAGTCGATTTCCCTTCGCAATGTGCGCAAAACGTTCCTGAAACCACATCGCCCTGTGAAAAAGTGATCCTCGGAAAAATGGCACCTGATGCGTGCCC
GTTTTTTGGTCAGGAATGCAAACCAGCATCCCCTAAAGGCGCATGTATGGTGTCTGATGAGGGGGCTTGCCGAATTTGGTATAGCTCTGGTGAGCGCTCGATAACAAATGTTATCAAAAAGGGGAATACGCTAAAGGTGGAGATGAAGTGATGACAACTGCCCGCACATA
TGTTGCCAATACGAATAAGAGTAAAACTATCGCCTATGAGTTGAACATTACTGGAGTCATCCAGGGGGTTGGGTTCAGACCGTTAGTTTACTGCTTAGCGTATGAAAAAAGTATTGTTGGTTGGGTACAAAACGACTGTGGATGTGTGCGAATACACGCTGAAGGCACTG
AGCTAGACGTTGAACAATTTGTATTCGACTTACTGAACAATGGGTCGCTAGTTTCCATATCCTTGCTGGAAAAAAAGCAGGTTAAACTGAGTAATGTAGACTCGTTCACGATAAAAGAAAGCGGTCATGATATAGCGTGCGGGGCGATATCAATCCCCAAAGATGTCTAT
TTGTGTGGAGCGTGTAAAGCTGAGTTACTGTCAAATACTAACCGACGTGGCACCTATAGCTTTATCGCTTGCAGTAAATGTGGTCCACGATTTTCTATGCTGCGAGCAATGCCATACGACAGAAAGAACATGAGTATGTCAGCATTTCCAATGTGTGAGTTATGTCGTAA
AGAATATCAATCGCCAACTGATAGGCGCTTTCATGCTCAACCAATAAGCTGTCGCGTATGCGGGCCTGAAGTATTTTGTTCAACTGTCGGCGGGCGCGTGGTCTCGCAAGGCGATGTTGATGTTGTAGCAACGGTTGTCGCGTATTTGAATCAAGGCAAAATTGTCGCGT
TAAAAAGTATAGGCGGTTATCACTTGCTCTGTGACGCACAAAGCACTGAAGCGGTAGACCTTTTACGCCAGCGTAAAAATAGACCAGATAAACCTTTCGCGGTCATGCTCCCAGAGCCTCGTGCAGACGAAACAAGTGAGGATTGGTTAGATAAATGCGTTGCTGTAGAC
AGTCATCAAAGAGCGCTCTTACTAAGCCCCGTGCGACCCATTTTACTTGCAAAAAAGCAATCGAATGTACCCATTGCAGATAACGTAGCGCCCATGCTTCGTGATTTGGGGGTGATGTTGCCGTGCAGCGGACTGCATTTACTCATTATGCAGCAATTCAATCGCCCCAT
GGTGGCTACGTCAGGAAATATTTACGGTGAACCGATGGTCACATCGACGTCATCGGCACAGCAACACCTTTCGATGTTGGCAGACATCTTCGTGCACCATAATCGAGACATTTTTCACAAACTGGATGATTCTGTGCTGCGAACGCTTAAGTCAGGCACCATTCCAATTC
GCCTGGGGCGGGGTATAAGTCCGATAGAATTATCATTGCCATATACTGTGCAGGAACCAATGCTTGCTGTTGGCGCGAATCAGAAAAATACAGTCGCAATAGCTTTGAACAATCGACTTATCGTAACACCGCATATTGGCGACCTCGATTCTCCGATAATGCAAGCGCAC
TTTGAAAAATGTATCAGTGACTTTCAAGCTCTCTATCATGTTCATCCACAGCGTATTCTTTGCGATAGCCATCCTGGCTATATCAGTTCCCGTTGGGCCAAAGGCAGTGAACTACCGACTACTAAAGTTTTACATCACCACGCTCATGCATCAGCGTGGGCATTAGCTGC
TGAAATATCGACACCAAGCTTGGTTTTCGTGTGGGATGGAACGGGATTAGGCGAGAATGGCGAGATGTGGGGAGGGGAAGTTTTTTGTGGGACGCCGGGCAATTGGAAACGTGTTGCCTCATTAGCGCCTATGAAGTTGCAAGGCGGCAACAAGGTTCCAAATCAACCAT
GGCGAAGTGCAGCATCACTAGTGTGGGGTACTAATTTCACCGGCATTGATTTTAAACCATTTGATCCACAACAATTAAGCTACAACGCATGGCAAAACGAACTCAATTGTCATGTTAGTAGTTCAATGGGAAGACTGTTCGACGCGGCGGCATTTGCCTGCTGTGAACAG
AGCGAAGTCTCTTATGAAGGGCAGGCGCCGATGATGTTGGAAAGCGTTGCGTCGACGCCCTCTAAGGTTATGCCGTTAACGTTGGTCCAGAAAAACGACGAATTTATTCAAATGGATTGGCGAACACTGGTACCGGAACTATTTGATTTCAAAGTGTCTCCTCAAACTCG
TGCTGCAAATTTCCACGCTTCGTTAATTGAAACGGTATGGGAGGTAACCCTACATTGCCGTAAAAAGTTTAATTTTGACCACATTGGATTGGCAGGAGGCGTCTTCCAAAATCGCATTTTGTGTGACGGTATATCACAACGGTTTTTACAATCAGACATTCCTGTGGTTA
TTCCAAGTAGTTTGCCATTAAACGATGCCGCAATAAGTGTTGGGCAAATCCACGAATACTGTTCACGGAGGTCATAATGAAAAGCGCAGAGGGCAATAATCATTCATCATCAGAGCCGTTGGGCACGGAATCTATTCAATTAGCTCAGGGGAACGGCGGAACACTGACAA
AAAGACTCATTGATCATGTTTTTAAAAATAAAGCGAACAAAGATCTAGATCTGCTTCATGATGCCGCCACAGTAACTTTTGATGGTCCTTATCTGAGTATAACAACGGATAGCTTCGTTGTATCCCCCCCAACATTTCCAGGCGGCAATGTAGGCGCGTTGAGTGTTTAC
GGTACCGTCAATGATTTAGCAGTCGTTGGGGCAACACCACGGTACATCAGTACTGCATTTATTATTGAAGAAGGCTTTTCCCTTTCAACATTGAAACAAATCGTTAATGGAATGCATCAAGCTGCCGAAGAAACGAAGGTCGCCATTGTAACAGGTGATACGAAAGTTGT
CCCGAAAGGCAGTGGTGGCGGCGTTTTTATCAATACAACGGGTGTTGGAGACAGCCGAGATGCGCCGATTTGGGATACCAGTCTTATAAAGGCAGGCGATCATGTACTGGTTAGTGGTTCTGTAGGTGATCACGGTGCGTGTGTCCTGCTTGCTCGCGAGGACTATGGTT
TGCAAGGTCAGCTAAAGTCTGATTGTGGAAGCGTTGTTCCTCTTATTGATCCAATCAAGCATTTGGAAGGTGTTCGTTTTGTGCGTGACCCAACTCGTGGAGGGTTGTCAGTATTACTCCATGACGTTGCTAATGAAACAGGCTTCGATATCGAGTTAATTGAAAATAAT
ATTCCGGTGCGACCTGAAGTGGCAAGCGTGTGTGAAATATTAGGCTTTGATCCGTTTGTTTTAGCGTGTGAGGGACGCATTGTTGCTGTTGTTGCCCCTGACATAAGCGGTAAGGTGCTAGAACACTGGCGGAGCATTCGAAACGGGGAGCAGGCCGAACACATTGGTGT
AATAGTAAAAAACACCGAATCAAAAGGGCGCGTGACCATTGTTACGCCAATGGGAGGAAGACGTTTCATGAATGAACTCGAAGATGAGCCGCTTCCAAGGATCTGCTGAGATCCTCTAGAGTCGACCTGCAGGCATGCAAGCTTGGCTGTTTTGGCGGATGAGAGAAGAT
TTTCAGCCTGATACAGATTAAATCAGAACGCAGAAGCGGTCTGATAAAACAGAATTTGCCTGGCGGCAGTAGCGCGGTGGTCCCACCTGACCCCATGCCGAACTCAGAAGTGAAACGCCGTAGCGCCGATGGTAGTGTGGGGTCTCCCCATGCGAGAGTAGGGAACTGCC
AGGCATCAAATAAAACGAAAGGCTCAGTCGAAAGACTGGGCCTTTCGTTTTATCTGTTGTTTGTCGGTGAACGCTCTCCTGAGTAGGACAAATCCGCCGGGAGCGGATTTGAACGTTGCGAAGCAACGGCCCGGAGGGTGGCGGGCAGGACGCCCGCCATAAACTGCCAG
GCATCAAATTAAGCAGAAGGCCATCCTGACGGATGGCCTTTTTGCGTTTCTACAAACTCTTTTGTTTATTTTTCTAAATACATTCAAATATGTATCCGCTCATGGCGGCCCAGATCCCCGGGTACCGAGCTCGAATTTCGAGCTTCTGGAGCAGGAAGATGTCGCGGGCA
TTAGCACCAGCGGTCTGCCAAGCCTCCGCCAGCCGTTGGGTCCCTTCCGCTTGAGCTTTTCCATCTTCGACGATACGGGCGGCGGCCCCCCGCGCTTCCGCGATCGCCCGTTTACAAGCTGCCTCAGCTGGGGCGATCACATCGGCTTGAAGTTGCTGCTGCACCTGTTT
GATCCGCTCCTGCTGCACAGGGAGTTCTGCTTGGCTACGAGCGACTTCGGTAGCAATGTCCGCTTCAGCTTCGGCCACCACCGCTTCGCGCCGCGTCAACGCATCCTGAATCCGGCGCTCGGCCTCGGCTTGGGCGATCGCTACATCGCGATCGATCCGACGCAGGGCCG
TGATCTTGTCATTTTCGGCCGTTTGGATCGCAGAGGCAGCCTGGGCATCGGCTTCAGCAATTCGGGCATCTCGCTGCAGATCAGCCCGCTGCTTGCGTCCACTAGCCGAGAGATAACCGACCTCATCGGAAATGTTCTGGACTTGCAGCGTATCGAGGACTAGACCCAGC
TGCTCAAGGTCATCCTCCGCCTCTTCCAGCAGACTTTTGGCAAAGGCAATTTTGTCCTCGTTGATCTGCTCCGGCGTGAGGCTGGCTAAAACACCACGCAAGTTGCCTTCGAGGGTCTCCTTGGCAATTTGCTCGATTTCCTTACGGTTTTTGCCAAGCAGCCGCTCGAT
CGCGTTGTGGATGGTCGGTTCTTCCCCAGCAATCTTGATATTGGCAACGCCTTCAACAGTCAGGGGAATGCCGCCCTTGGAGAAGGCATTGGAAACGCGCAACTCAATGATCATGTTGGTCAGATCCATGCGGAGCGCTTTTTCCAGCAGAGGTACCCGCAGGCTGCTGC
CGCCCTTGACCAAGCGATAGCCAACTCGGCGGCCATCACTACTGCGGCGACTACTGCCAGCAAAGATCAAAATTTCACTGGGTTGGCAGATGTAGTAGAGATTGCGCAGGACTAAGCTGCCAGCCCCGGCGACTCACCAGTCACAGAAAAGCATCTTACGGATGGCATGA
CAGTAAGAGAATTATGCAGTGCTGCCATAACCATGAGTGATAACACTGCGGCCAACTTACTTCTGACAACGATCGGAGGACCGAAGGAGCTAACCGCTTTTTTGCACAACATGGGGGATCATGTAACTCGCCTTGATCGTTGGGAACCGGAGCTGAATGAAGCCATACCA
AACGACGAGCGTGACACCACGATGCCTGCAGCAATGGCAACAACGTTGCGCAAACTATTAACTGGCGAACTACTTACTCTAGCTTCCCGGCAACAATTAATAGACTGGATGGAGGCGGATAAAGTTGCAGGACCACTTCTGCGCTCGGCCCTTCCGGCTGGCTGGTTTAT
TGCTGATAAATCTGGAGCCGGTGAGCGTGGGTCTCGCGGTATCATTGCAGCACTGGGGCCAGATGGTAAGCCCTCCCGTATCGTAGTTATCTACACGACGGGGAGTCAGGCAACTATGGATGAACGAAATAGACAGATCGCTGAGATAGGTGCCTCACTGATTAAGCATT
GGTAACTGTCAGACCAAGTTTACTCATATATACTTTAGATTGATTTAAAACTTCATTTTTAATTTAAAAGGATCTAGGTGAAGATCCTTTTTGATAATCTCATGACCAAAATCCCTTAACGTGAGTTTTCGTTCCACTGAGCGTCAGACCCCGTAGAAAAGATCAAAGGA
TCTTC

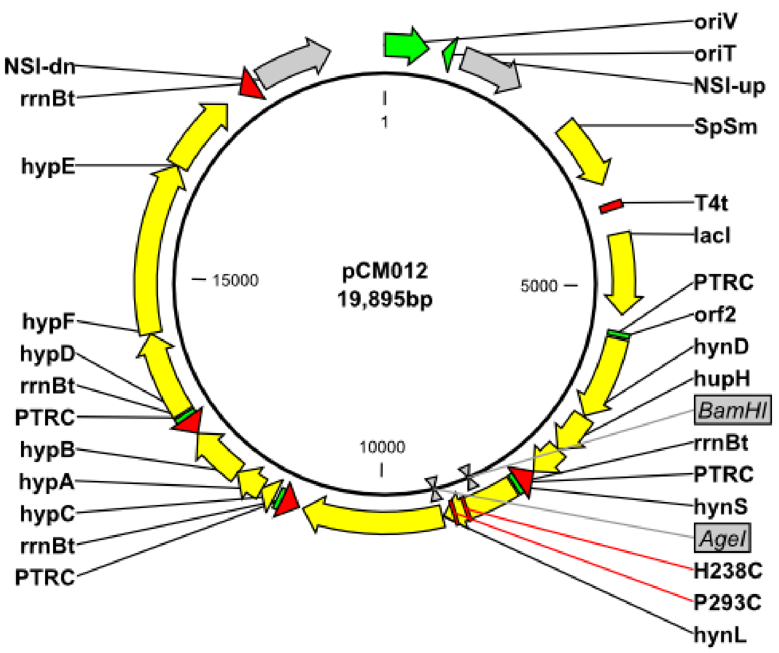


```
TTGAGATCCTTTTTTTCTGCGCGTAATCTGCTGCTTGCAAACAAAAAAACCACCGCTACCAGCGGTGGTTTGTTTGCCGGATCAAGAGCTACCAACTCTTTTTCCGAAGGTAACTGGCTTCAGCAGAGCGCAGATACCAAATACTGTCCTTCTAGTGTAGCCGTAGTTAG
GCCACCACTTCAAGAACTCTGTAGCACCGCCTACATACCTCGCTCTGCTAATCCTGTTACCAGTGGCTGCTGCCAGTGGCGATAAGTCGTGTCTTACCGGGTTGGACTCAAGACGATAGTTACCGGATAAGGCGCAGCGGTCGGGCTGAACGGGGGGTTCGTGCACACAG
CCCAGCTTGGAGCGAACGACCTACACCGAACTGAGATACCTACAGCGTGAGCTATGAGAAAGCGCCACGCTTCCCGAAGGGAGAAAGGCGGACAGGTATCCGGTAAGCGGCAGGGTCGGAACAGGAGAGCGCACGAGGGAGCTTCCAGGGGGAAACGCCTGGTATCTTTA
TAGTCCTGTCGGGTTTCGCCACCTCTGACTTGAGCGTCGATTTTTGTGATGCTCGTCAGGGGGGCGGAGCCTATGGAAAAACGCCAGCAACGCGGCCTTTTTACGGTTCCTGGCCTTTTGCTGGCCTTTTGCTCACATGTTCTTTCCTGCGTTATCCCCTGATTCTGTGG
ATAACCGTATTACCGCCTTTGAGTGAGCTGATACCGCTCGCCGCAGCCGAACGACCGAGCGCAGCGAGTCAGTGAGCGAGGAAGCGGAAGAGCGCCTGATGCGGTATTTTCTCCTTACGCATCTGTGCGGTATTTCACACCGCATATGGTGCACTCTCAGTACAATCTGC
TCTGATGCCGCATAGTTAAGCCAGTATACACTCCGCTATCGCTACGTGACTGGGTCATGGCTGCGCCCCGACACCCGCCAACACCCGCTGACGCGCCCTGACGGGCTTGTCTGCTCCCGGCATCCGCTTACAGACAAGCTGTGACCGTCTCCGGGAGCTGCATGTGTCAG
AGGTTTTCACCGTCATCACCGAAACGCGCGAGGCAGGATATCCGGCAGCCGGCGGAGCGCTGCTTTCTTGGCAAGCGGTCGCCAGCCCCAACGCCAGGGCTGCCAGCCCGAAACAGCGGGGCAAGGCAGCTTGGAAGGGCGATCGCAGCACGGGCATGGCAATGTCTCTC
TGAAGGAATGCAGACCTTATTCGTACAGCCAGGGTTGAATCGTGGGGGTCCAATCACTTAGCTCTGCTGGGCTAAACCAGAGAGCAATTTCCTGTTGTGCTGTTTCGATTGCATCCGAGCCATGGATGATGTTGCGGCCAATATTGACACCAAAATCACCACGGATGGTG
CCCGGTTCTGCCGTCAGCGGATTGGTAGCGCCGATCAACTTGCGAGCAGCCGCCACAACGCCTTCGCCTTCCAAGACGATCGCCACGATCGGCCCAGAGGTGATGAACTCGACGAGGCCATTGAAGAAGGGGCGCTCGCGGTGGACAGCATAGTGCTGTTCGGCCAGCTC
GCGACTGGGCTTCAGCTGCTTTAGGCCCACCAGTTTGAAGCCTTTTTGCTCAAAGCGGCCGATGATCGTACCGACCAAACCCCGCTGAACGCCATCGGGCTTGATGGCAATAAATGTGCGTTCCACAGACATCTAGATAGTCCTCAAGACGAGGCAAGCATTGAGCTTGC
CTTCCTATGGTTCGGGATCACTGGGATTCTTGACAAGCGATCGCGGTCACATCGCTATCTCTTAGGACTTCGCAGCGGGCGAGTCGGATTGACCCGGTAGGGATTTCGCCAGATCAATGCCCGTGGTTTGTTTCAGCTTCTCCAGCAAGCTAGCGATTTGGGTAGCGCTG
CCTTCCCCTTCGCCAATCACAGTGATCGACTCCACGTCGATATCTGGCACGGTGCCTGAAAGCGTGACGAGCAGGGACTCGAAGCTTGCATGCCTGCAGGTCGACTCTAGAGCTTTATGCTTGTAAACCGTTTTGTGAAAAAATTTTTAAAATAAAAAAGGGGACCTCTA
GGGTCCCCAATTAATTAGTAATATAATCTATTAAAGGTCATTCAAAAGGTCATCCACCGGATCAATTCCCCTGCTCGCGCAGGCTGGGTGCCAAGCTCTCGGGTAACATCAAGGCCCGATCCTTGGAGCCCTTGCCCTCCCGCACGATGATCGTGCCGTGATCGAAATCC
AGATCCTTGACCCGCAGTTGCAAACCCTCACTGATCCGCATGCCCGTTCCATACAGAAGCTGGGCGAACAAACGATGCTCGCCTTCCAGAAAACCGAGGATGCGAACCACTTCATCCGGGGTCAGCACCACCGGCAAGCGCCGCGACGGCCGAGGTCTTCCGATCTCCTG
AAGCCAGGGCAGATCCGTGCACAGCACCTTGCCGTAGAAGAACAGCAAGGCCGCCAATGCCTGACGATGCGTGGAGACCGAAACCTTGCGCTCGTTCGCCAGCCAGGACAGAAATGCCTCGACTTCGCTGCTGCCCAAGGTTGCCGGGTGACGCACACCGTGGAAACGGA
TGAAGGCACGAACCCAGTGGACATAAGCCTGTTCGGTTCGTAAGCTGTAATGCAAGTAGCGTATGCGCTCACGCAACTGGTCCAGAACCTTGACCGAACGCAGCGGTGGTAACGGCGCAGTGGCGGTTTTCATGGCTTGTTATGACTGTTTTTTTGGGGTACAGTCTATG
CCTCGGGCATCCAAGCAGCAAGCGCGTTACGCCGTGGGTCGATGTTTGATGTTATGGAGCAGCAACGATGTTACGCAGCAGGGCAGTCGCCCTAAAACAAAGTTAAACATCATGAGGGAAGCGGTGATCGCCGAAGTATCGACTCAACTATCAGAGGTAGTTGGCGTCAT
CGAGCGCCATCTCGAACCGACGTTGCTGGCCGTACATTTGTACGGCTCCGCAGTGGATGGCGGCCTGAAGCCACACAGTGATATTGATTTGCTGGTTACGGTGACCGTAAGGCTTGATGAAACAACGCGGCGAGCTTTGATCAACGACTTTTGGAAACTTCGGCTTCCCC
TGGAGAGAGCGAGATTCTCCGCGCTGTAGAAGTCACCATTGTTGTGCACGACGACATCATTCCGTGGCGTTATCCAGCTAAGCGCGAACTGCAATTTGGAGAATGGCAGCGCAATGACATTCTTGCAGGTATCTTCGAGCCAGCCACGATCGACATTGATCTGGCTATCT
TGCTGACAAAAGCAAGAGAACATAGCGTTGCCTTGGTAGGTCCAGCGGCGGAGGAACTCTTTGATCCGGTTCCTGAACAGGATCTATTTGAGGCGCTAAATGAAACCTTAACGCTATGGAACTCGCCGCCCGACTGGGCTGGCGATGAGCGAAATGTAGTGCTTACGTTG
TCCCGCATTTGGTACAGCGCAGTAACCGGCAAAATCGCGCCGAAGGATGTCGCTGCCGACTGGGCAATGGAGCGCCTGCCGGCCCAGTATCAGCCCGTCATACTTGAAGCTAGACAGGCTTATCTTGGACAAGAAGAAGATCGCTTGGCCTCGCGCGCAGATCAGTTGGA
AGAATTTGTCCACTACGTGAAAGGCGAGATCACCAAGGTAGTCGGCAAATAATGTCTAACAATTCGTTCAAGCCGACGCCGCTTCGCGGCGCGGCTTAACTCAAGCGTTAGATGCACTAAGCACATAATTGCTCACAGCCAAACTATCAGGTCAAGTCTGCTTTTATTAT
TTTTAAGCGTGCATAATAAGCCCTACACAAATTGGGAGATATATCATGAAAGGCTGGCTTTTTCTTGTTATCGCAATAGTTGGCGAAGTAATCGCAACATCCGCATTAAAATCTAGCGAGGGCTTTACTAAGCTGATCCGGTGGATGACCTTTTGAATGACCTTTAATAG
ATTATATTACTAATTAATTGGGGACCCTAGAGGTCCCCTTTTTTATTTTAAAAATTTTTTCACAAAACGGTTTACAAGCATACTAGAGGATCGGCGGCCGCGGATCTGGGCCGCGGGTCATGGCTGCGCCCCGACACCCGCCAACACCCGCTGACGCGCCCTGACGGGCT
TGTCTGCTCCCGGCATCCGCTTACAGACAAGCTGTGACCGTCTCCGGGAGCTGCATGTGTCAGAGGTTTTCACCGTCATCACCGAAACGCGCGAGGCAGCAGATCAATTCGCGCGCGAAGGCGAAGCGGCATGCATTTACGTTGACACCATCGAATGGTGCAAAACCTTT
CGCGGTATGGCATGATAGCGCCCGGAAGAGAGTCAATTCAGGGTGGTGAATGTGAAACCAGTAACGTTATACGATGTCGCAGAGTATGCCGGTGTCTCTTATCAGACCGTTTCCCGCGTGGTGAACCAGGCCAGCCACGTTTCTGCGAAAACGCGGGAAAAAGTGGAAGC
GGCGATGGCGGAGCTGAATTACATTCCCAACCGCGTGGCACAACAACTGGCGGGCAAACAGTCGTTGCTGATTGGCGTTGCCACCTCCAGTCTGGCCCTGCACGCGCCGTCGCAAATTGTCGCGGCGATTAAATCTCGCGCCGATCAACTGGGTGCCAGCGTGGTGGTGT
CGATGGTAGAACGAAGCGGCGTCGAAGCCTGTAAAGCGGCGGTGCACAATCTTCTCGCGCAACGCGTCAGTGGGCTGATCATTAACTATCCGCTGGATGACCAGGATGCCATTGCTGTGGAAGCTGCCTGCACTAATGTTCCGGCGTTATTTCTTGATGTCTCTGACCAG
ACACCCATCAACAGTATTATTTTCTCCCATGAAGACGGTACGCGACTGGGCGTGGAGCATCTGGTCGCATTGGGTCACCAGCAAATCGCGCTGTTAGCGGGCCCATTAAGTTCTGTCTCGGCGCGTCTGCGTCTGGCTGGCTGGCATAAATATCTCACTCGCAATCAAAT
TCAGCCGATAGCGGAACGGGAAGGCGACTGGAGTGCCATGTCCGGTTTTCAACAAACCATGCAAATGCTGAATGAGGGCATCGTTCCCACTGCGATGCTGGTTGCCAACGATCAGATGGCGCTGGGCGCAATGCGCGCCATTACCGAGTCCGGGCTGCGCGTTGGTGCGG
ATATCTCGGTAGTGGGATACGACGATACCGAAGACAGCTCATGTTATATCCCGCCGTTAACCACCATCAAACAGGATTTTCGCCTGCTGGGGCAAACCAGCGTGGACCGCTTGCTGCAACTCTCTCAGGGCCAGGCGGTGAAGGGCAATCAGCTGTTGCCCGTCTCACTG
GTGAAAAGAAAAACCACCCTGGCGCCCAATACGCAAACCGCCTCTCCCCGCGCGTTGGCCGATTCATTAATGCAGCTGGCACGACAGGTTTCCCGACTGGAAAGCGGGCAGTGAGCGCAACGCAATTAATGTAAGTTAGCGCGAATTGATCTGGTTTGACAGCTTATCAT
CGACTGCACGGTGCACCAATGCTTCTGGCGTCAGGCAGCCATCGGAAGCTGTGGTATGGCTGTGCAGGTCGTAAATCACTGCATAATTCGTGTCGCTCAAGGCGCACTCCCGTTCTGGATAATGTTTTTTGCGCCGACATCATAACGGTTCTGGCAAATATTCTGAAATG
AGCTGTTGACAATTAATCATCCGGCTCGTATAATGTGTGGAATTGTGAGCGGATAACAATTTCACACAGGAAACAGACCATGGAggaggaactagATGGCTTCCATTATTGGTAGCGGCTTTTTGATCATTGCACCACTGCTGCATTCGGTAATGGGGAAATGGGCTCTG
CTTGGAATAGTGTTGTTGTCCATTCTGGCATATGCGCTGGGGTCGGTTATTCGCTTCAATATACGCCATGCAGAACCTGAGCTAGCTAGCAATCAACAAAGCAGTATTGTGACGCTTGAAAAAGCAAGTCAATGGGCGCTTGGTGCGGCATATGCCATTTCCGTGGCGTT
TTATATTAGTTTGTTTGCAGCGTTTGTTTTTGATCGTTTATCAATCTCTGATACGACGTACATCAAACTGTTTACCAGTGGGTTGCTTGTCATCATTACGGTGGTCGCTTGGCTCCGTGGTGCTAGGGGGCTAGAGACCATTGAGCTATTCGCCGTGACAATAAAGTTGG
CTATTATAGTAGGTGTGCTCGCAGCGCTGGCAACCTACGACATACAAGTACAAAGTGCTTGGTTTCAACATGAAGCGATACAGGCATTGAGCCATTTTGAAACTGTTAGCATGCTCGCAGGCATGCTCATGGTAACGCAAGGGTTCGAGACAACCCGTTTTATGGGGAAT
AACTACACGCCAGAACAGCGCATTAAAGCGAGTCGTTATGCTCAGTGGATCGCTATTTTTCTCTATGTTGTTTTTATCGGTCTAACGTGTCCTATTTTTCTGGATTTTCCGATCACGGAATTAAACGAAACAACGATTAGCTACACCCTAGGGCAAGCAATATGGGTATT
GCCGATTTTATTACTGGTTGCCGCCACGGCAAGTCAATTAAGTGCAGCATTAGCCGATACTATCGGTGGCGGAGGCCTCTTAAAAGAGCTTTTCCATTTACCTATCTCCCCGCAATTTTACTACGTTTTAGTCATCGCCATCGCAGGTATATTAGTTTGGTCATCCAACG
TATTTGAAATTATTAATCTCGCTTCAAAAGGGTTTGCACTTTATTACCTGATCCAAACAATAATCGCGGTCAAACTCGTGATGCGTCAAAGCACAGGTCAACGCATTAGGTCAACAAAATTACTTGGTCTATCGGTGATCGTTCTCTGTTTATTCTTTGTGATTGGCTGG
TCAATTCCAGCACCTCATAGCTAATAAGGAAAATACCGATGCTATCAGTAGAAACTACACCACCAATTGACCAAATCAAAAACGCGGCTAAGCGCTACGCTATCATTGGTGTTGGAAATCTATTGCAGCGAGATGATGGCGTTGGTGTGCACGCGGTTCGTGCTATGCAA
CCCCTGCTAAAGTCTTATTCAAATGTATCTTGCATTGACGCTGGGACACTGAGCTATGAACTTTTGGAGTGGGTTGCGAGTTCCGATCATACGATAGTCATCGACGCGGCTTATATGCGCTGTCCGCCTGGCACGGTGAGAGTGTTTGAGAACGAGGCAATTGCGGATCA
GTTTCAGCAAGCGACGACCCATTCCGTTCACCAGATCACGTTACGCGACACATTGTTGACCAGCCAAACGTTATATTCAAAACCTTCTGCGGTGACGTTAATTGGCGTAGAGCCGGAGGCGATTGAGTGGGGAACCTCTTTGAGTCCAAAGGTAAATGCAGCGCTATCAT
CGATCATGGATATCGCATTTGCTTGCTTATCAGGCGACAACGTTCAGACCTATAAACCGGCAACCCAAAAGGAGGCACGATGACCGAAGAACTCATTTTTATGACATCAAATGCTGATAAAACGGGCAACGTAATGCCGCTTTTGCATCAGATACGTCATGCGTTGTCTC
AGTTAATTGAACGACAAGAACAAACCACGATTGATCTGCGACGATTGCCTTTAAGCGCTAGCGAAGAAGCGCAGTTAGAAGCTTTTCTTGGGCATGGAGAGGTTAAAGCTGATATTCAGGCGCTGGGTGATACGGTGCTAATCGAATCACGCTACGCAGGCGTTTGGCTT
GAAATTCACTATAACGAAGATGTGGAGATCATGGGCAAATACGTTCATATCTGTACCTGTCCACCAATCATAAAATCTCAGCCAGAAGATATGGTGTTATCGCTCAGCAATATAGTGTCTGACATCCATTCGTTGTCTCATCAATCTTCTGATGAAACGGCTAAGGAGGA
TTGACATGCGAGAGTAGGGAACTGCCAGGCATCAAATAAAACGAAAGGCTCAGTCGAAAGACTGGGCCTTTCGTTTTATCTGTTGTTTGTCGGTGAACGCTCTCCTGAGTAGGACAAATCCGCCGGGAGCGGATTTGAACGTTGCGAAGCAACGGCCCGGAGGGTGGCGG
GCAGGACGCCCGCCATAAACTGCCAGGCATCAAATTAAGCAGAAGGCCATCCTGACGGATGGCCTTTTTGCGTTTCTACTACTAGTTGACAATTAATCATCCGGCTCGTATAATGTGTGGAATTGTGAGCGGATAACAATTTCACACATACTAGAAAGAGGAGAAATACT
AGATGGCGCTACCAACATTAAACAAGCAGTTACAAGCGTCCGGTATCTCAAGACGCACATTTCTTAAGTTTTGCGCTACAACGGCGTCTTTACTGGCATTGCCGCAAAGCGCTGTTGCCGATTTGGCGACTGCCCTTGGCAATGCGAGAAGACCCTCTGTGATTTGGTTG
CCCTTTCAAGAGTGCACAGGGTGTACCGAAGCAATATTGCGCTCTCATGCTCCCACATTGGAAAGCCTTATTTTCGATCATATTTCGTTGGATTATCAGCATACGATAATGGCTGCTGCGGGAGAGCAAGCTGAAGACGCTAGGCGTGCGGCGATGAACGCGCACAAAGG
GCAATATTTGTTGTTGGTTGATGGTTCGGTTCCGGTGGGTAACCCAGGATACTCAACGATCAGTGGCATGAGTAATGTCGATATGCTGAGAGAATCGGCAAAAGATGCTGCGGGTATTATTGCCATCGGTACCTGCGCGTCTTTTGGCGGGATCCCTAAAGCAAACCCAA
ATCCGACGGGGGCAGTGGCAGTAAGCGACATTATTACAGACAAGCCAATCGTAAACATTTCAGGCTGTCCGCCACTGCCTATCGCGATTACAGCTGTGTTGGTTCATTACCTGACGTTTAAGCGTTTCCCTGATCTCGACGAATTACAACGCCCACTCGCTTTTTTTGGT
GAAAGCATCTGTGACAGATGTTATCGACGCCCATTTTTTGAACAACGTAAATTTGCAAAATCGTTTGATGATGAGGGGGCAAAAAATGGGTGGTGTTTGTTCGAACTTGGTTGTAAAGGGCCCGAAACCTTTAACGCATGTGCAACGGTTAAATGGAATCAAGGCACGAG
```

TTTTTGTATCGAATCTGGCCATCCGTGTCTTGGTTGCTCTGAGCCCGATTTCTGGGATAAAAGCAGCTTTTACCAAGCCTTGGGTCCATGGGAGTGGTACAAATCCAAACCCGGCAAAGGTGCACAGAAGCATGCTGGGAAAAACTCATAAGATAATAGGCAAGGCTCAT
GGAAAATACAGCAAGTAACAACCGGTTAGTCGTCGATCCTATCACTCGAATTGAAGGGCATCTTCGAATAGAAGCTGAAATGGATGGGAATACCATCAAACAGGCGTTCTCATCAGGCACGTCTGTTCGGGGAATTGAACTGATTTTACAAGGCAGAGATCCGCGTGACG
CTTGGGCTTTTGCGCAACGTATCTGTGGCGTCTGCACGCTGGTGCATGGTATGGCATCGGTGCGTGCTGTCGAAGATGCAATTAGAAAAGCTTGGCGGTCAAACGCAAAATTAGGGGTTGCCATTGGAAAGCCCTCCATGACATCTATGCCAAAAGGACCGATGCAACAT
GGTAAAAAAGGGCACCGACAGTCACGTACTTCAATAGGCGTACTGAGTGAAGCAGAAATGGCTATCCCTCAAAATGCACAACTGATAAGAAACATCATGATTGCAACCCAATATGTGCATGATCACGTGATGCATTTTTATCACTTGCATGCCCTAGATTGGGTTGACGT
TGTTTCTGCACTAGATGCAGATCCAACGAGAACCGCTACGCTCGCCGGTCAATTGAGTGATTATCCTCGTTCATCGCCGGGATATTTCAAAGATGTGAAGCAAAAAGTCAAAACGCTGGTTGAGTCTGGGCAGCTAGGGATATTCAGTAACGCGTATTGGGGGCATCCTG
GCTATAAACTGCCACCTGAAGTTAACTTGATGGCATTAGCACATTATTTAGATGCGCTAACGTGGCAGCGTGAAGTTGTAAAAGTTCACACCATATTTGGAGGGAAAAACCCTCATCCTAATTTTGTCGTTGGCGGCGTGCCTTCACCGATTAATCTCAATGCGTCAACG
GGTATTAACACGAGTCGATTAGTGCAACTACAAGATGCTATCACGCAAATGAAGAGCTTTGTCGATCAGGTGTATTACCCCGATATTGTGGCGATTGCGGGTTATTACAAAGAGTGGGGGACACGAGGGGAAGGGCTGGGTAACTTTCTTACCTATGGAGACTTACCTAT
GACATCAATGGATGACCCTGATTCTTTCTTGTTTCCACGAGGTGCAATACTTGGTCGAGACTTGAGTAAAGTGCATGACCTTGATCTAGATGATCCCTCTGAAATTCAAGAATTCGTCTCTTCCTCCTGGTATCGATATAGTGGAGGGAACGCAAGTGGTTTACACCCTT
TTAATGGACAAACAACACTCGAGTATACTGGTCCGAAACCGCCTTACAAGCACCTAAATGTAGGGGCTGAATATTCATGGTTGAAAAGTCCGCGTTGGAAAGGCCATGCGATGGAGGTGGGACCGCTGGCTCGCGTGCTAATGATGTATGCTAAAAAAGATGCCGCTGCG
CAAGACATCGTTAATCGATCTCTTTCTATCTTGGATTTAGAGACCTCTGCACTTTTCTCCACACTCGGTAGGACGCTCGCCAGAGCAGTGGAAACAAAAATTGTGGTTAACCAGCTACAGTCTTGGTATGACCAACTATTGGATAATATCGCAAAGGGCGATACCGATAC
GTTTAACCCTCTGTATTTTGACCCTACCAATTGGCCAATTAAAGGCCAGGGGGTAGGCGTGATGGAAGCGCCTCGTGGCGCGTTGGGGCATTGGTTGGTCATGCAAAATGGCAAAATTGAGAATTACCAATGTGTCGTGCCTACGACATGGAACGCTGGACCTCGAGATC
CCAACTCACAGGCAGGTGCTTATGAAGCCGCTCTGCAAGATAAACATACGCTACATGATCCTGACCAACCTTTAGAGATTTTGCGAACACTTCATAGTTTTGACCCCTGCTTAGCATGTGCCGTGCACGTAATGGACGAAACAGGGGAAGAGCGTTTGCGTCTAAAAGTT
CGTTAAgatcctctacgccggacgcatcgtggccggTCGAGCTCAAGGAGGAATAACACATGCGAGAGTAGGGAACTGCCAGGCATCAAATAAAACGAAAGGCTCAGTCGAAAGACTGGGCCTTTCGTTTTATCTGTTGTTTGTCGGTGAACGCTCTCCTGAGTAGGACA
AATCCGCCGGGAGCGGATTTGAACGTTGCGAAGCAACGGCCCGGAGGGTGGCGGGCAGGACGCCCGCCATAAACTGCCAGGCATCAAATTAAGCAGAAGGCCATCCTGACGGATGGCCTTTTTGCGTTTCTACTACTAGTTGACAATTAATCATCCGGCTCGTATAATGT
GTGGAATTGTGAGCGGATAACAATTTCACACATACTAGAAAGAGGAGAAATACTAGATGTGTCTCGCTATCCCCATGAAAGTCATTGCAATTAAAGGCTTTAACGCAACGTGTGAAGCGAAAGGTGTCTCTCGCGAGGTGAGTTTACATTTAGTGCAAGGGCTAGAGGTC
AAAGTTGGCGACTATGTGATGGTACATGTTGGTTATGCTCTCCAAGTGATCACCTATGATGAAGCGCAGGTGACATGGGAGATGCTCGATCAGGTCATTGCTTACGATGCATGAAATTAGTCTTTGCTATAGCTTGCTTGACACAGTCGCTGTTCACCAGCGAGCAAACA
TGAATAAGTCTGTCAGTCTGGTTCACGTCAAAGTCGGCCCACTGTCAGGTGTTGAGCCTGACTTGCTGCACCATGCGTTCTTGGCGTGTAGAACTCACACCATTGGCGATCAGGCAACGCTTAGAATTGACACAAGCGCTATCAAGATACGCTGTAAATTGTGTGGCGAA
TTCAGCCGAGTTTCGGTGAATAGCATCCTATGTGCAAGTTGCGGTGCTTGGCAAACCGACCTCATTGAGGGCGATGAATTTATATTGCAACGAATAGAGTTTATCGCACCTGACAATTTTACAGAATCAAAGGAGAGAAAACATGTGCGGTAACTGTGGTTGCGAAACGC
AAGTAAAATCAGAGAAAACTGACAAGCCATCAGCAAATATCGTTGAGGTTCACGCAAATTTAAAAAGCAACAATGACAACCAAGCGATAGCAAACCGTGCTTTATTTGACGCACATAATGTACTAGTGATTAATTTGATGTCATCCCCAGGCAGTGGAAAAACCAGGCTA
CTCGAAGAAACGATACGTGCGCTTAAGTCGCAATACGCGATGGCGGTGATTGAAGGCGACCTCGAAACTGAAAATGACGCAAATAGAATTAGACGTCATGGTGTGCAGGCGGAGCAAATCGCCACGGGCCAAGGGTGTCACTTGGACGCCAGTATGGTGCAAAAGGTACT
TGGAAGATTTAGCTTACAAAAACTAGACGTTTTGTTTATAGAGAATGTTGGCAATTTGATTTGTCCAGCGTGTTTTGATTTAGGACAGCACTTAAACATTATTTTGCTGTCCGTACCAGAAGGTGATGATAAGCCTGAAAAGTACCCAGTGATGTTTCGTGCCGCGGACG
TCATGCTTATTTCTAAGACCGATTACTTGCAATTTCACGATGAATTCAATGTACCTCGCGCGATTGAATCATTTCGTAAAGTGGGCAACGATGCGCCCGTTCTCGAAGTGTCTTCATTGAAAAGTCAACATCTAGAGCGTTGGTTTTCTTATTTTACGCACGCCATTACA
GCTCATCAGTCTCAGTCGTTACAAAATAGCGGAGTCTAACATGCGAGAGTAGGGAACTGCCAGGCATCAAATAAAACGAAAGGCTCAGTCGAAAGACTGGGCCTTTCGTTTTATCTGTTGTTTGTCGGTGAACGCTCTCCTGAGTAGGACAAATCCGCCGGGAGCGGATT
TGAACGTTGCGAAGCAACGGCCCGGAGGGTGGCGGGCAGGACGCCCGCCATAAACTGCCAGGCATCAAATTAAGCAGAAGGCCATCCTGACGGATGGCCTTTTTGCGTTTCTACTACTAGTTGACAATTAATCATCCGGCTCGTATAATGTGTGGAATTGTGAGCGGATA
ACAATTTCACACATACTAGAAAGAGGAGAAATACTAGATGTACAGTGTTGAATCGTTGCTAAGAGACATTAAGAATACCTCGCTTTCAAAACCGTTTCGTATTCTTAATGTTTGCGGTGGCCATGAGCGTGCGATTACGCGCGCTGGATTTAGAACGTTGTTTCAGCACA
ATATTCATCTGATACCTGGGCCAGGGTGCCCCGTTTGTATTTGCCCCGAAGAGGATATTGCATACGCGATACACTTAGCCATTAACGAAAATGTCGTTATCGTTAGTTTTGGTGACATGCTCCGCGTCCCAGTCGAACACGAAATGGGTGGCTGTAGCTCATTGATAGAC
GCAAAAAATCAAGGGGCCGATATCCGTCCGATATCTTCTCCTCAAGAAGCGGTATCTATAGCATTGGAAGAACCTCGTAAGGTTATTGTGTTTTTTGCTGTTGGTTTTGAAACGACTATGGCACCGATTGCAGCAACACTGCTTAATGATTTACCCAATAACTTTAAAGT
ATTATTGTCTGGACGACTTACTTGGCCAGCAGTTGCCCATGTGCTTGAGAGTCAACCGAATACTTTTGATGCACTGATTGCGCCAGGCCACGTTGCGACAATTATGGGTAGTGAAGAATGGCAGTTTGCAATCGACCATCATAATTTGCCTGTCAGTATTGCTGGTTTTC
ATCCAGAAAGTTTGCTGTTGTCGCTCCAAACGCTGCTCGGTAATTGTTCAAATAAAGTCGTCACACTGAGTAACCGCTATCCCGAAGTGGTAAAGCAAAACGGCAATGCAGCAGCTAAAGCAATAATCAATAAAGCGTTCACTATCGTTGACGCACATTGGCGAGGAATT
GGTGTTATTCCTGGTTCTGGATTTTCCTTTGCTTCGAGACTATCGCACCTAGACGCCACAAACGATTATGCGCCAGTCGATTTCCCTTCGCAATGTGCGCAAAACGTTCCTGAAACCACATCGCCCTGTGAAAAAGTGATCCTCGGAAAAATGGCACCTGATGCGTGCCC
GTTTTTTGGTCAGGAATGCAAACCAGCATCCCCTAAAGGCGCATGTATGGTGTCTGATGAGGGGGCTTGCCGAATTTGGTATAGCTCTGGTGAGCGCTCGATAACAAATGTTATCAAAAAGGGGAATACGCTAAAGGTGGAGATGAAGTGATGACAACTGCCCGCACATA
TGTTGCCAATACGAATAAGAGTAAAACTATCGCCTATGAGTTGAACATTACTGGAGTCATCCAGGGGGTTGGGTTCAGACCGTTAGTTTACTGCTTAGCGTATGAAAAAAGTATTGTTGGTTGGGTACAAAACGACTGTGGATGTGTGCGAATACACGCTGAAGGCACTG
AGCTAGACGTTGAACAATTTGTATTCGACTTACTGAACAATGGGTCGCTAGTTTCCATATCCTTGCTGGAAAAAAAGCAGGTTAAACTGAGTAATGTAGACTCGTTCACGATAAAAGAAAGCGGTCATGATATAGCGTGCGGGGCGATATCAATCCCCAAAGATGTCTAT
TTGTGTGGAGCGTGTAAAGCTGAGTTACTGTCAAATACTAACCGACGTGGCACCTATAGCTTTATCGCTTGCAGTAAATGTGGTCCACGATTTTCTATGCTGCGAGCAATGCCATACGACAGAAAGAACATGAGTATGTCAGCATTTCCAATGTGTGAGTTATGTCGTAA
AGAATATCAATCGCCAACTGATAGGCGCTTTCATGCTCAACCAATAAGCTGTCGCGTATGCGGGCCTGAAGTATTTTGTTCAACTGTCGGCGGGCGCGTGGTCTCGCAAGGCGATGTTGATGTTGTAGCAACGGTTGTCGCGTATTTGAATCAAGGCAAAATTGTCGCGT
TAAAAAGTATAGGCGGTTATCACTTGCTCTGTGACGCACAAAGCACTGAAGCGGTAGACCTTTTACGCCAGCGTAAAAATAGACCAGATAAACCTTTCGCGGTCATGCTCCCAGAGCCTCGTGCAGACGAAACAAGTGAGGATTGGTTAGATAAATGCGTTGCTGTAGAC
AGTCATCAAAGAGCGCTCTTACTAAGCCCCGTGCGACCCATTTTACTTGCAAAAAAGCAATCGAATGTACCCATTGCAGATAACGTAGCGCCCATGCTTCGTGATTTGGGGGTGATGTTGCCGTGCAGCGGACTGCATTTACTCATTATGCAGCAATTCAATCGCCCCAT
GGTGGCTACGTCAGGAAATATTTACGGTGAACCGATGGTCACATCGACGTCATCGGCACAGCAACACCTTTCGATGTTGGCAGACATCTTCGTGCACCATAATCGAGACATTTTTCACAAACTGGATGATTCTGTGCTGCGAACGCTTAAGTCAGGCACCATTCCAATTC
GCCTGGGGCGGGGTATAAGTCCGATAGAATTATCATTGCCATATACTGTGCAGGAACCAATGCTTGCTGTTGGCGCGAATCAGAAAAATACAGTCGCAATAGCTTTGAACAATCGACTTATCGTAACACCGCATATTGGCGACCTCGATTCTCCGATAATGCAAGCGCAC
TTTGAAAAATGTATCAGTGACTTTCAAGCTCTCTATCATGTTCATCCACAGCGTATTCTTTGCGATAGCCATCCTGGCTATATCAGTTCCCGTTGGGCCAAAGGCAGTGAACTACCGACTACTAAAGTTTTACATCACCACGCTCATGCATCAGCGTGGGCATTAGCTGC
TGAAATATCGACACCAAGCTTGGTTTTCGTGTGGGATGGAACGGGATTAGGCGAGAATGGCGAGATGTGGGGAGGGGAAGTTTTTTGTGGGACGCCGGGCAATTGGAAACGTGTTGCCTCATTAGCGCCTATGAAGTTGCAAGGCGGCAACAAGGTTCCAAATCAACCAT
GGCGAAGTGCAGCATCACTAGTGTGGGGTACTAATTTCACCGGCATTGATTTTAAACCATTTGATCCACAACAATTAAGCTACAACGCATGGCAAAACGAACTCAATTGTCATGTTAGTAGTTCAATGGGAAGACTGTTCGACGCGGCGGCATTTGCCTGCTGTGAACAG
AGCGAAGTCTCTTATGAAGGGCAGGCGCCGATGATGTTGGAAAGCGTTGCGTCGACGCCCTCTAAGGTTATGCCGTTAACGTTGGTCCAGAAAAACGACGAATTTATTCAAATGGATTGGCGAACACTGGTACCGGAACTATTTGATTTCAAAGTGTCTCCTCAAACTCG
TGCTGCAAATTTCCACGCTTCGTTAATTGAAACGGTATGGGAGGTAACCCTACATTGCCGTAAAAAGTTTAATTTTGACCACATTGGATTGGCAGGAGGCGTCTTCCAAAATCGCATTTTGTGTGACGGTATATCACAACGGTTTTTACAATCAGACATTCCTGTGGTTA
TTCCAAGTAGTTTGCCATTAAACGATGCCGCAATAAGTGTTGGGCAAATCCACGAATACTGTTCACGGAGGTCATAATGAAAAGCGCAGAGGGCAATAATCATTCATCATCAGAGCCGTTGGGCACGGAATCTATTCAATTAGCTCAGGGGAACGGCGGAACACTGACAA
AAAGACTCATTGATCATGTTTTTAAAAATAAAGCGAACAAAGATCTAGATCTGCTTCATGATGCCGCCACAGTAACTTTTGATGGTCCTTATCTGAGTATAACAACGGATAGCTTCGTTGTATCCCCCCCAACATTTCCAGGCGGCAATGTAGGCGCGTTGAGTGTTTAC
GGTACCGTCAATGATTTAGCAGTCGTTGGGGCAACACCACGGTACATCAGTACTGCATTTATTATTGAAGAAGGCTTTTCCCTTTCAACATTGAAACAAATCGTTAATGGAATGCATCAAGCTGCCGAAGAAACGAAGGTCGCCATTGTAACAGGTGATACGAAAGTTGT
CCCGAAAGGCAGTGGTGGCGGCGTTTTTATCAATACAACGGGTGTTGGAGACAGCCGAGATGCGCCGATTTGGGATACCAGTCTTATAAAGGCAGGCGATCATGTACTGGTTAGTGGTTCTGTAGGTGATCACGGTGCGTGTGTCCTGCTTGCTCGCGAGGACTATGGTT
TGCAAGGTCAGCTAAAGTCTGATTGTGGAAGCGTTGTTCCTCTTATTGATCCAATCAAGCATTTGGAAGGTGTTCGTTTTGTGCGTGACCCAACTCGTGGAGGGTTGTCAGTATTACTCCATGACGTTGCTAATGAAACAGGCTTCGATATCGAGTTAATTGAAAATAAT
ATTCCGGTGCGACCTGAAGTGGCAAGCGTGTGTGAAATATTAGGCTTTGATCCGTTTGTTTTAGCGTGTGAGGGACGCATTGTTGCTGTTGTTGCCCCTGACATAAGCGGTAAGGTGCTAGAACACTGGCGGAGCATTCGAAACGGGGAGCAGGCCGAACACATTGGTGT
AATAGTAAAAAACACCGAATCAAAAGGGCGCGTGACCATTGTTACGCCAATGGGAGGAAGACGTTTCATGAATGAACTCGAAGATGAGCCGCTTCCAAGGATCTGCTGAGATCCTCTAGAGTCGACCTGCAGGCATGCAAGCTTGGCTGTTTTGGCGGATGAGAGAAGAT
TTTCAGCCTGATACAGATTAAATCAGAACGCAGAAGCGGTCTGATAAAACAGAATTTGCCTGGCGGCAGTAGCGCGGTGGTCCCACCTGACCCCATGCCGAACTCAGAAGTGAAACGCCGTAGCGCCGATGGTAGTGTGGGGTCTCCCCATGCGAGAGTAGGGAACTGCC
AGGCATCAAATAAAACGAAAGGCTCAGTCGAAAGACTGGGCCTTTCGTTTTATCTGTTGTTTGTCGGTGAACGCTCTCCTGAGTAGGACAAATCCGCCGGGAGCGGATTTGAACGTTGCGAAGCAACGGCCCGGAGGGTGGCGGGCAGGACGCCCGCCATAAACTGCCAG
GCATCAAATTAAGCAGAAGGCCATCCTGACGGATGGCCTTTTTGCGTTTCTACAAACTCTTTTGTTTATTTTTCTAAATACATTCAAATATGTATCCGCTCATGGCGGCCCAGATCCCCGGGTACCGAGCTCGAATTTCGAGCTTCTGGAGCAGGAAGATGTCGCGGGCA
TTAGCACCAGCGGTCTGCCAAGCCTCCGCCAGCCGTTGGGTCCCTTCCGCTTGAGCTTTTCCATCTTCGACGATACGGGCGGCGGCCCCCGCGCTTCCGCGATCGCCCGTTTACAAGCTGCCTCAGCTGGGGCGATCACATCGGCTTGAAGTTGCTGCTGCACCTGTTT
GATCCGCTCCTGCTGCACAGGGAGTTCTGCTTGGCTACGAGCGACTTCGGTAGCAATGTCCGCTTCAGCTTCGGCCACCACCGCTTCGCGCCGCGTCAACGCATCCTGAATCCGGCGCTCGGCCTCGGCTTGGGCGATCGCTACATCGCGATCGATCCGACGCAGGGCCG
TGATCTTGTCATTTTCGGCCGTTTGGATCGCAGAGGCAGCCTGGGCATCGGCTTCAGCAATTCGGGCATCTCGCTGCAGATCAGCCCGCTGCTTGCGTCCACTAGCCGAGAGATAACCGACCTCATCGGAAATGTTCTGGACTTGCAGCGTATCGAGGACTAGACCCAGC
TGCTCAAGGTCATCCTCCGCCTCTTCCAGCAGACTTTTGGCAAAGGCAATTTTGTCCTCGTTGATCTGCTCCGGCGTGAGGCTGGCTAAAACACCACGCAAGTTGCCTTCGAGGGTCTCCTTGGCAATTTGCTCGATTTCCTTACGGTTTTTGCCAAGCAGCCGCTCGAT
CGCGTTGTGGATGGTCGGTTCTTCCCCAGCAATCTTGATATTGGCAACGCCTTCAACAGTCAGGGGAATGCCGCCCTTGGAGAAGGCATTGGAAACGCGCAACTCAATGATCATGTTGGTCAGATCCATGCGGAGCGCTTTTTCCAGCAGAGGTACCCGCAGGCTGCTGC
CGCCCTTGACCAAGCGATAGCCAACTCGGCGGCCATCACTACTGCGGCGACTACTGCCAGCAAAGATCAAAATTTCACTGGGTTGGCAGATGTAGTAGAGATTGCGCAGGACTAAGCTGCCAGCCCCGGCGACTCACCAGTCACAGAAAAGCATCTTACGGATGGCATGA
CAGTAAGAGAATTATGCAGTGCTGCCATAACCATGAGTGATAACACTGCGGCCAACTTACTTCTGACAACGATCGGAGGACCGAAGGAGCTAACCGCTTTTTTGCACAACATGGGGGATCATGTAACTCGCCTTGATCGTTGGGAACCGGAGCTGAATGAAGCCATACCA
AACGACGAGCGTGACACCACGATGCCTGCAGCAATGGCAACAACGTTGCGCAAACTATTAACTGGCGAACTACTTACTCTAGCTTCCCGGCAACAATTAATAGACTGGATGGAGGCGGATAAAGTTGCAGGACCACTTCTGCGCTCGGCCCTTCCGGCTGGCTGGTTTAT
TGCTGATAAATCTGGAGCCGGTGAGCGTGGGTCTCGCGGTATCATTGCAGCACTGGGGCCAGATGGTAAGCCCTCCCGTATCGTAGTTATCTACACGACGGGGAGTCAGGCAACTATGGATGAACGAAATAGACAGATCGCTGAGATAGGTGCCTCACTGATTAAGCATT
GGTAACTGTCAGACCAAGTTTACTCATATATACTTTAGATTGATTTAAAACTTCATTTTTAATTTAAAAGGATCTAGGTGAAGATCCTTTTTGATAATCTCATGACCAAAATCCCTTAACGTGAGTTTTCGTTCCACTGAGCGTCAGACCCCGTAGAAAAGATCAAAGGA
TCTTC

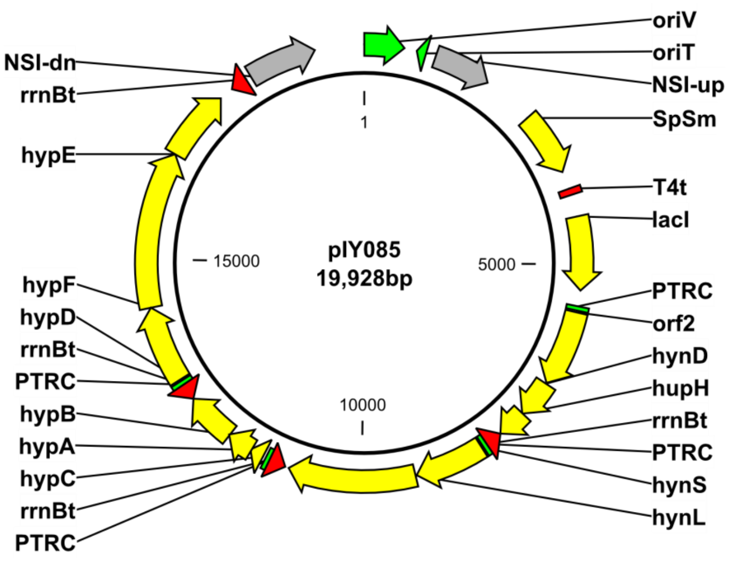

TTGAGATCCTTTTTTTCTGCGCGTAATCTGCTGCTTGCAAACAAAAAAACCACCGCTACCAGCGGTGGTTTGTTTGCCGGATCAAGAGCTACCAACTCTTTTTCCGAAGGTAACTGGCTTCAGCAGAGCGCAGATACCAAATACTGTCCTTCTAGTGTAGCCGTAGTTAG
GCCACCACTTCAAGAACTCTGTAGCACCGCCTACATACCTCGCTCTGCTAATCCTGTTACCAGTGGCTGCTGCCAGTGGCGATAAGTCGTGTCTTACCGGGTTGGACTCAAGACGATAGTTACCGGATAAGGCGCAGCGGTCGGGCTGAACGGGGGGTTCGTGCACACAG
CCCAGCTTGGAGCGAACGACCTACACCGAACTGAGATACCTACAGCGTGAGCTATGAGAAAGCGCCACGCTTCCCGAAGGGAGAAAGGCGGACAGGTATCCGGTAAGCGGCAGGGTCGGAACAGGAGAGCGCACGAGGGAGCTTCCAGGGGGAAACGCCTGGTATCTTTA
TAGTCCTGTCGGGTTTCGCCACCTCTGACTTGAGCGTCGATTTTTGTGATGCTCGTCAGGGGGGCGGAGCCTATGGAAAAACGCCAGCAACGCGGCCTTTTTACGGTTCCTGGCCTTTTGCTGGCCTTTTGCTCACATGTTCTTTCCTGCGTTATCCCCTGATTCTGTGG
ATAACCGTATTACCGCCTTTGAGTGAGCTGATACCGCTCGCCGCAGCCGAACGACCGAGCGCAGCGAGTCAGTGAGCGAGGAAGCGGAAGAGCGCCTGATGCGGTATTTTCTCCTTACGCATCTGTGCGGTATTTCACACCGCATATGGTGCACTCTCAGTACAATCTGC
TCTGATGCCGCATAGTTAAGCCAGTATACACTCCGCTATCGCTACGTGACTGGGTCATGGCTGCGCCCCGACACCCGCCAACACCCGCTGACGCGCCCTGACGGGCTTGTCTGCTCCCGGCATCCGCTTACAGACAAGCTGTGACCGTCTCCGGGAGCTGCATGTGTCAG
AGGTTTTCACCGTCATCACCGAAACGCGCGAGGCAGGATATCCGGCAGCCGGCGGAGCGCTGCTTTCTTGGCAAGCGGTCGCCAGCCCCAACGCCAGGGCTGCCAGCCCGAAACAGCGGGGCAAGGCAGCTTGGAAGGGCGATCGCAGCACGGGCATGGCAATGTCTCTC
TGAAGGAATGCAGACCTTATTCGTACAGCCAGGGTTGAATCGTGGGGGTCCAATCACTTAGCTCTGCTGGGCTAAACCAGAGAGCAATTTCCTGTTGTGCTGTTTCGATTGCATCCGAGCCATGGATGATGTTGCGGCCAATATTGACACCAAAATCACCACGGATGGTG
CCCGGTTCTGCCGTCAGCGGATTGGTAGCGCCGATCAACTTGCGAGCAGCCGCCACAACGCCTTCGCCTTCCAAGACGATCGCCACGATCGGCCCAGAGGTGATGAACTCGACGAGGCCATTGAAGAAGGGGCGCTCGCGGTGGACAGCATAGTGCTGTTCGGCCAGCTC
GCGACTGGGCTTCAGCTGCTTTAGGCCCACCAGTTTGAAGCCTTTTTGCTCAAAGCGGCCGATGATCGTACCGACCAAACCCCGCTGAACGCCATCGGGCTTGATGGCAATAAATGTGCGTTCCACAGACATCTAGATAGTCCTCAAGACGAGGCAAGCATTGAGCTTGC
CTTCCTATGGTTCGGGATCACTGGGATTCTTGACAAGCGATCGCGGTCACATCGCTATCTCTTAGGACTTCGCAGCGGGCGAGTCGGATTGACCCGGTAGGGATTTCGCCAGATCAATGCCCGTGGTTTGTTTCAGCTTCTCCAGCAAGCTAGCGATTTGGGTAGCGCTG
CCTTCCCCTTCGCCAATCACAGTGATCGACTCCACGTCGATATCTGGCACGGTGCCTGAAAGCGTGACGAGCAGGGACTCGAAGCTTGCATGCCTGCAGGTCGACTCTAGAGCTTTATGCTTGTAAACCGTTTTGTGAAAAAATTTTTAAAATAAAAAAGGGGACCTCTA
GGGTCCCCAATTAATTAGTAATATAATCTATTAAAGGTCATTCAAAAGGTCATCCACCGGATCAATTCCCCTGCTCGCGCAGGCTGGGTGCCAAGCTCTCGGGTAACATCAAGGCCCGATCCTTGGAGCCCTTGCCCTCCCGCACGATGATCGTGCCGTGATCGAAATCC
AGATCCTTGACCCGCAGTTGCAAACCCTCACTGATCCGCATGCCCGTTCCATACAGAAGCTGGGCGAACAAACGATGCTCGCCTTCCAGAAAACCGAGGATGCGAACCACTTCATCCGGGGTCAGCACCACCGGCAAGCGCCGCGACGGCCGAGGTCTTCCGATCTCCTG
AAGCCAGGGCAGATCCGTGCACAGCACCTTGCCGTAGAAGAACAGCAAGGCCGCCAATGCCTGACGATGCGTGGAGACCGAAACCTTGCGCTCGTTCGCCAGCCAGGACAGAAATGCCTCGACTTCGCTGCTGCCCAAGGTTGCCGGGTGACGCACACCGTGGAAACGGA
TGAAGGCACGAACCCAGTGGACATAAGCCTGTTCGGTTCGTAAGCTGTAATGCAAGTAGCGTATGCGCTCACGCAACTGGTCCAGAACCTTGACCGAACGCAGCGGTGGTAACGGCGCAGTGGCGGTTTTCATGGCTTGTTATGACTGTTTTTTTGGGGTACAGTCTATG
CCTCGGGCATCCAAGCAGCAAGCGCGTTACGCCGTGGGTCGATGTTTGATGTTATGGAGCAGCAACGATGTTACGCAGCAGGGCAGTCGCCCTAAAACAAAGTTAAACATCATGAGGGAAGCGGTGATCGCCGAAGTATCGACTCAACTATCAGAGGTAGTTGGCGTCAT
CGAGCGCCATCTCGAACCGACGTTGCTGGCCGTACATTTGTACGGCTCCGCAGTGGATGGCGGCCTGAAGCCACACAGTGATATTGATTTGCTGGTTACGGTGACCGTAAGGCTTGATGAAACAACGCGGCGAGCTTTGATCAACGACTTTTGGAAACTTCGGCTTCCCC
TGGAGAGAGCGAGATTCTCCGCGCTGTAGAAGTCACCATTGTTGTGCACGACGACATCATTCCGTGGCGTTATCCAGCTAAGCGCGAACTGCAATTTGGAGAATGGCAGCGCAATGACATTCTTGCAGGTATCTTCGAGCCAGCCACGATCGACATTGATCTGGCTATCT
TGCTGACAAAAGCAAGAGAACATAGCGTTGCCTTGGTAGGTCCAGCGGCGGAGGAACTCTTTGATCCGGTTCCTGAACAGGATCTATTTGAGGCGCTAAATGAAACCTTAACGCTATGGAACTCGCCGCCCGACTGGGCTGGCGATGAGCGAAATGTAGTGCTTACGTTG
TCCCGCATTTGGTACAGCGCAGTAACCGGCAAAATCGCGCCGAAGGATGTCGCTGCCGACTGGGCAATGGAGCGCCTGCCGGCCCAGTATCAGCCCGTCATACTTGAAGCTAGACAGGCTTATCTTGGACAAGAAGAAGATCGCTTGGCCTCGCGCGCAGATCAGTTGGA
AGAATTTGTCCACTACGTGAAAGGCGAGATCACCAAGGTAGTCGGCAAATAATGTCTAACAATTCGTTCAAGCCGACGCCGCTTCGCGGCGCGGCTTAACTCAAGCGTTAGATGCACTAAGCACATAATTGCTCACAGCCAAACTATCAGGTCAAGTCTGCTTTTATTAT
TTTTAAGCGTGCATAATAAGCCCTACACAAATTGGGAGATATATCATGAAAGGCTGGCTTTTTCTTGTTATCGCAATAGTTGGCGAAGTAATCGCAACATCCGCATTAAAATCTAGCGAGGGCTTTACTAAGCTGATCCGGTGGATGACCTTTTGAATGACCTTTAATAG
ATTATATTACTAATTAATTGGGGACCCTAGAGGTCCCCTTTTTTATTTTAAAAATTTTTTCACAAAACGGTTTACAAGCATACTAGAGGATCGGCGGCCGCGGATCTGGGCCGCGGGTCATGGCTGCGCCCCGACACCCGCCAACACCCGCTGACGCGCCCTGACGGGCT
TGTCTGCTCCCGGCATCCGCTTACAGACAAGCTGTGACCGTCTCCGGGAGCTGCATGTGTCAGAGGTTTTCACCGTCATCACCGAAACGCGCGAGGCAGCAGATCAATTCGCGCGCGAAGGCGAAGCGGCATGCATTTACGTTGACACCATCGAATGGTGCAAAACCTTT
CGCGGTATGGCATGATAGCGCCCGGAAGAGAGTCAATTCAGGGTGGTGAATGTGAAACCAGTAACGTTATACGATGTCGCAGAGTATGCCGGTGTCTCTTATCAGACCGTTTCCCGCGTGGTGAACCAGGCCAGCCACGTTTCTGCGAAAACGCGGGAAAAAGTGGAAGC
GGCGATGGCGGAGCTGAATTACATTCCCAACCGCGTGGCACAACAACTGGCGGGCAAACAGTCGTTGCTGATTGGCGTTGCCACCTCCAGTCTGGCCCTGCACGCGCCGTCGCAAATTGTCGCGGCGATTAAATCTCGCGCCGATCAACTGGGTGCCAGCGTGGTGGTGT
CGATGGTAGAACGAAGCGGCGTCGAAGCCTGTAAAGCGGCGGTGCACAATCTTCTCGCGCAACGCGTCAGTGGGCTGATCATTAACTATCCGCTGGATGACCAGGATGCCATTGCTGTGGAAGCTGCCTGCACTAATGTTCCGGCGTTATTTCTTGATGTCTCTGACCAG
ACACCCATCAACAGTATTATTTTCTCCCATGAAGACGGTACGCGACTGGGCGTGGAGCATCTGGTCGCATTGGGTCACCAGCAAATCGCGCTGTTAGCGGGCCCATTAAGTTCTGTCTCGGCGCGTCTGCGTCTGGCTGGCTGGCATAAATATCTCACTCGCAATCAAAT
TCAGCCGATAGCGGAACGGGAAGGCGACTGGAGTGCCATGTCCGGTTTTCAACAAACCATGCAAATGCTGAATGAGGGCATCGTTCCCACTGCGATGCTGGTTGCCAACGATCAGATGGCGCTGGGCGCAATGCGCGCCATTACCGAGTCCGGGCTGCGCGTTGGTGCGG
ATATCTCGGTAGTGGGATACGACGATACCGAAGACAGCTCATGTTATATCCCGCCGTTAACCACCATCAAACAGGATTTTCGCCTGCTGGGGCAAACCAGCGTGGACCGCTTGCTGCAACTCTCTCAGGGCCAGGCGGTGAAGGGCAATCAGCTGTTGCCCGTCTCACTG
GTGAAAAGAAAAACCACCCTGGCGCCCAATACGCAAACCGCCTCTCCCCGCGCGTTGGCCGATTCATTAATGCAGCTGGCACGACAGGTTTCCCGACTGGAAAGCGGGCAGTGAGCGCAACGCAATTAATGTAAGTTAGCGCGAATTGATCTGGTTTGACAGCTTATCAT
CGACTGCACGGTGCACCAATGCTTCTGGCGTCAGGCAGCCATCGGAAGCTGTGGTATGGCTGTGCAGGTCGTAAATCACTGCATAATTCGTGTCGCTCAAGGCGCACTCCCGTTCTGGATAATGTTTTTTGCGCCGACATCATAACGGTTCTGGCAAATATTCTGAAATG
AGCTGTTGACAATTAATCATCCGGCTCGTATAATGTGTGGAATTGTGAGCGGATAACAATTTCACACAGGAAACAGACCATGGAggaggaactagATGGCTTCCATTATTGGTAGCGGCTTTTTGATCATTGCACCACTGCTGCATTCGGTAATGGGGAAATGGGCTCTG
CTTGGAATAGTGTTGTTGTCCATTCTGGCATATGCGCTGGGGTCGGTTATTCGCTTCAATATACGCCATGCAGAACCTGAGCTAGCTAGCAATCAACAAAGCAGTATTGTGACGCTTGAAAAAGCAAGTCAATGGGCGCTTGGTGCGGCATATGCCATTTCCGTGGCGTT
TTATATTAGTTTGTTTGCAGCGTTTGTTTTTGATCGTTTATCAATCTCTGATACGACGTACATCAAACTGTTTACCAGTGGGTTGCTTGTCATCATTACGGTGGTCGCTTGGCTCCGTGGTGCTAGGGGGCTAGAGACCATTGAGCTATTCGCCGTGACAATAAAGTTGG
CTATTATAGTAGGTGTGCTCGCAGCGCTGGCAACCTACGACATACAAGTACAAAGTGCTTGGTTTCAACATGAAGCGATACAGGCATTGAGCCATTTTGAAACTGTTAGCATGCTCGCAGGCATGCTCATGGTAACGCAAGGGTTCGAGACAACCCGTTTTATGGGGAAT
AACTACACGCCAGAACAGCGCATTAAAGCGAGTCGTTATGCTCAGTGGATCGCTATTTTTCTCTATGTTGTTTTTATCGGTCTAACGTGTCCTATTTTTCTGGATTTTCCGATCACGGAATTAAACGAAACAACGATTAGCTACACCCTAGGGCAAGCAATATGGGTATT
GCCGATTTTATTACTGGTTGCCGCCACGGCAAGTCAATTAAGTGCAGCATTAGCCGATACTATCGGTGGCGGAGGCCTCTTAAAAGAGCTTTTCCATTTACCTATCTCCCCGCAATTTTACTACGTTTTAGTCATCGCCATCGCAGGTATATTAGTTTGGTCATCCAACG
TATTTGAAATTATTAATCTCGCTTCAAAAGGGTTTGCACTTTATTACCTGATCCAAACAATAATCGCGGTCAAACTCGTGATGCGTCAAAGCACAGGTCAACGCATTAGGTCAACAAAATTACTTGGTCTATCGGTGATCGTTCTCTGTTTATTCTTTGTGATTGGCTGG
TCAATTCCAGCACCTCATAGCTAATAAGGAAAATACCGATGCTATCAGTAGAAACTACACCACCAATTGACCAAATCAAAAACGCGGCTAAGCGCTACGCTATCATTGGTGTTGGAAATCTATTGCAGCGAGATGATGGCGTTGGTGTGCACGCGGTTCGTGCTATGCAA
CCCCTGCTAAAGTCTTATTCAAATGTATCTTGCATTGACGCTGGGACACTGAGCTATGAACTTTTGGAGTGGGTTGCGAGTTCCGATCATACGATAGTCATCGACGCGGCTTATATGCGCTGTCCGCCTGGCACGGTGAGAGTGTTTGAGAACGAGGCAATTGCGGATCA
GTTTCAGCAAGCGACGACCCATTCCGTTCACCAGATCACGTTACGCGACACATTGTTGACCAGCCAAACGTTATATTCAAAACCTTCTGCGGTGACGTTAATTGGCGTAGAGCCGGAGGCGATTGAGTGGGGAACCTCTTTGAGTCCAAAGGTAAATGCAGCGCTATCAT
CGATCATGGATATCGCATTTGCTTGCTTATCAGGCGACAACGTTCAGACCTATAAACCGGCAACCCAAAAGGAGGCACGATGACCGAAGAACTCATTTTTATGACATCAAATGCTGATAAAACGGGCAACGTAATGCCGCTTTTGCATCAGATACGTCATGCGTTGTCTC
AGTTAATTGAACGACAAGAACAAACCACGATTGATCTGCGACGATTGCCTTTAAGCGCTAGCGAAGAAGCGCAGTTAGAAGCTTTTCTTGGGCATGGAGAGGTTAAAGCTGATATTCAGGCGCTGGGTGATACGGTGCTAATCGAATCACGCTACGCAGGCGTTTGGCTT
GAAATTCACTATAACGAAGATGTGGAGATCATGGGCAAATACGTTCATATCTGTACCTGTCCACCAATCATAAAATCTCAGCCAGAAGATATGGTGTTATCGCTCAGCAATATAGTGTCTGACATCCATTCGTTGTCTCATCAATCTTCTGATGAAACGGCTAAGGAGGA
TTGACATGCGAGAGTAGGGAACTGCCAGGCATCAAATAAAACGAAAGGCTCAGTCGAAAGACTGGGCCTTTCGTTTTATCTGTTGTTTGTCGGTGAACGCTCTCCTGAGTAGGACAAATCCGCCGGGAGCGGATTTGAACGTTGCGAAGCAACGGCCCGGAGGGTGGCGG
GCAGGACGCCCGCCATAAACTGCCAGGCATCAAATTAAGCAGAAGGCCATCCTGACGGATGGCCTTTTTGCGTTTCTACTACTAGTTGACAATTAATCATCCGGCTCGTATAATGTGTGGAATTGTGAGCGGATAACAATTTCACACATACTAGAAAGAGGAGAAATACT
AGATGGCGCTACCAACATTAAACAAGCAGTTACAAGCGTCCGGTATCTCAAGACGCACATTTCTTAAGTTTTGCGCTACAACGGCGTCTTTACTGGCATTGCCGCAAAGCGCTGTTGCCGATTTGGCGACTGCCCTTGGCAATGCGAGAAGACCCTCTGTGATTTGGTTG
CCCTTTCAAGAGTGCACAGGGTGTACCGAAGCAATATTGCGCTCTCATGCTCCCACATTGGAAAGCCTTATTTTCGATCATATTTCGTTGGATTATCAGCATACGATAATGGCTGCTGCGGGAGAGCAAGCTGAAGACGCTAGGCGTGCGGCGATGAACGCGCACAAAGG
GCAATATTTGTTGTTGGTTGATGGTTCGGTTCCGGTGGGTAACCCAGGATACTCAACGATCAGTGGCATGAGTAATGTCGATATGCTGAGAGAATCGGCAAAAGATGCTGCGGGTATTATTGCCATCGGTACCTGCGCGTCTTTTGGCGGGATCCCTAAAGCAAACCCAA
ATCCGACGGGGGCAGTGGCAGTAAGCGACATTATTACAGACAAGCCAATCGTAAACATTTCAGGCTGTCCGCCACTGCCTATCGCGATTACAGCTGTGTTGGTTCATTACCTGACGTTTAAGCGTTTCCCTGATCTCGACGAATTACAACGCCCACTCGCTTTTTTTGGT
GAAAGCATCCATGACAGATGTTATCGACGCCCATTTTTTGAACAACGTAAATTTGCAAAATCGTTTGATGATGAGGGGGCAAAAAATGGGTGGTGTTTGTTCGAACTTGGTTGTAAAGGGCCCGAAACCTTTAACGCATGTGCAACGGTTAAATGGAATCAAGGCACGAG
TTTTCCTATCGAATCTGGCCATCCGTGTCTTGGTTGCTCTGAGCCCGATTTCTGGGATAAAAGCAGCTTTTACCAAGCCTTGGGTCCATGGGAGTGGTACAAATCCAAACCCGGCAAAGGTGCACAGAAGCATGCTGGGAAAAACTCACGTAGCGCTTGGTCCCACCCGC
AGTTCGAAAAATAAGATAATAGGCAAGGCTCATGGAAAATACAGCAAGTAACAACCGGTTAGTCGTCGATCCTATCACTCGAATTGAAGGGCATCTTCGAATAGAAGCTGAAATGGATGGGAATACCATCAAACAGGCGTTCTCATCAGGCACGTCTGTTCGGGGAATTG
AACTGATTTTACAAGGCAGAGATCCGCGTGACGCTTGGGCTTTTGCGCAACGTATCTGTGGCGTCTGCACGCTGGTGCATGGTATGGCATCGGTGCGTGCTGTCGAAGATGCAATTAGAAAAGCTTGGCGGTCAAACGCAAAATTAGGGGTTGCCATTGGAAAGCCCTCC
ATGACATCTATGCCAAAAGGACCGATGCAACATGGTAAAAAAGGGCACCGACAGTCACGTACTTCAATAGGCGTACTGAGTGAAGCAGAAATGGCTATCCCTCAAAATGCACAACTGATAAGAAACATCATGATTGCAACCCAATATGTGCATGATCACGTGATGCATTT
TTATCACTTGCATGCCCTAGATTGGGTTGACGTTGTTTCTGCACTAGATGCAGATCCAACGAGAACCGCTACGCTCGCCGGTCAATTGAGTGATTATCCTCGTTCATCGCCGGGATATTTCAAAGATGTGAAGCAAAAAGTCAAAACGCTGGTTGAGTCTGGGCAGCTAG
GGATATTCAGTAACGCGTATTGGGGGCATCCTGGCTATAAACTGCCACCTGAAGTTAACTTGATGGCATTAGCACATTATTTAGATGCGCTAACGTGGCAGCGTGAAGTTGTAAAAGTTCACACCATATTTGGAGGGAAAAACCCTCATCCTAATTTTGTCGTTGGCGGC
GTGCCTTCACCGATTAATCTCAATGCGTCAACGGGTATTAACACGAGTCGATTAGTGCAACTACAAGATGCTATCACGCAAATGAAGAGCTTTGTCGATCAGGTGTATTACCCCGATATTGTGGCGATTGCGGGTTATTACAAAGAGTGGGGGACACGAGGGGAAGGGCT
GGGTAACTTTCTTACCTATGGAGACTTACCTATGACATCAATGGATGACCCTGATTCTTTCTTGTTTCCACGAGGTGCAATACTTGGTCGAGACTTGAGTAAAGTGCATGACCTTGATCTAGATGATCCCTCTGAAATTCAAGAATTCGTCTCTTCCTCCTGGTATCGAT
ATAGTGGAGGGAACGCAAGTGGTTTACACCCTTTTAATGGACAAACAACACTCGAGTATACTGGTCCGAAACCGCCTTACAAGCACCTAAATGTAGGGGCTGAATATTCATGGTTGAAAAGTCCGCGTTGGAAAGGCCATGCGATGGAGGTGGGACCGCTGGCTCGCGTG
CTAATGATGTATGCTAAAAAAGATGCCGCTGCGCAAGACATCGTTAATCGATCTCTTTCTATCTTGGATTTAGAGACCTCTGCACTTTTCTCCACACTCGGTAGGACGCTCGCCAGAGCAGTGGAAACAAAAATTGTGGTTAACCAGCTACAGTCTTGGTATGACCAACT

ATTGGATAATATCGCAAAGGGCGATACCGATACGTTTAACCCTCTGTATTTTGACCCTACCAATTGGCCAATTAAAGGCCAGGGGGTAGGCGTGATGGAAGCGCCTCGTGGCGCGTTGGGGCATTGGTTGGTCATGCAAAATGGCAAAATTGAGAATTACCAATGTGTCG
TGCCTACGACATGGAACGCTGGACCTCGAGATCCCAACTCACAGGCAGGTGCTTATGAAGCCGCTCTGCAAGATAAACATACGCTACATGATCCTGACCAACCTTTAGAGATTTTGCGAACACTTCATAGTTTTGACCCCTGCTTAGCATGTGCCGTGCACGTAATGGAC
GAAACAGGGGAAGAGCGTTTGCGTCTAAAAGTTCGTTAAgatcctctacgccggacgcatcgtggccggTCGAGCTCAAGGAGGAATAACACATGCGAGAGTAGGGAACTGCCAGGCATCAAATAAAACGAAAGGCTCAGTCGAAAGACTGGGCCTTTCGTTTTATCTGT
TGTTTGTCGGTGAACGCTCTCCTGAGTAGGACAAATCCGCCGGGAGCGGATTTGAACGTTGCGAAGCAACGGCCCGGAGGGTGGCGGGCAGGACGCCCGCCATAAACTGCCAGGCATCAAATTAAGCAGAAGGCCATCCTGACGGATGGCCTTTTTGCGTTTCTACTACT
AGTTGACAATTAATCATCCGGCTCGTATAATGTGTGGAATTGTGAGCGGATAACAATTTCACACATACTAGAAAGAGGAGAAATACTAGATGTGTCTCGCTATCCCCATGAAAGTCATTGCAATTAAAGGCTTTAACGCAACGTGTGAAGCGAAAGGTGTCTCTCGCGAG
GTGAGTTTACATTTAGTGCAAGGGCTAGAGGTCAAAGTTGGCGACTATGTGATGGTACATGTTGGTTATGCTCTCCAAGTGATCACCTATGATGAAGCGCAGGTGACATGGGAGATGCTCGATCAGGTCATTGCTTACGATGCATGAAATTAGTCTTTGCTATAGCTTGC
TTGACACAGTCGCTGTTCACCAGCGAGCAAACATGAATAAGTCTGTCAGTCTGGTTCACGTCAAAGTCGGCCCACTGTCAGGTGTTGAGCCTGACTTGCTGCACCATGCGTTCTTGGCGTGTAGAACTCACACCATTGGCGATCAGGCAACGCTTAGAATTGACACAAGC
GCTATCAAGATACGCTGTAAATTGTGTGGCGAATTCAGCCGAGTTTCGGTGAATAGCATCCTATGTGCAAGTTGCGGTGCTTGGCAAACCGACCTCATTGAGGGCGATGAATTTATATTGCAACGAATAGAGTTTATCGCACCTGACAATTTTACAGAATCAAAGGAGAG
AAAACATGTGCGGTAACTGTGGTTGCGAAACGCAAGTAAAATCAGAGAAAACTGACAAGCCATCAGCAAATATCGTTGAGGTTCACGCAAATTTAAAAAGCAACAATGACAACCAAGCGATAGCAAACCGTGCTTTATTTGACGCACATAATGTACTAGTGATTAATTTG
ATGTCATCCCCAGGCAGTGGAAAAACCAGGCTACTCGAAGAAACGATACGTGCGCTTAAGTCGCAATACGCGATGGCGGTGATTGAAGGCGACCTCGAAACTGAAAATGACGCAAATAGAATTAGACGTCATGGTGTGCAGGCGGAGCAAATCGCCACGGGCCAAGGGTG
TCACTTGGACGCCAGTATGGTGCAAAAGGTACTTGGAAGATTTAGCTTACAAAAACTAGACGTTTTGTTTATAGAGAATGTTGGCAATTTGATTTGTCCAGCGTGTTTTGATTTAGGACAGCACTTAAACATTATTTTGCTGTCCGTACCAGAAGGTGATGATAAGCCTG
AAAAGTACCCAGTGATGTTTCGTGCCGCGGACGTCATGCTTATTTCTAAGACCGATTACTTGCAATTTCACGATGAATTCAATGTACCTCGCGCGATTGAATCATTTCGTAAAGTGGGCAACGATGCGCCCGTTCTCGAAGTGTCTTCATTGAAAAGTCAACATCTAGAG
CGTTGGTTTTCTTATTTTACGCACGCCATTACAGCTCATCAGTCTCAGTCGTTACAAAATAGCGGAGTCTAACATGCGAGAGTAGGGAACTGCCAGGCATCAAATAAAACGAAAGGCTCAGTCGAAAGACTGGGCCTTTCGTTTTATCTGTTGTTTGTCGGTGAACGCTC
TCCTGAGTAGGACAAATCCGCCGGGAGCGGATTTGAACGTTGCGAAGCAACGGCCCGGAGGGTGGCGGGCAGGACGCCCGCCATAAACTGCCAGGCATCAAATTAAGCAGAAGGCCATCCTGACGGATGGCCTTTTTGCGTTTCTACTACTAGTTGACAATTAATCATCC
GGCTCGTATAATGTGTGGAATTGTGAGCGGATAACAATTTCACACATACTAGAAAGAGGAGAAATACTAGATGTACAGTGTTGAATCGTTGCTAAGAGACATTAAGAATACCTCGCTTTCAAAACCGTTTCGTATTCTTAATGTTTGCGGTGGCCATGAGCGTGCGATTA
CGCGCGCTGGATTTAGAACGTTGTTTCAGCACAATATTCATCTGATACCTGGGCCAGGGTGCCCCGTTTGTATTTGCCCCGAAGAGGATATTGCATACGCGATACACTTAGCCATTAACGAAAATGTCGTTATCGTTAGTTTTGGTGACATGCTCCGCGTCCCAGTCGAA
CACGAAATGGGTGGCTGTAGCTCATTGATAGACGCAAAAAATCAAGGGGCCGATATCCGTCCGATATCTTCTCCTCAAGAAGCGGTATCTATAGCATTGGAAGAACCTCGTAAGGTTATTGTGTTTTTTGCTGTTGGTTTTGAAACGACTATGGCACCGATTGCAGCAAC
ACTGCTTAATGATTTACCCAATAACTTTAAAGTATTATTGTCTGGACGACTTACTTGGCCAGCAGTTGCCCATGTGCTTGAGAGTCAACCGAATACTTTTGATGCACTGATTGCGCCAGGCCACGTTGCGACAATTATGGGTAGTGAAGAATGGCAGTTTGCAATCGACC
ATCATAATTTGCCTGTCAGTATTGCTGGTTTTCATCCAGAAAGTTTGCTGTTGTCGCTCCAAACGCTGCTCGGTAATTGTTCAAATAAAGTCGTCACACTGAGTAACCGCTATCCCGAAGTGGTAAAGCAAAACGGCAATGCAGCAGCTAAAGCAATAATCAATAAAGCG
TTCACTATCGTTGACGCACATTGGCGAGGAATTGGTGTTATTCCTGGTTCTGGATTTTCCTTTGCTTCGAGACTATCGCACCTAGACGCCACAAACGATTATGCGCCAGTCGATTTCCCTTCGCAATGTGCGCAAAACGTTCCTGAAACCACATCGCCCTGTGAAAAAGT
GATCCTCGGAAAAATGGCACCTGATGCGTGCCCGTTTTTGGTCAGGAATGCAAACCAGCATCCCCTAAAGGCGCATGTATGGTGTCTGATGAGGGGGCTTGCCGAATTTGGTATAGCTCTGGTGAGCGCTCGATAACAAATGTTATCAAAAAGGGGAATACGCTAAAGG
TGGAGATGAAGTGATGACAACTGCCCGCACATATGTTGCCAATACGAATAAGAGTAAAACTATCGCCTATGAGTTGAACATTACTGGAGTCATCCAGGGGGTTGGGTTCAGACCGTTAGTTTACTGCTTAGCGTATGAAAAAAGTATTGTTGGTTGGGTACAAAACGACT
GTGGATGTGTGCGAATACACGCTGAAGGCACTGAGCTAGACGTTGAACAATTTGTATTCGACTTACTGAACAATGGGTCGCTAGTTTCCATATCCTTGCTGGAAAAAAAGCAGGTTAAACTGAGTAATGTAGACTCGTTCACGATAAAAGAAAGCGGTCATGATATAGCG
TGCGGGGCGATATCAATCCCCAAAGATGTCTATTTGTGTGGAGCGTGTAAAGCTGAGTTACTGTCAAATACTAACCGACGTGGCACCTATAGCTTTATCGCTTGCAGTAAATGTGGTCCACGATTTTCTATGCTGCGAGCAATGCCATACGACAGAAAGAACATGAGTAT
GTCAGCATTTCCAATGTGTGAGTTATGTCGTAAAGAATATCAATCGCCAACTGATAGGCGCTTTCATGCTCAACCAATAAGCTGTCGCGTATGCGGGCCTGAAGTATTTTGTTCAACTGTCGGCGGGCGCGTGGTCTCGCAAGGCGATGTTGATGTTGTAGCAACGGTTG
TCGCGTATTTGAATCAAGGCAAAATTGTCGCGTTAAAAAGTATAGGCGGTTATCACTTGCTCTGTGACGCACAAAGCACTGAAGCGGTAGACCTTTTACGCCAGCGTAAAAATAGACCAGATAAACCTTTCGCGGTCATGCTCCCAGAGCCTCGTGCAGACGAAACAAGT
GAGGATTGGTTAGATAAATGCGTTGCTGTAGACAGTCATCAAAGAGCGCTCTTACTAAGCCCCGTGCGACCCATTTTACTTGCAAAAAAGCAATCGAATGTACCCATTGCAGATAACGTAGCGCCCATGCTTCGTGATTTGGGGGTGATGTTGCCGTGCAGCGGACTGCA
TTTACTCATTATGCAGCAATTCAATCGCCCCATGGTGGCTACGTCAGGAAATATTTACGGTGAACCGATGGTCACATCGACGTCATCGGCACAGCAACACCTTTCGATGTTGGCAGACATCTTCGTGCACCATAATCGAGACATTTTTCACAAACTGGATGATTCTGTGC
TGCGAACGCTTAAGTCAGGCACCATTCCAATTCGCCTGGGGCGGGGTATAAGTCCGATAGAATTATCATTGCCATATACTGTGCAGGAACCAATGCTTGCTGTTGGCGCGAATCAGAAAAATACAGTCGCAATAGCTTTGAACAATCGACTTATCGTAACACCGCATATT
GGCGACCTCGATTCTCCGATAATGCAAGCGCACTTTGAAAAATGTATCAGTGACTTTCAAGCTCTCTATCATGTTCATCCACAGCGTATTCTTTGCGATAGCCATCCTGGCTATATCAGTTCCCGTTGGGCCAAAGGCAGTGAACTACCGACTACTAAAGTTTTACATCA
CCACGCTCATGCATCAGCGTGGGCATTAGCTGCTGAAATATCGACACCAAGCTTGGTTTTCGTGTGGGATGGAACGGGATTAGGCGAGAATGGCGAGATGTGGGGAGGGGAAGTTTTTTGTGGGACGCCGGGCAATTGGAAACGTGTTGCCTCATTAGCGCCTATGAAGT
TGCAAGGCGGCAACAAGGTTCCAAATCAACCATGGCGAAGTGCAGCATCACTAGTGTGGGGTACTAATTTCACCGGCATTGATTTTAAACCATTTGATCCACAACAATTAAGCTACAACGCATGGCAAAACGAACTCAATTGTCATGTTAGTAGTTCAATGGGAAGACTG
TTCGACGCGGCGGCATTTGCCTGCTGTGAACAGAGCGAAGTCTCTTATGAAGGGCAGGCGCCGATGATGTTGGAAAGCGTTGCGTCGACGCCCTCTAAGGTTATGCCGTTAACGTTGGTCCAGAAAAACGACGAATTTATTCAAATGGATTGGCGAACACTGGTACCGGA
ACTATTTGATTTCAAAGTGTCTCCTCAAACTCGTGCTGCAAATTTCCACGCTTCGTTAATTGAAACGGTATGGGAGGTAACCCTACATTGCCGTAAAAAGTTTAATTTTGACCACATTGGATTGGCAGGAGGCGTCTTCCAAAATCGCATTTTGTGTGACGGTATATCAC
AACGGTTTTTACAATCAGACATTCCTGTGGTTATTCCAAGTAGTTTGCCATTAAACGATGCCGCAATAAGTGTTGGGCAAATCCACGAATACTGTTCACGGAGGTCATAATGAAAAGCGCAGAGGGCAATAATCATTCATCATCAGAGCCGTTGGGCACGGAATCTATTC
AATTAGCTCAGGGGAACGGCGGAACACTGACAAAAAGACTCATTGATCATGTTTTTAAAAATAAAGCGAACAAAGATCTAGATCTGCTTCATGATGCCGCCACAGTAACTTTTGATGGTCCTTATCTGAGTATAACAACGGATAGCTTCGTTGTATCCCCCCCAACATTT
CCAGGCGGCAATGTAGGCGCGTTGAGTGTTTACGGTACCGTCAATGATTTAGCAGTCGTTGGGGCAACACCACGGTACATCAGTACTGCATTTATTATTGAAGAAGGCTTTTCCCTTTCAACATTGAAACAAATCGTTAATGGAATGCATCAAGCTGCCGAAGAAACGAA
GGTCGCCATTGTAACAGGTGATACGAAAGTTGTCCCGAAAGGCAGTGGTGGCGGCGTTTTTATCAATACAACGGGTGTTGGAGACAGCCGAGATGCGCCGATTTGGGATACCAGTCTTATAAAGGCAGGCGATCATGTACTGGTTAGTGGTTCTGTAGGTGATCACGGTG
CGTGTGTCCTGCTTGCTCGCGAGGACTATGGTTTGCAAGGTCAGCTAAAGTCTGATTGTGGAAGCGTTGTTCCTCTTATTGATCCAATCAAGCATTTGGAAGGTGTTCGTTTTGTGCGTGACCCAACTCGTGGAGGGTTGTCAGTATTACTCCATGACGTTGCTAATGAA
ACAGGCTTCGATATCGAGTTAATTGAAAATAATATTCCGGTGCGACCTGAAGTGGCAAGCGTGTGTGAAATATTAGGCTTTGATCCGTTTGTTTTAGCGTGTGAGGGACGCATTGTTGCTGTTGTTGCCCCTGACATAAGCGGTAAGGTGCTAGAACACTGGCGGAGCAT
TCGAAACGGGGAGCAGGCCGAACACATTGGTGTAATAGTAAAAAACACCGAATCAAAAGGGCGCGTGACCATTGTTACGCCAATGGGAGGAAGACGTTTCATGAATGAACTCGAAGATGAGCCGCTTCCAAGGATCTGCTGAGATCCTCTAGAGTCGACCTGCAGGCATG
CAAGCTTGGCTGTTTTGGCGGATGAGAGAAGATTTTCAGCCTGATACAGATTAAATCAGAACGCAGAAGCGGTCTGATAAAACAGAATTTGCCTGGCGGCAGTAGCGCGGTGGTCCCACCTGACCCCATGCCGAACTCAGAAGTGAAACGCCGTAGCGCCGATGGTAGTG
TGGGGTCTCCCCATGCGAGAGTAGGGAACTGCCAGGCATCAAATAAAACGAAAGGCTCAGTCGAAAGACTGGGCCTTTCGTTTTATCTGTTGTTTGTCGGTGAACGCTCTCCTGAGTAGGACAAATCCGCCGGGAGCGGATTTGAACGTTGCGAAGCAACGGCCCGGAGG
GTGGCGGGCAGGACGCCCGCCATAAACTGCCAGGCATCAAATTAAGCAGAAGGCCATCCTGACGGATGGCCTTTTTGCGTTTCTACAAACTCTTTTGTTTATTTTTCTAAATACATTCAAATATGTATCCGCTCATGGCGGCCCAGATCCCCGGGTACCGAGCTCGAATT
TCGAGCTTCTGGAGCAGGAAGATGTCGCGGGCATTAGCACCAGCGGTCTGCCAAGCCTCCGCCAGCCGTTGGGTCCCTTCCGCTTGAGCTTTTCCATCTTCGACGATACGGGCGGCGGCCCCCCGCGCTTCCGCGATCGCCCGTTTACAAGCTGCCTCAGCTGGGGCGAT
CACATCGGCTTGAAGTTGCTGCTGCACCTGTTTGATCCGCTCCTGCTGCACAGGGAGTTCTGCTTGGCTACGAGCGACTTCGGTAGCAATGTCCGCTTCAGCTTCGGCCACCACCGCTTCGCGCCGCGTCAACGCATCCTGAATCCGGCGCTCGGCCTCGGCTTGGGCGA
TCGCTACATCGCGATCGATCCGACGCAGGGCCGTGATCTTGTCATTTTCGGCCGTTTGGATCGCAGAGGCAGCCTGGGCATCGGCTTCAGCAATTCGGGCATCTCGCTGCAGATCAGCCCGCTGCTTGCGTCCACTAGCCGAGAGATAACCGACCTCATCGGAAATGTTC
TGGACTTGCAGCGTATCGAGGACTAGACCCAGCTGCTCAAGGTCATCCTCCGCCTCTTCCAGCAGACTTTTGGCAAAGGCAATTTTGTCCTCGTTGATCTGCTCCGGCGTGAGGCTGGCTAAAACACCACGCAAGTTGCCTTCGAGGGTCTCCTTGGCAATTTGCTCGAT
TTCCTTACGGTTTTTGCCAAGCAGCCGCTCGATCGCGTTGTGGATGGTCGGTTCTTCCCCAGCAATCTTGATATTGGCAACGCCTTCAACAGTCAGGGGAATGCCGCCCTTGGAGAAGGCATTGGAAACGCGCAACTCAATGATCATGTTGGTCAGATCCATGCGGAGCG
CTTTTTCCAGCAGAGGTACCCGCAGGCTGCTGCCGCCCTTGACCAAGCGATAGCCAACTCGGCGGCCATCACTACTGCGGCGACTACTGCCAGCAAAGATCAAAATTTCACTGGGTTGGCAGATGTAGTAGAGATTGCGCAGGACTAAGCTGCCAGCCCCGGCGACTCAC
CAGTCACAGAAAAGCATCTTACGGATGGCATGACAGTAAGAGAATTATGCAGTGCTGCCATAACCATGAGTGATAACACTGCGGCCAACTTACTTCTGACAACGATCGGAGGACCGAAGGAGCTAACCGCTTTTTTGCACAACATGGGGGATCATGTAACTCGCCTTGAT
CGTTGGGAACCGGAGCTGAATGAAGCCATACCAAACGACGAGCGTGACACCACGATGCCTGCAGCAATGGCAACAACGTTGCGCAAACTATTAACTGGCGAACTACTTACTCTAGCTTCCCGGCAACAATTAATAGACTGGATGGAGGCGGATAAAGTTGCAGGACCACT
TCTGCGCTCGGCCCTTCCGGCTGGCTGGTTTATTGCTGATAAATCTGGAGCCGGTGAGCGTGGGTCTCGCGGTATCATTGCAGCACTGGGGCCAGATGGTAAGCCCTCCCGTATCGTAGTTATCTACACGACGGGGAGTCAGGCAACTATGGATGAACGAAATAGACAGA
TCGCTGAGATAGGTGCCTCACTGATTAAGCATTGGTAACTGTCAGACCAAGTTTACTCATATATACTTTAGATTGATTTAAAACTTCATTTTTAATTTAAAAGGATCTAGGTGAAGATCCTTTTTGATAATCTCATGACCAAAATCCCTTAACGTGAGTTTTCGTTCCAC
TGAGCGTCAGACCCCGTAGAAAAGATCAAAGGATCTTC

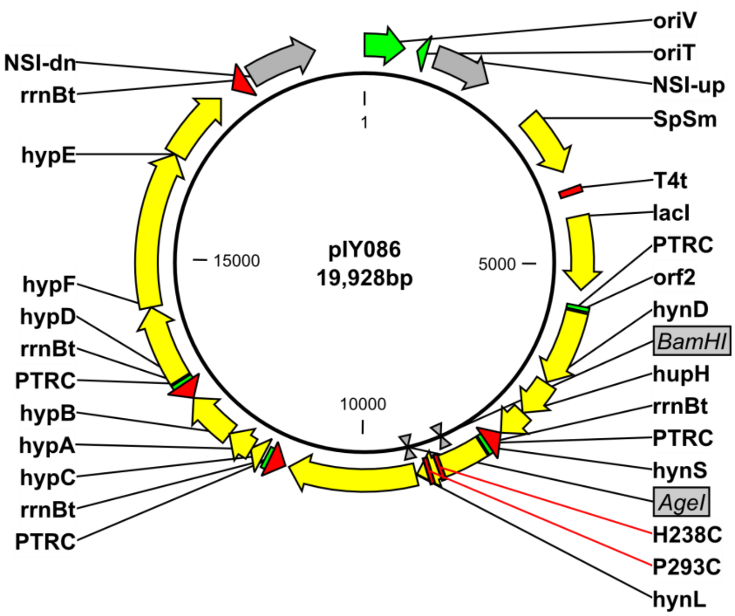

TTGAGATCCTTTTTTTCTGCGCGTAATCTGCTGCTTGCAAACAAAAAAACCACCGCTACCAGCGGTGGTTTGTTTGCCGGATCAAGAGCTACCAACTCTTTTTCCGAAGGTAACTGGCTTCAGCAGAGCGCAGATACCAAATACTGTCCTTCTAGTGTAGCCGTAGTTAG
GCCACCACTTCAAGAACTCTGTAGCACCGCCTACATACCTCGCTCTGCTAATCCTGTTACCAGTGGCTGCTGCCAGTGGCGATAAGTCGTGTCTTACCGGGTTGGACTCAAGACGATAGTTACCGGATAAGGCGCAGCGGTCGGGCTGAACGGGGGGTTCGTGCACACAG
CCCAGCTTGGAGCGAACGACCTACACCGAACTGAGATACCTACAGCGTGAGCTATGAGAAAGCGCCACGCTTCCCGAAGGGAGAAAGGCGGACAGGTATCCGGTAAGCGGCAGGGTCGGAACAGGAGAGCGCACGAGGGAGCTTCCAGGGGGAAACGCCTGGTATCTTTA
TAGTCCTGTCGGGTTTCGCCACCTCTGACTTGAGCGTCGATTTTTGTGATGCTCGTCAGGGGGGCGGAGCCTATGGAAAAACGCCAGCAACGCGGCCTTTTTACGGTTCCTGGCCTTTTGCTGGCCTTTTGCTCACATGTTCTTTCCTGCGTTATCCCCTGATTCTGTGG
ATAACCGTATTACCGCCTTTGAGTGAGCTGATACCGCTCGCCGCAGCCGAACGACCGAGCGCAGCGAGTCAGTGAGCGAGGAAGCGGAAGAGCGCCTGATGCGGTATTTTCTCCTTACGCATCTGTGCGGTATTTCACACCGCATATGGTGCACTCTCAGTACAATCTGC
TCTGATGCCGCATAGTTAAGCCAGTATACACTCCGCTATCGCTACGTGACTGGGTCATGGCTGCGCCCCGACACCCGCCAACACCCGCTGACGCGCCCTGACGGGCTTGTCTGCTCCCGGCATCCGCTTACAGACAAGCTGTGACCGTCTCCGGGAGCTGCATGTGTCAG
AGGTTTTCACCGTCATCACCGAAACGCGCGAGGCAGGATATCCGGCAGCCGGCGGAGCGCTGCTTTCTTGGCAAGCGGTCGCCAGCCCCAACGCCAGGGCTGCCAGCCCGAAACAGCGGGGCAAGGCAGCTTGGAAGGGCGATCGCAGCACGGGCATGGCAATGTCTCTC
TGAAGGAATGCAGACCTTATTCGTACAGCCAGGGTTGAATCGTGGGGGTCCAATCACTTAGCTCTGCTGGGCTAAACCAGAGAGCAATTTCCTGTTGTGCTGTTTCGATTGCATCCGAGCCATGGATGATGTTGCGGCCAATATTGACACCAAAATCACCACGGATGGTG
CCCGGTTCTGCCGTCAGCGGATTGGTAGCGCCGATCAACTTGCGAGCAGCCGCCACAACGCCTTCGCCTTCCAAGACGATCGCCACGATCGGCCCAGAGGTGATGAACTCGACGAGGCCATTGAAGAAGGGGCGCTCGCGGTGGACAGCATAGTGCTGTTCGGCCAGCTC
GCGACTGGGCTTCAGCTGCTTTAGGCCCACCAGTTTGAAGCCTTTTTGCTCAAAGCGGCCGATGATCGTACCGACCAAACCCCGCTGAACGCCATCGGGCTTGATGGCAATAAATGTGCGTTCCACAGACATCTAGATAGTCCTCAAGACGAGGCAAGCATTGAGCTTGC
CTTCCTATGGTTCGGGATCACTGGGATTCTTGACAAGCGATCGCGGTCACATCGCTATCTCTTAGGACTTCGCAGCGGGCGAGTCGGATTGACCCGGTAGGGATTTCGCCAGATCAATGCCCGTGGTTTGTTTCAGCTTCTCCAGCAAGCTAGCGATTTGGGTAGCGCTG
CCTTCCCCTTCGCCAATCACAGTGATCGACTCCACGTCGATATCTGGCACGGTGCCTGAAAGCGTGACGAGCAGGGACTCGAAGCTTGCATGCCTGCAGGTCGACTCTAGAGCTTTATGCTTGTAAACCGTTTTGTGAAAAAATTTTTAAAATAAAAAAGGGGACCTCTA
GGGTCCCCAATTAATTAGTAATATAATCTATTAAAGGTCATTCAAAAGGTCATCCACCGGATCAATTCCCCTGCTCGCGCAGGCTGGGTGCCAAGCTCTCGGGTAACATCAAGGCCCGATCCTTGGAGCCCTTGCCCTCCCGCACGATGATCGTGCCGTGATCGAAATCC
AGATCCTTGACCCGCAGTTGCAAACCCTCACTGATCCGCATGCCCGTTCCATACAGAAGCTGGGCGAACAAACGATGCTCGCCTTCCAGAAAACCGAGGATGCGAACCACTTCATCCGGGGTCAGCACCACCGGCAAGCGCCGCGACGGCCGAGGTCTTCCGATCTCCTG
AAGCCAGGGCAGATCCGTGCACAGCACCTTGCCGTAGAAGAACAGCAAGGCCGCCAATGCCTGACGATGCGTGGAGACCGAAACCTTGCGCTCGTTCGCCAGCCAGGACAGAAATGCCTCGACTTCGCTGCTGCCCAAGGTTGCCGGGTGACGCACACCGTGGAAACGGA
TGAAGGCACGAACCCAGTGGACATAAGCCTGTTCGGTTCGTAAGCTGTAATGCAAGTAGCGTATGCGCTCACGCAACTGGTCCAGAACCTTGACCGAACGCAGCGGTGGTAACGGCGCAGTGGCGGTTTTCATGGCTTGTTATGACTGTTTTTTTGGGGTACAGTCTATG
CCTCGGGCATCCAAGCAGCAAGCGCGTTACGCCGTGGGTCGATGTTTGATGTTATGGAGCAGCAACGATGTTACGCAGCAGGGCAGTCGCCCTAAAACAAAGTTAAACATCATGAGGGAAGCGGTGATCGCCGAAGTATCGACTCAACTATCAGAGGTAGTTGGCGTCAT
CGAGCGCCATCTCGAACCGACGTTGCTGGCCGTACATTTGTACGGCTCCGCAGTGGATGGCGGCCTGAAGCCACACAGTGATATTGATTTGCTGGTTACGGTGACCGTAAGGCTTGATGAAACAACGCGGCGAGCTTTGATCAACGACTTTTGGAAACTTCGGCTTCCCC
TGGAGAGAGCGAGATTCTCCGCGCTGTAGAAGTCACCATTGTTGTGCACGACGACATCATTCCGTGGCGTTATCCAGCTAAGCGCGAACTGCAATTTGGAGAATGGCAGCGCAATGACATTCTTGCAGGTATCTTCGAGCCAGCCACGATCGACATTGATCTGGCTATCT
TGCTGACAAAAGCAAGAGAACATAGCGTTGCCTTGGTAGGTCCAGCGGCGGAGGAACTCTTTGATCCGGTTCCTGAACAGGATCTATTTGAGGCGCTAAATGAAACCTTAACGCTATGGAACTCGCCGCCCGACTGGGCTGGCGATGAGCGAAATGTAGTGCTTACGTTG
TCCCGCATTTGGTACAGCGCAGTAACCGGCAAAATCGCGCCGAAGGATGTCGCTGCCGACTGGGCAATGGAGCGCCTGCCGGCCCAGTATCAGCCCGTCATACTTGAAGCTAGACAGGCTTATCTTGGACAAGAAGAAGATCGCTTGGCCTCGCGCGCAGATCAGTTGGA
AGAATTTGTCCACTACGTGAAAGGCGAGATCACCAAGGTAGTCGGCAAATAATGTCTAACAATTCGTTCAAGCCGACGCCGCTTCGCGGCGCGGCTTAACTCAAGCGTTAGATGCACTAAGCACATAATTGCTCACAGCCAAACTATCAGGTCAAGTCTGCTTTTATTAT
TTTTAAGCGTGCATAATAAGCCCTACACAAATTGGGAGATATATCATGAAAGGCTGGCTTTTTCTTGTTATCGCAATAGTTGGCGAAGTAATCGCAACATCCGCATTAAAATCTAGCGAGGGCTTTACTAAGCTGATCCGGTGGATGACCTTTTGAATGACCTTTAATAG
ATTATATTACTAATTAATTGGGGACCCTAGAGGTCCCCTTTTTTATTTTAAAAATTTTTTCACAAAACGGTTTACAAGCATACTAGAGGATCGGCGGCCGCGGATCTGGGCCGCGGGTCATGGCTGCGCCCCGACACCCGCCAACACCCGCTGACGCGCCCTGACGGGCT
TGTCTGCTCCCGGCATCCGCTTACAGACAAGCTGTGACCGTCTCCGGGAGCTGCATGTGTCAGAGGTTTTCACCGTCATCACCGAAACGCGCGAGGCAGCAGATCAATTCGCGCGCGAAGGCGAAGCGGCATGCATTTACGTTGACACCATCGAATGGTGCAAAACCTTT
CGCGGTATGGCATGATAGCGCCCGGAAGAGAGTCAATTCAGGGTGGTGAATGTGAAACCAGTAACGTTATACGATGTCGCAGAGTATGCCGGTGTCTCTTATCAGACCGTTTCCCGCGTGGTGAACCAGGCCAGCCACGTTTCTGCGAAAACGCGGGAAAAAGTGGAAGC
GGCGATGGCGGAGCTGAATTACATTCCCAACCGCGTGGCACAACAACTGGCGGGCAAACAGTCGTTGCTGATTGGCGTTGCCACCTCCAGTCTGGCCCTGCACGCGCCGTCGCAAATTGTCGCGGCGATTAAATCTCGCGCCGATCAACTGGGTGCCAGCGTGGTGGTGT
CGATGGTAGAACGAAGCGGCGTCGAAGCCTGTAAAGCGGCGGTGCACAATCTTCTCGCGCAACGCGTCAGTGGGCTGATCATTAACTATCCGCTGGATGACCAGGATGCCATTGCTGTGGAAGCTGCCTGCACTAATGTTCCGGCGTTATTTCTTGATGTCTCTGACCAG
ACACCCATCAACAGTATTATTTTCTCCCATGAAGACGGTACGCGACTGGGCGTGGAGCATCTGGTCGCATTGGGTCACCAGCAAATCGCGCTGTTAGCGGGCCCATTAAGTTCTGTCTCGGCGCGTCTGCGTCTGGCTGGCTGGCATAAATATCTCACTCGCAATCAAAT
TCAGCCGATAGCGGAACGGGAAGGCGACTGGAGTGCCATGTCCGGTTTTCAACAAACCATGCAAATGCTGAATGAGGGCATCGTTCCCACTGCGATGCTGGTTGCCAACGATCAGATGGCGCTGGGCGCAATGCGCGCCATTACCGAGTCCGGGCTGCGCGTTGGTGCGG
ATATCTCGGTAGTGGGATACGACGATACCGAAGACAGCTCATGTTATATCCCGCCGTTAACCACCATCAAACAGGATTTTCGCCTGCTGGGGCAAACCAGCGTGGACCGCTTGCTGCAACTCTCTCAGGGCCAGGCGGTGAAGGGCAATCAGCTGTTGCCCGTCTCACTG
GTGAAAAGAAAAACCACCCTGGCGCCCAATACGCAAACCGCCTCTCCCCGCGCGTTGGCCGATTCATTAATGCAGCTGGCACGACAGGTTTCCCGACTGGAAAGCGGGCAGTGAGCGCAACGCAATTAATGTAAGTTAGCGCGAATTGATCTGGTTTGACAGCTTATCAT
CGACTGCACGGTGCACCAATGCTTCTGGCGTCAGGCAGCCATCGGAAGCTGTGGTATGGCTGTGCAGGTCGTAAATCACTGCATAATTCGTGTCGCTCAAGGCGCACTCCCGTTCTGGATAATGTTTTTTGCGCCGACATCATAACGGTTCTGGCAAATATTCTGAAATG
AGCTGTTGACAATTAATCATCCGGCTCGTATAATGTGTGGAATTGTGAGCGGATAACAATTTCACACAGGAAACAGACCATGGAggaggaactagATGGCTTCCATTATTGGTAGCGGCTTTTTGATCATTGCACCACTGCTGCATTCGGTAATGGGGAAATGGGCTCTG
CTTGGAATAGTGTTGTTGTCCATTCTGGCATATGCGCTGGGGTCGGTTATTCGCTTCAATATACGCCATGCAGAACCTGAGCTAGCTAGCAATCAACAAAGCAGTATTGTGACGCTTGAAAAAGCAAGTCAATGGGCGCTTGGTGCGGCATATGCCATTTCCGTGGCGTT
TTATATTAGTTTGTTTGCAGCGTTTGTTTTTGATCGTTTATCAATCTCTGATACGACGTACATCAAACTGTTTACCAGTGGGTTGCTTGTCATCATTACGGTGGTCGCTTGGCTCCGTGGTGCTAGGGGGCTAGAGACCATTGAGCTATTCGCCGTGACAATAAAGTTGG
CTATTATAGTAGGTGTGCTCGCAGCGCTGGCAACCTACGACATACAAGTACAAAGTGCTTGGTTTCAACATGAAGCGATACAGGCATTGAGCCATTTTGAAACTGTTAGCATGCTCGCAGGCATGCTCATGGTAACGCAAGGGTTCGAGACAACCCGTTTTATGGGGAAT
AACTACACGCCAGAACAGCGCATTAAAGCGAGTCGTTATGCTCAGTGGATCGCTATTTTTCTCTATGTTGTTTTTATCGGTCTAACGTGTCCTATTTTTCTGGATTTTCCGATCACGGAATTAAACGAAACAACGATTAGCTACACCCTAGGGCAAGCAATATGGGTATT
GCCGATTTTATTACTGGTTGCCGCCACGGCAAGTCAATTAAGTGCAGCATTAGCCGATACTATCGGTGGCGGAGGCCTCTTAAAAGAGCTTTTCCATTTACCTATCTCCCCGCAATTTTACTACGTTTTAGTCATCGCCATCGCAGGTATATTAGTTTGGTCATCCAACG
TATTTGAAATTATTAATCTCGCTTCAAAAGGGTTTGCACTTTATTACCTGATCCAAACAATAATCGCGGTCAAACTCGTGATGCGTCAAAGCACAGGTCAACGCATTAGGTCAACAAAATTACTTGGTCTATCGGTGATCGTTCTCTGTTTATTCTTTGTGATTGGCTGG
TCAATTCCAGCACCTCATAGCTAATAAGGAAAATACCGATGCTATCAGTAGAAACTACACCACCAATTGACCAAATCAAAAACGCGGCTAAGCGCTACGCTATCATTGGTGTTGGAAATCTATTGCAGCGAGATGATGGCGTTGGTGTGCACGCGGTTCGTGCTATGCAA
CCCCTGCTAAAGTCTTATTCAAATGTATCTTGCATTGACGCTGGGACACTGAGCTATGAACTTTTGGAGTGGGTTGCGAGTTCCGATCATACGATAGTCATCGACGCGGCTTATATGCGCTGTCCGCCTGGCACGGTGAGAGTGTTTGAGAACGAGGCAATTGCGGATCA
GTTTCAGCAAGCGACGACCCATTCCGTTCACCAGATCACGTTACGCGACACATTGTTGACCAGCCAAACGTTATATTCAAAACCTTCTGCGGTGACGTTAATTGGCGTAGAGCCGGAGGCGATTGAGTGGGGAACCTCTTTGAGTCCAAAGGTAAATGCAGCGCTATCAT
CGATCATGGATATCGCATTTGCTTGCTTATCAGGCGACAACGTTCAGACCTATAAACCGGCAACCCAAAAGGAGGCACGATGACCGAAGAACTCATTTTTATGACATCAAATGCTGATAAAACGGGCAACGTAATGCCGCTTTTGCATCAGATACGTCATGCGTTGTCTC
AGTTAATTGAACGACAAGAACAAACCACGATTGATCTGCGACGATTGCCTTTAAGCGCTAGCGAAGAAGCGCAGTTAGAAGCTTTTCTTGGGCATGGAGAGGTTAAAGCTGATATTCAGGCGCTGGGTGATACGGTGCTAATCGAATCACGCTACGCAGGCGTTTGGCTT
GAAATTCACTATAACGAAGATGTGGAGATCATGGGCAAATACGTTCATATCTGTACCTGTCCACCAATCATAAAATCTCAGCCAGAAGATATGGTGTTATCGCTCAGCAATATAGTGTCTGACATCCATTCGTTGTCTCATCAATCTTCTGATGAAACGGCTAAGGAGGA
TTGACATGCGAGAGTAGGGAACTGCCAGGCATCAAATAAAACGAAAGGCTCAGTCGAAAGACTGGGCCTTTCGTTTTATCTGTTGTTTGTCGGTGAACGCTCTCCTGAGTAGGACAAATCCGCCGGGAGCGGATTTGAACGTTGCGAAGCAACGGCCCGGAGGGTGGCGG
GCAGGACGCCCGCCATAAACTGCCAGGCATCAAATTAAGCAGAAGGCCATCCTGACGGATGGCCTTTTTGCGTTTCTACTACTAGTTGACAATTAATCATCCGGCTCGTATAATGTGTGGAATTGTGAGCGGATAACAATTTCACACATACTAGAAAGAGGAGAAATACT
AGATGGCGCTACCAACATTAAACAAGCAGTTACAAGCGTCCGGTATCTCAAGACGCACATTTCTTAAGTTTTGCGCTACAACGGCGTCTTTACTGGCATTGCCGCAAAGCGCTGTTGCCGATTTGGCGACTGCCCTTGGCAATGCGAGAAGACCCTCTGTGATTTGGTTG
CCCTTTCAAGAGTGCACAGGGTGTACCGAAGCAATATTGCGCTCTCATGCTCCCACATTGGAAAGCCTTATTTTCGATCATATTTCGTTGGATTATCAGCATACGATAATGGCTGCTGCGGGAGAGCAAGCTGAAGACGCTAGGCGTGCGGCGATGAACGCGCACAAAGG
GCAATATTTGTTGTTGGTTGATGGTTCGGTTCCGGTGGGTAACCCAGGATACTCAACGATCAGTGGCATGAGTAATGTCGATATGCTGAGAGAATCGGCAAAAGATGCTGCGGGTATTATTGCCATCGGTACCTGCGCGTCTTTTGGCGGGATCCCTAAAGCAAACCCAA
ATCCGACGGGGGCAGTGGCAGTAAGCGACATTATTACAGACAAGCCAATCGTAAACATTTCAGGCTGTCCGCCACTGCCTATCGCGATTACAGCTGTGTTGGTTCATTACCTGACGTTTAAGCGTTTCCCTGATCTCGACGAATTACAACGCCCACTCGCTTTTTTTGGT
GAAAGCATCTGTGACAGATGTTATCGACGCCCATTTTTTGAACAACGTAAATTTGCAAAATCGTTTGATGATGAGGGGGCAAAAAATGGGTGGTGTTTGTTCGAACTTGGTTGTAAAGGGCCCGAAACCTTTAACGCATGTGCAACGGTTAAATGGAATCAAGGCACGAG
TTTTTGTATCGAATCTGGCCATCCGTGTCTTGGTTGCTCTGAGCCCGATTTCTGGGATAAAAGCAGCTTTTACCAAGCCTTGGGTCCATGGGAGTGGTACAAATCCAAACCCGGCAAAGGTGCACAGAAGCATGCTGGGAAAAACTCACGTAGCGCTTGGTCCCACCCGC
AGTTCGAAAAATAAGATAATAGGCAAGGCTCATGGAAAATACAGCAAGTAACAACCGGTTAGTCGTCGATCCTATCACTCGAATTGAAGGGCATCTTCGAATAGAAGCTGAAATGGATGGGAATACCATCAAACAGGCGTTCTCATCAGGCACGTCTGTTCGGGGAATTG
AACTGATTTTACAAGGCAGAGATCCGCGTGACGCTTGGGCTTTTGCGCAACGTATCTGTGGCGTCTGCACGCTGGTGCATGGTATGGCATCGGTGCGTGCTGTCGAAGATGCAATTAGAAAAGCTTGGCGGTCAAACGCAAAATTAGGGGTTGCCATTGGAAAGCCCTCC
ATGACATCTATGCCAAAAGGACCGATGCAACATGGTAAAAAAGGGCACCGACAGTCACGTACTTCAATAGGCGTACTGAGTGAAGCAGAAATGGCTATCCCTCAAAATGCACAACTGATAAGAAACATCATGATTGCAACCCAATATGTGCATGATCACGTGATGCATTT
TTATCACTTGCATGCCCTAGATTGGGTTGACGTTGTTTCTGCACTAGATGCAGATCCAACGAGAACCGCTACGCTCGCCGGTCAATTGAGTGATTATCCTCGTTCATCGCCGGGATATTTCAAAGATGTGAAGCAAAAAGTCAAAACGCTGGTTGAGTCTGGGCAGCTAG
GGATATTCAGTAACGCGTATTGGGGGCATCCTGGCTATAAACTGCCACCTGAAGTTAACTTGATGGCATTAGCACATTATTTAGATGCGCTAACGTGGCAGCGTGAAGTTGTAAAAGTTCACACCATATTTGGAGGGAAAAACCCTCATCCTAATTTTGTCGTTGGCGGC
GTGCCTTCACCGATTAATCTCAATGCGTCAACGGGTATTAACACGAGTCGATTAGTGCAACTACAAGATGCTATCACGCAAATGAAGAGCTTTGTCGATCAGGTGTATTACCCCGATATTGTGGCGATTGCGGGTTATTACAAAGAGTGGGGGACACGAGGGGAAGGGCT

GGGTAACTTTCTTACCTATGGAGACTTACCTATGACATCAATGGATGACCCTGATTCTTTCTTGTTTCCACGAGGTGCAATACTTGGTCGAGACTTGAGTAAAGTGCATGACCTTGATCTAGATGATCCCTCTGAAATTCAAGAATTCGTCTCTTCCTCCTGGTATCGAT
ATAGTGGAGGGAACGCAAGTGGTTTACACCCTTTTAATGGACAAACAACACTCGAGTATACTGGTCCGAAACCGCCTTACAAGCACCTAAATGTAGGGGCTGAATATTCATGGTTGAAAAGTCCGCGTTGGAAAGGCCATGCGATGGAGGTGGGACCGCTGGCTCGCGTG
CTAATGATGTATGCTAAAAAAGATGCCGCTGCGCAAGACATCGTTAATCGATCTCTTTCTATCTTGGATTTAGAGACCTCTGCACTTTTCTCCACACTCGGTAGGACGCTCGCCAGAGCAGTGGAAACAAAAATTGTGGTTAACCAGCTACAGTCTTGGTATGACCAACT
ATTGGATAATATCGCAAAGGGCGATACCGATACGTTTAACCCTCTGTATTTTGACCCTACCAATTGGCCAATTAAAGGCCAGGGGGTAGGCGTGATGGAAGCGCCTCGTGGCGCGTTGGGGCATTGGTTGGTCATGCAAAATGGCAAAATTGAGAATTACCAATGTGTCG
TGCCTACGACATGGAACGCTGGACCTCGAGATCCCAACTCACAGGCAGGTGCTTATGAAGCCGCTCTGCAAGATAAACATACGCTACATGATCCTGACCAACCTTTAGAGATTTTGCGAACACTTCATAGTTTTGACCCCTGCTTAGCATGTGCCGTGCACGTAATGGAC
GAAACAGGGGAAGAGCGTTTGCGTCTAAAAGTTCGTTAAgatcctctacgccggacgcatcgtggccggTCGAGCTCAAGGAGGAATAACACATGCGAGAGTAGGGAACTGCCAGGCATCAAATAAAACGAAAGGCTCAGTCGAAAGACTGGGCCTTTCGTTTTATCTGT
TGTTTGTCGGTGAACGCTCTCCTGAGTAGGACAAATCCGCCGGGAGCGGATTTGAACGTTGCGAAGCAACGGCCCGGAGGGTGGCGGGCAGGACGCCCGCCATAAACTGCCAGGCATCAAATTAAGCAGAAGGCCATCCTGACGGATGGCCTTTTTGCGTTTCTACTACT
AGTTGACAATTAATCATCCGGCTCGTATAATGTGTGGAATTGTGAGCGGATAACAATTTCACACATACTAGAAAGAGGAGAAATACTAGATGTGTCTCGCTATCCCCATGAAAGTCATTGCAATTAAAGGCTTTAACGCAACGTGTGAAGCGAAAGGTGTCTCTCGCGAG
GTGAGTTTACATTTAGTGCAAGGGCTAGAGGTCAAAGTTGGCGACTATGTGATGGTACATGTTGGTTATGCTCTCCAAGTGATCACCTATGATGAAGCGCAGGTGACATGGGAGATGCTCGATCAGGTCATTGCTTACGATGCATGAAATTAGTCTTTGCTATAGCTTGC
TTGACACAGTCGCTGTTCACCAGCGAGCAAACATGAATAAGTCTGTCAGTCTGGTTCACGTCAAAGTCGGCCCACTGTCAGGTGTTGAGCCTGACTTGCTGCACCATGCGTTCTTGGCGTGTAGAACTCACACCATTGGCGATCAGGCAACGCTTAGAATTGACACAAGC
GCTATCAAGATACGCTGTAAATTGTGTGGCGAATTCAGCCGAGTTTCGGTGAATAGCATCCTATGTGCAAGTTGCGGTGCTTGGCAAACCGACCTCATTGAGGGCGATGAATTTATATTGCAACGAATAGAGTTTATCGCACCTGACAATTTTACAGAATCAAAGGAGAG
AAAACATGTGCGGTAACTGTGGTTGCGAAACGCAAGTAAAATCAGAGAAAACTGACAAGCCATCAGCAAATATCGTTGAGGTTCACGCAAATTTAAAAAGCAACAATGACAACCAAGCGATAGCAAACCGTGCTTTATTTGACGCACATAATGTACTAGTGATTAATTTG
ATGTCATCCCCAGGCAGTGGAAAAACCAGGCTACTCGAAGAAACGATACGTGCGCTTAAGTCGCAATACGCGATGGCGGTGATTGAAGGCGACCTCGAAACTGAAAATGACGCAAATAGAATTAGACGTCATGGTGTGCAGGCGGAGCAAATCGCCACGGGCCAAGGGTG
TCACTTGGACGCCAGTATGGTGCAAAAGGTACTTGGAAGATTTAGCTTACAAAAACTAGACGTTTTGTTTATAGAGAATGTTGGCAATTTGATTTGTCCAGCGTGTTTTGATTTAGGACAGCACTTAAACATTATTTTGCTGTCCGTACCAGAAGGTGATGATAAGCCTG
AAAAGTACCCAGTGATGTTTCGTGCCGCGGACGTCATGCTTATTTCTAAGACCGATTACTTGCAATTTCACGATGAATTCAATGTACCTCGCGCGATTGAATCATTTCGTAAAGTGGGCAACGATGCGCCCGTTCTCGAAGTGTCTTCATTGAAAAGTCAACATCTAGAG
CGTTGGTTTTCTTATTTTACGCACGCCATTACAGCTCATCAGTCTCAGTCGTTACAAAATAGCGGAGTCTAACATGCGAGAGTAGGGAACTGCCAGGCATCAAATAAAACGAAAGGCTCAGTCGAAAGACTGGGCCTTTCGTTTTATCTGTTGTTTGTCGGTGAACGCTC
TCCTGAGTAGGACAAATCCGCCGGGAGCGGATTTGAACGTTGCGAAGCAACGGCCCGGAGGGTGGCGGGCAGGACGCCCGCCATAAACTGCCAGGCATCAAATTAAGCAGAAGGCCATCCTGACGGATGGCCTTTTTGCGTTTCTACTACTAGTTGACAATTAATCATCC
GGCTCGTATAATGTGTGGAATTGTGAGCGGATAACAATTTCACACATACTAGAAAGAGGAGAAATACTAGATGTACAGTGTTGAATCGTTGCTAAGAGACATTAAGAATACCTCGCTTTCAAAACCGTTTCGTATTCTTAATGTTTGCGGTGGCCATGAGCGTGCGATTA
CGCGCGCTGGATTTAGAACGTTGTTTCAGCACAATATTCATCTGATACCTGGGCCAGGGTGCCCCGTTTGTATTTGCCCCGAAGAGGATATTGCATACGCGATACACTTAGCCATTAACGAAAATGTCGTTATCGTTAGTTTTGGTGACATGCTCCGCGTCCCAGTCGAA
CACGAAATGGGTGGCTGTAGCTCATTGATAGACGCAAAAAATCAAGGGGCCGATATCCGTCCGATATCTTCTCCTCAAGAAGCGGTATCTATAGCATTGGAAGAACCTCGTAAGGTTATTGTGTTTTTTGCTGTTGGTTTTGAAACGACTATGGCACCGATTGCAGCAAC
ACTGCTTAATGATTTACCCAATAACTTTAAAGTATTATTGTCTGGACGACTTACTTGGCCAGCAGTTGCCCATGTGCTTGAGAGTCAACCGAATACTTTTGATGCACTGATTGCGCCAGGCCACGTTGCGACAATTATGGGTAGTGAAGAATGGCAGTTTGCAATCGACC
ATCATAATTTGCCTGTCAGTATTGCTGGTTTTCATCCAGAAAGTTTGCTGTTGTCGCTCCAAACGCTGCTCGGTAATTGTTCAAATAAAGTCGTCACACTGAGTAACCGCTATCCCGAAGTGGTAAAGCAAAACGGCAATGCAGCAGCTAAAGCAATAATCAATAAAGCG
TTCACTATCGTTGACGCACATTGGCGAGGAATTGGTGTTATTCCTGGTTCTGGATTTTCCTTTGCTTCGAGACTATCGCACCTAGACGCCACAAACGATTATGCGCCAGTCGATTTCCCTTCGCAATGTGCGCAAAACGTTCCTGAAACCACATCGCCCTGTGAAAAAGT
GATCCTCGGAAAAATGGCACCTGATGCGTGCCCGTTTTTTGGTCAGGAATGCAAACCAGCATCCCCTAAAGGCGCATGTATGGTGTCTGATGAGGGGGCTTGCCGAATTTGGTATAGCTCTGGTGAGCGCTCGATAACAAATGTTATCAAAAAGGGGAATACGCTAAAGG
TGGAGATGAAGTGATGACAACTGCCCGCACATATGTTGCCAATACGAATAAGAGTAAAACTATCGCCTATGAGTTGAACATTACTGGAGTCATCCAGGGGGTTGGGTTCAGACCGTTAGTTTACTGCTTAGCGTATGAAAAAAGTATTGTTGGTTGGGTACAAAACGACT
GTGGATGTGTGCGAATACACGCTGAAGGCACTGAGCTAGACGTTGAACAATTTGTATTCGACTTACTGAACAATGGGTCGCTAGTTTCCATATCCTTGCTGGAAAAAAAGCAGGTTAAACTGAGTAATGTAGACTCGTTCACGATAAAAGAAAGCGGTCATGATATAGCG
TGCGGGGCGATATCAATCCCCAAAGATGTCTATTTGTGTGGAGCGTGTAAAGCTGAGTTACTGTCAAATACTAACCGACGTGGCACCTATAGCTTTATCGCTTGCAGTAAATGTGGTCCACGATTTTCTATGCTGCGAGCAATGCCATACGACAGAAAGAACATGAGTAT
GTCAGCATTTCCAATGTGTGAGTTATGTCGTAAAGAATATCAATCGCCAACTGATAGGCGCTTTCATGCTCAACCAATAAGCTGTCGCGTATGCGGGCCTGAAGTATTTTGTTCAACTGTCGGCGGGCGCGTGGTCTCGCAAGGCGATGTTGATGTTGTAGCAACGGTTG
TCGCGTATTTGAATCAAGGCAAAATTGTCGCGTTAAAAAGTATAGGCGGTTATCACTTGCTCTGTGACGCACAAAGCACTGAAGCGGTAGACCTTTTACGCCAGCGTAAAAATAGACCAGATAAACCTTTCGCGGTCATGCTCCCAGAGCCTCGTGCAGACGAAACAAGT
GAGGATTGGTTAGATAAATGCGTTGCTGTAGACAGTCATCAAAGAGCGCTCTTACTAAGCCCCGTGCGACCCATTTTACTTGCAAAAAAGCAATCGAATGTACCCATTGCAGATAACGTAGCGCCCATGCTTCGTGATTTGGGGGTGATGTTGCCGTGCAGCGGACTGCA
TTTACTCATTATGCAGCAATTCAATCGCCCCATGGTGGCTACGTCAGGAAATATTTACGGTGAACCGATGGTCACATCGACGTCATCGGCACAGCAACACCTTTCGATGTTGGCAGACATCTTCGTGCACCATAATCGAGACATTTTTCACAAACTGGATGATTCTGTGC
TGCGAACGCTTAAGTCAGGCACCATTCCAATTCGCCTGGGGCGGGGTATAAGTCCGATAGAATTATCATTGCCATATACTGTGCAGGAACCAATGCTTGCTGTTGGCGCGAATCAGAAAAATACAGTCGCAATAGCTTTGAACAATCGACTTATCGTAACACCGCATATT
GGCGACCTCGATTCTCCGATAATGCAAGCGCACTTTGAAAAATGTATCAGTGACTTTCAAGCTCTCTATCATGTTCATCCACAGCGTATTCTTTGCGATAGCCATCCTGGCTATATCAGTTCCCGTTGGGCCAAAGGCAGTGAACTACCGACTACTAAAGTTTTACATCA
CCACGCTCATGCATCAGCGTGGGCATTAGCTGCTGAAATATCGACACCAAGCTTGGTTTTCGTGTGGGATGGAACGGGATTAGGCGAGAATGGCGAGATGTGGGGAGGGGAAGTTTTTTGTGGGACGCCGGGCAATTGGAAACGTGTTGCCTCATTAGCGCCTATGAAGT
TGCAAGGCGGCAACAAGGTTCCAAATCAACCATGGCGAAGTGCAGCATCACTAGTGTGGGGTACTAATTTCACCGGCATTGATTTTAAACCATTTGATCCACAACAATTAAGCTACAACGCATGGCAAAACGAACTCAATTGTCATGTTAGTAGTTCAATGGGAAGACTG
TTCGACGCGGCGGCATTTGCCTGCTGTGAACAGAGCGAAGTCTCTTATGAAGGGCAGGCGCCGATGATGTTGGAAAGCGTTGCGTCGACGCCCTCTAAGGTTATGCCGTTAACGTTGGTCCAGAAAAACGACGAATTTATTCAAATGGATTGGCGAACACTGGTACCGGA
ACTATTTGATTTCAAAGTGTCTCCTCAAACTCGTGCTGCAAATTTCCACGCTTCGTTAATTGAAACGGTATGGGAGGTAACCCTACATTGCCGTAAAAAGTTTAATTTTGACCACATTGGATTGGCAGGAGGCGTCTTCCAAAATCGCATTTTGTGTGACGGTATATCAC
AACGGTTTTTACAATCAGACATTCCTGTGGTTATTCCAAGTAGTTTGCCATTAAACGATGCCGCAATAAGTGTTGGGCAAATCCACGAATACTGTTCACGGAGGTCATAATGAAAAGCGCAGAGGGCAATAATCATTCATCATCAGAGCCGTTGGGCACGGAATCTATTC
AATTAGCTCAGGGGAACGGCGGAACACTGACAAAAAGACTCATTGATCATGTTTTTAAAAATAAAGCGAACAAAGATCTAGATCTGCTTCATGATGCCGCCACAGTAACTTTTGATGGTCCTTATCTGAGTATAACAACGGATAGCTTCGTTGTATCCCCCCCAACATTT
CCAGGCGGCAATGTAGGCGCGTTGAGTGTTTACGGTACCGTCAATGATTTAGCAGTCGTTGGGGCAACACCACGGTACATCAGTACTGCATTTATTATTGAAGAAGGCTTTTCCCTTTCAACATTGAAACAAATCGTTAATGGAATGCATCAAGCTGCCGAAGAAACGAA
GGTCGCCATTGTAACAGGTGATACGAAAGTTGTCCCGAAAGGCAGTGGTGGCGGCGTTTTTATCAATACAACGGGTGTTGGAGACAGCCGAGATGCGCCGATTTGGGATACCAGTCTTATAAAGGCAGGCGATCATGTACTGGTTAGTGGTTCTGTAGGTGATCACGGTG
CGTGTGTCCTGCTTGCTCGCGAGGACTATGGTTTGCAAGGTCAGCTAAAGTCTGATTGTGGAAGCGTTGTTCCTCTTATTGATCCAATCAAGCATTTGGAAGGTGTTCGTTTTGTGCGTGACCCAACTCGTGGAGGGTTGTCAGTATTACTCCATGACGTTGCTAATGAA
ACAGGCTTCGATATCGAGTTAATTGAAAATAATATTCCGGTGCGACCTGAAGTGGCAAGCGTGTGTGAAATATTAGGCTTTGATCCGTTTGTTTTAGCGTGTGAGGGACGCATTGTTGCTGTTGTTGCCCCTGACATAAGCGGTAAGGTGCTAGAACACTGGCGGAGCAT
TCGAAACGGGGAGCAGGCCGAACACATTGGTGTAATAGTAAAAAACACCGAATCAAAAGGGCGCGTGACCATTGTTACGCCAATGGGAGGAAGACGTTTCATGAATGAACTCGAAGATGAGCCGCTTCCAAGGATCTGCTGAGATCCTCTAGAGTCGACCTGCAGGCATG
CAAGCTTGGCTGTTTTGGCGGATGAGAGAAGATTTTCAGCCTGATACAGATTAAATCAGAACGCAGAAGCGGTCTGATAAAACAGAATTTGCCTGGCGGCAGTAGCGCGGTGGTCCCACCTGACCCCATGCCGAACTCAGAAGTGAAACGCCGTAGCGCCGATGGTAGTG
TGGGGTCTCCCCATGCGAGAGTAGGGAACTGCCAGGCATCAAATAAAACGAAAGGCTCAGTCGAAAGACTGGGCCTTTCGTTTTATCTGTTGTTTGTCGGTGAACGCTCTCCTGAGTAGGACAAATCCGCCGGGAGCGGATTTGAACGTTGCGAAGCAACGGCCCGGAGG
GTGGCGGGCAGGACGCCCGCCATAAACTGCCAGGCATCAAATTAAGCAGAAGGCCATCCTGACGGATGGCCTTTTTGCGTTTCTACAAACTCTTTTGTTTATTTTTCTAAATACATTCAAATATGTATCCGCTCATGGCGGCCCAGATCCCCGGGTACCGAGCTCGAATT
TCGAGCTTCTGGAGCAGGAAGATGTCGCGGGCATTAGCACCAGCGGTCTGCCAAGCCTCCGCCAGCCGTTGGGTCCCTTCCGCTTGAGCTTTTCCATCTTCGACGATACGGGCGGCGGCCCCCCGCGCTTCCGCGATCGCCCGTTTACAAGCTGCCTCAGCTGGGGCGAT
CACATCGGCTTGAAGTTGCTGCTGCACCTGTTTGATCCGCTCCTGCTGCACAGGGAGTTCTGCTTGGCTACGAGCGACTTCGGTAGCAATGTCCGCTTCAGCTTCGGCCACCACCGCTTCGCGCCGCGTCAACGCATCCTGAATCCGGCGCTCGGCCTCGGCTTGGGCGA
TCGCTACATCGCGATCGATCCGACGCAGGGCCGTGATCTTGTCATTTTCGGCCGTTTGGATCGCAGAGGCAGCCTGGGCATCGGCTTCAGCAATTCGGGCATCTCGCTGCAGATCAGCCCGCTGCTTGCGTCCACTAGCCGAGAGATAACCGACCTCATCGGAAATGTTC
TGGACTTGCAGCGTATCGAGGACTAGACCCAGCTGCTCAAGGTCATCCTCCGCCTCTTCCAGCAGACTTTTGGCAAAGGCAATTTTGTCCTCGTTGATCTGCTCCGGCGTGAGGCTGGCTAAAACACCACGCAAGTTGCCTTCGAGGGTCTCCTTGGCAATTTGCTCGAT
TTCCTTACGGTTTTTGCCAAGCAGCCGCTCGATCGCGTTGTGGATGGTCGGTTCTTCCCCAGCAATCTTGATATTGGCAACGCCTTCAACAGTCAGGGGAATGCCGCCCTTGGAGAAGGCATTGGAAACGCGCAACTCAATGATCATGTTGGTCAGATCCATGCGGAGCG
CTTTTTCCAGCAGAGGTACCCGCAGGCTGCTGCCGCCCTTGACCAAGCGATAGCCAACTCGGCGGCCATCACTACTGCGGCGACTACTGCCAGCAAAGATCAAAATTTCACTGGGTTGGCAGATGTAGTAGAGATTGCGCAGGACTAAGCTGCCAGCCCCGGCGACTCAC
CAGTCACAGAAAAGCATCTTACGGATGGCATGACAGTAAGAGAATTATGCAGTGCTGCCATAACCATGAGTGATAACACTGCGGCCAACTTACTTCTGACAACGATCGGAGGACCGAAGGAGCTAACCGCTTTTTTGCACAACATGGGGGATCATGTAACTCGCCTTGAT
CGTTGGGAACCGGAGCTGAATGAAGCCATACCAAACGACGAGCGTGACACCACGATGCCTGCAGCAATGGCAACAACGTTGCGCAAACTATTAACTGGCGAACTACTTACTCTAGCTTCCCGGCAACAATTAATAGACTGGATGGAGGCGGATAAAGTTGCAGGACCACT
TCTGCGCTCGGCCCTTCCGGCTGGCTGGTTTATTGCTGATAAATCTGGAGCCGGTGAGCGTGGGTCTCGCGGTATCATTGCAGCACTGGGGCCAGATGGTAAGCCCTCCCGTATCGTAGTTATCTACACGACGGGGAGTCAGGCAACTATGGATGAACGAAATAGACAGA
TCGCTGAGATAGGTGCCTCACTGATTAAGCATTGGTAACTGTCAGACCAAGTTTACTCATATATACTTTAGATTGATTTAAAACTTCATTTTTAATTTAAAAGGATCTAGGTGAAGATCCTTTTTGATAATCTCATGACCAAAATCCCTTAACGTGAGTTTTCGTTCCAC
TGAGCGTCAGACCCCGTAGAAAAGATCAAAGGATCTTC

Figure S1, A representative enzyme activity progress curve over four hours, using the doubly substituted enzyme expressed in BL21ΔH$_4$ cells. Data points represent three technical replicates of an experiment conducted using the procedures described in the methods section. There is some deviation from linearity at 12h, likely due to consumption of reductive potential.

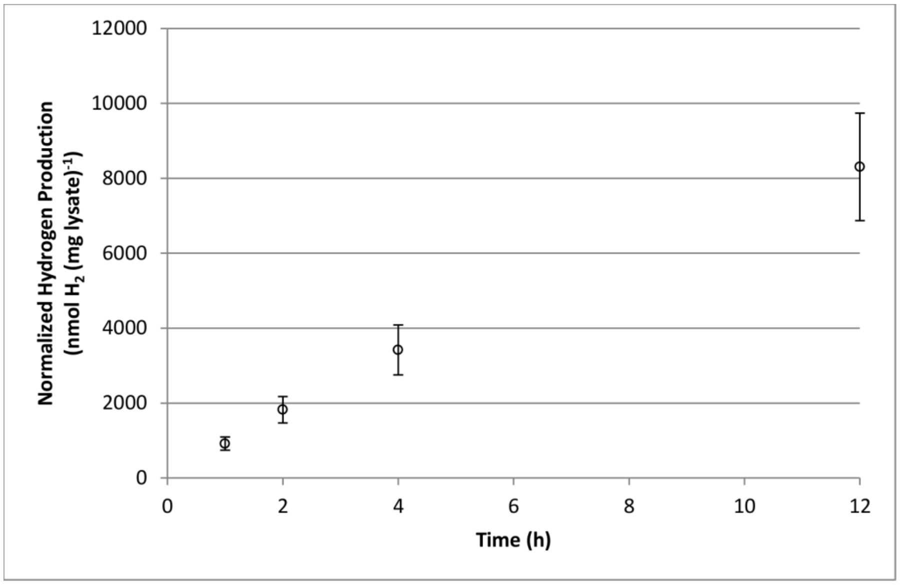

Figure S2, Specific activities of samples from western blot experiment (bars correspond to samples from gel lanes as found in main text Fig. 4)

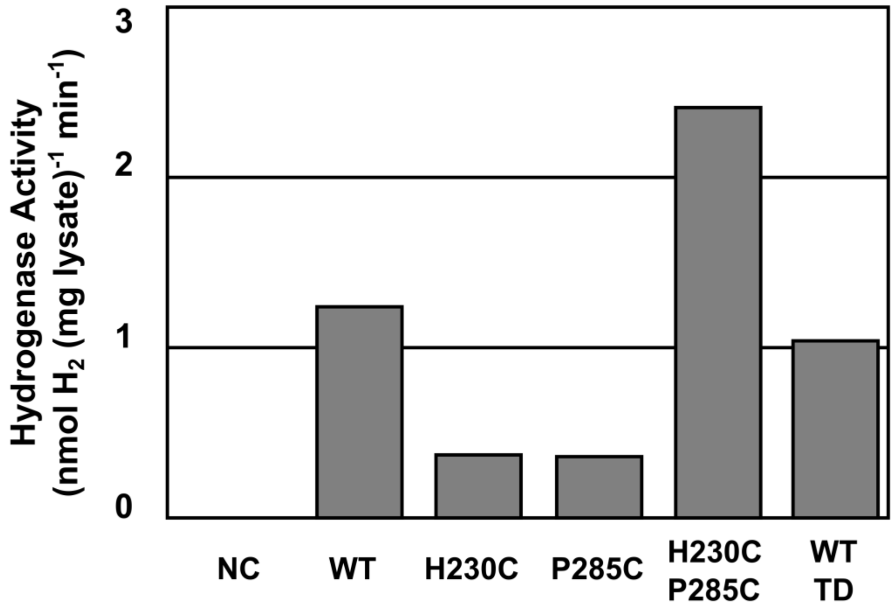

Supplement: Additional file 1: Table S1 — Specific activities of enzyme preparations. Table S2, Primers used in this study. Table S3, (22pp) Maps and sequences for vectors used in this study. Figure S1, A representative enzyme activity progress curve. [file 1754-1611-7-17-S1.pdf]
